# Bis-Tetrazine Fluorogenic (Silicon)-Rhodamine Dyes for Live-Cell Labeling

**DOI:** 10.1021/jacs.6c04723

**Published:** 2026-07-11

**Authors:** Sabrina Giofrè, Lukas Schartel, Richard Wombacher, Edward A. Lemke

**Affiliations:** † Biocenter, Johannes Gutenberg University Mainz, 55128 Mainz, Germany; ‡ Institute of Molecular Biology postdoctoralprogram, 55128 Mainz, Germany; § Departments of Biology and Chemistry, IMPRS on Cellular Biophysic, 55128 Mainz, Germany; ∥ Department of Chemical Biology, Max Planck Institute for Medical Research, 69120 Heidelberg, Germany; ⊥ Institute of Molecular Biology (IMB gGmbH), 55128 Mainz, Germany

## Abstract

Fluorogenic click dyes are valuable tools for biorthogonal labeling, enabling real-time visualization of biomolecules and cellular processes in their native environments. However, achieving efficient quenching and high fluorescence turn-on within a single dye scaffold remains a significant challenge. Herein, we report a class of fluorogenic click dyes based on a structural modification of (silicon)-rhodamines at the amino groups of the xanthene scaffold, resulting in a particularly short linker and a highly optimized quenched state. This modification enables the synthesis of both mono- and bis-functional derivatives. The monofunctional dyes are fully compatible with established click-labeling strategies and display exceptional fluorogenic responses, with fluorescence enhancements of up to 2 orders of magnitude. Notably, the bis-functional derivatives are fluorogenic dyes that exhibit fluorescence turn-on ratios approaching 3 orders of magnitude upon biorthogonal reaction, making them particularly suitable for live-cell applications. We show that the short bis-linker has high potential for anisotropy measurements that can report on protein size and dynamics. We further demonstrate the unique utility of these bis-functional dyes for peptide cyclization, enhancing cellular uptake while enabling real-time visualization. Together, this work introduces a versatile dye class that substantially expands the scope of click chemistry and will advance applications in live-cell imaging as well as studies of protein structure and dynamics.

## Graphical abstract

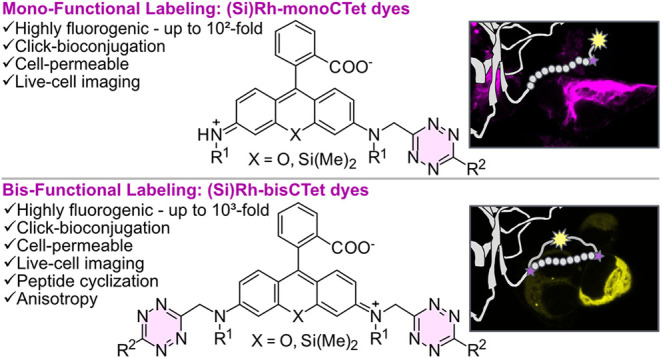

## Introduction

The ability to label proteins with high spatial, temporal, and chemical precision is central to modern chemical biology and cell biology.^1,2^ Fluorescent labeling enables visualization of protein localization, protein–protein interactions, and dynamics in complex biological environments, particularly in living cells.^3,4^ Among available strategies, genetically encoded fluorescent proteins (FPs)^5^ such as GFP and its derivatives, remain widely used due to their ease of implementation and genetic specificity.^6–8^ Despite their broad use, FP-based labeling suffers from intrinsic limitations that limit its suitability for many applications. A key limitation of FPs is their relatively large size (∼25–30 kDa), which can approach or exceed that of many target proteins.^9^ This substantial modification can perturb the protein’s native tertiary structure, alter its interactome, affect its subcellular localization, and interfere with native dynamics.^10,11^ Moreover, fluorescent proteins exhibit limited photophysical tunability.

To overcome these limitations in fluorescent labeling, small-molecule fluorescent probes conjugated via biorthogonal chemistry have emerged as powerful alternatives.^12^ Compared to fluorescent proteins, small-molecule dyes offer superior brightness, photostability, spectral flexibility, and minimal steric perturbation. To harness the advantages of small-molecule fluorescent probes, a variety of self-labeling protein tags^13^ have been developed that undergo selective covalent reactions with appropriately functionalized fluorophores. While comparable in size to FPs, SNAP-tag^14^ exhibits high specificity and rapid labeling kinetics, alongside related systems such as HaloTag^15^ and CLIP-tag.^16^ However, the use of conventional fluorescent dyes introduces their own challenges, including background fluorescence from unreacted probes, limited fluorogenic response upon conjugation, and restricted applicability in live-cell environments. In contrast, fluorogenic probes partially address these challenges by remaining nonemissive until covalent attachment to their target, thereby improving signal-to-noise. To this end, several fluorogenic (silicon)-rhodamines have been developed that exploit the dye’s spirolactone equilibrium, which maintains the dye in a closed, nonfluorescent form until engagement with the protein tag. To maximize fluorogenicity, the self-labeling protein tag is designed to stabilize the dye’s open, fluorescent form.^17–19^

However, those strategies rely on the specific microenvironment provided by the self-labeling protein tag to shift the spirolactone equilibrium toward the fluorescent open form. Consequently, these advantages cannot be readily translated to approaches that aim to bypass bulky self-labeling tags and instead achieve direct labeling of the peptide chain.

Suitable reactive yet biorthogonal chemical handles can be introduced with residue precision in live cells using e.g., protein splicing methods,^20^ or with genetic code expansion technology (GCE).^21,22^ GCE is a highly versatile yet selective strategy that allows the site-specific incorporation of noncanonical amino acids (ncAAs) with single-amino acid precision. In this approach, specific codons, most commonly the amber stop codon, are reassigned through engineered orthogonal tRNA/tRNA synthetase pairs evolved to selectively incorporate the desired ncAA at a defined position within the protein. By incorporating an ncAA bearing a strained alkyne or alkene, it becomes possible to exploit the inverse electron-demand Diels–Alder (iEDDA)^23,24^ reaction with tetrazines, enabling fast reaction kinetics. At the same time, the tetrazine moiety can act as an efficient fluorescence quencher when conjugated in a suitable way to the dye of interest, thereby providing a fluorogenic response upon reaction.

Most reported live-cell tetrazine-based fluorogenic dyes exhibit high fluorogenicity in the green-emitting spectral region (Figure 1, top, tetrazine-conjugated label),^25–30^ but with modest fluorescence turn-on ratios approaching the red or far-red regions. This is where efficient quenching is known to be increasingly challenging and typically results in only modest fluorescence enhancements for silicon-rhodamine dyes (1–4-fold,^26,31^ and rarely up to ∼50-fold^32^). Although monochromophoric designs have demonstrated outstanding 2 orders of magnitude turn-on fluorescence,^33–37^ including the red and far-red regions, their application in live-cell imaging remains limited in some cases.

**Figure 1:**
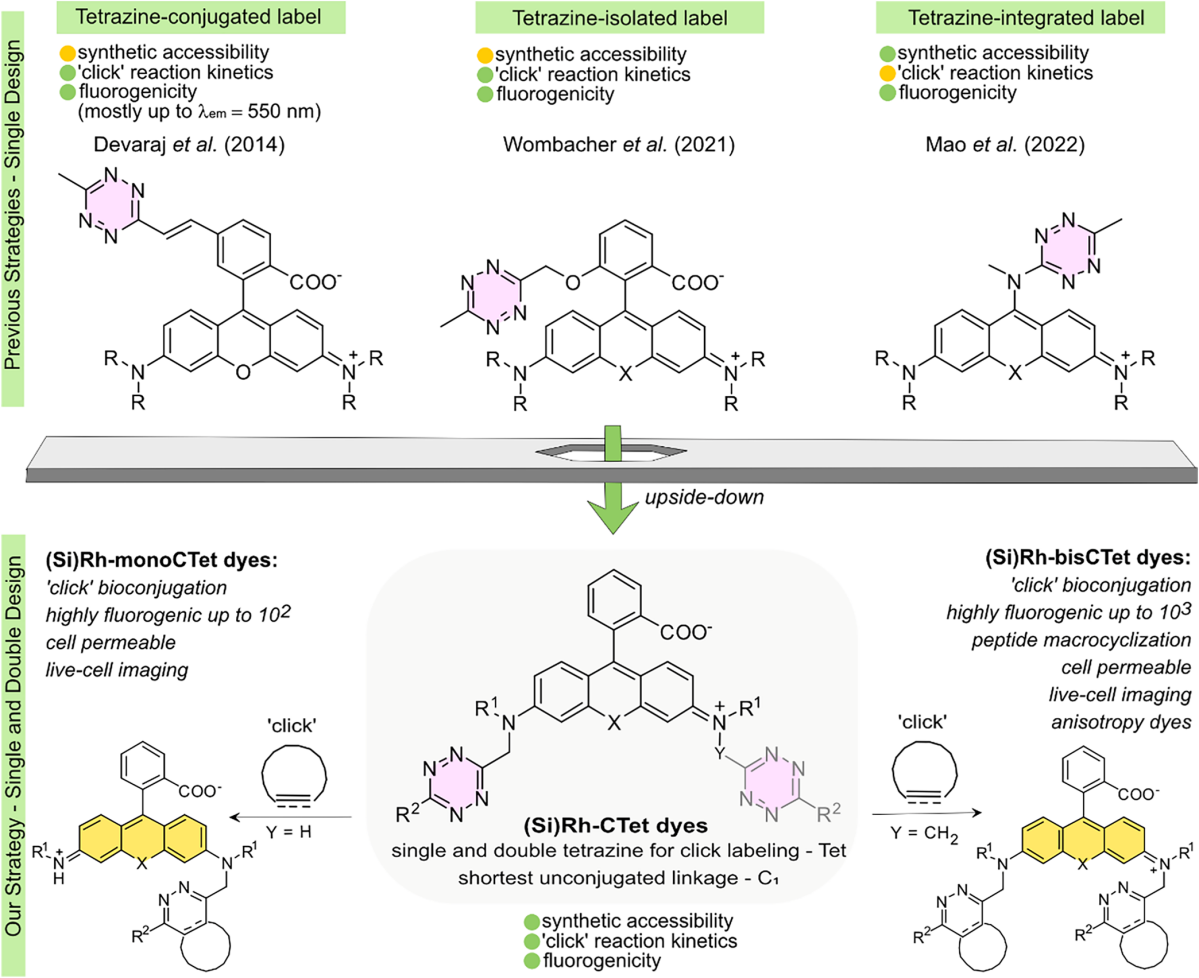
State-of-the-art fluorogenic (silicon)-rhodamines incorporating a single tetrazine moiety, that serves as a fluorescence quencher and as a biorthogonal “click” handle (top). Rational design of the new mono- and bis-functional (silicon)-rhodamine dyes, (**Si)­Rh-monoCTet** and (**Si)­Rh-bisCTet**, respectively (bottom).

This is largely because excitation often requires UV light,^33–35^ and the presence of a nitrogen atom adjacent to the tetrazine^36^ can slow reaction kinetics, potentially hindering rapid labeling in live cells (Figure 1, top, tetrazine-integrated label). Moreover, the design of those fluorogenic dyes is mostly limited to monofunctional derivatives (Figure 1, top). Although such monofunctional dye designs are generally easier to implement than dual-tagging strategies, the dual-tagging approach may offer advantages in applications where minimizing background fluorescence is particularly important. Because full fluorescence enhancement requires the cooperative engagement of two ncAAs positioned at defined sites, this strategy has the potential to reduce off-target labeling commonly observed in GCE systems and thereby improve the overall signal-to-noise ratio.

Furthermore, a bis-functional approach offers particular advantages in fluorescence anisotropy experiments.^38^ Fluorescence anisotropy is a powerful technique for probing protein size, conformations, assemblies, and dynamics.^39,40^ This is achieved by measuring the polarization status of the dye. The more rigid the dye is attached to the protein, the more directly the readout reflects the protein’s behavior. As monofunctional dyes typically retain rotational freedom around the attachment linker, a substantial loss in anisotropy resolution is often observed.

Another powerful application of bis-functional dyes lies in their ability to act as efficient bridging scaffolds, enabling end-to-end conjugation when complementary biorthogonal handles are strategically positioned at the termini of biomolecules, including peptides. In the literature, there are relatively few examples of cyclic peptides in which the fluorescent chromophore is integrated into the cyclic backbone,^41–44^ and additionally fluorescence detection usually relies on ultraviolet excitation (350–400 nm), which is associated with phototoxicity and may limit live-cell compatibility. Therefore, the use of bright fluorophores excitable with visible light is desirable and can further facilitate detection at lower peptide concentrations in a cellular context while improving signal-to-noise ratios.

In order to achieve a dual-tetrazine molecular design, we employed a conceptually distinct “upside-down” approach in which the tetrazine moiety was positioned in close proximity to the fluorophore via an unconjugated linkage on the amino group of the xanthene core. This differs from other systems, in which the lower ring of the rhodamine structure is typically modified (Figure 1, top vs bottom). We reasoned that this shorter and less flexible linkage would increase quenching efficiency prior to the biorthogonal reaction by enhancing interactions between the fluorophore and the tetrazine moieties. This molecular design enables synthetic access to both mono- and bis-functional dyes within a single chemical platform. While the monofunctional dyes provide robust fluorogenicity for conventional click labeling, the bis-functional derivatives unlock additional capabilities by covalently constraining dye motion and enabling diverse applications.

We demonstrate that this compound class supports fluorescence turn-on ratios approaching three orders of magnitude, enabling applications in peptide macrocyclization, live-cell labeling of proteins, and anisotropy-based measurements of protein rotational dynamics.

## Results and Discussion

### Rational Design of Mono- and Bis-Fluorogenic Dyes with Enhanced Quenching Efficiency

The development of fluorogenic dyes requires tailoring of multiple, often competing molecular features within a single molecular scaffold, including efficient quenching in the unreacted state, brightness, suitable spectral characteristics, photostability, and compatibility with live-cell imaging. An effective design should further enable modular chemical modification of the fluorophore, allowing fine-tuning of photophysical properties and the incorporation of additional functionalities.

To address this challenge, we synthesized mono- and bis-functionalized tetrazinyl rhodamine (Rh) and silicon–rhodamine (SiRh) fluorogenic dyes in which the interchromophore distance between tetrazine and fluorophore is minimized (Figure 1, bottom), labeled as **(Si)­Rh-CTet** dyes. Specifically, compound designation was assigned based on the xanthene core (Rh or SiRh), nature of the linker between tetrazine and xanthene core (C_1_) and the tetrazine itself (Tet). This molecular design allows systematic modulation of the xanthene scaffold through heteroatom substitution, providing access to dyes spanning multiple spectral regions. Importantly, our compounds maintain a spirolactone equilibrium between closed and open forms, which is advantageous for live-cell imaging because the closed, nonfluorescent form can cross the membrane via passive diffusion.^45^ In addition, the carboxyphenyl ring offers several positions for further substitution, facilitating downstream functional diversification. Substitution at the amino groups enables fine-tuning of absorption and emission wavelengths as well as fluorescence brightness (Figure 1, substituent = R^1^). Furthermore, late-stage functionalization with the tetrazine moiety enables the introduction of additional substituents without significantly perturbing the core photophysical properties, thereby providing a modular and flexible design strategy (Figure 1, substituent = R^2^). From a synthetic perspective, the xanthene scaffold can be prepared on a gram scale from phthalic anhydride and aminophenol, while the silicon-rhodamine analogue is obtained through a six-step synthesis involving temporary allyl protection, removed in the final step prior to functionalization with bromomethyl tetrazine (see Scheme S1). The bromomethyl tetrazine was synthesized following a reported procedure by Mao et al.,^46^ followed by demethylation with BBr_3_ and subsequent bromination as described by Werther et al.^32^ (Scheme 1a). The late-stage nucleophilic substitution was conducted at 0 °C in the presence of Cs_2_CO_3_ to favor the closed spirolactone form of the Si- and O-rhodamine core and to activate the weakly nucleophilic aniline amino group. By adjusting the stoichiometry of the bromomethyl tetrazine and of the base, mono- and bis-functionalized derivatives could be obtained (Scheme 1b). The nucleophilicity of the amino group was found to play a key role in these transformations. Si-rhodamine derivatives exhibited higher reactivity, whereas rhodamine analogues showed reduced amino-group nucleophilicity. In function of the degree of tetrazine substitutions (mono or bis), compounds were further designated as **(Si)­Rh-monoCTet** and **(Si)­Rh-bisCTet dyes**. Overall, this design establishes a general strategy for positioning tetrazine quenchers in close proximity to rhodamine-based fluorophores while retaining modularity and features desirable for live-cell imaging.

**Scheme 1:**
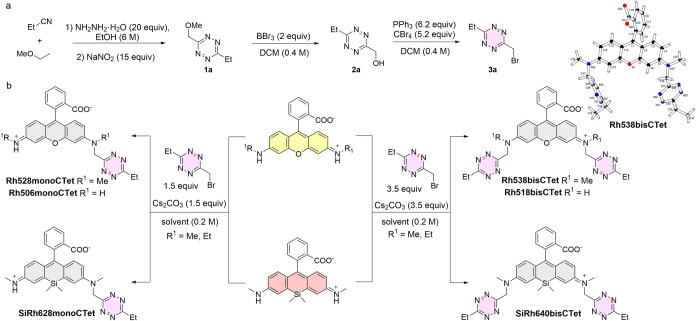
(a) Synthetic Route for Accessing Bromomethyl Tetrazine. (b) Synthetic Approach Based on a Late-Stage Functionalization of the Amino Groups with Tetrazine to Provide Mono- and Bis-Functional Dyes, (**Si)­Rh-monoCTet** and (**Si)­Rh-bisCTet**; CCDC 2548790 Contains the Supplementary Crystallographic Data of **Rh538bisCTet**

### Spectroscopic Properties

With synthetic access to the mono- and bis-functionalized tetrazine dyes established, we next investigated their spectroscopic properties. Absorption (λ_abs_) and fluorescence emission (λ_em_) maxima, extinction coefficients (ε), quantum yields (Φ_F_) and fluorescence lifetime (τ_ns_) were determined before and after reaction with (bicyclo[6.1.0]­non-4-yn-9-yl)­methanol (BCN–OH), through iEDDA reaction (see Figure 2a,b for normalized absorbance/emission spectra and Table 1 for spectroscopic values). BCN was selected as the model dienophile due to its desirable properties, including high chemical stability, formation of a single cycloaddition product, and well-defined reaction kinetics.^47–50^

**Figure 2:**
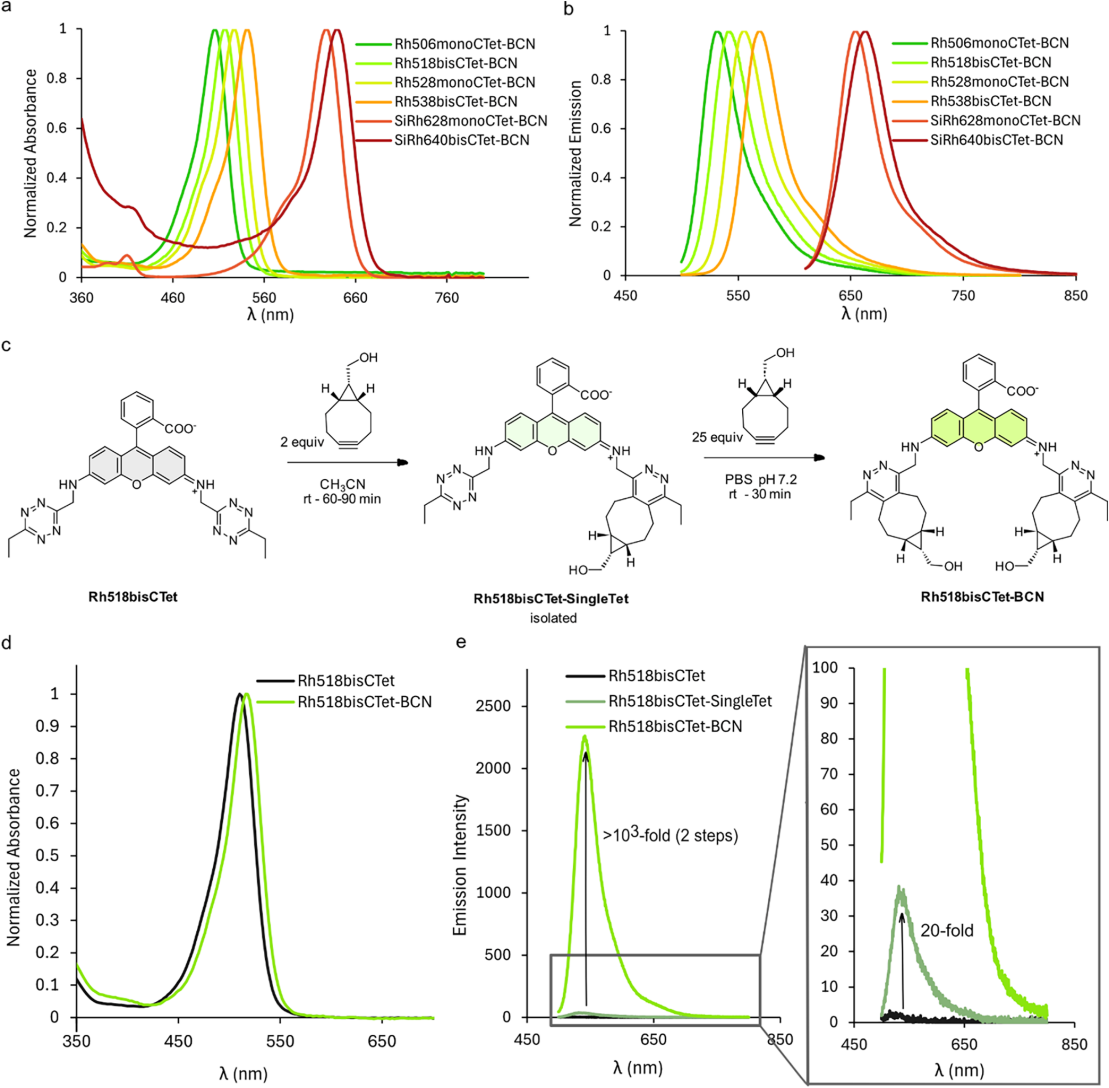
Spectral properties of (Si)­Rh-CTet upon click reaction. (a) Normalized absorbance of (Si)­Rh-CTet after click reaction. (b) Normalized emission of (Si)­Rh-CTet after click reaction. (c) Click reaction between **Rh518bisCTet** and 2 equiv of BCN–OH to provide the mono clicked intermediate **Rh518bisCTet-SingleTet**, followed by incubation with 25 equiv BCN–OH in PBS (pH = 7.2) to obtain the fully converted bis-derivative. (d) Normalized absorption spectra of **Rh518bisCTet** and **Rh518bisCTet-BCN**, indicating moderate bathochromic shift after click reaction with BCN–OH. (e) Emission spectra of **Rh518bisCTet, Rh518bisCTet-SingleTet** and **Rh518bisCTet-BCN** showing the contribution of each tetrazine to the fluorogenic response after click reaction with BCN–OH.

**Table 1:** Spectral Properties of (Si)­Rh-monoCTet and (Si)­Rh-bisCTet Dyes^a^,^b^,^f^

compound	λ_abs_ [nm]	λ_em_ [nm]	ε [10^4^ (M cm)^–1^]	Φ_F_	τ [ns]^e^	turn-on
**Rh506monoCTet**	504	525	7.5	0.021	3.8	13^c^, 11^d^
(+BCN–OH)	(506)	(530)	(7.0)	(0.52)		
**Rh528monoCTet**	526	550	7.9	0.03	3.5	7^c^, 8^d^
(+BCN–OH)	(528)	(555)	(7.7)	(0.32)		
**SiRh628monoCTet** ^b^	626	649	30.7	0.0002	3.2	60^c^, 279^d^
(+BCN–OH)^b^	(628)	(655)	(28.5)	(0.43)		
**Rh518bisCTet**	512	527	3.9	0.0005	3.7	1144^c^, 1435^d^
(+BCN–OH)	(518)	(541)	(3.1)	(>0.98)		
**Rh538bisCTet**	534	549	7.7	0.003	3.3	71^c^, 140^d^
(+BCN–OH)	(538)	(567)	(7.6)	(0.82)		
**SiRh640bisCTet** ^b^	630	655	3.1	0.01	n.d	32^c^, 32^d^
(+BCN–OH)^b^	(640)	(663)	(4.5)	(0.17)		

a All measurements were performed in phosphate-buffered saline (PBS, pH 7.2) and turn-on experiments with BCN (25 equiv). Properties of the respective BCN-cycloaddition products are given in parentheses (see Figure S1–S6 for all structures and spectra).

b 0.1% SDS in PBS pH 7.2 was used.

c For the turn-on calculation the area under the curve has been considered.

d For the turn-on calculation the emission maxima intensity has been considered.

e Fluorescence lifetime has been calculated on the BCN-clicked adduct.

f Each synthesized dye was named based on its absorbance maximum above 500 nm.

The monofunctionalized rhodamine derivatives exhibited moderate fluorogenic responses. For example, **Rh506monoCTet** and **Rh528monoCTet** displayed a 13- and 7-fold fluorescence enhancement upon reaction with BCN, respectively, whereas the red-shifted analogue exhibited a substantially higher fluorescence turn-on of up to 60-fold or 279-fold if the maximum intensity was considered, a notable value for dyes emitting in the red and far-red spectral regions.^31–33^ Importantly, the introduction of a second tetrazine markedly increased fluorogenicity: **Rh518bisCTet** exhibited a 10^3^-fold fluorescence enhancement (Table 1). Likewise, **Rh538bisCTet** exhibited a 140-fold turn-on. In order to observe the effect after each click reaction on the fluorescence emission, we selectively generated the monoreacted intermediate **Rh518bisCTet-SingleTet** from **Rh518bisCTet** by treatment with 2 equiv of BCN–OH (Figures 2c and S7).

Comparison of the unreacted dye with the monoreacted species revealed a ca. 20-fold fluorescence enhancement, while conversion from the monoreacted to the bis-cycloadduct led to an additional ca. 60-fold increase. In the case of the bis-functionalized silicon-rhodamine derivative **SiRh640bisCTet**, we observed the prevalence of the spirolactone closed form. Accordingly, pH-dependent absorption and emission measurements were performed (see SI, Figure S8). At pH 3.2, **SiRh640bisCTet** was exclusively in the open form; at physiological pH, around 12% of the zwitterionic open form was detected, resulting in reduced brightness. Notably, the fluorescence turn-on observed at acidic pH was substantially higher, reflecting increased emission from the BCN cycloadduct while the tetrazine precursor remained strongly quenched (see SI, Figure S8). Due to the more hydrophobic nature of Si-rhodamine compared to O-rhodamine, the development of more hydrophilic variants,^51^ as well as new derivatives lacking the spirolactone group, could expand the repertoire of bifunctional dyes in the red and far-red spectral region. The fluorogenic behavior of these dyes was envisioned to rely primarily on a Dexter-type quenching mechanism,^52^ which requires close proximity between the fluorophore and the tetrazine quencher to enable short-range orbital interactions. Upon reaction with BCN, the highly electron-deficient tetrazine is converted into the less quenching pyridazine, thereby restoring fluorescence.^53^ To experimentally probe this distance dependence, we synthesized the **SiRh628monoCTet** analogue, featuring an extended C3 spacer between the two moieties, named as **SiRh634monoC3Tet**. This elongation of the linker resulted in a pronounced decrease in fluorogenic response: while **SiRh628monoCTet** (C1 linker) exhibited a 279-fold fluorescence enhancement, **SiRh634monoC3Tet** showed only a 3.4-fold increase (see Figure 3). This marked difference supports a short-range, distance-dependent quenching mechanism consistent with Dexter-type exchange^52^ and/or tetrazine-promoted intersystem crossing. In addition, **SiRh628monoCTet** displays a larger Stokes shift (26 nm) compared to its analogue **SiRh634monoC3Tet** (19 nm) (Figure 3). This difference is the result of the modification of xanthene’s amino groups with tetrazines, which consistently induced hypsochromic shifts in the absorption spectra of both mono- and bis-functionalized derivatives, typically on the order of ∼10 nm compared to structurally related O-rhodamine and Si-rhodamine dyes bearing similar *N*-substitution patterns. The hypsochromic shift observed in the absorption spectrum is most likely attributable to the strong electron-withdrawing (−I) effect of the tetrazine moiety.^54^ Following the biorthogonal click reaction, the absorbance maxima undergo bathochromic shifts (Figures 2d and S9), which are pronounced for the bis-derivatives (Δλ_abs_ ≈ 6–10 nm) and less significant for the monoderivatives (Δλ_abs_ = 2 nm), and consistent with the distinct electronic structures of tetrazine and the corresponding pyridazine adduct. Taken together, the observed ground-state spectral perturbations support partial electronic coupling between the tetrazine and the fluorophore, consistent with a proximity-enabled nonradiative quenching mechanism, such as Dexter-type exchange and/or tetrazine-promoted intersystem crossing. On the other hand, an intramolecular photoinduced electron transfer (PET) pathway was ruled out, as no solvent-dependent effects on fluorescence emission were observed (Figure S10).^25,55^ Importantly, this strong quenching efficiency is maintained under biologically relevant conditions: although partial cleavage of the tetrazine pendant was observed for the bis-functionalized dyes upon prolonged incubation in PBS. Stability studies revealed that around 5% cleavage occurred after 2 h at ambient temperature (Figure S12). This ratio stayed quite constant up to 24 h, but becomes more pronounced after 3 days. On the other hand, reduced stability was observed within hours when the dye was incubated in enriched aqueous media such as high-glucose DMEM supplemented with fetal bovine serum (FBS). In contrast, improved stability was maintained in serum-free DMEM (FluoroBrite), with the dye remaining stable for up to 3 h at ambient temperature, a time scale that is relevant for prospective live-cell and in vivo applications. Conversely, the corresponding BCN cycloadducts exhibited excellent stability, with no detectable degradation observed under any of the tested conditions for up to 9 days. The observed species likely originates from degradation of the rhodamine-like scaffold, potentially driven by tautomerization and charge delocalization within the xanthene core. In this process, the tetrazinyl substituent may act as a better leaving group than the corresponding pyridazinyl analogue, thereby facilitating the transformation.^56^ This instability is characteristic of systems containing reactive or labile groups, and in particular tetrazinyl moieties, which are known to undergo degradation or side reactions in complex biological media.^57,58^

**Figure 3:**
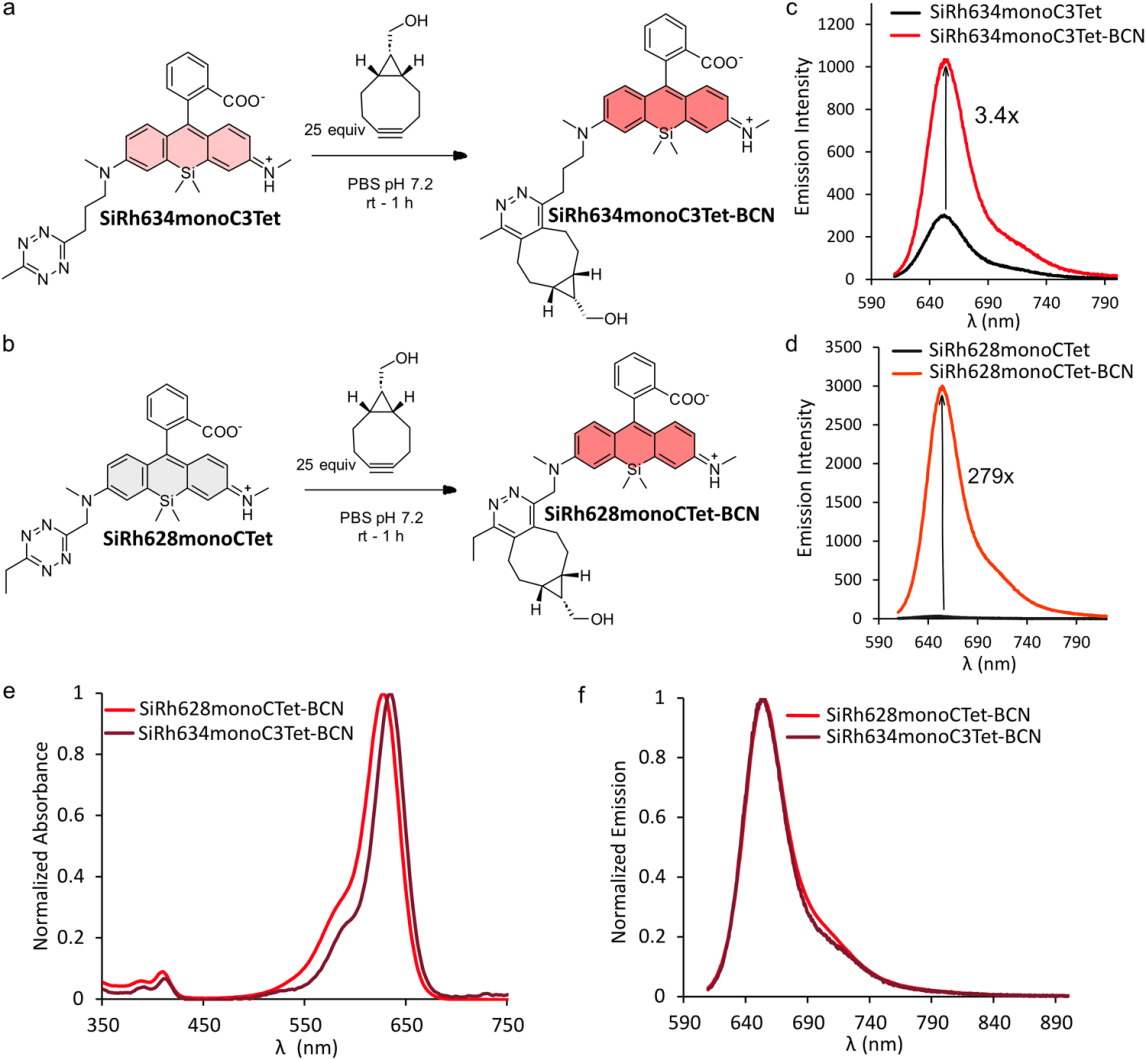
Linker effect on fluorescence quenching. (a) To test for a Dexter-type quenching the derivative with a longer linker was synthesized, **SiRh634monoC3Tet**. (b–d) **SiRh634monoC3Tet** displayed poor turn-on compared to its shorter derivative **SiRh628monoCTet** (3.4- versus 279-fold fluorescence enhancement, respectively, calculated in function of peak intensity). (e) Normalized absorbance after click reaction, showing the difference in absorbance maxima wavelength between the shorter derivative, **SiRh628monoCTet** (λ_max_ = 628 nm) and the longer one, **SiRh634monoC3Tet** (λ_max_ = 634 nm). (f) Normalized emission after click reaction, showing the less pronounced difference in emission maxima between **SiRh628monoCTet** (λ_max_ = 654 nm) and **SiRh634monoC3Tet** (λ_max_ = 653 nm).

### Bis-Functionalized Dyes for Peptide Macrocyclization

To evaluate the applicability of the bis-functional dyes for peptide and protein dual-click, a series of peptide sequences bearing two BCN moieties at defined positions were designed (Table 2). In all cases, the BCN group was linked to the *ε*-amino group of lysine via its hydroxy group as carbamate. The sequences of peptides Pep1 and Pep5 were adapted from previous studies on bifunctional dyes evaluated *in vitro*,^59–61^ in which near-quantitative fluorescence enhancement relative to the linear adduct control was reported. In addition, we designed further peptides (Pep2–4, 6–8) with either shorter or longer sequences to modulate backbone flexibility and secondary structure propensity, ranging from glycine/serine-rich to more α-helix–favoring glutamate/aspartate-rich sequences. Spacings between the two BCN-modified residues were varied from three amino acid units (*i,i* + 4) in Pep8 to 11 amino acid units (*i,i* + 12) in Pep4 to assess the influence of intramolecular distance and conformational freedom on macrocyclization efficiency and fluorogenic response. iEDDA reaction between all the peptides and **Rh518bisCTet** was analyzed by HPLC coupled to ESI-MS.

**Table 2:** Peptide Sequences Investigated in the Click Reaction with Rh518bisCTet

click partner	sequence	turn-on	*k(k*_1_ & *k*_2_ *)* s^–1^ M^–1^
**BCNK**	-	>1400	4.00
**Pep1**	K(BCN)**AEAADAEAA**-K(BCN)	1524	42.6 ± 7.3
**Pep2**	K(BCN)**GSAAGSA**-K(BCN)	1297	74.0 ± 7.2
**Pep3**	K(BCN)**GSAAEAA**-K(BCN)	1423	37.8 ± 1.4
**Pep4**	K(BCN)**ADAAEADAAEA**-K(BCN)	1366	12.2 ± 1.3
**Pep5**	K(BCN)**AAEA**K(BCN)	643	n.d.
**Pep6**	K(BCN)**ADAAEA**K(BCN)	1009	n.d.
**Pep7**	K(BCN)**GGSG**K(BCN)	1004	n.d.
**Pep8**	K(BCN)**GSG**K(BCN)	1287	n.d.

In all cases, the dominant detected species indicated a 1:1 stoichiometric reaction and the efficient formation of cyclized products, suggesting a strong preference for intramolecular second cycloaddition under the tested conditions (Figures S15–S22). Notably, all cyclic peptide conjugates remained stable for at least 25 days in aqueous/acetonitrile mixtures (Figures S15b–22b).

Having established the intramolecular reaction as the preferred pathway, fluorescence enhancement upon click was then investigated. Among the peptides tested, reaction of Pep5 with **Rh518bisCTet** resulted in the lowest fluorescence enhancement, reaching approximately half of the maximal turn-on observed for the linear reference (**Rh518bisCTet-BCN**) (Table 2 and Figure 4c).

**Figure 4:**
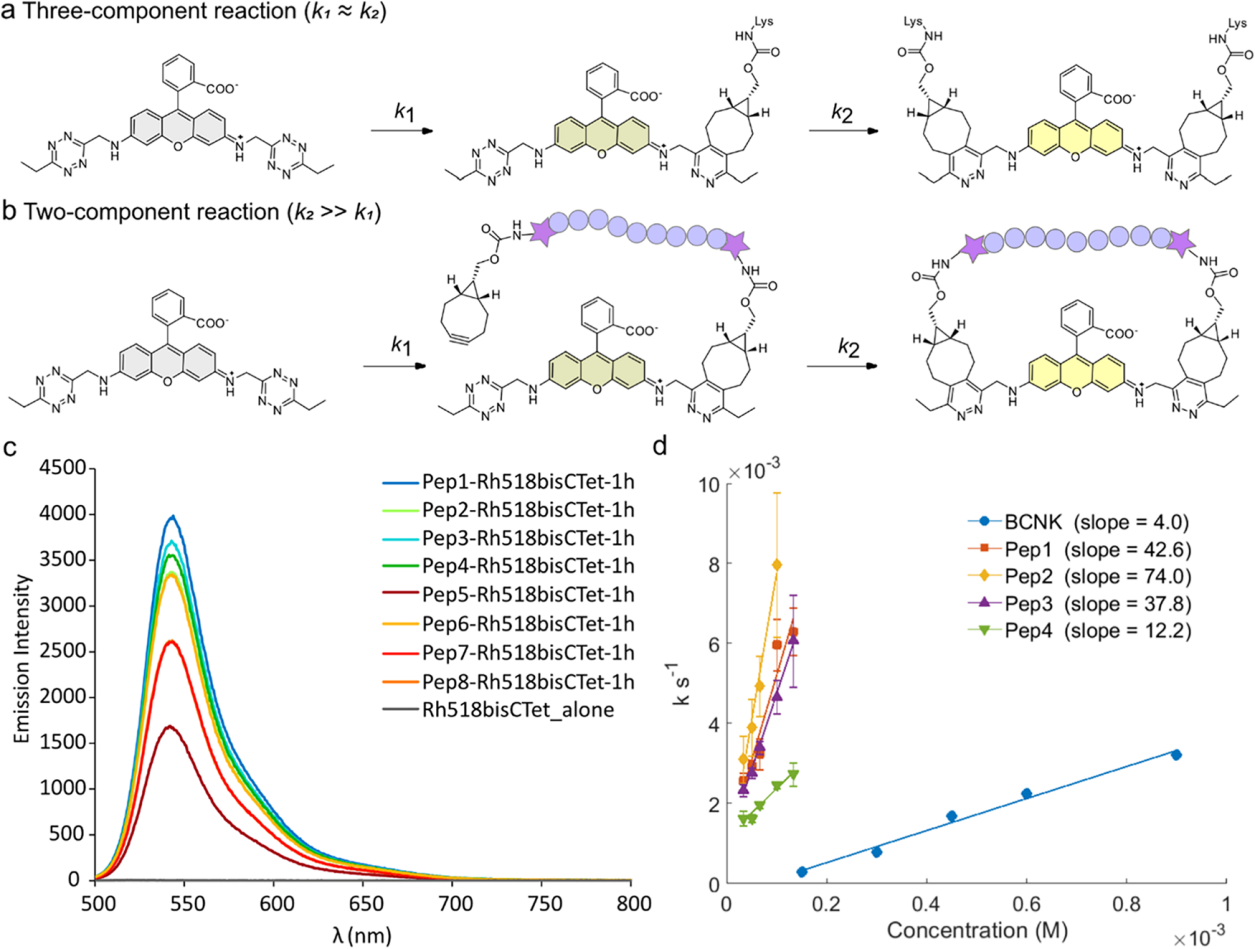
Click reaction studies on peptides bearing BCN as chemical handle. (a) Click reaction on **Rh518bisCTet** via a three-component reaction. (b) Click reaction on the **Rh518bisCTet** via a two-component reaction. (c) Fluorescence enhancement upon click reaction with Pep1–8. (d) Apparent reaction kinetics between **Rh518bisCTet** and BCNK or Pep1–4.

Conversely, the remaining peptides exhibited fluorogenic responses comparable to those obtained for the linear reference, with Pep1 showing the most pronounced response among this group (Table 2 and Figure 4c). To further probe the preference for intra- versus intermolecular reactions, kinetic experiments were performed using BCN-lysine (BCNK) and selected BCN-modified peptides (Pep1–Pep4). Pseudo-first-order rate constant were achieved by employing an excess of BCN-containing peptide (5 up to 40 equiv of peptide relative to the dye) or BCNK (60 up to 360 equiv of BCNK relative to the dye). However, at peptide loadings above 40 equiv intermolecular side reactions became apparent; therefore, all experiments were conducted using no more than 40 equiv. Additionally, because the reaction shown in Figure 4a involves three components, the effective BCNK concentration was adjusted on an equivalent-reactive-handle basis to allow direct comparison with reactions involving mono- and bis-functional substrates. This correction avoids systematic underestimation of the apparent rate constants for BCNK. We experimentally measured an observed rate constant *k*_obs_, which reflects the kinetically dominant process governing the overall reaction. For BCNK, the reaction is expected to proceed through two independent, kinetically comparable pathways (*k*_1_ ≈ *k*_2_), such that no single pathway is strongly rate-limiting and the observed kinetics report on an effective rate constant of product formation (Figure 4a). Conversely, for a two-component system involving a bis-functional substrate, an increase in the apparent rate constant requires that the second cycloaddition proceed more rapidly than the first (*k*_2_ > *k*_1_) (Figure 4b). Consistent with this assumption, the rate constant for the second cycloaddition (*k*_2_) was significantly higher than the first (*k*_1_) throughout all peptides. This indicates that once the initial cycloaddition positions the remaining BCN group in close proximity to the second reactive site, the consecutive intramolecular reaction proceeds more rapidly. The highest apparent rate constant was observed for Pep2 (apparent *k* = 72 M^–1^ s^–1^), consistent with its increased conformational flexibility. In contrast, Pep4, featuring the longest sequence and biggest separation between BCN groups exhibited markedly slower kinetics (Table 2 and Figure 4d). Because intramolecular cyclization is favored over intermolecular cross-linking, this system addresses an important requirement for live-cell applications, where minimizing cross-reactivity between distinct proteins is desirable. Building on these promising results, we next investigated the behavior of the resulting cyclic peptides in cellular environment.

### Cell Treatment with Cyclic and Linear Peptides

Stapled and cyclic peptides are well established to exhibit improved properties relative to their linear counterparts, including enhanced resistance to proteolytic degradation, increased membrane permeability, and improved target engagement because of conformational preorganization.^62^ These advantages have contributed significantly to the growing interest in peptides as drug candidates.^63–65^ Recent advances in metal-free peptide cyclization strategies have further expanded the accessible chemical space while improving biocompatibility.^66,67^ Copper-free click chemistry has previously been explored for peptide and protein stapling in biological environments. Spring and co-workers reported strain-promoted azide–alkyne cycloaddition (SPAAC) using Sondheimer dialkynes^68,69^ as a stapling strategy that can be performed directly in cell culture and enables parallel screening of cell-active stapled peptides. However, the broader biological applicability of this approach is limited by the poor aqueous solubility and stability of the Sondheimer dialkyne (*t*_1_/_2_ ≈ 10 min at pH = 7.4), and despite subsequent improvements, these constraints continue to restrict its general use. Kele and co-workers also developed bis-azide cyanine dyes that undergo SPAAC-mediated peptide cyclization, resulting in fluorescence enhancements of up to 39-fold.^59,60^ However, further biological investigation were not reported.

Here, we sought to demonstrate, as a proof of concept, the applicability of our bis-functionalized dyes for advancing peptide cyclization by iEDDA chemistry,^61^ known for its fast kinetics and better compatibility with living systems. This approach enables peptide cyclization while simultaneously providing a built-in fluorescent readout using visible-light excitation.^70^ To assess its feasibility, we monitored the cellular uptake of cyclic peptides by fluorescence microscopy. Pep1 was selected as the representative cyclic peptide, and Pep9, the corresponding linear analogue bearing only a single BCN moiety, serving as a reference (Figures 5a and S23).

**Figure 5:**
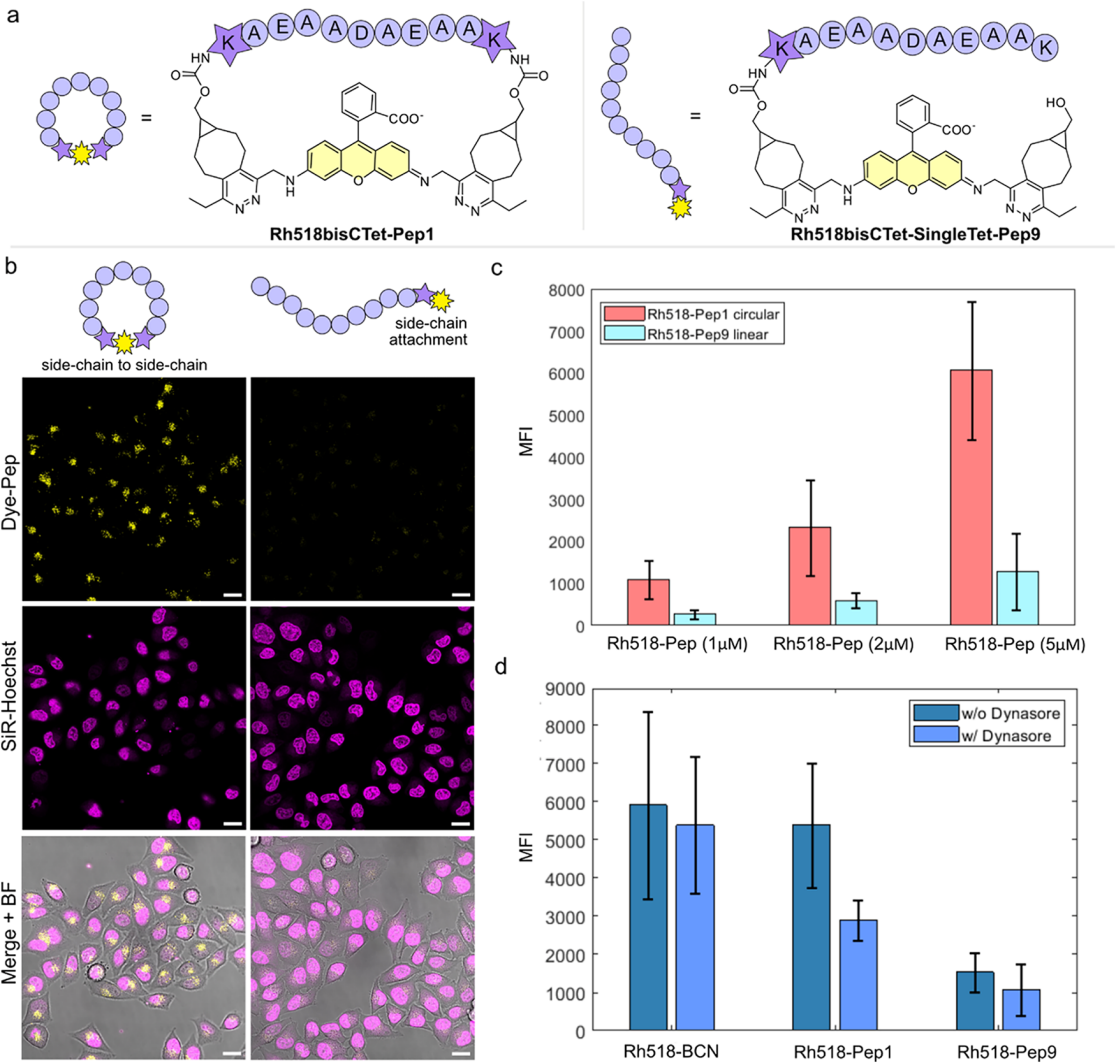
Confocal microscopy and flow cytometry of HeLa cells incubated with Rh518-Pep conjugates. (a) Schematic structure and primary sequence of the peptide-dye adduct tested. (b) Confocal microscopy images of HeLa cells treated with 4 μM Rh518–Pep conjugates, including **Rh518bisCTet**-Pep1 and the monoclicked control **Rh518bisCTet-SingleTet**-Pep9. Conjugates were prepared by reacting the dye with 5 equiv of Pep1 (for the cyclic peptide) and Pep9 (for the linear peptide), respectively, in PBS or DMEM buffer for 2–3 h to ensure complete conversion prior to the addition to the cell culture medium, as monitored via LC-MS (see Figure S26). Cells were incubated for 3 h at 37 °C and subsequently stained with SiR-Hoechst for nuclear visualization. Scale bar 20 μm. (c) Median fluorescence intensity (MFI) as a function of conjugate concentration, shown as bar plots from three independent replicates, after subtraction of the signals obtained from untreated samples (the 488 nm laser with a 532/30 nm bypass filter was used; note, this setup is suboptimal for detecting Rh518). (d) MFI of HeLa cells treated with 5 μM of dye-peptide conjugate, in the absence or presence of Dynasore (80 μM), shown as bar plots from three independent replicates, after subtraction of the signals obtained from untreated samples and samples treated with Dynasore alone.

HeLa cells were incubated with dye-peptide conjugates (4 μM) for 3 h at 37 °C in their medium. Under these conditions, enhanced intracellular fluorescence was observed for the cyclic peptide conjugate relative to the linear analogue, as detected in the dye channel, with SiR–Hoechst used as a nuclear counterstain (Figure 5b). Additionally, for quantitative analysis, flow cytometry was employed. Increasing concentrations of dye-peptide conjugates led to a higher median fluorescence intensity (MFI), with a more pronounced MFI observed for the cyclic peptide relative to its linear counterpart (Figure 5c).

Cell viability was assessed by staining with 4′,6-diamidin-2-phenylindol (DAPI) during flow cytometry analysis. The stability of the formed cycloadducts, **Rh518bisCTet**-Pep1 and **Rh518bisCTet-SingleTet**-Pep9, under the conditions used for microscopy/flow cytometry, as well as their fluorescence response, were evaluated in advance to ensure that no degradation occurred under the experimental conditions (Figure S26). Given the punctate fluorescence patterns observed in the microscopy images (Figure 5b), suggestive of endosomal uptake, experiments were also performed in the presence of Dynasore, a known inhibitor of dynamin-dependent endocytic pathways.^71^ For Rh518–Pep1, treatment with Dynasore resulted in an approximately 2-fold reduction in MFI, indicating a significant contribution of endocytic uptake mechanisms (Figure 5d). In contrast, the control probe, corresponding to the dye fully reacted with BCN and therefore expected to cross the membrane predominantly via passive diffusion, showed no significant change in fluorescence intensity upon Dynasore treatment. HeLa cells maintained high viability following 4 h of incubation under the conditions tested (Figure S28a). Comparative uptake studies among Pep1-Pep4 dye conjugates revealed broadly similar cellular internalization profiles, except for the Pep4 conjugate, which exhibited reduced uptake (Figure S28b). This behavior is likely attributed to its larger size and higher net negative charge. Despite the requirement of using a dienophile-functionalized peptide,^72^ collectively, these findings establish bis-functional fluorogenic dyes as effective tools for generating and monitoring cyclic peptides in living cells, thereby providing a foundation for their broader use in live-cell labeling strategies and other applications.

### Fluorescence Anisotropy with Bis-Functional Dye

One of the most compelling potential applications of bis-functional dyes is their use in fluorescence anisotropy measurements. Fluorescence anisotropy directly correlates with the rotational mobility of a fluorophore when comparing polarized emission intensities parallel and perpendicular to the polarized excitation wavelength.^73,74^ When a fluorophore is freely rotating, rapid depolarization results in low anisotropy values, whereas restricted rotational motion, such as when the fluorophore is rigidly associated with a protein, leads to higher anisotropy. E.g., in fluorescent proteins such as GFP and YFP, their chromophore is rigidly embedded within the protein scaffold, yielding a steady-state anisotropy of *r* ≈ = 0.3.^73^ In contrast, small-molecule dyes freely diffusing in solution typically display very low anisotropy values (*r* ≈ 0.01–0.05). Consequently, studies of protein–protein interactions and macromolecular assemblies often rely on fusion of fluorescent proteins to the protein of interest. However, fluorescent protein fusion strategies introduce intrinsic limitations. Fluorescent proteins are comparable in size to, or larger than, many target proteins, which can perturb native structure, mask subtle conformational changes, or restrict labeling to the N- or C-termini. Small-molecule dyes have also been used for anisotropy measurements, but even when covalently attached to a protein, residual rotational freedom of the fluorophore can dominate the anisotropy signal, obscuring underlying protein dynamics (Figure 6a).

**Figure 6:**
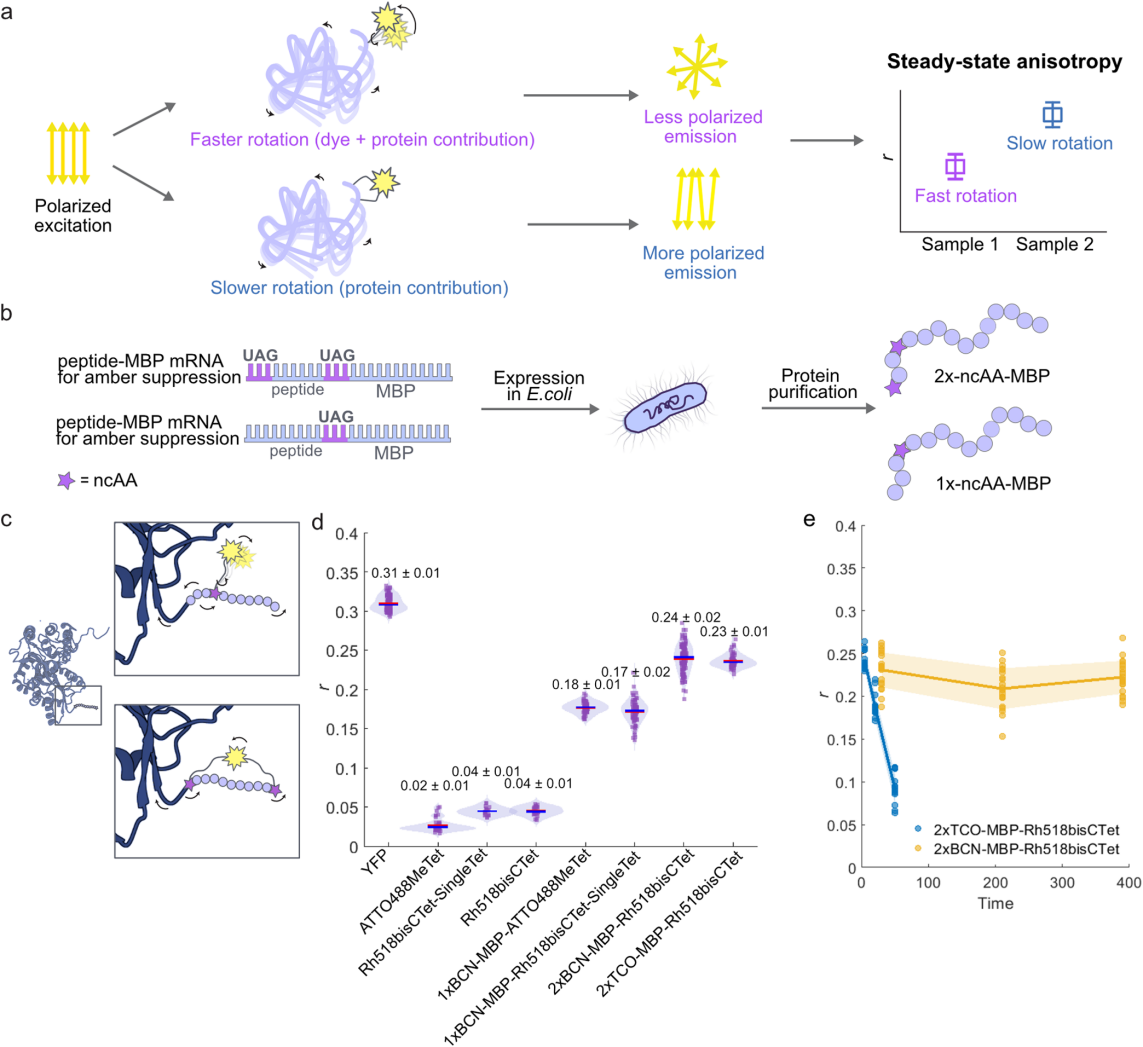
Fluorescence anisotropy measurements *in vitro.* (a) Schematic illustrations of the contribution of fluorophore rotational freedom to the measured protein rotation in fluorescence anisotropy as single-site or double-site-labeled. (b) Experimental design for site-specific double incorporation of ncAAs via two amber codons in the model protein MBP. (c) Visualization of MBP labeled with the fluorogenic dye through either single or double site-specific conjugation. (d) Anisotropy steady state values (calculated from at least three replicates) for the reference (YPF), the free-dyes, previously reacted with BCN–OH (**ATTO488MeTet, Rh518bisCTet-SingleTet** and **Rh518bisCTet**), single-labeled protein (1xBCN-MBP-**ATTO488MeTet** and 1xBCN-MBP-**Rh518bisCTet-SingleTet**) and double-labeled protein (2xBCN-MBP-**Rh518bisCTet** and 2xTCO-MBP-**Rh518bisCTet**). Median values are reported as red line, while mean values are reported as blue line. (e) Time-dependent steady-state fluorescence anisotropy measurements of TCO- and BCN-labeled proteins were performed to assess measurement stability over time. The results show that the BCN derivative exhibits consistently stable anisotropy values throughout the observation period, whereas the TCO-labeled proteins display greater temporal variability.

For example, recent work has demonstrated that rigid, bifunctional labeling can substantially reduce this limitation.^75^ By conjugating a single, rigidly oriented rhodamine dye to a specific α-helix in the RCK domain of the MthK potassium channel, it was possible to resolve Ångström-scale conformational changes by mapping small helix rotations onto changes in fluorophore dipole orientation. In this system, the achieved spatial resolution (∼3.4–8.1 Å) arose from precise coupling between protein motion and dye orientation, enabled by rigid bifunctional attachment.^76^ Despite these impressive results, this approach is currently largely limited to in vitro experiments, as it relies on cysteine-targeting bifunctional rhodamines and requires protein purification, immobilization, and extensive removal of unreacted dye to minimize background fluorescence arising from fluorescent unreacted dye.

The key features of an ideal small-molecule fluorescence anisotropy probe include several interconnected photophysical and biochemical properties,^77^ which adds to the key properties required for live cell imaging, such as cell permeability, photostability and excitation and emission in the visible range. First, the probe should exhibit high brightness in order to enable measurements at low protein concentrations and maximize signal sensitivity. Second, it should be attached to the protein in a manner that efficiently restricts local rotational freedom without distorting the fluorophore itself, as excessive twisting or conformational strain can reduce fluorescence quantum yield^60^ and compromise anisotropy performance. Third, the fluorophore should possess a fluorescence lifetime that is appropriately matched to the rotational correlation time of the biomolecular system under investigation. Finally, the probe should ideally remain quenched or weakly emissive in its unreacted state, thereby minimizing background fluorescence from free dye. Such fluorogenic behavior eliminates or reduces the need for extensive purification after labeling and prevents free fluorophore from lowering the measured anisotropy signal. This feature is particularly important for applications in complex biological environments and live-cell settings.

In principle, the BisCTet dyes reported in this study possess the key features required for accurate fluorescence anisotropy measurements. Building on this rationale, we employed our bis-labeling technology to perform proof-of-concept fluorescence anisotropy measurements *in vitro* and evaluate the bis-functional dye **Rh518bisCTet** to improve rotational restriction upon protein conjugation (Figure 6b).

Maltose-binding protein (MBP), as the model target protein, was expressed in *E. coli* with two amber codons, positioned at an amino acid distance defined by Pep1, to enable site-specific incorporation of two *trans*-cyclooct-2-en *L*-lysine, TCO*AK or two BCNK units. MBP was also expressed with only a single amber codon to test monofunctional labeling. Following purification, click labeling with **Rh518bisCTet** was confirmed by SDS-PAGE analysis (Figure S30).

Steady-state fluorescence anisotropy measurements were then performed in a cuvette. As an additional control, a commercially available monofunctional dye ATTO488-tetrazine (ATTO488-MeTet), which exhibits fluorescence lifetimes of 4.0 ns in aqueous solution,^78^ was included as a reference dye. These values are close to those of our test dye (τ = 3.7 ns for **Rh518bisCTet**; see Table 1). As expected, free dyes exhibited low anisotropy values (*r* ≈ 0.02–0.04), consistent with rapid rotational diffusion (Figure 6c,d). Upon conjugation to MBP, monofunctional dyes showed an increase in anisotropy (*r* ≈ 0.17–0.18 for the BCN-MBP). Strikingly, bis-functional labeling of control peptide (see Figure S31) and proteins resulted in a pronounced increase, yielding anisotropy values of *r* ≈ 0.23 for TCO-MBP and 0.24 for BCN-MBP. These results indicate that bis-functional attachment effectively restricts fluorophore rotation and improves coupling between fluorophore orientation and protein motion. The lower anisotropy values observed for the single-site attached dyes likely arise from residual rotational freedom of the fluorophore around the attachment linker. Notably, click labeling of TCO-functionalized MBP and the subsequent anisotropy measurements had to be performed rapidly, as anisotropy values for all samples decreased significantly within 1 h, indicating progressive detachment of the dye from the protein (Figure 6e).^79^ This behavior was observed for all synthesized dyes (see Figures S13 and S14 for fluorogenic response) as well as for ATTO488-MeTet. In contrast, BCN-functionalized MBP exhibited stable anisotropy values throughout the 6 h monitoring period, highlighting the superior stability of this conjugation strategy (Figure 6e). Taken together, these findings demonstrate that bis-functional dyes can substantially reduce the intrinsic rotational freedom associated with monofunctional dyes and improve steady-state anisotropy readouts of proteins.

This approach provides a practical and modular alternative to fluorescent protein fusions and establishes a foundation for future studies of protein conformational dynamics, including disordered regions and transient states in live cells. Beyond anisotropy measurements, the biorthogonal and bifunctional nature of these dyes also opens opportunities for proximity-driven cross-linking^80,81^ and controlled release strategies^82,83^ in chemical biology and drug development, owing to the click-to-release behavior of this class of dyes upon reaction with TCO, combined with the high stability of the corresponding conjugates formed with BCN (Figures S32–S33).

### Live-Cell Labeling with (Si)­Rh-CTet Dyes

Given the favorable performance of bis-functional dyes in cyclic peptide studies, including efficient cyclization, we next assessed their suitability for live-cell protein labeling. In live-cell and in vivo settings, unreacted excess fluorophore is particularly difficult to remove and can lead to elevated background signal and imaging artifacts.^32^ Fluorogenic probes offer a significant improvement of signal-to-noise ratio in live cell imaging. This phenomenon has been demonstrated in numerous live-cell imaging studies, particularly those employing GCE labeling approach.^84–86^ Nevertheless, misincorporated and nonincorporated ncAA pose a significant challenge,^87^ as they are difficult to remove and may react with fluorogenic probes, thereby generating undesired fluorescence and increasing nonspecific background signal. Bistetrazine fluorogenic probes offer a promising solution to this limitation and can be used in living cells with high target selectivity and minimal nonspecific background fluorescence. Our^88–90^ and other research groups^91–94^ have established methodologies for incorporating two ncAAs into a single target protein, thereby laying the foundation for the successful use of the bistetrazine fluorogenic probes described herein. This dual-incorporation strategy is expected to further reduce background fluorescence and enhance imaging contrast in live-cell microscopy. We first assessed the fluorogenic performance of **(Si)­Rh-monoCTet** dyes in live-cell labeling using GCE, which enables site-specific incorporation of ncAAs with single-amino acid precision via reassignment of a nonsense codon, in our case the amber (TAG) codon. To verify ncAA incorporation and to confirm selective localization of the dye at the protein of interest, HEK293T cells were transiently transfected with a vimentin construct containing an amber stop codon at position 116. Vimentin is a filamentous cytoplasmatic protein, which in our case, was fused with mCerulean (mCer) at the C-terminus. mCer, is a cyan-fluorescent protein chosen to avoid spectral overlap with the rhodamine-based dyes and used, in our case, as a benchmark for dye labeling via colocalization analysis (Figure S34a). BCNK was selected as the ncAA for these experiments, as the engineered tRNA/PylRS^AF^ pair enables its incorporation at amber codons.^95,96^ After 24 h of expression, cells were washed to remove excess BCNK and subsequently incubated with 2 μM of the monofunctional dye for 30 min, using either green- or red-emitting derivatives. Efficient labeling and colocalization with vimentin were observed for both dyes tested (Figure S34). As a negative control, vimentin was expressed in the presence of *N*-Boc protected lysine, BocK, instead of BCNK. Since BocK lacks a reactive dienophile, any observed fluorescence would indicate nonspecific labeling. No significant fluorescence was detected under these conditions for all dyes, with lower background fluorescence in the case of **SiRh628monoCTet**, confirming the specificity of the labeling reaction and the fluorogenicity of these dyes (Figure S34d,e). A comparison with the widely used commercial red fluorophore SiR-Tet was also performed,^97^ highlighting the improved background suppression achieved with our design (see SI, Figure S35).

Despite many recent advances of GCE in mammalian cells,^88,98–101^ it still has some limitations that hinder its full potential. In particular, fluorescence background may arise from misincorporation of the ncAA at native amber stop codons. Although amber codons occur less frequently than other stop codons, they still represent a measurable fraction of total codons, which can lead to off-target incorporation events. While orthogonal organelle design can introduce mRNA specificity, it cannot completely eliminate the problem.^88,99^ Additionally, nonincorporated ncAAs may stick to the cellular environment or bound to the suppressor tRNA or the synthetase. Bistetrazine fluorogenic probes offer a promising strategy to mitigate these issues. Because bistetrazine dyes are designed to remain nonfluorescent in their monoclicked state (see Figure 2e), fluorescence activation should occur predominantly after completion of the dual biorthogonal reaction. As a result, we expect these probes to substantially reduce background signals arising from misincorporated ncAAs or from residual, nonincorporated ncAAs, thereby improving the signal-to-noise ratio and enhancing the overall performance of GCE-based labeling approaches. Thus, to evaluate bis-functional dyes in live cells, a double-amber strategy was employed (Figure 7a). Two amber codons were introduced at defined positions, using the above tested Pep1-derived sequences (see Table 2) as a suitable linker for bis-conjugation *in vivo* fused to the C-terminus of vimentin. Cells were transfected following the same protocol as for the single-amber constructs. In this case, efficient labeling and colocalization were again observed (Figure 7b). To further quantify the fluorescence enhancement, we performed flow cytometry analysis (Figures S40–S42), expressing singleTAG-Vimentin-iRFP and doubleTAG-Vimentin-iRFP constructs in HEK293T cells.

**Figure 7:**
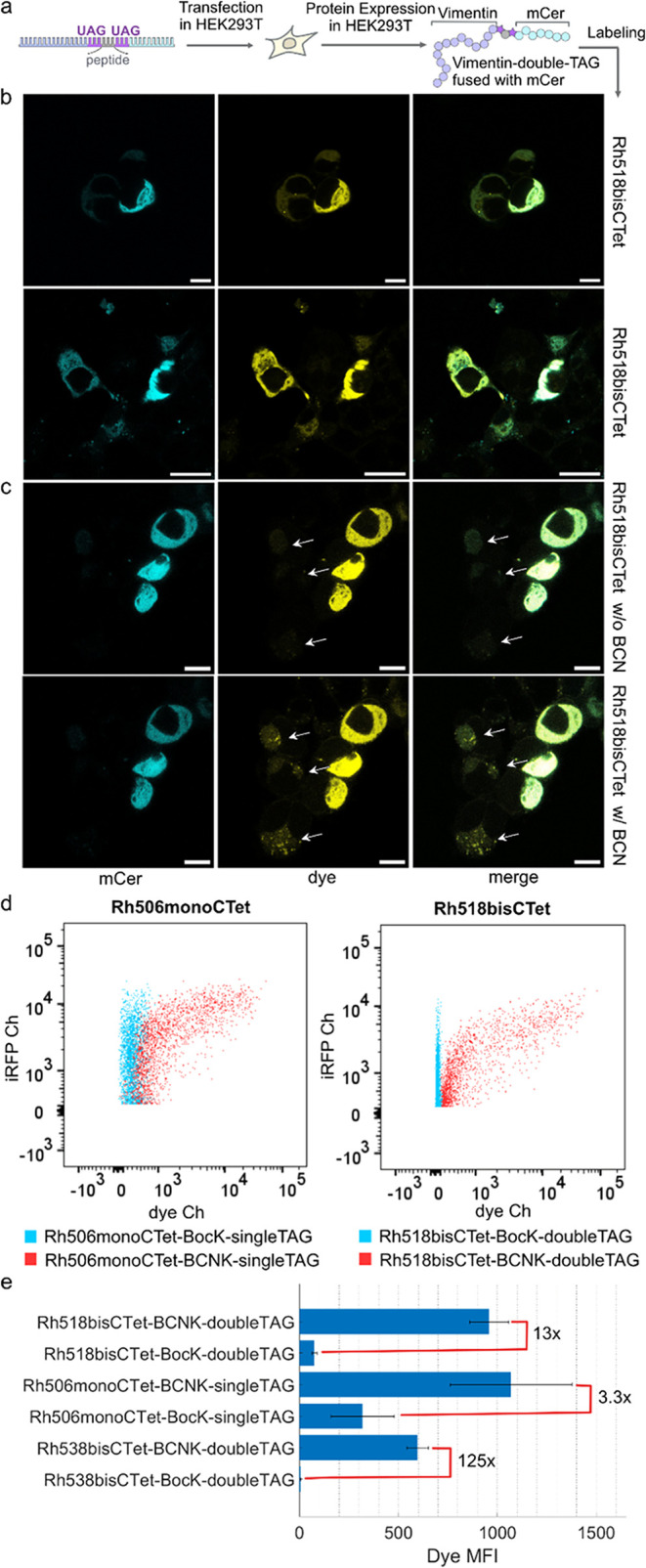
Live-cell labeling of HEK293T cells transiently expressing the protein of interest. (a) Double-amber GCE for site-specific incorporation of two ncAAs into the Vim–mCer construct, followed by (b) dual-tagging labeling with **Rh518bisCTet** and (c) dual-tagging labeling with **Rh518bisCTet**, and BCN–OH treatment. Same well images were acquired before and after addition of BCN–OH. Media was replaced with fresh FluoroBrite DMEM containing BCN–OH and images were acquired after 20 min at RT. Exemplary white arrows highlight representative regions with increased background signal. Brightness/contrast in (c) has been adjusted consistently for better visualization between ± BCN–OH. Scale bars: 10 μm. Colors have been adapted. (d) Representative flow cytometry dot plot showing dye fluorescence plotted against iRFP fluorescence for individual cells after gating on iRFP positive cells (see Figure S40). Each event corresponds to a single cell, enabling assessment of dye-positive (488 nm channel), iRFP-positive (630 nm channel) and double-positive populations among Bock (light blue) and BCNK (red) conditions for **Rh506monoCTet** and **Rh518bisCTet**. (e) Bar plot shows the MFI of the dye channel in iRFP positive cells for each condition, together with the reported MFI ratio between the corresponding BocK and BCNK samples. Because the dyes display different excitation efficiencies depending on the laser used, MFI values were corrected according to the relative excitation at the selected laser wavelength. Specifically, excitation was normalized to the excitation maximum, with relative excitation values of 0.58 for **Rh506monoCTet**, 0.40 for **Rh518bisCTet** for the green laser, and 0.33 for **Rh538bisCTet** for the yellow laser. For **Rh538bisCTet**, an additional correction was applied because the detector voltage was set to 200 instead of 250; this correction was used only to improve visualization and comparison across samples. All corrections were applied consistently across BocK and BCNK replicates.

In this case, iRFP (infrared fluorescent protein) was chosen to avoid spectral overlapping with the dyes available under the flow cytometer settings. Compared to BocK controls, a pronounced fluorescence enhancement was observed for the bifunctional dyes, particularly **Rh538bisCTet** with a 125-fold MFI enhancement (Figure 7d). For **Rh518bisCTet** a residual fluorescence signal was nevertheless observed in the BocK samples, which reduced its fluorescence turn-on. Based on our stability studies (Figure S12), this background signal is likely attributable to partial formation of **Rh506monoCTet** during the course of the experiment. Notably, under the laser settings used for flow cytometry, this degradation product is excited more efficiently than the parent bifunctional dye, which can lead to an apparent increase in background fluorescence despite the overall specificity observed for the BCNK/doubleTAG condition. The reduced background observed for the bis-functional dyes (Figures 7d,e and S36) could be asserted to their higher fluorogenicity compared to the monofunctional ones. Moreover, the monoclicked intermediate of **Rh518bisCTet** (**Rh518bisCTet-SingleTet**, Figure 2) showed only weak turn-on fluorescence intensity. This is due to the fact that the free dye has to undergo two consecutive reactions to reach its highest fluorogenicity. However, in the case of double-site-tagged proteins, induced proximity via the first click reaction makes the second reaction more likely and thus, faster, as demonstrated previously (three-component reactions instead of two-component reaction, see Figure 4).

This induced proximity effect also mitigates potential artifacts arising from premature translational termination at the first amber codon, a well-known limitation of GCE, since codon suppression inherently competes with the endogenous translation termination machinery whenever a stop codon is encountered.^102^ Specifically, dyes reacting with truncated proteins containing only a single reactive handle are expected to remain largely nonfluorescent, as the likelihood of incorporating a second ncAA at a suitable distance to enable intramolecular activation is low. To experimentally test this concept, cells were labeled with the dye and washed only once to remove excess fluorophore. After imaging, BCN–OH was added to the medium and images were acquired again (Figures 7c and S38). Within 20 min, increased fluorescence was observed at cellular regions not colocalizing with mCer, consistent with activation of previously quenched dye molecules accumulated or bound at nonspecific sites. To further demonstrate that labeling occurs specifically at the intended target site, where the two ncAAs are positioned at the defined distance required by our linker, we coexpressed singleTAG-Vim fused to iRFP together with doubleTAG-Vim fused to mCer (Figure 8a–b). When labeling was performed using the monofunctional dye **Rh506monoCTet**, stochastic labeling of both constructs was observed, as expected for single-site labeling approaches (Figure 8a; see also Figure S39a for labeling with **Rh528monoCTet**).

**Figure 8:**
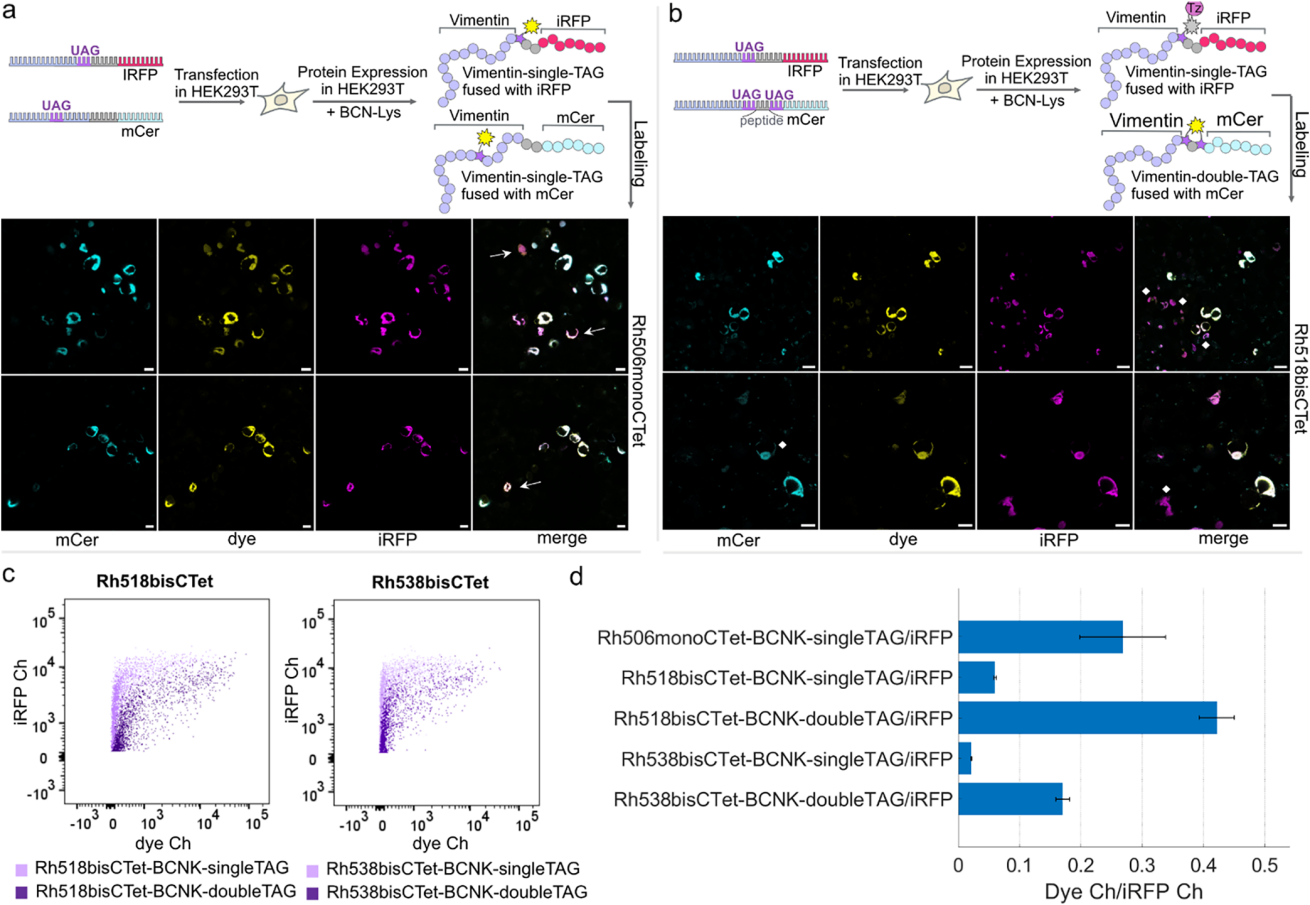
Live-cell labeling of HEK293T cells transiently expressing the proteins of interest. HEK293T cells were cotransfected with either (a) two single-amber constructs (singleTAG-Vim-mCer and single-TAG-Vim-iRFP) and labeled with the monofunctional dye **Rh506monoCTet**, or (b) a combination of single- and double-amber constructs (singleTAG-Vim-iRFP and double-TAG-Vim-mCer) and labeled with the bis-functional dye **Rh518bisCTet**. White arrows in (a) highlight stochastic labeling arising from nonselective labeling of both singleTAG proteins by the monofunctional dye. In contrast, diamonds in (b) indicate the absence/reduce labeling on the singleTAG protein when using the bis-functional dye. Consequently, a higher degree of colocalization between the dye and mCer channels is observed specifically for the doubleTAG protein. Brightness and contrast have been consistently adjusted. Scale bar 10 μm. (c) Representative flow cytometry dot plot showing dye fluorescence plotted against iRFP fluorescence for individual cells. Each event corresponds to a single cell, enabling assessment of dye-positive, iRFP-positive and double-positive populations. (d) Flow cytometry data shown as the ratio of MFI in the dye channel (488 nm excitation for **Rh518bisCTet** or 561 nm excitation for **Rh538bisCTet**) to the iRFP channel (640 nm) in iRFP-positive cells. These results demonstrate the stronger fluorescence turn-on of the bis-functional dye in the double-amber construct, whereas the same dye remains comparatively dim in the single-amber construct. Normalization to the iRFP channel was necessary to account for the higher expression levels observed in the single-amber construct relative to the double-amber construct.

In contrast, upon incubation with the bifunctional dye **Rh518bisCTet**, efficient labeling was observed preferentially for the doubleTAG construct containing the two correctly positioned ncAAs. Importantly, although single-site GCE is intrinsically more efficient than double-site GCE, colocalized fluorescence was detected for the doubleTAG protein (mCerulean channel) (Figure 8b; see also Figure S39b for labeling with **Rh538bisCTet**). These results strongly support the specificity of the bifunctional labeling strategy, suggesting that isolated ncAA misincorporation events, represented here by the singleTAG-protein, remain largely nonfluorescent, whereas productive fluorescence activation requires the simultaneous incorporation of two ncAAs in close spatial proximity. The mixed-plasmid coexpression experiment, in which plasmid concentrations were kept low to generate a heterogeneous cell population with differential iRFP and mCerulean expression levels, provides strong evidence that fluorescence enhancement is highly dependent on the intended dual-labeling geometry rather than nonspecific incorporation events. To further quantify the fluorescence enhancement, we also performed flow cytometry analysis in this case by independently expressing single-amber-Vim-iRFP and double-amber-Vim-iRFP in HEK293T cells, followed by labeling with **Rh518bisCTet** and **Rh538bisCTet** (Figures 8c and S40–S42). Fluorescence intensity was normalized to expression levels by calculating the ratio between the MFI of the dye channel and the MFI of the iRFP channel (Figure 8d). Compared to single-amber protein expression, a pronounced fluorescence enhancement was observed for the bifunctional dyes (Figure 8c,d). For **Rh518bisCTet** and **Rh538bisCTet**, a significant fluorescence response was detected specifically in the doubleTAG construct in the presence of BCNK. These results confirm that monoclicked bis-functional dyes remain effectively dark in live cells with negligible effect on fluorescence intensity.

By demonstrating their suitability for in vivo applications and defining the key parameters required for efficient live-cell labeling, this work substantially broadens the applicability of bis-functional dyes in complex biological environments. These probes open new opportunities for real-time monitoring of protein dynamics and conformational changes directly in living cells. Combined with fluorescence anisotropy measurements, they may enable the investigation of rotational mobility, interaction kinetics, and transient protein–protein interactions with high temporal resolution in real-time. Such approaches could facilitate the identification of previously unknown interaction partners and provide deeper insight into dynamic cellular processes under physiologically relevant conditions.

## Conclusions

In summary, we have developed a fluorogenic dye platform that addresses several long-standing limitations in protein labeling strategies. An unprecedented tetrazine functionalization at the amino groups of (silicon)-rhodamine scaffolds yields an exceptionally short unconjugated linker and enables highly efficient quenching, providing access to both mono- and bis-functional fluorogenic dyes within a unified chemical framework. The monofunctional red-emitting derivative enhances conventional click labeling through its high fluorogenic response, while the bis-functional dyes represent an early example of this class to combine live-cell compatibility with fluorescence turn-on ratios approaching 10^3^ upon biorthogonal reaction. Beyond single-site applications, the bis-functional architecture enables capabilities that are difficult to achieve with conventional fluorescent proteins, self-labeling tags, or monofunctional dyes, including peptide macrocyclization to improve cellular uptake, in vivo protein clicking to further improve selectivity, and fluorescence anisotropy measurements that more accurately report on protein rotational dynamics. Together, these studies reveal an intrinsic limitation of traditional monofunctional dye-labeling approaches, in which residual dye rotation can obscure subtle yet biologically relevant protein motions, and demonstrate the advantages of bifunctional fluorogenic probes for real-time investigations in living systems. Although this class of dyes currently shows somewhat reduced stability during prolonged incubation in cellular media, no limitations were observed under the experimental conditions used in this study. Moreover, the substantially enhanced stability of the corresponding BCN cycloadducts highlights promising opportunities for further optimization. For example, replacing strongly electron-withdrawing tetrazine substituents with less electron-deficient motifs, such as triazines, or introducing electron-donating and/or sterically bulky substituents onto the tetrazine scaffold, could further improve stability during extended live-cell experiments, albeit potentially at the expense of reduced iEDDA reaction kinetics. Nevertheless, such modifications may still preserve favorable fluorogenic properties while providing a more balanced trade-off between reactivity and stability.

In addition, the modular synthetic strategy of this scaffold, based on a late-stage functionalization with tetrazines, provides broad opportunities for photophysical tuning. Red-shifted derivatives suitable for far-red and near-infrared imaging could be achieved through heteroatom substitution within the xanthene core,^103^ for example by incorporation of germanium, phosphorus^104^ and selenium or by extending the conjugation system in silicon-rhodamine.^105^ Furthermore, replacement of the spirolactone moiety with alternative ring systems known to enhance Stokes shifts may improve spectral separation and reduce background interference in cellular imaging applications.^106,107^ Taken together, this work establishes a versatile new class of fluorogenic dyes that integrates chemical properties with biological functionality, while also defining key design criteria for the application of small fluorophores in fluorescence anisotropy measurements *in cellulo*. We anticipate that this platform will find broad utility in chemical biology, live-cell imaging, and studies of protein structure and dynamics, and will provide a foundation for future developments in multivalent biorthogonal probes and super-resolution microscopy.

## Supplementary Material



## Notes

Syntheses, compound characterization data, and details of anisotropy studies as well as imaging experiments (PDF)

## Abbreviations

BCN: (bicyclo­[6.1.0]­non-4-yn-9-yl)­methanol
BCNK: BCN–lysine
DMEM: Dulbecco's modified eagle medium
FPs: fluorescent proteins
GCE: genetic code expansion
GFP: green fluorescent protein
iEDDA: inverse electron-demand Diels–Alder reaction
iRFP: infrared fluorescent protein
ncAAs: noncanonical amino acids
MBP: maltose-binding protein
MFI: median fluorescence intensity
PBS: phosphate buffered saline
PET: photoinduced electron transfer
SPAAC: strain-promoted azide–alkyne cycloaddition
TCO: *trans*-cyclooctene
YFP: yellow fluorescent protein
