# Supplementary material for: Bis-Tetrazine Fluorogenic (Silicon)-Rhodamine Dyes for Live-Cell Labeling
Source: J Am Chem Soc. 2026 Jul 11;148(29):30887–904. doi: 10.1021/jacs.6c04723 (PMC13426259; doi:10.1021/jacs.6c04723)

Supporting Information for

# Bis-Tetrazine Fluorogenic (Silicon)-Rhodamine Dyes for Live-Cell Labeling

Sabrina Giofrè[1,2], Lukas Schartel[1,3], Richard Wombacher[4], Edward A. Lemke[1,5]*

Corresponding author: edlemke@uni-mainz.de

[1] Biocenter, Johannes Gutenberg University Mainz, 55128 Mainz, Germany

[2] Institute of Molecular Biology postdoctoral program, 55128 Mainz, Germany

[3] Departments of Biology and Chemistry, IMPRS on Cellular Biophysics, 55128 Mainz, Germany

[4] Department of Chemical Biology, Max Planck Institute for Medical Research, 69120 Heidelberg, Germany

[5] Institute of Molecular Biology (IMB gGmbH), 55128 Mainz, Germany

## Table of Contents


Abbreviations 4

General Information 5

Extinction Coefficients and Fluorescence Quantum Yields 6

Fluorescence Lifetime Measurements 6

Cloning 7

HEK293T culture, transfection and labeling 8

Live-cell imaging 9

HeLa culture and linear and cyclic peptide treatment 9

Flow Cytometry (FC) of HeLa cells 9

Flow cytometry (FC) of HEK293T cells 10

MBP protein expression and purification 11

Anisotropy Measurement 12

Click reaction between synthetized dyes and BCN-OH 13

Intramolecular click reaction between Rh518bisCTet and bis-cyclooctynyl peptides, Pep1-8 and cyclooctynyl peptide, Pep9. 27

Kinetics of click reaction 45

Stability monitoring of the peptide-dye conjugate for FC 48

Flow Cytometry data for HeLa cells 49

Protein labeling in vitro 51

Anisotropy data 53

Click-to-release: doxorubicin as a drug model 54

Live cell data 56

Flow Cytometry data for HEK293T cells 62

Summary of synthetic steps 67

Synthesis of 3-ethyl-6-(methoxymethyl)-1,2,4,5-tetrazine (compound 1a) 68

Synthesis of (6-ethyl-1,2,4,5-tetrazin-3-yl)methanol (compound 2a) 68

Synthesis of 3-(bromomethyl)-6-ethyl-1,2,4,5-tetrazine (compound 3a) 69

Synthesis of 4-hydroxybutanamide (SI1) 69

Synthesis of 4-((tert-butyldimethylsilyl)oxy)butanamide (SI2) 70

Synthesis of 4-((*tert*-butyldimethylsilyl)oxy)butanenitrile (SI3) 70

Synthesis of 4-hydroxybutanenitrile (SI4) 71

Synthesis of 3-(6-methyl-1,2,4,5-tetrazin-3-yl)propan-1-ol (compound 2b) 71

Synthesis of 3-(3-bromopropyl)-6-methyl-1,2,4,5-tetrazine (compound 3b) 72

Synthesis of 3-(methoxymethyl)-6-phenyl-1,2,4,5-tetrazine (compound 1c) 72

Synthesis of (6-phenyl-1,2,4,5-tetrazin-3-yl)methanol (compound 2c) 73

Synthesis of 3-(bromomethyl)-6-phenyl-1,2,4,5-tetrazine (compound 3c) 73

Synthesis of 3-(methylamino)phenol (compound 4) 74

Synthesis of 2-(6-(methylamino)-3-(methyliminio)-3H-xanthen-9-yl)benzoate (compound 5a) 75

Synthesis of 2-(6-amino-3-iminio-3H-xanthen-9-yl)benzoate (compound 5b) 75

Synthesis of *tert*-butyl 3-bromobenzoate (compound 6) 76

Synthesis of 3-bromo-*N*-methylaniline 76

Synthesis of *N*-allyl-3-bromo-*N*-methylaniline (compound 7) 77

Synthesis of 4,4'-methylenebis(*N*-allyl-3-bromo-*N*-methylaniline) (compound 8) 77

Synthesis of 3,7-bis(allyl(methyl)amino)-5,5-dimethyldibenzo[*b,e*]silin-10(5H)-one (compound 9) 77

Synthesis of 3,7-bis(allyl(methyl)amino)-5,5-dimethyl-3'H,5H-spiro[dibenzo[*b,e*]siline-10,1'-isobenzofuran]-3'-one (compound 10) 78

Synthesis of 5,5-dimethyl-3,7-bis(methylamino)-3'H,5H-spiro[dibenzo[*b,e*]siline-10,1'-isobenzofuran]-3'-one (compound 11) 79

Synthesis of 3'-amino-6'-(((6-ethyl-1,2,4,5-tetrazin-3-yl)methyl)amino)-3H-spiro[isobenzofuran-1,9'-xanthen]-3-one (Rh506monoCTet) 80

Synthesis of 3',6'-bis(((6-ethyl-1,2,4,5-tetrazin-3-yl)methyl)amino)-3H-spiro[isobenzofuran-1,9'-xanthen]-3-one (Rh518bisCTet) 81

Synthesis of 3'-(((6-ethyl-1,2,4,5-tetrazin-3-yl)methyl)(methyl)amino)-6'-(methylamino)-3H-spiro[isobenzofuran-1,9'-xanthen]-3-one (Rh528monoCTet) 82

Synthesis of 3',6'-bis(((6-ethyl-1,2,4,5-tetrazin-3-yl)methyl)(methyl)amino)-3H-spiro[isobenzofuran-1,9'-xanthen]-3-one (Rh538bisCTet) 83

Synthesis of 3-(((6-ethyl-1,2,4,5-tetrazin-3-yl)methyl)(methyl)amino)-5,5-dimethyl-7-(methylamino)-3'H,5H-spiro[dibenzo[b,e]siline-10,1'-isobenzofuran]-3'-one (SiRh628monoCTet) 84

Synthesis of 3,7-bis(allylamino)-5,5-dimethyl-3'H,5H-spiro[dibenzo[*b,e*]siline-10,1'-isobenzofuran]-3'-one (SiRh640bisCTet) 85

Synthesis of 3-(dimethylamino)-5,5-dimethyl-7-(methyl(3-(6-methyl-1,2,4,5-tetrazin-3-yl)propyl)amino)-3'H,5H-spiro[dibenzo[*b,e*]siline-10,1'-isobenzofuran]-3'-one (SiRh634monoC3Tet) 86

Synthesis of (*S,E*)-cyclooct-2-en-1-yl ((1*S*,2*R*,3*R*,5*R*)-2-hydroxy-3-methyl-5-(((2*R*,4*S*)-2,5,12-trihydroxy-2-(2-hydroxyacetyl)-7-methoxy-6,11-dioxo-3,4,6,11-tetrahydro-2H-naphtho[2,3-g]chromen-4-yl)oxy)cyclohexyl)carbamate (compound 12) 87

Synthesis of the Rh518bisCTet-Pep1 adduct 88

X-Ray Crystallography 89

References 92

HRMS Spectra 94

NMR Spectra 99

## Abbreviations

| | |
|---|---|
| AUC | Area Under Curve |
| BCN-OH | Bicyclo[6.1.0]non-4-yne alcohol |
| BCNK | Bicyclononyne Lysine |
| BSA | Bovine Serum Albumin |
| CMV | Cytomegalovirus |
| DAPI | 4′,6-Diamidin-2-phenylindol |
| DCM | Dichloromethane |
| DIPEA | Diisopropyl ethyl amine |
| DMAP | Dimethyl amino pyridine |
| DMEM | Dulbecco's Modified Eagle's Medium |
| DMF | Dimethylformamide |
| DMSO | Dimethyl Sulfoxide |
| Doxo | Doxorubicin |
| EDTA | Ethylenediaminetetraacetic acid |
| ESI | Electrospray Ionization |
| FC | Flow Cytometry |
| HEPES | 4-(2-Hydroxyethyl)-1-piperazineethanesulfonic Acid |
| HPLC | High Performance Liquid Chromatography |
| HRMS | High Resolution Mass Spectrometry |
| IRF | Instrument Response Function |
| iRFP | infra-Red Fluorescent Protein |
| LCMS | Liquid Chromatography Mass Spectrometry |
| MBP | Maltose Binding Protein |
| MES | 2-(*N*-Morpholino)ethanesulfonic Acid |
| MOPS | 3-(*N*-Morpholino)propanesulfonic Acid |
| NES | Nuclear Export Signal |
| NHS | *N*-Hydroxysuccinimide |
| NMR | Nuclear Magnetic Resonance |
| PBS | Phosphate Buffered Saline |
| PCR | Polymerase Chain Reaction |
| PEI | Polyethylenimine |
| PMSF | Phenylmethylsulfonyl Fluoride |
| rcf | relative centrifugal force |
| RT | Room Temperature |
| SCOK | Strained Cyclooctyne Lysine |
| SEC | Size Exclusion Chromatography |
| TBAF | Tetrabutylammonium Fluoride |
| TBDMSiCl | *Tert*-Butyl Dimethyl Silyl Chloride |
| TCEP | Tris(2-carboxyethyl)phosphine |
| TCOA*K | *trans*-Cyclooctene axial Lysine |
| TFAA | Trifluoroacetic anhydride |
| THF | Tetrahydrofuran |
| TLC | Thin Layer Chromatography |
| TMS | Tetramethylsilane |
| YFP | Yellow Fluorescent Protein |

## General Information

Reaction temperatures were measured in degree Celsius (°C). Reaction times were recorded in minutes (min), hours (h) or days (d). All yields are isolated yields, unless noted differently. Room temperature (RT) refers to an ambient temperature of approximately 22 °C.

**Purchased Chemicals**

Unless noted otherwise, all commercially available chemicals were purchased from common suppliers, Sigma-Aldrich and abcr, and used without further purification. Solvents were bought in p.a. quality and used without further purification. Dry solvents were bought in extra dry quality from Sigma-Aldrich (stored in sealed brown-glass bottles and kept under argon atmosphere). Peptides were synthetized from piChem, Austria, by requiring a customized synthesis.

**Thin Layer Chromatography (TLC)**

For analytical TLC, silica gel 60 coated (0.25 mm) F254 aluminum foils from Supelco - Sigma-Aldrich were used. The TLC plates were visualized under UV fluorescence (λ = 254 nm, 365 nm), and/or by staining with $KMnO_4$ and Molybd staining solution followed by heating.

$KMnO_4$ staining: $KMnO_4$ (1.5 g), $K_2CO_3$ (10 g), aq NaOH solution (10 wt %, 1.25 mL) in H2O (200 mL). Molybd staining: phosphomolybdic acid (20 g) in EtOH (100 mL).

**Flash Column Chromatography**

Flash column chromatography was carried out on silica gel 60 (0.04 - 0.063 mm, 230 - 240 mesh) from Macherey-Nagel GmbH & Co. KG. Automatized flash chromatography was carried on a Biotage Selekt, equipped with either silica column for direct phase chromatography or C18 column for inverse phase chromatography.

**High Performance Liquid Chromatography (HPLC)**

Purification through High Performance Liquid Chromatography (HPLC) was carried out on a Shimadzu, equipped with CBM-40 system controller, DGU-405 degassing unit, LC-20AP pumps for preparative chromatography, SPD-M40 photo-diode-array detector, RF-20A fluorescence detector and FRC-40 fraction collector, using C18 column (250 mm x 10 mm) with 5 µm silica (100 Å pore size) from Macherey-Nagel GmbH & Co. KG. Linear gradient elution for the final purification of the synthetized dyes: Phase A (0.1% TFA in water) and Phase B (MeCN); a 5% B to 80% or 90% B gradient was used at a flow rate of 5 mL/min. Peaks were detected at 520 nm, or 620 nm.

**Nuclear Magnetic Resonance (NMR) Spectroscopy**

The $^{1}H$ and $^{13}C$ NMR spectra of the synthetized compounds are herein reported. All NMR experiments were performed in the analytic department of the Department of Chemistry at the Johannes Gutenberg University Mainz. $^{1}H$ and $^{13}C$ spectra were recorded on either a BRUKER Avance III 600 at a frequency of 600 MHz ($^{1}H$) and 150 MHz ($^{13}C$) or a BRUKER Avance III HD 400 at a frequency of 400 MHz ($^{1}H$) and 100 MHz ($^{13}C$). All $^{1}H$ NMR spectra are reported in parts per million (ppm) downfield of TMS and were measured relative to the $^{1}H$ NMR signals of the deuterated solvent. All $^{13}C$ spectra were reported in ppm relative to

the $^{13}C$ NMR signal of the solvent and were obtained with $^{1}H$ decoupling. The assignment of all $^{1}H$ and $^{13}C$ NMR signals was based on common 2D spectroscopic NMR experiments (DQF-COSY, edHSQC and HMBC). The following abbreviations indicate the multiplicity of $^{1}H$ signals: (s), singlet; (d), doublet; (t), triplet; (q), quartet; (sept), septet; (m), multiplet; and combinations thereof.

**Mass spectrometry (HRMS) – (LCMS)**

Electrospray ionization (ESI) mass spectrometry was performed on a single quadrupole mass spectrometer LC-MS2020 from Shimadzu. For liquid chromatography-mass spectrometry (LC-MS) the mass spectrometer LC-MS2020 from Shimadzu was constituted by two LD-40D pump units, an SPD-M40 photo-diode-array detector, a SIL-40C autosampler, CTO-40C column oven, DGU-405 degassing unit and SCL-40 system controller. High resolution mass spectrometry (HRMS) was performed on a G6545A Q-ToF (Agilent GmbH, Waldbronn, Germany) with ESI. Sample injection was performed via a 1260 Infinity II HPLC system (Agilent GmbH, Waldbronn, Germany) with G7111B 1260 quaternary pump, G7129A 1260 vial sampler, and G7116A 1260 multicolumn thermostat. Mass calibration was performed on the day of measurement using an external standard. HRMS spectra were recorded in the analytic department of the Organic Chemistry at the Johannes Gutenberg University Mainz. Ions are given as mass to charge ratios (m/z).

## Extinction Coefficients and Fluorescence Quantum Yields

Fluorescence and absorbance spectra were recorded using a HORIBA Duetta Fluorescence and Absorbance Spectrometer. Extinction coefficients were determined in PBS buffer (pH = 7.2) by measuring the absorbance at four different concentrations in the range 0.5-10 µM. Quantum yields were determined in PBS buffer (pH = 7.2) relative to Rhodamine B ($\Phi_f$ = 0.31 , λex = 490 nm)[1] for rhodamine dyes, and to ATTO655 ($\Phi_f$ = 0.31 , λex = 600 nm) for (silicon)-rhodamine dyes. Fluorescence turn on was measured as following: for rhodamine dyes, the excitation wavelength was 490 nm, and fluorescence emission was recorded over the 500–800 nm range; for (silicon)-rhodamine dyes, excitation was performed at 600 nm, and emission was collected from 610–800 nm. Quartz cuvettes with path length of 1 cm were used.

## Fluorescence Lifetime Measurements

Fluorescence lifetime of the dye-BCN adducts were conducted on a custom-built confocal microscope equipped with picosecond pulsed laser diode (488 nm: Omicron, Brixx; 660 nm: LDH-D-C-660, PicoQuant) controlled by a Sepia II PDL 828 multichannel laser driver. Excitation light was focused onto the sample using a 60× water-immersion objective (1.27 NA, Nikon). Fluorescence emission was spectrally filtered through a 100 µm pinhole and recorded with a HydraHarp 400 TCSPC module, providing a time resolution of 16 ps. Data were acquired using SymPhoTime 64 software (v2.6, PicoQuant). An instrument response function (IRF) was measured daily using a freshly prepared saturated solution of potassium iodide and Erythrosine B to account for instrumental timing variations. Fluorescent lifetime was calculated using PicoQuant software, by performing initial curve fitting. The value obtained from the initial fit from the software were in accordance with the fitting performed using MATLAB-based PAM software.[2]

## Cloning

Plasmids containing NES-PylRS(AF) (plasmid 1) under the control of a CMV promoter and tRNAPyl (plasmid 2) under the control of a U6 promoter were constructed using restriction ligation. Vimentin (N116TAG)-mCerulean3 (plasmid 3), Vimentin-(TAG)AEAADAEAA(TAG)-mCerulean3 (plasmid 4), Vimentin-(TAG)-iRFP713 (plasmid 5) and Vimentin-(TAG)AEAADAEAA(TAG)-iRFP713 (plasmid 6) amber mutants were constructed using site directed mutagenesis. All assembled plasmids were transformed into electrocompetent TOP10 *E. coli* cells (Invitrogen) and purified using PureLink™ Quick Plasmid MiniPrep-Kit (Invitrogen, K210011) or purified using PureLink™ HiPure Plasmid Maxiprep Kit (Invitrogen, K210007).

Plasmid 3: Vim-(N116TAG)-mCerluean3

MSTRSVSSSSYRRMFGGPGTASRPSSSRSYVTTSTRTYSLGSALRPSTSRSLYASSPGGVYATRSSAVRLRSSVPGVRLLQDS
VDFSLADAINTEFKNTRTNEKVELQELNDRFA*YIDKVRFLEQQNKILLAELEQLKGQGKSRLGDLYEEEMRELRRQVDQL
TNDKARVEVERDNLAEDIMRLREKLQEEMLQREEAENTLQSFRQDVDNASLARLDLERKVESLQEEIAFLKKLHEEEIQEL
QAQIQEQHVQIDVDVSKPDLTAALRDVRQQYESVAAKNLQEAEEWYKSKFADLSEAANRNNDALRQAKQESTEYRRQV
QSLTCEVDALKGTNESLERQMREMEENFAVEAANYQDTIGRLQDEIQNMKEEMARHLREYQDLLNVKMALDIEIATYRK
LLEGEESRISLPLPNFSSLNLRETNLDSLPLVDTHSKRTLLIKTVETRDGQVINETSQHHDDLEGDPPVATMVSKGEELFTGV
VPILVELDGDVNGHKFSVSGEGEGDATYGKLTLKFICTTGKLPVPWPTLVTTLSWGVQCFARYPDHMKQHDFFKSAMPE
GYVQERTIFFKDDGNYKTRAEVKFEGDTLVNRIELKGIDFKEDGNILGHKLEYNAIHGNVYITADKQKNGIKANFGLNCNIE
DGSVQLADHYQQNTPIGDGPVLLPDNHYLSTQSKLSKDPNEKRDHMVLLEFVTAAGITLGMDELYK

Plasmid 4: Vim-Pep1linker-(2xTAG)-mCerluean3

MSTRSVSSSSYRRMFGGPGTASRPSSSRSYVTTSTRTYSLGSALRPSTSRSLYASSPGGVYATRSSAVRLRSSVPGVRLLQDS
VDFSLADAINTEFKNTRTNEKVELQELNDRFANYIDKVRFLEQQNKILLAELEQLKGQGKSRLGDLYEEEMRELRRQVDQL
TNDKARVEVERDNLAEDIMRLREKLQEEMLQREEAENTLQSFRQDVDNASLARLDLERKVESLQEEIAFLKKLHEEEIQEL
QAQIQEQHVQIDVDVSKPDLTAALRDVRQQYESVAAKNLQEAEEWYKSKFADLSEAANRNNDALRQAKQESTEYRRQV
QSLTCEVDALKGTNESLERQMREMEENFAVEAANYQDTIGRLQDEIQNMKEEMARHLREYQDLLNVKMALDIEIATYRK
LLEGEESRISLPLPNFSSLNLRETNLDSLPLVDTHSKRTLLIKTVETRDGQVINETSQHHDDLEGDPGGSG*AEAADAEAA*
SGGSGPVATMVSKGEELFTGVVPILVELDGDVNGHKFSVSGEGEGDATYGKLTLKFICTTGKLPVPWPTLVTTLSWGVQC
FARYPDHMKQHDFFKSAMPEGYVQERTIFFKDDGNYKTRAEVKFEGDTLVNRIELKGIDFKEDGNILGHKLEYNAIHGNV
YITADKQKNGIKANFGLNCNIEDGSVQLADHYQQNTPIGDGPVLLPDNHYLSTQSKLSKDPNEKRDHMVLLEFVTAAGIT
LGMDELYK

Plasmid 5: Vim-Pep1linker-(TAG)-iRFP713

MSTRSVSSSSYRRMFGGPGTASRPSSSRSYVTTSTRTYSLGSALRPSTSRSLYASSPGGVYATRSSAVRLRSSVPGVRLLQDS
VDFSLADAINTEFKNTRTNEKVELQELNDRFANYIDKVRFLEQQNKILLAELEQLKGQGKSRLGDLYEEEMRELRRQVDQL
TNDKARVEVERDNLAEDIMRLREKLQEEMLQREEAENTLQSFRQDVDNASLARLDLERKVESLQEEIAFLKKLHEEEIQEL
QAQIQEQHVQIDVDVSKPDLTAALRDVRQQYESVAAKNLQEAEEWYKSKFADLSEAANRNNDALRQAKQESTEYRRQV
QSLTCEVDALKGTNESLERQMREMEENFAVEAANYQDTIGRLQDEIQNMKEEMARHLREYQDLLNVKMALDIEIATYRK
LLEGEESRISLPLPNFSSLNLRETNLDSLPLVDTHSKRTLLIKTVETRDGQVINETSQHHDDLEGDPGGSG*AEAADAEAAY
SGGSGAEGSVARQPDLLTCDDEPIHIPGAIQPHGLLLALAADMTIVAGSDNLPELTGLAIGALIGRSAADVFDSETHNRLTI
ALAEPGAAVGAPITVGFTMRKDAGFIGSWHRHDQLIFLELEPPQRDVAEPQAFFRRTNSAIRRLQAAETLESACAAAAQE

VRKITGFDRVMIYRFASDFSGEVIAEDRCAEVESKLGLHYPASTVPAQARRLYTINPVRIIPDINYRPVPVTPDLNPVTGRPI
DLSFAILRSVSPVHLEFMRNIGMHGTMSISILRGERLWGLIVCHHRTPYYVDLDGRQACELVAQVLAWQIGVMEE

Plasmid 6: Vim-Pep1linker-(2xTAG)-iRFP713

MSTRSVSSSSYRRMFGGPGTASRPSSSRSYVTTSTRTYSLGSALRPSTSRSLYASSPGGVYATRSSAVRLRSSVPGVRLLQDS
VDFSLADAINTEFKNTRTNEKVELQELNDRFANYIDKVRFLEQQNKILLAELEQLKGQGKSRLGDLYEEEMRELRRQVDQL
TNDKARVEVERDNLAEDIMRLREKLQEEMLQREEAENTLQSFRQDVDNASLARLDLERKVESLQEEIAFLKKLHEEEIQEL
QAQIQEQHVQIDVDVSKPDLTAALRDVRQQYESVAAKNLQEAEEWYKSKFADLSEAANRNNDALRQAKQESTEYRRQV
QSLTCEVDALKGTNESLERQMREMEENFAVEAANYQDTIGRLQDEIQNMKEEMARHLREYQDLLNVKMALDIEIATYRK
LLEGEESRISLPLPNFSSLNLRETNLDSLPLVDTHSKRTLLIKTVETRDGQVINETSQHHDDLEGDPGGSG*AEAADAEAA*
SGGSGAEGSVARQPDLLTCDDEPIHIPGAIQPHGLLLALAADMTIVAGSDNLPELTGLAIGALIGRSAADVFDSETHNRLTI
ALAEPGAAVGAPITVGFTMRKDAGFIGSWHRHDQLIFLELEPPQRDVAEPQAFFRRTNSAIRRLQAAETLESACAAAAQE
VRKITGFDRVMIYRFASDFSGEVIAEDRCAEVESKLGLHYPASTVPAQARRLYTINPVRIIPDINYRPVPVTPDLNPVTGRPI
DLSFAILRSVSPVHLEFMRNIGMHGTMSISILRGERLWGLIVCHHRTPYYVDLDGRQACELVAQVLAWQIGVMEE

## HEK293T culture, transfection and labeling

HEK293T (ATCC, CRL-3216) cells were maintained in Dulbecco's modified Eagle's medium (DMEM, Gibco 41965-039) supplemented with 10% v/v fetal bovine serum (FBS) (Sigma-Aldrich F7524), 1% penicillin-streptomycin (Sigma-Aldrich P0781), 1% L-Glutamine (Sigma-Aldrich G7513), and 1% sodium pyruvate (Life Technologies 11360). Cells were cultured at 37°C in a 5% $CO_2$ atmosphere and passaged every 2-3 days up to 15 passages. Cells were trypsinized (trypsin-EDTA) and seeded at $5.5*10^4$ cells into a µ-Slide eight-well glass bottom (80827, ibidi) coated with poly-l-lysine, 15-20 h before transfection. The cells were transfected at a confluency of 60–70% with plasmids of interest. Plasmid 1, 2 and plasmid 3 or 4 were transfected at a total DNA ratio of 1:1:1 (200 ng for each well), using polyethylenimine (PEI), as transfecting agent and DMEM for PEI as transfecting buffer, according to the manufacturer's protocol. After 4 h, the medium was changed, and 10 mM HEPES with 250 µM *(endo)*-BCN-*L*-lysine (100 mM stock of BCNK, prepared according to the manufacturer's protocol - Sichem, SC-8014) was added (or Boc-*L*-lysine for the negative control). Cells were grown for 24h and then washed for two hours with fresh medium to remove residual BCNK. Cells were labeled for 30 min using 2 µM (Si)Rh-CTet dyes in DMEM at RT. After the labelling, the media was replaced only one time with fresh culture medium. Cells were imaged immediately at RT in FluoroBrite DMEM buffer. The lid of the imaging dish was kept closed for the duration of cell imaging (2-3 h).

<u>Co-transfections with both single-amber constructs together or with the single-amber and double-amber constructs combined (as in Fig. 8 and Fig. S39).</u> For the double single-amber mix, plasmids 1, 2, 3, and 5 were transfected at a total DNA ratio of 1:1:0.6:0.4, where one ratio unit corresponded to 200 ng DNA per well. Polyethylenimine (PEI) was used as the transfection reagent, and DMEM served as the PEI dilution buffer. For the single-amber and double-amber combinations, plasmids 1, 2, 4, and 5 were transfected at a total DNA ratio of 1:1:0.6:0.4 (1 ratio unit = 200 ng DNA per well), using PEI as the transfection reagent and DMEM as the PEI dilution buffer. All remaining steps were performed as previously described.

## Live-cell imaging

Confocal images were acquired on a Leica TCS SP5 microscope using 405 nm (laser power 10%, for mCerulean), 514 nm (laser power 20%, for **Rh506monoCTet-BCN** and **Rh518bisCTet-BCN**) and 633 nm (laser power 15% for **Si-Rh628monoCTet-BCN** and iRFP) laser lines for excitation. Emission light was collected with detectors at 420-480 nm for mCer, 525-625 nm for **Rh506monoCTet-BCN** and **Rh518bisCTet-BCN**, 643-800 nm for **Si-Rh628monoCTet-BCN** and 700-800 nm for iRFP. The images were taken using Leica 63x/1.4 oil immersion objective.

## HeLa culture and linear and cyclic peptide treatment

Hela cells were maintained in Dulbecco's modified Eagle's medium (DMEM+++) supplemented with 10% v/v FBS (Sigma-Aldrich F7524), 1% penicillin-streptomycin (Sigma-Aldrich P0781) and 1% l-glutamine (Sigma-Aldrich G7513) at 37 °C and 5% $CO_2$, and passaged every 2-3 days up to 15-20 passages. The Hela cells were authenticated by the manufacturer, validated by morphology and regularly tested for mycoplasma contamination, with negative results. Cells were seeded 15-20 h prior to peptide-dye adduct treatment at a density of $2.2*10^4$ cells in 8-well plates for microscopy or in 48-well plates for flow cytometry (FC). After 16-18h the cells were treated with **Rh518bisCTet**-peptide adduct (Pep1-Pep4) and with **Rh518bisCTet-SingleTet**-peptide adduct (Pep9). Pep-dye adduct were prepared by mixing **Rh518bisCTet** (from 2mM DMSO stock solution) with 5 equiv of each peptide in FluoroBrite DMEM to obtain a final dye concentration of 8 µM. The reaction mixtures were incubated for 2-3 h at room temperature. Subsequently, the conjugate solutions were diluted with DMEM to obtain final concentrations of 1, 2, and 5 µM. For Dynasore treatment experiments, the 8 µM dye-peptide conjugate solutions were diluted to a final conjugate concentration of 5 µM and supplemented with Dynasore (a dynamin inhibitor) to a final concentration of 80 µM. Thus, HeLa cells were treated with dye-peptide conjugates at different concentration and in the absence or presence of Dynasore. As a negative control for Dynasore, **Rh518bisCTet** was fully reacted with 5 equiv of BCN-OH and the resulting free dye solution was added to the medium at a final concentration of 5 µM, both in the absence and presence of Dynasore. Medium was exchanged after 3-3.30 h, and then the samples prepared according to the flow cytometry protocol (see next paragraph). For microscopy the dye-peptide conjugates were added to HeLa cells at a 4 µM final concentration in DMEM+++. After 3 h, cells were washed with FluoroBrite DMEM and were imaged immediately at room temperature. The lid of the imaging dish was kept closed for the duration of cell imaging (2-3 h). Stability of the dye-peptide conjugates was tested in different conditions, showing high stability in PBS pH 7.2, FluoroBrite DMEM and DMEM+++ (see Figure S15-22 and S26). Fluorescence output given by **Rh518bisCTet**-Pep1 and **Rh518bisCTet-SingleTet**-Pep9 was also evaluated after incubation under conditions simulating the FC protocol (Figure S26).

## Flow Cytometry (FC) of HeLa cells

HeLa cells were seeded at $2.2*10^4$ cells in 48-well plates. After 16-18h they were treated with **Rh518bisCTet**-peptide adduct (Pep1-Pep4) and with **Rh518bisCTet-SingleTet**-peptide adduct (Pep9) for 2-3 h. Pep-dye adduct were prepared by mixing **Rh518bisCTet** (from 2mM DMSO stock solution) with 5 equiv

of each peptide in FluoroBrite DMEM at a concentration of 8 µM, kept to react for 2-3 h at RT, before diluting with DMEM medium to the respective concentration, 1µM, 2 µM, 5µM. Medium was exchanged after 3-3.30 h, and then prepared for FC analysis. Cells were rinsed once with 100 µL of 1× PBS, detached using 50 µL of trypsin-EDTA (0.05%, with phenol red, Gibco, 25300-054) and incubated for 5 min at 37 °C and 5% $CO_2$. Next, 450 µL of resuspension buffer 1 (1× PBS containing 10% FBS, 2 mM sodium azide, and 2 mM EDTA) was added to each well, and the cells were collected on ice, transferred to 1.5 mL tubes, and centrifuged for 5 min at 400 × *g* and 4 °C. The supernatants were discarded, and the pellets were washed with 450 µL of resuspension buffer 2 (1× PBS containing 3% bovine serum albumin (BSA), 2 mM sodium azide, and 2 mM EDTA). Then, the cells were again centrifuged for 5 min at 400 × *g* and 4 °C. The supernatants were discarded and the cell pellets were resuspended in 200 µL of resuspension buffer 2. Untreated cells and cells treated with only 80 µM of Dynasore were used as controls for fluorescence background subtraction for their respective samples. Before measurement 2 µL of DAPI (50 µg/mL) were added to the cell suspension for live cell staining, followed by incubation of the samples for 1-2 min on ice before measurement. Data acquisition was performed with an LSRFortessa Cell Analyzer (BD Biosciences), using the following voltage: 319 V for FSC, 206 V for SSC, 258 V for 405nm laser and 391 V for 488 nm laser. The fluorescence of **Rh518bisCTet**-peptide adduct was acquired using a 488 nm laser and a 530/30 bandpass filter. For detecting DAPI, a 405 nm laser with a 450/50 bandpass filter was used. Analysis was performed using FlowJo version 10.7.1 (BD Biosciences). First, the population of HeLa cells was gated (using FSC-A x SSC-A parameters), and then a single cell population was selected (FSC-W x FSC-A). Next, live cells were picked (FSC-A x 405–450/50 channel). At least 10,000 events were collected and analyzed. At least three experimental (different passage/day) replicates were performed: repetitions were made respectively at passages 7, 9, 10 and 12.

## Flow cytometry (FC) of HEK293T cells

HEK283T cells were seeded at $5.5*10^4$ cells in 48-well plates, 15-20 h before transfection. The cells were transfected at a confluency of 60-70% with plasmids of interest. In this case plasmid 5 and 6 with iRFP713 were necessary in order to not have cross-talk in the channel between mCer and dye, due to a low absorbance of mCer at 488 nm, which might impair the BocK control, and in order to use DAPI as live cell control. Plasmid 1, 2 and plasmid 5 or 6 were transfected at a total DNA ratio of 1:1:1 (200 ng for each well), using polyethylenimine (PEI), as transfecting agent and DMEM for PEI as transfecting buffer, according to the manufacturer's protocol. After 4 h, the medium was changed, and 10 mM HEPES with 250 µM (*endo)*-BCN-*L*-lysine (100 mM stock of BCNK, prepared according to the manufacturer's protocol - Sichem, SC-8014) was added (or Boc-*L*-lysine for the negative control). Cells were grown for 36h and then washed for two hours with fresh medium to remove residual BCNK. Cells were labeled for 30 min using 2 µM (Si)Rh-CTet dyes in DMEM at RT. After the labelling, samples were prepared for FC analysis. Cells were rinsed once with 100 µL of 1× PBS, detached using 50 µL of trypsin-EDTA (0.05%, with phenol red, Gibco, 25300-054) and incubated for 5 min. Next, 450 µL of resuspension buffer 2 was added to each well, and the cells were collected on ice, transferred to 1.5 mL tubes, and centrifuged for 5 min at 400 × *g* and 4 °C. The supernatants were discarded, and the pellets were washed again with 450 µL of resuspension buffer 2. Then, the cells were again centrifuged for 5 min at 400 × *g* and 4 °C. The supernatants were discarded and the cell pellets were resuspended in 200 µL of resuspension buffer 2. Untreated cells and cells treated

with plasmids in the presence of only BCNK were used as controls for fluorescence background subtraction and for selecting iRFP positive cells. Before measurement 2 µL of DAPI (50 µg/mL) were added to the cell suspension for live cell staining, followed by incubation of the samples on ice before measurement. Data acquisition was performed with an LSRFortessa Cell Analyzer (BD Biosciences), using the following voltage: 319 V for FSC, 206 V for SSC, 258 V for 405nm laser, 250 V for 488 nm laser, 200 V for 561 nm laser and 350 V for 640 nm laser. The fluorescence of the dyes was acquired using a 488 nm laser and a 530/30 bandpass filter for **Rh506monoCTet** and **Rh518bisCTet**; using a 561 nm laser and a 586/15 bandpass filter for **Rh538bisCTet**; and using a 640 nm laser and a 730/45 bandpass filter for iRFP. For detecting DAPI, a 405 nm laser with a 450/50 bandpass filter was used. Analysis was performed using FlowJo version 10.7.1 (BD Biosciences). First, the population of HEK293T cells was gated (using FSC-A x SSC-A parameters), and then a single cell population was selected (FSC-W x FSC-A). Next, live cells were picked (FSC-A x 405-450/50 channel), subsequently, iRFP-positive cells were selected from transfected samples cultured in the presence of BCNK and in the absence of dye (FSC-A x 640-730/45 channel), as described in Figure S40. At least 10,000 events were collected and analyzed. At least two experimental (different passage/day) replicates were performed: repetitions were made respectively at passages 6, 9 and 12.

## MBP protein expression and purification

A Maltose binding protein domain (MBP) construct, containing a FLAG-Tag as well as a short linker (AEAADAEAA, from Pep1) in front of the MBP sequence was prepared. The FLAG-tag and the linker were cloned by conventional overlap PCR and ligated into a pBAD-MBP-8His plasmid using restriction enzymes. Variants, which harbored one or two Amber STOP codons instead of the lysine residues in the short linker sequence (KAEAADAEAA-TAG and TAG-AEAADAEAA-TAG) were cloned by site directed mutagenesis. The plasmids were co-transformed together with pEvol-PylRSAF into Bl21(DE3) AI cells (Invitrogen). Expression was done in 500 mL TB medium adding 100 µg/mL Ampicillin (for the pBAD plasmid), 33 µg/mL Chloramphenicol (for the pEvol plasmid) and 5 mL (for SCO- or TCO-expression) and 10 mL (for BCN-expression) of the corresponding overnight culture, shaking at 37 °C. When an $OD_{600}$ of 0.2-0.35 was reached, SCO, TCO*AK or BCNK was added to a final concentration of 1mM (the non-canonical amino acids were prepared according to the manufacture´s protocol, by dissolving them in 0.2 N NaOH with 10% DMSO). The protein expression was induced with 0.02 % Arabinose at an $OD_{600}$ of 0.4–0.6. After 8–12 hours of incubation at 37 °C (for BCN-MBP expression, incubation was carried for 9 h at 30 °C), the cells were harvested by centrifugation (30 min at 4500 rcf). The pellets were taken up in 5 mL (SCO- or TCO-expression) or 20 mL (BCN-expression) of lysis buffer (4×PBS, 0.2 mM TCEP, 1 mM PMSF, 5 mM imidazole) and sonicated on ice for 3×30 seconds. After one hour centrifugation step at 4 °C at 20000 rpm in a Beckman JA-25.50 rotor, the cleared supernatant was incubated on nickel beads and washed with lysis buffer. After 1-2 hour, the nickel beads were collected in polypropylene columns, and the protein was further purified with 10 mM imidazole in the lysis buffer and finally eluted with 500 mM imidazole in lysis buffer. To improve the purity of the protein, size-exclusion chromatography (SEC) was used. The collected fraction were concentrated down, buffer was exchanged with SEC buffer containing 50% glycerol (1×PBS, 0.2 mM TCEP) and afterwards the protein was flash-frozen in liquid nitrogen and stored at -80 °C. Protein concentration was estimated using a UV-spectrometer.[3] Single-TAG-BCN-MBP was obtained at a concentration of 82 µM (total volume 450 µL), with a protein yield of 1.57 mg per 500 mL expression.

Double-TAG-BCN-MBP was obtained at a concentration of 26 µM (total volume 520 µL), with a protein yield of 0.57 mg per 500 mL expression. Double-TAG-TCO-MBP was obtained at a concentration of 182 µM (total volume 55 µL), with a protein yield of 0.43 mg per 500 mL expression.

## Anisotropy Measurement

Fluorescence anisotropy measurements were performed using a HORIBA Duetta Fluorescence and Absorbance Spectrometer equipped with detection channels for parallel and perpendicular emission. Fluorescence anisotropy steady-state was calculated as:

$$r = \frac{(I \parallel - GI \perp)}{(I \parallel + G2I \perp)}$$

where *r* is the fluorescence anisotropy, *G* is the g-factor, and $I\parallel$ and $I\perp$ are the fluorescence intensities in the parallel and perpendicular axes, respectively. Yellow fluorescent protein (YFP) was used as a reference standard. The instrumental *G*-factor was determined from YFP measurements (500 nM in PBS) and found to be 1.0103 ± 0.0086, across different day measurements, which was subsequently applied to the anisotropy calculations day by day. Measurements were carried out as replicate, and YFP and the *G*-factor were measured daily to ensure instrument consistency. The double-labeled MBP construct (TCO-peptide-TCO-MBP) was diluted in PBS (pH 7.2) to a final concentration of 1 µM. The fluorescent dye was added to a final concentration of 5 µM, and the reaction mixture was incubated for 5 min prior to anisotropy measurements. The double-labeled-BCN MBP construct (BCN-peptide-BCN-MBP) was diluted in PBS (pH 7.2) to a final concentration of 1 µM. The fluorescent dye was added to a final concentration of 5 µM (concentration of dyes up to 8 µM were also tested), and the reaction mixture was incubated for 40-60 min prior to anisotropy measurements. Measurements were performed in a 1 cm path length cuvette with a total volume of 120 µL, using excitation at 514 nm and emission detection at 527 nm. To determine the steady-state anisotropy of the free dyes, samples were incubated with BCN-OH for 1 h prior to measurement. The same procedure was applied to the commercially available ATTO488-MeTet dye. Time-dependent anisotropy was also measured for both TCO- and BCN-labeled proteins. Specifically, for all the TCO-derivatives data were acquired at time 5, 15 and 45 min, while for the BCN-derivates data were acquired at time 30, 210 and 390 min. The *r* value was also calculated for the clicked peptides, Pep1 and Pep9, using 500 nM concentration in PBS and 5 equiv of the dye (see Figure S31). All measurements have been carried out as at least three replicates.

## Click reaction between synthetized dyes and BCN-OH

Commercially available (1α, 8α, 9β)-bicyclo[6.1.0]non-4-yne-9- methanol (BCN-OH) was dissolved in DMSO to prepare a 50 mM stock solution. The synthetized dyes were dissolved in DMSO to prepare 2 mM stock solutions. For the click reaction, 0.48 µl of the dye stock solution was diluted in PBS (pH 7.2) to 120 µL and absorbance and emission spectra were measured (final dye concentration 8 µM, due to high fluorogenic response after click reaction for **Rh506monoCTet** a 6 µM solution was prepared). Then, 0.48 µL or 0.96 µL of 50 mM BCN-OH solution (25 equiv) were added to the solution of the mono- and bis-dye, respectively. The mixtures were kept for 1 or 2 hours at room temperature in the dark. In case of silicon-rhodamine dye 0.1% SDS PBS was used.

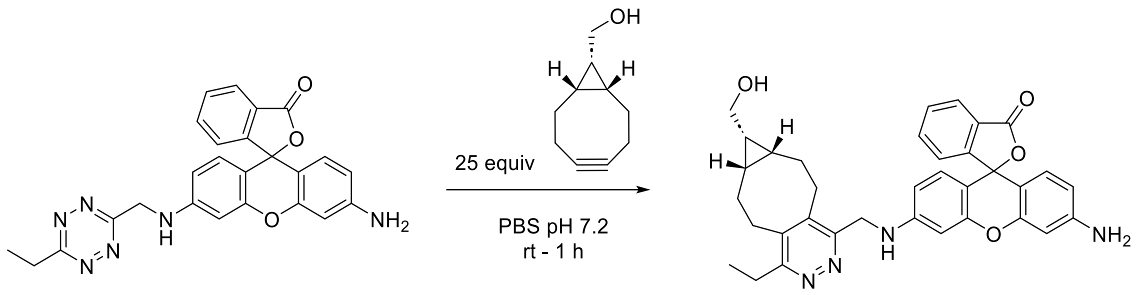


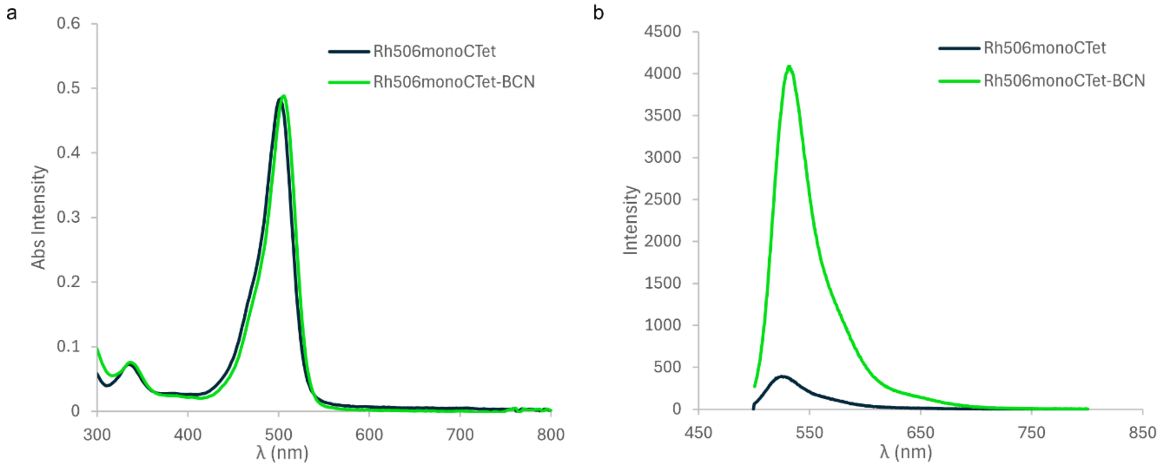


**Figure S1**. **Spectroscopic data of Rh506monoCTet.** a) Absorbance and b) emission spectra of **Rh506monoCTet** before and after click reaction with BCN-OH in PBS (dye concentration 6 µM).

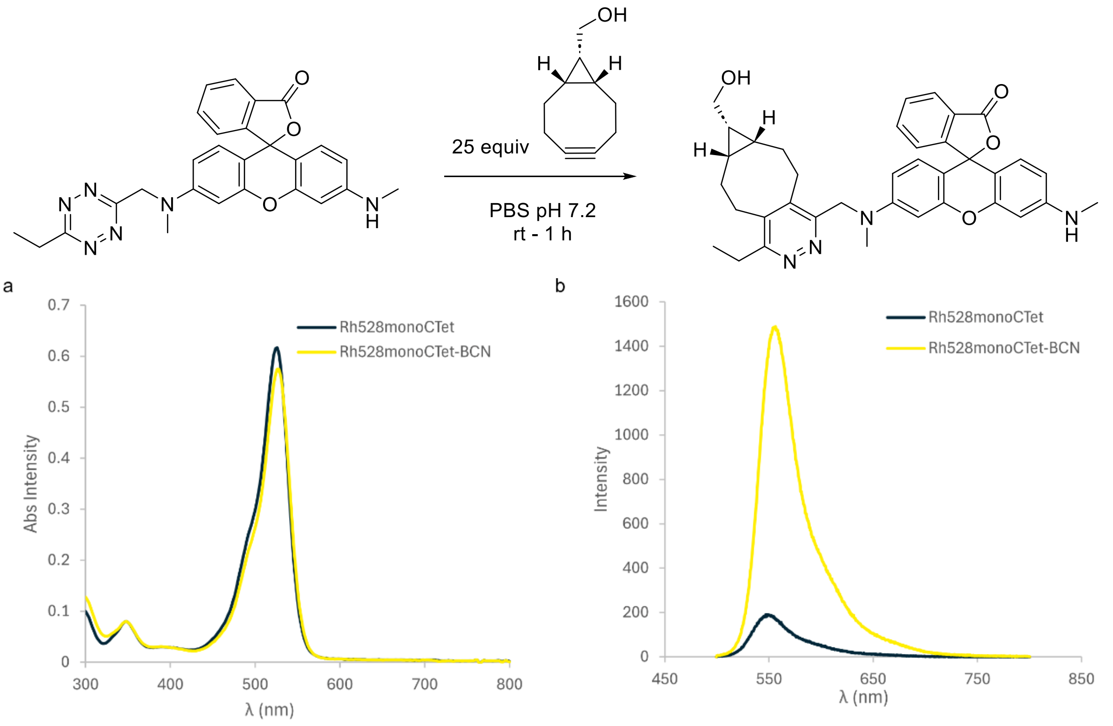


**Figure S2**. **Spectroscopic data of Rh528monoCTet.** a) Absorbance and b) emission spectra of **Rh528monoCTet** before and after click reaction with BCN-OH in PBS (dye concentration 8 µM).

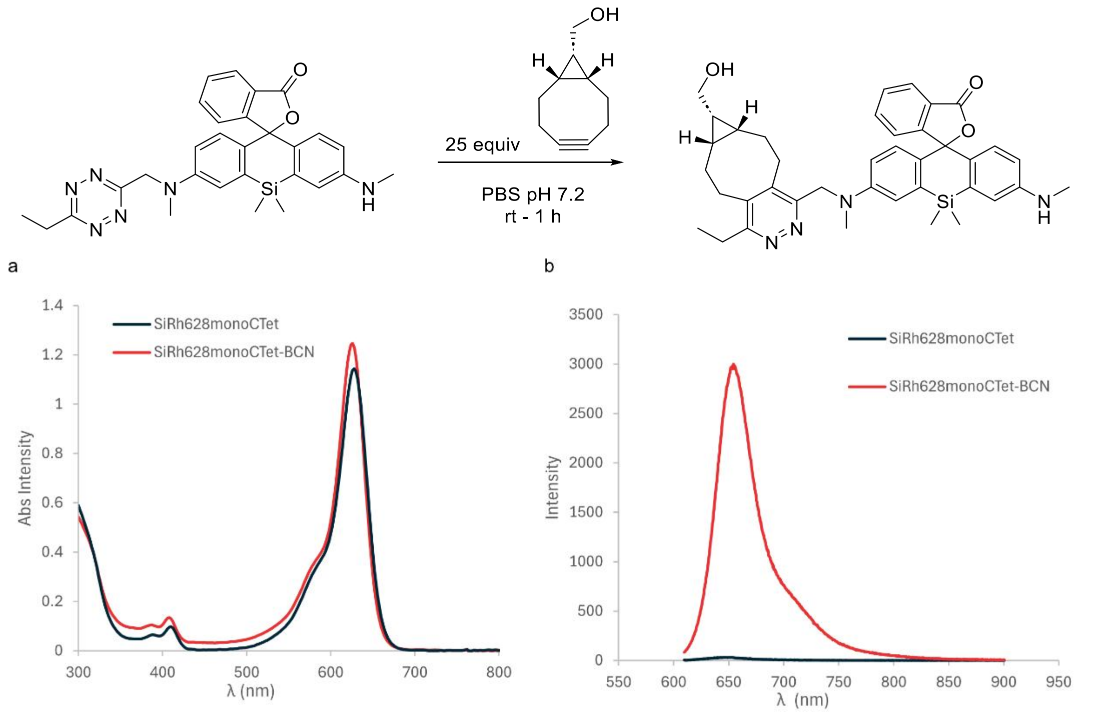


**Figure S3**. **Spectroscopic data of SiRh628monoCTet.** a) Absorbance and b) emission spectra of **SiRh628monoCTet** before and after click reaction with BCN-OH in 0.1% SDS PBS (dye concentration 8 µM).

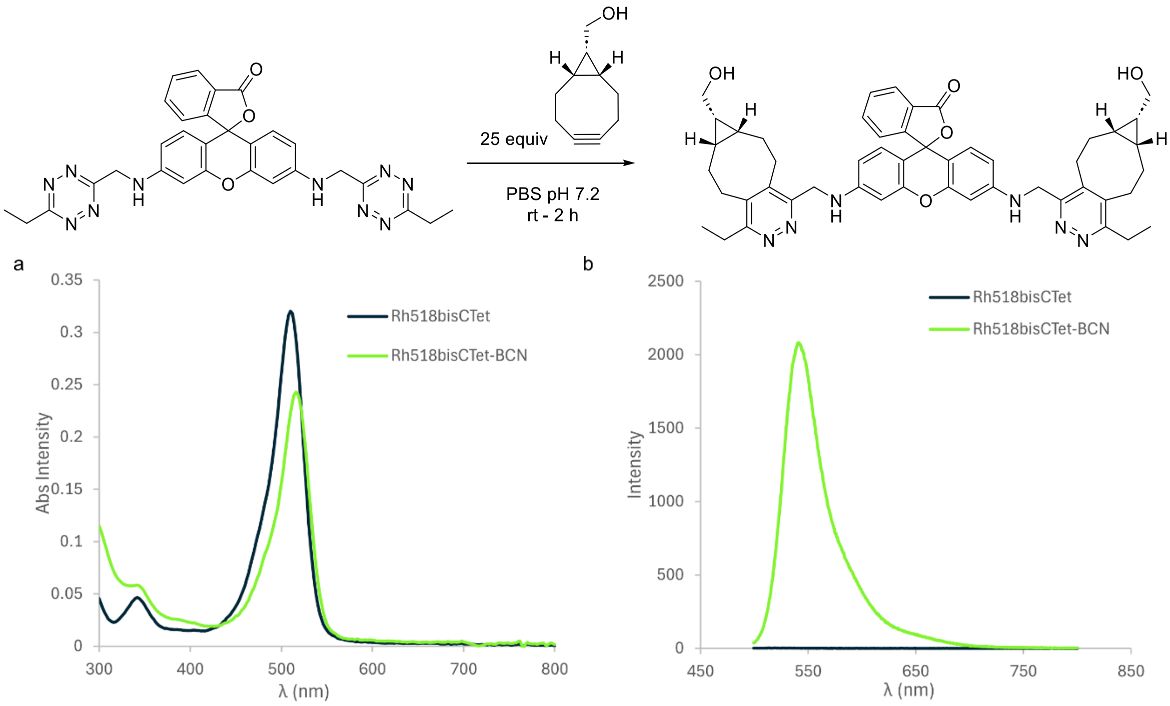


**Figure S4**. **Spectroscopic data of Rh518bisCTet.** a) Absorbance and b) emission spectra of **Rh518bisCTet** before and after click reaction with BCN-OH in PBS (dye concentration 8 µM).

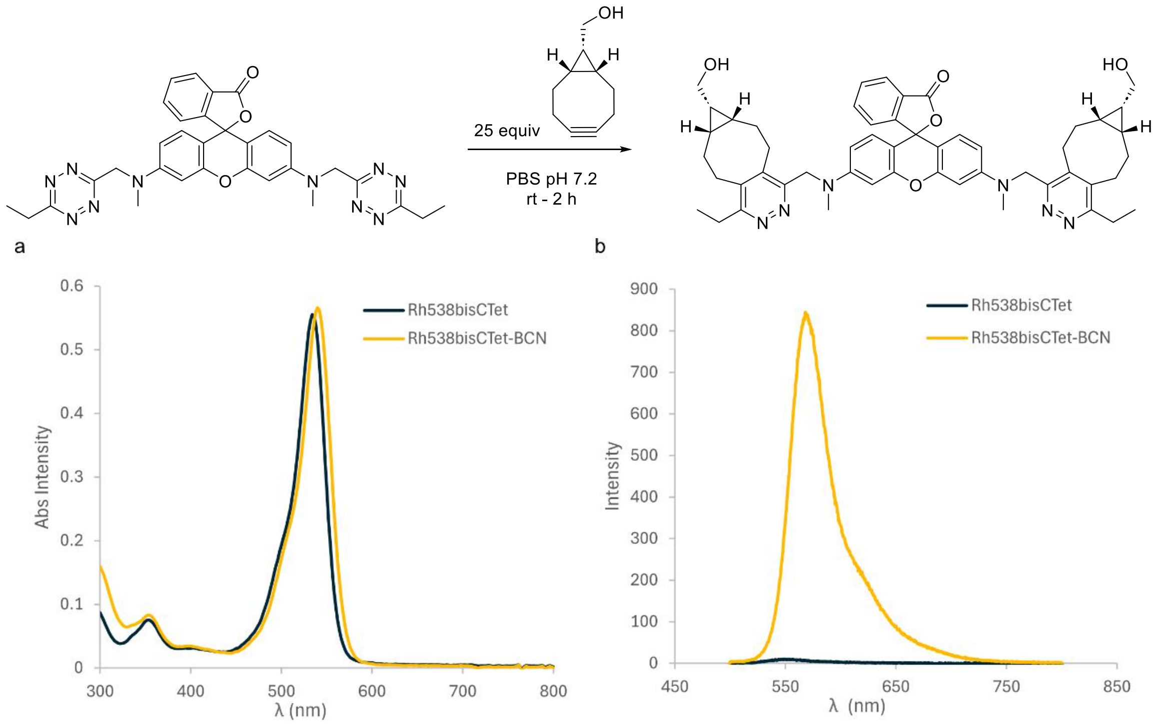


**Figure S5**. **Spectroscopic data of Rh538bisCTet.** a) Absorbance and b) emission spectra of **Rh538bisCTet** before and after click reaction with BCN-OH in PBS (dye concentration 8 µM).

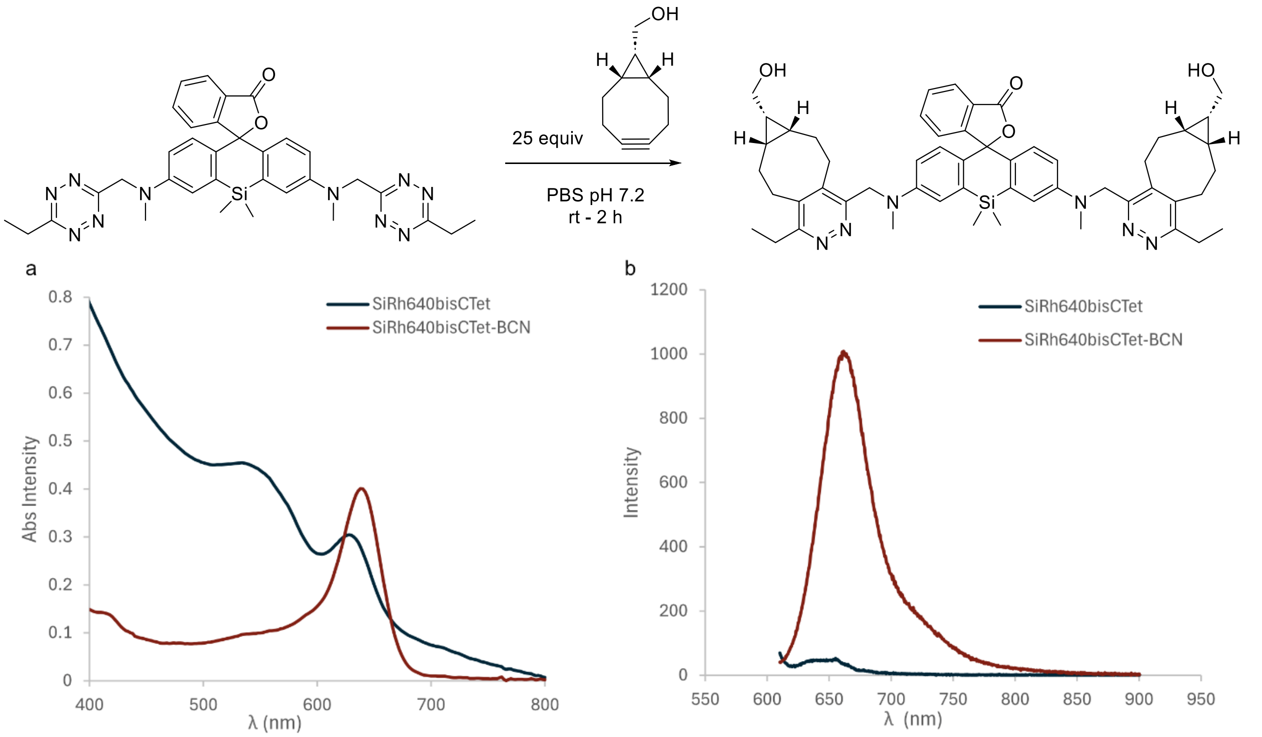


**Figure S6**. **Spectroscopic data of SiRh638bisCTet.** a) Absorbance and b) emission spectra of **SiRh640bisCTet** before and after click reaction with BCN-OH in 0.1% SDS PBS (dye concentration 8 µM).

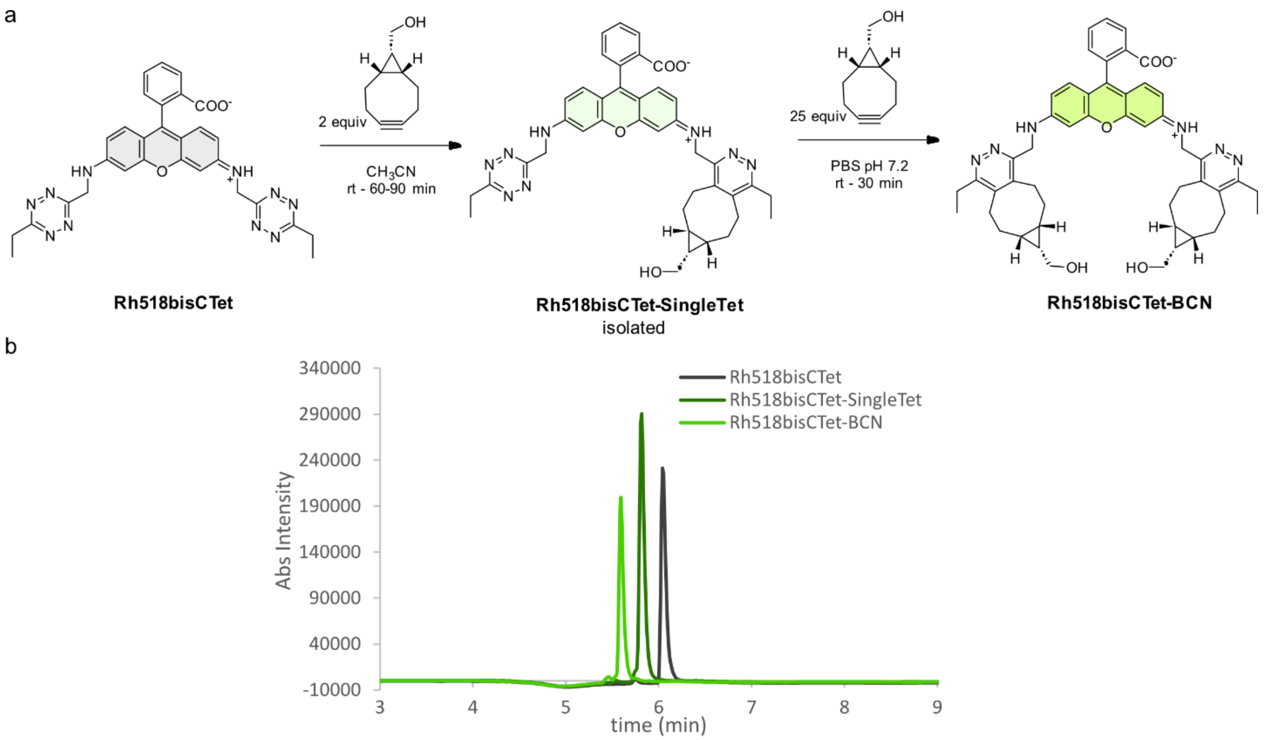


**Figure S7.** Stepwise reaction of **Rh518bisCTet** with BCN-OH. a) Click reaction between **Rh518bisCTet** and 2 equiv of BCN-OH to provide the mono clicked intermediate **Rh518bisCTet-SingleTet**, followed by incubation with 25 equiv BCN-OH in PBS (pH = 7.2) to get the bis-derivative **Rh518bisCTet-BCN**. b) HPLC chromatogram of the three different compounds.

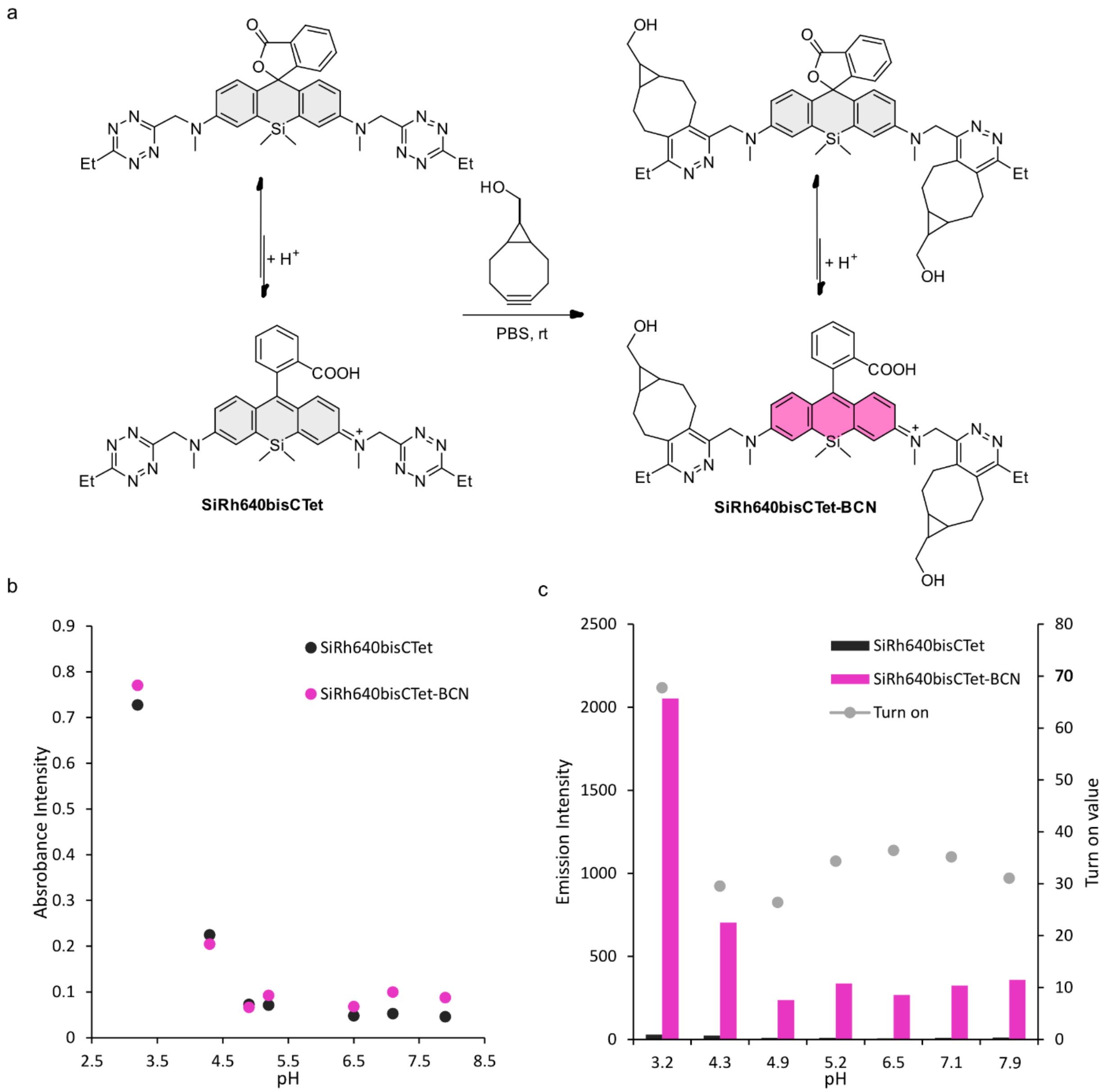


**Figure S8. Spirolactone-zwitterion equilibria of SiRh640bisCTet and its respective cycloadduct, SiRh640bisCTet-BCN**. (a) Molecular mechanism of fluorescence on/off switching and turn-on. (b) pH-dependent absorbance of 4 µM dye solutions in sodium phosphate buffers referring to the absorbance peak at 640 nm. (c) pH-dependent emission of 4 µM dye solutions in sodium phosphate buffers with the relative emission turn-on between “unclicked” (black) and “clicked” (fuchsia).

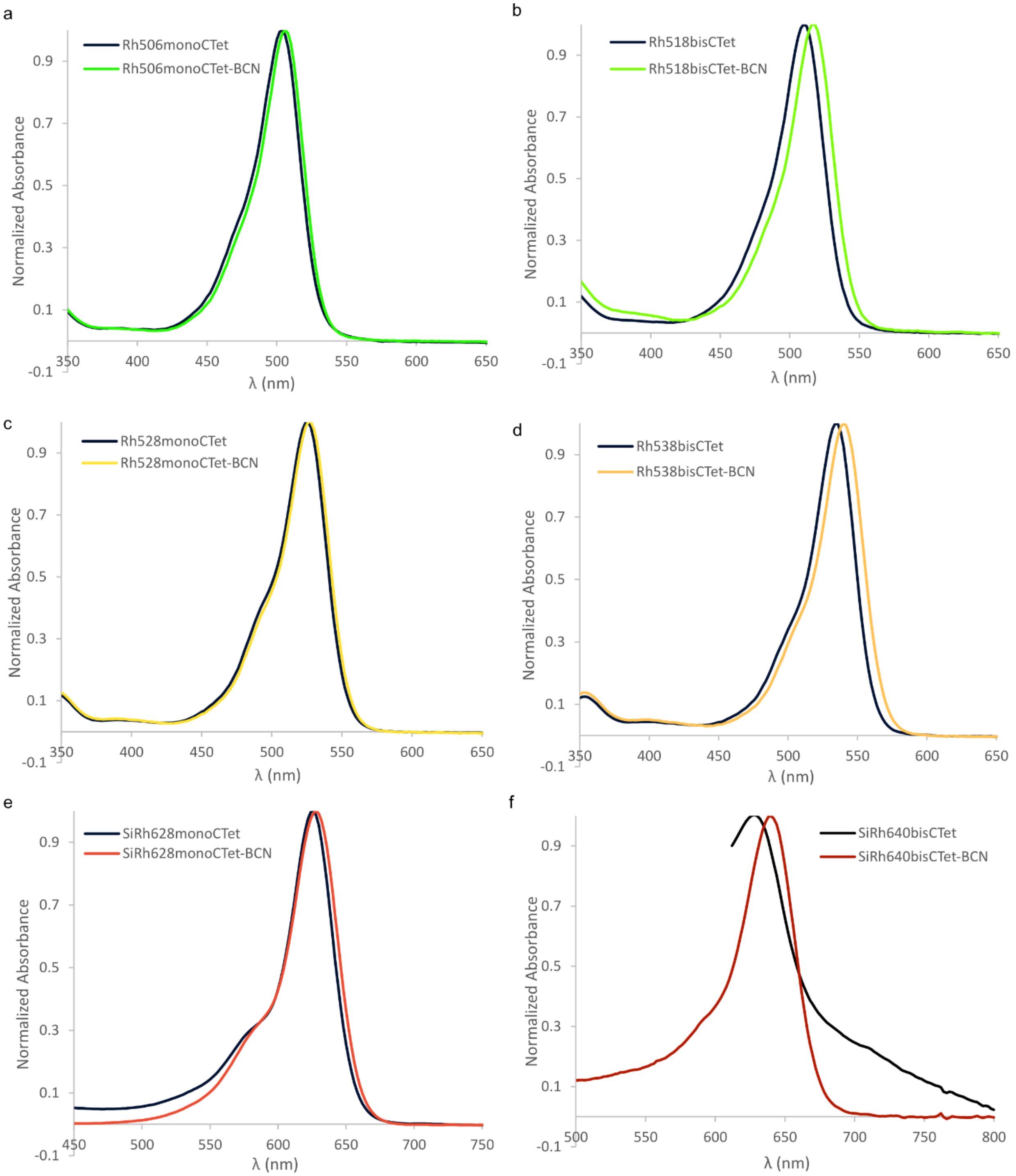


**Figure S9.** Normalized absorbance before and after click reaction with BCN-OH of the synthetized mono- and bis-functional dyes. Enhanced bathochromic shift is observed for the bis-derivatives ($\Delta\lambda_{em}$ = 6 nm, b-d) in comparison to the mono-dyes ($\Delta\lambda_{em}$ = 2 nm, a,c,e). f) For the silicon-rhodamine, **SiRh640bisCTet**, $\Delta\lambda_{em}$ = 10 nm is observed. Since the compound before click reaction is mainly in the closed form (see Figure S6) without major peak at ≈ 630-640 nm, the absorbance spectrum has been selectively cut in order to normalize the peak to the peak of interest.

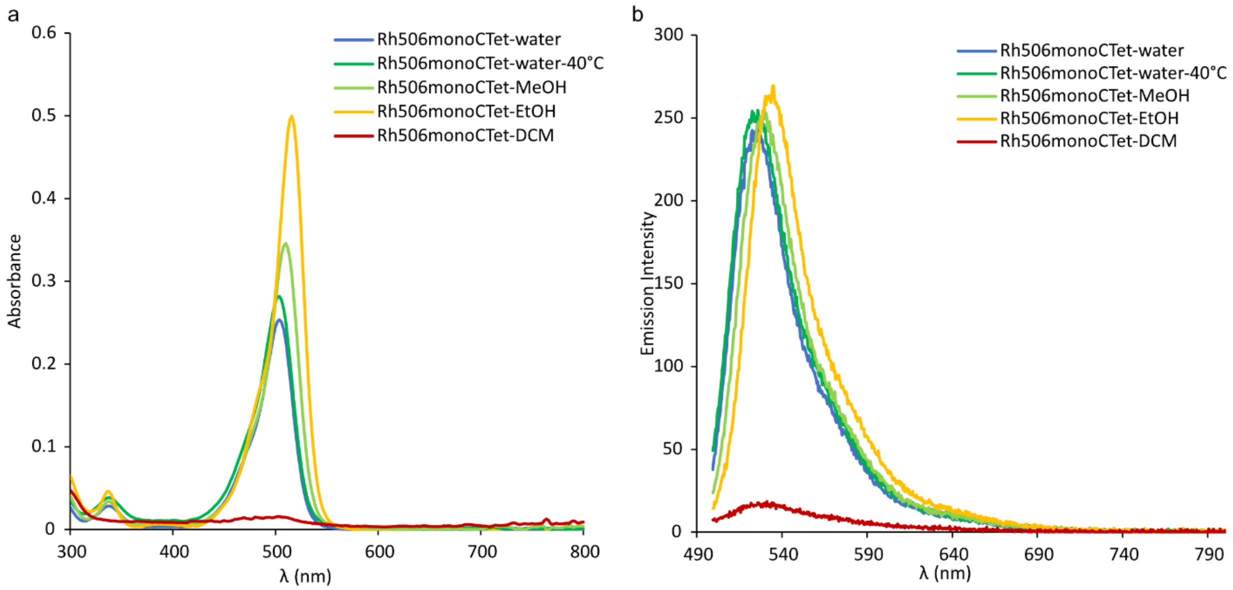


**Figure S10. Solvent effect on the absorbance and emission spectra of unclicked Rh506monoCTet.** Solvent effect on the a) absorbance spectrum and b) fluorescence quenching of **Rh506monoCTet**. Starting from the same stock concentration of dye ($c$ = 500 µM), final concentrations ($c$ = 5 µM) of **Rh506monoCTet** were prepared in the desired solvent. In dichloromethane (DCM) no emission was observed due to the shift to the closed spirolactone form.

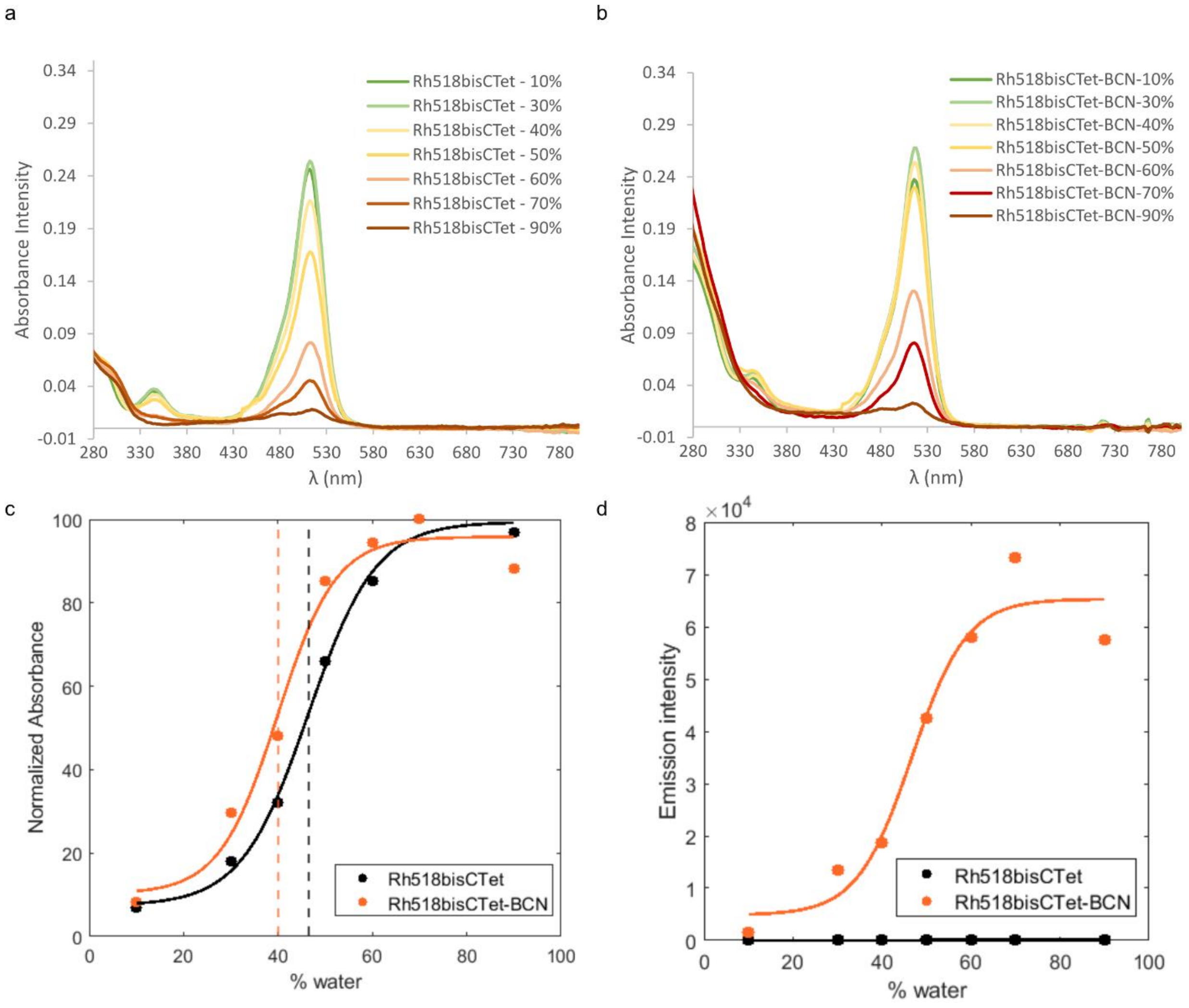


**Figure S11. Solvatochromic spirocyclization properties of Rh518bisCTet and its BCN adduct.** a) Absorbance spectra of **Rh518bisCTet** in different dioxane/water mixtures (v/v; 10/90 to 90/10, percentage relative to dioxane) measured at *c* = 5 µM; b) absorbance spectra of **Rh518bisCTet-BCN** in different dioxane/water mixtures (v/v; 10/90 to 90/10, percentage relative to dioxane), measured at a *c* = 5 µM; c) Normalized absorbance at $\lambda_{max}$ of **Rh518bisCTet** and respective cycloadduct as a function of the dielectric constant of water/dioxane mixtures (v/v; 10/90 to 90/10). x50 = 46.45 **%** for **Rh518bisCTet** and x50 = 39.93 for **Rh518bisCTet-BCN**, showing the propensity of the clicked product to be in the open-fluorescent form compared to the unclicked dyes; d) area under the curve of the emission intensity as a function of the dielectric constant of water/dioxane mixtures (v/v; 10/90 to 90/10).

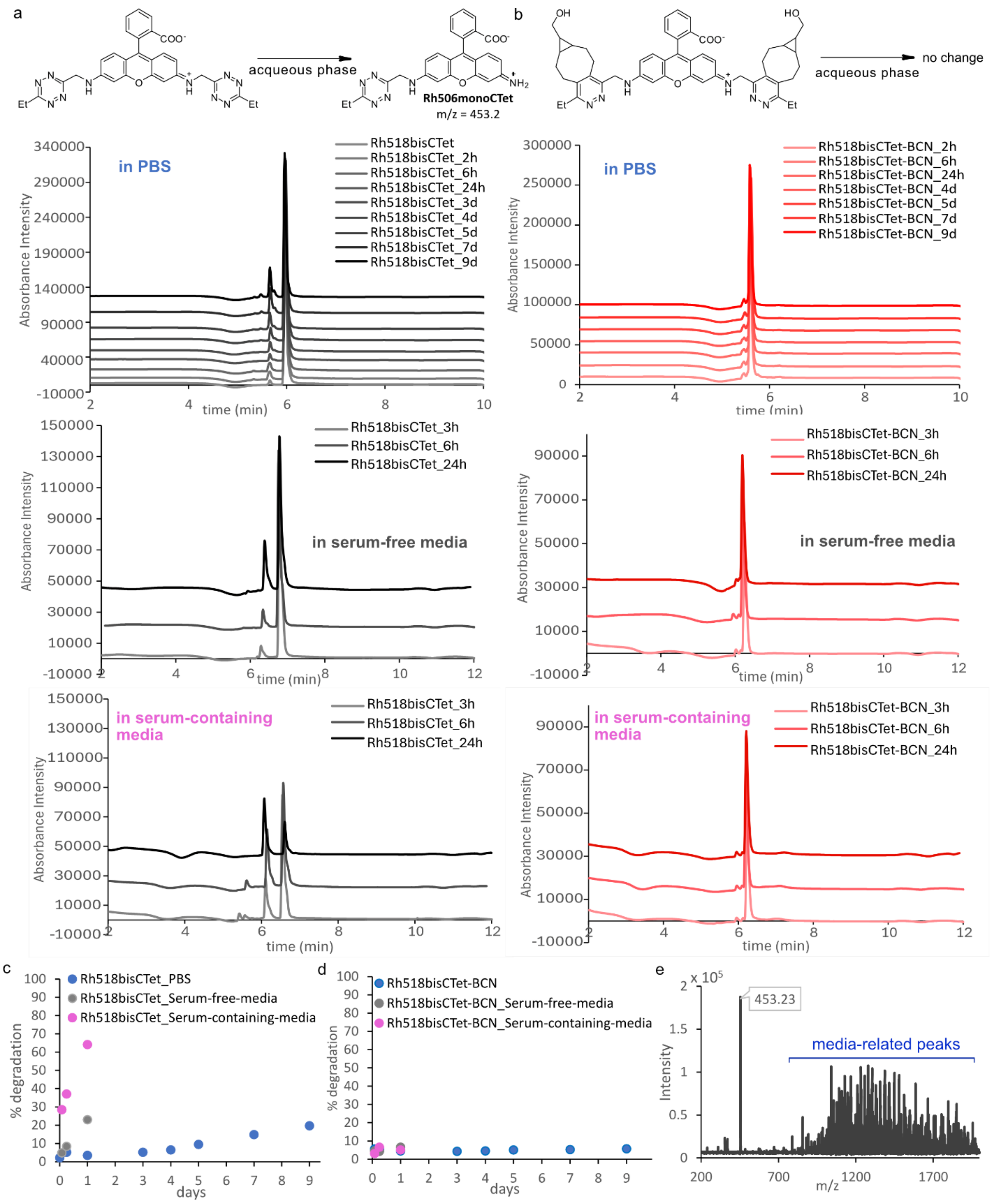


**Figure S12.** Stability of a) **Rh518bisCTet** and b) respective BCN-cycloadduct in PBS pH 7.2, in serum-free media (FluoroBrite DMEM) and in serum-containing media (DMEM+++) at RT at different time points measured by LC-MS. The percentage of decomposition of **Rh518bisCTet** was quantified by peak integration at 520 nm and nature of generated impurities analyzed by ESI. Percentage of impurities over time have been plotted in c) for **Rh518bisCTet** and in d) for the respective BCN-cycloadduct. e) Mass spectrum of the

peak at 6.2 min from **Rh518bisCTet** in serum-containing medium after 24 h incubation, showing a dominant ion at m/z 453.23 together with signals corresponding to components of the culture medium. LC-MS settings: eluent A (0.1% formic acid in water) and eluent B ($CH_3CN$) were used at a flow rate of 0.3 mL/min at 40°C, injection volume 10 μl. For the in PBS-conditions the following linear gradient elution was used: 2 min 10% B, 6 min 90%, 8 min 90% B, 8.50 min 10% B. For the in media-conditions the following linear gradient elution was used: 2 min 10% B, 5 min 45% B, 8 min 45% B, 9 min 90%, 10.45 min 90% B, 10.50 min 10% B.

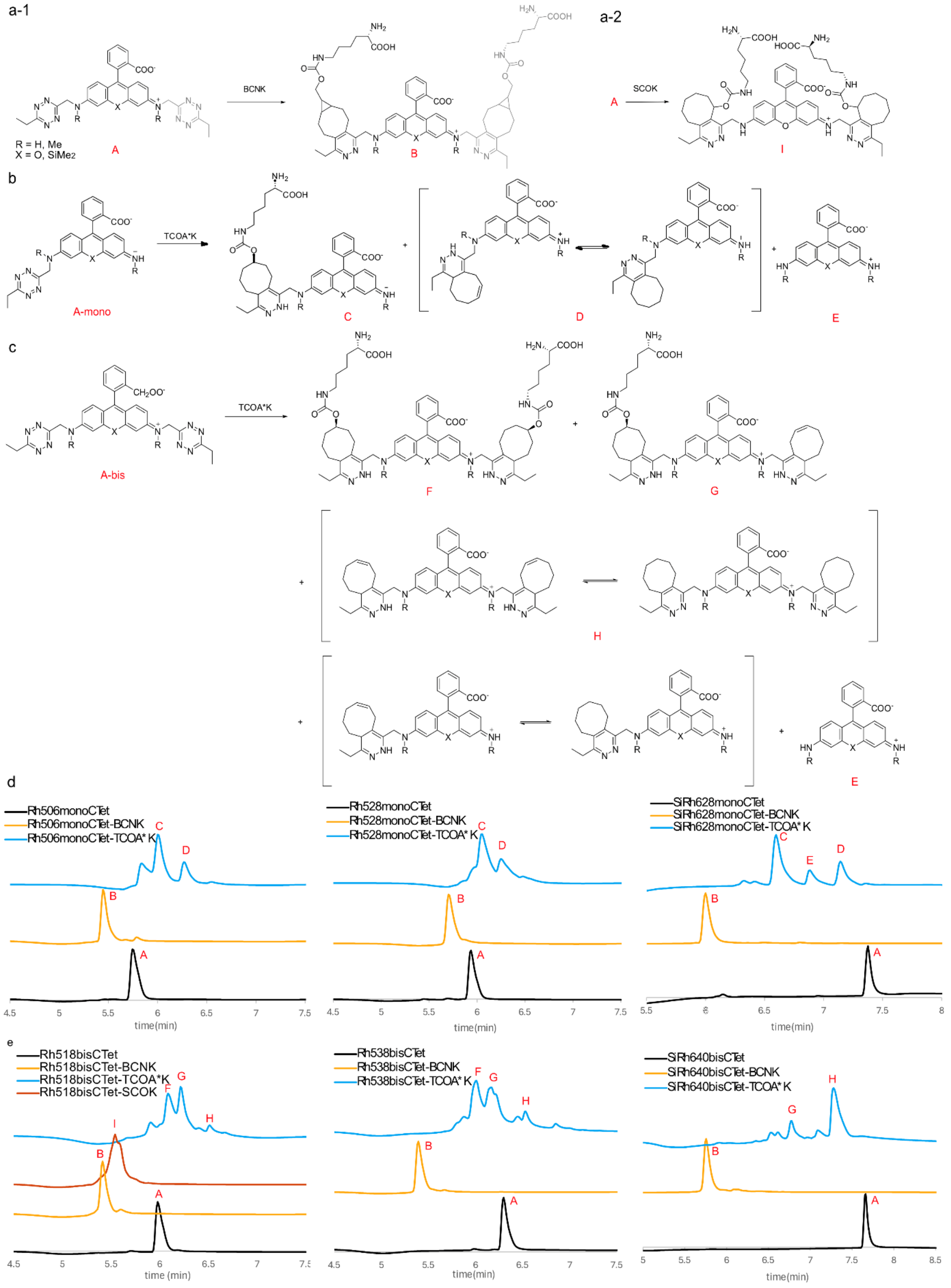


**Figure S13**. a-1) Schematic representation of the reaction between (Si)Rh-CTet dyes and BCNK, providing one single product; a-2) schematic representation of the reaction between **Rh518bisCTet** dyes and SCOK,

providing one single product; b) schematic representation of the reaction with (Si)Rh-monoCTet dyes with TCOA*K, providing the cycloadduct product and the corresponding release products; c) schematic representation of the reaction with (Si)Rh-bisCTet dyes with TCOA*K, providing the cycloadduct product and the corresponding release products. d-e) HPLC chromatograms with peak indicating the corresponding products for (d) mono-derivatives and (e) bis-derivatives. Absorbance peaks were registered at 510, 520, 530, 540, 630 and 280 nm for **Rh506monoCTet, Rh518bisCTet, Rh528monoCTet, Rh538bisCTet, SiRh628monoCTet** and **SiRh640bisCTet**, respectively. The click reaction with SCOK was performed exclusively with **Rh518bisCTet** because of its comparatively low reactivity and its structural similarity to BCNK, both containing a strained alkyne moiety. Complete reaction at room temperature was observed only after several days.

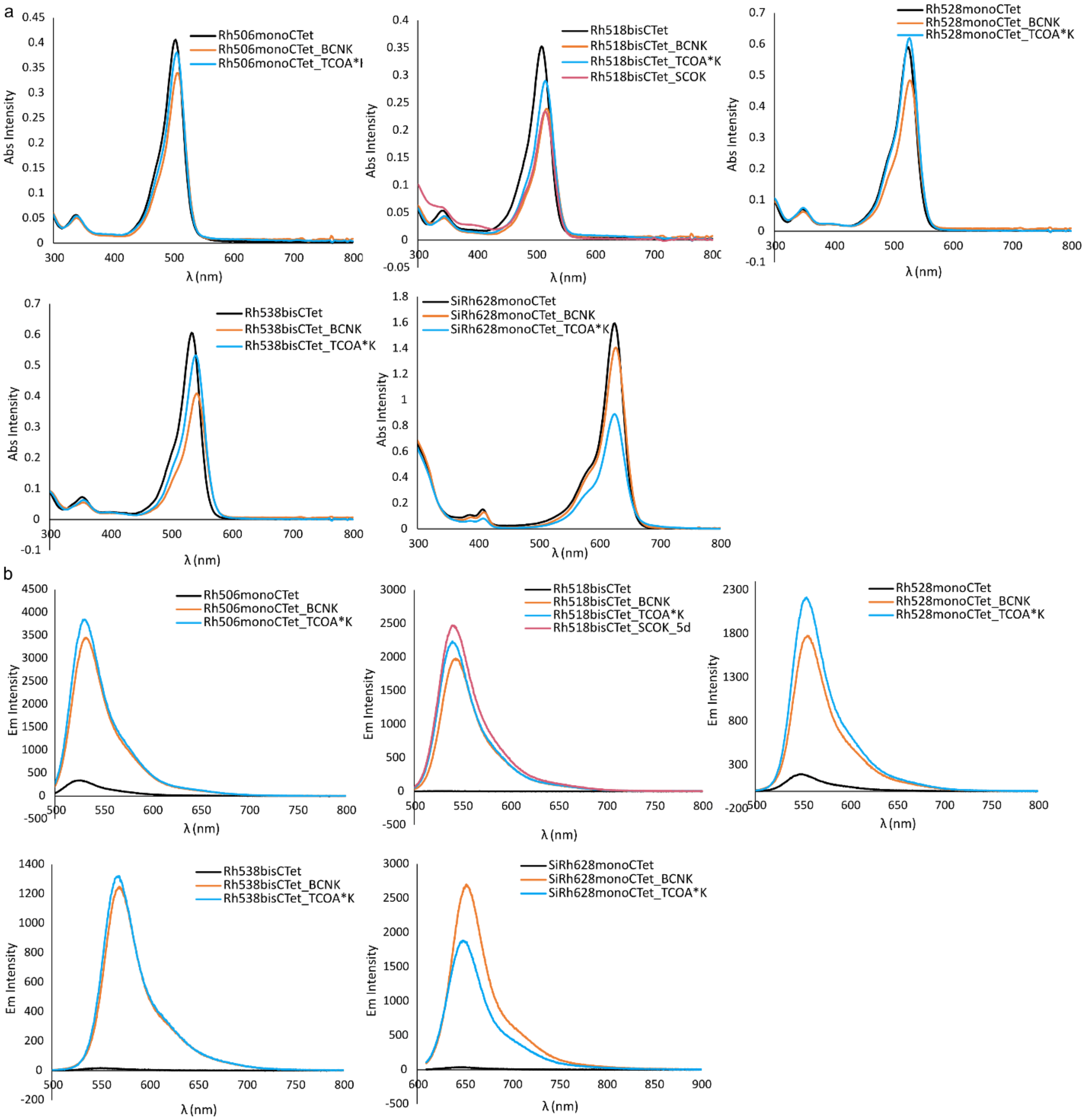


**Figure S14.** a) Absorbance and b) emission spectra of 8 µM solutions of (Si)Rh-CTet dyes in pH 7.2 PBS before and after the reaction in the presence of 12.5 equiv of BCNK (data acquired after 2 h) and TCOA*K (data acquired after 5 min). Each dye was prepared from a common 8 µM stock solution and then divided into three aliquots for data acquisition before click (sample 1), after click with BCNK (sample 2), and after click with TCOA*K (sample 3). Given the high brightness of **Rh506monoCTet**, a 6 µM final concentration was used. For the reaction between **Rh518bisCTet** and SCOK, an 8 µM solution was prepared and incubated at RT for up to 5 days.

### Intramolecular click reaction between Rh518bisCTet and bis-cyclooctynyl peptides, Pep1-8 and cyclooctynyl peptide, Pep9.

Peptides 1-9, purchased from piChem, were dissolved in anhydrous DMSO to create 30 µM stock solutions. On demand, these stock solutions were further diluted to 10 µM stock solutions. In a brown-glass HPLC vial, 1 µL of bis-cyclooctynyl peptide (conc. stock solution 10 mM) was diluted in 240 µL of PBS/MeCN 1:1 solution (MeCN is necessary to increase solubility of the peptide upon cyclization with the dye). Afterwards **Rh518bisCTet** was added (1 µL from 2 mM stock solution). Thus, the final concentration was 8.3 µM. The resulting mixtures were kept for 30 min at RT. Afterward, half the amount (120 µL) was analyzed via a spectrometer, while the remaining amount was analyzed via LC-MS. All fluorescence measurements were carried out in a standard cell quartz cuvette with 1 cm light path length and a minimum volume of 120 µL. Absorbance was detected between 300-800 nm, while emission between 500-800 nm (with excitation wavelength of 490 nm). PBS solutions with DMSO (0.5 µL) were used as blank corrections. Analytical LC-MS experiments of the peptide cyclization induced by click reaction were performed on a SHIMADZU LCMS-2020 system with a photodiode array (200-700 nm) and an ESI-MS detector. A linear gradient was employed using eluent A (0.1% formic acid in water) and eluent B ($CH_3CN$) under the following conditions: 2 min 10% B, 5 min 45% B, 8 min 45% B, 9 min 90%, 10.45 min 90% B, 10.50 min 10% B. The flow rate was set to 0.3 mL/min at 40°C with an injection volume of 10 µl.

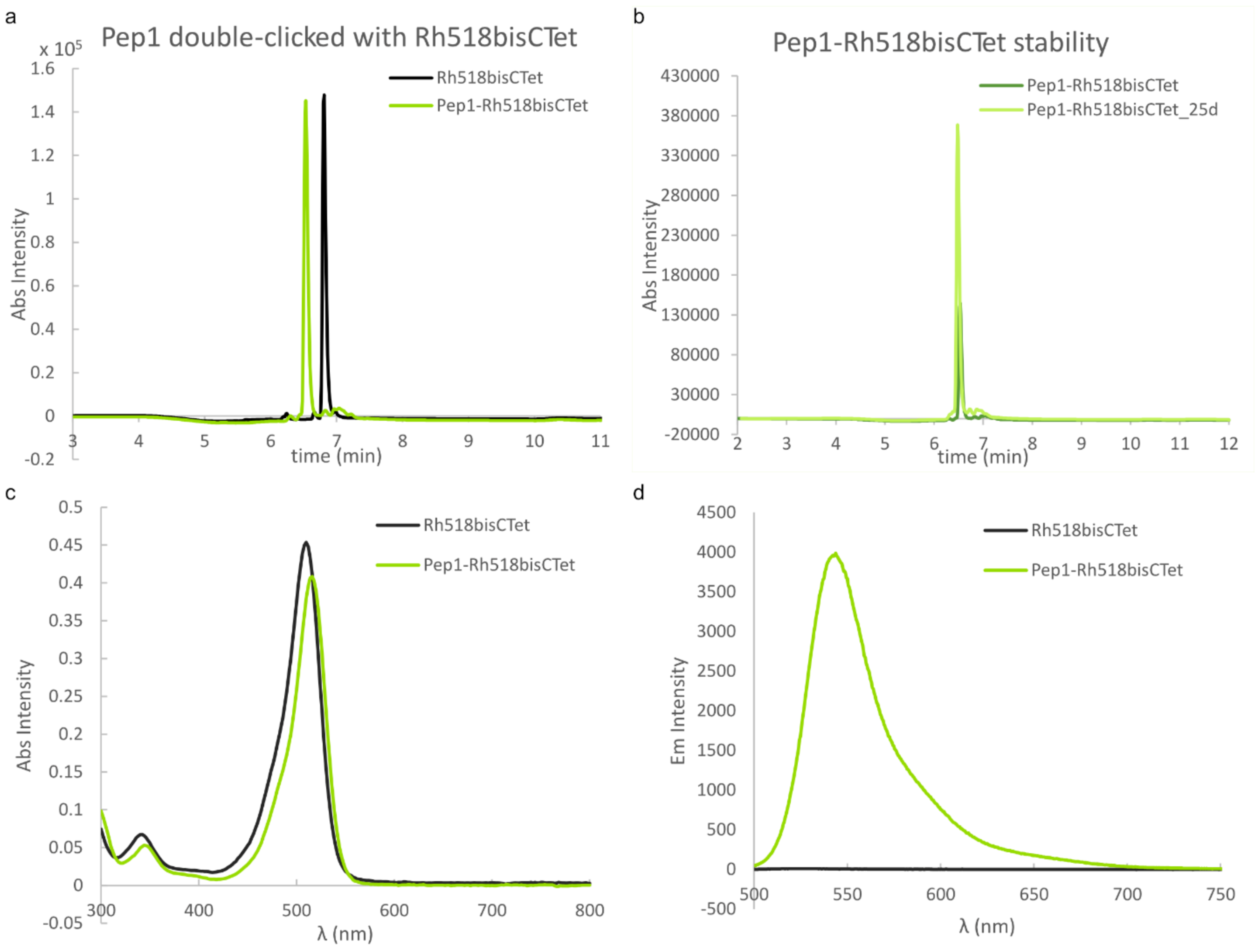


**Figure S15-1.** Peptide cyclization with **Rh518bisCTet**: Peptide1 AcK(BCN)**AEAADAEAA**K(BCN)-OH. a) HPLC chromatogram of **Rh518bisCTet** before and after click reaction with Pep1 at 520 nm. b) Stability of Pep1-**Rh518bisCTet** over 25 days in PBS/$CH_3CN$ mixture at ambient temperature, monitored via HPLC coupled with ESI-MS. Retention time of the samples after 25 days, labeled as Pep1-Rh518bisCTet_25d, had to be corrected from 6.40 to 6.48 min, an adjustment by 0.08 seconds (eight hundredths). Concentration of Pep1-Rh518bisCTet_25d was higher due to partial evaporation of the solvent. c) Absorbance and d) emission spectra of **Rh518bisCTet** before and after click reaction with Pep1.

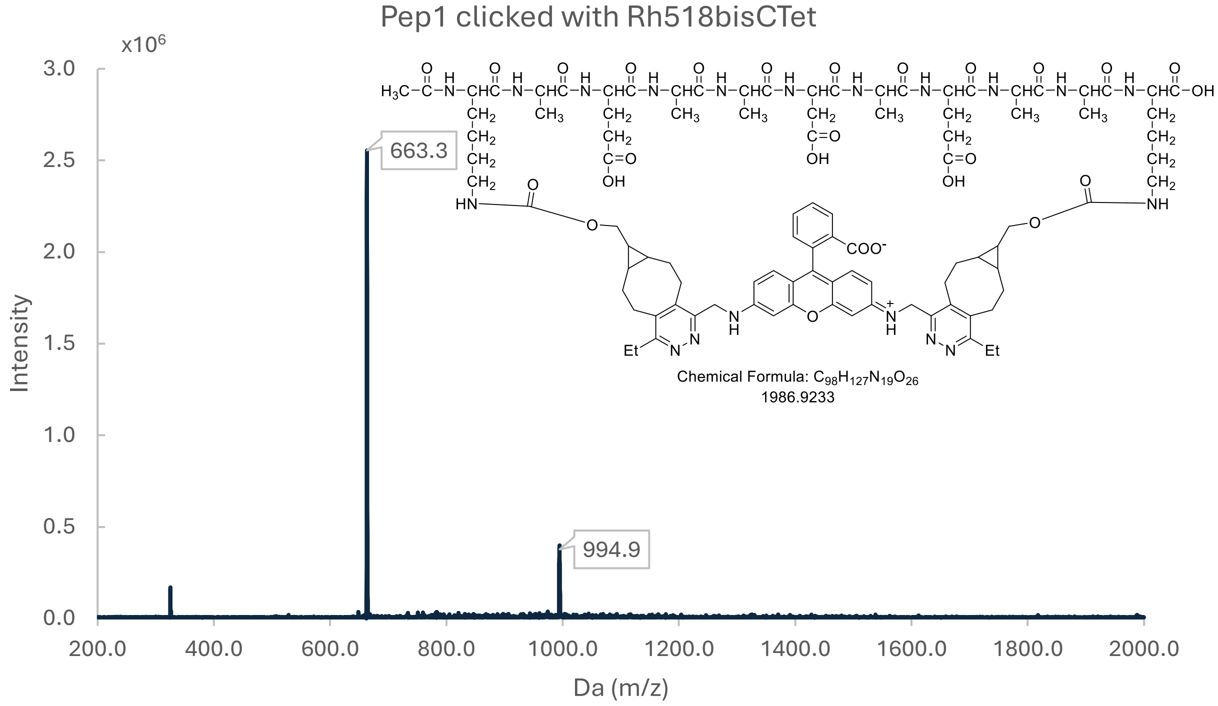


**Figure S15-2.** ESI$^+$ spectra of the Pep1-**Rh518bisCTet** adduct, as double and triple charged adduct.

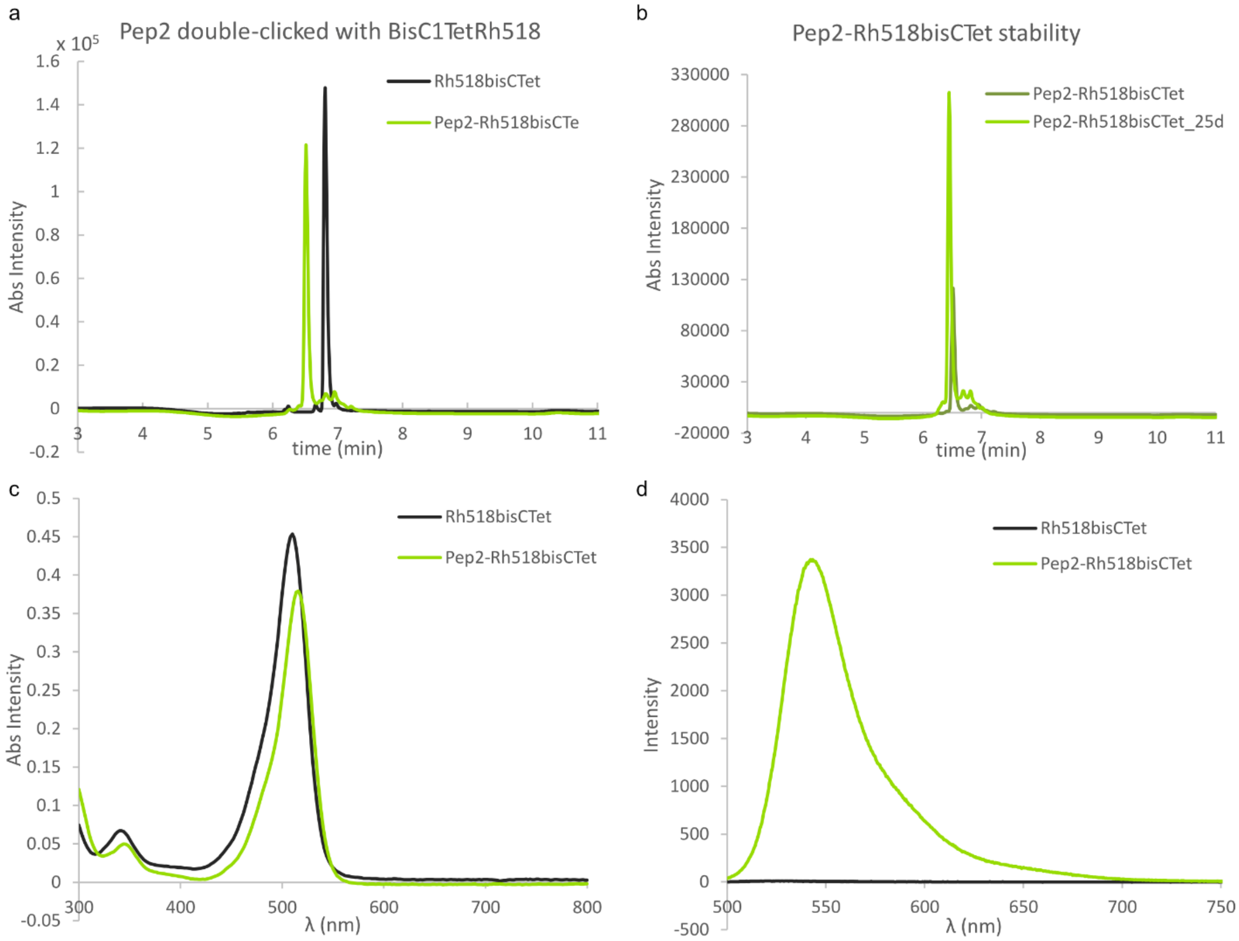


**Figure S16-1.** Peptide cyclization with **Rh518bisCTet**: Peptide2 AcK(BCN)**GSAAGSA**K(BCN)-OH. a) HPLC chromatogram of **Rh518bisCTet** before and after click reaction with Pep2 at 520 nm. b) Stability of Pep2-**Rh518bisCTet** over 25 days in PBS/$CH_3CN$ mixture at ambient temperature, monitored via HPLC coupled with ESI-MS. Retention time of the samples after 25 days, labeled as Pep2-Rh518bisCTet_25d, had to be corrected from 6.38 to 6.45 min, an adjustment by 0.07 seconds (seven hundredths). Concentration of Pep2-Rh518bisCTet_25d was higher due to partial evaporation of the solvent. c) Absorbance and d) emission spectra of **Rh518bisCTet** before and after click reaction with Pep2.

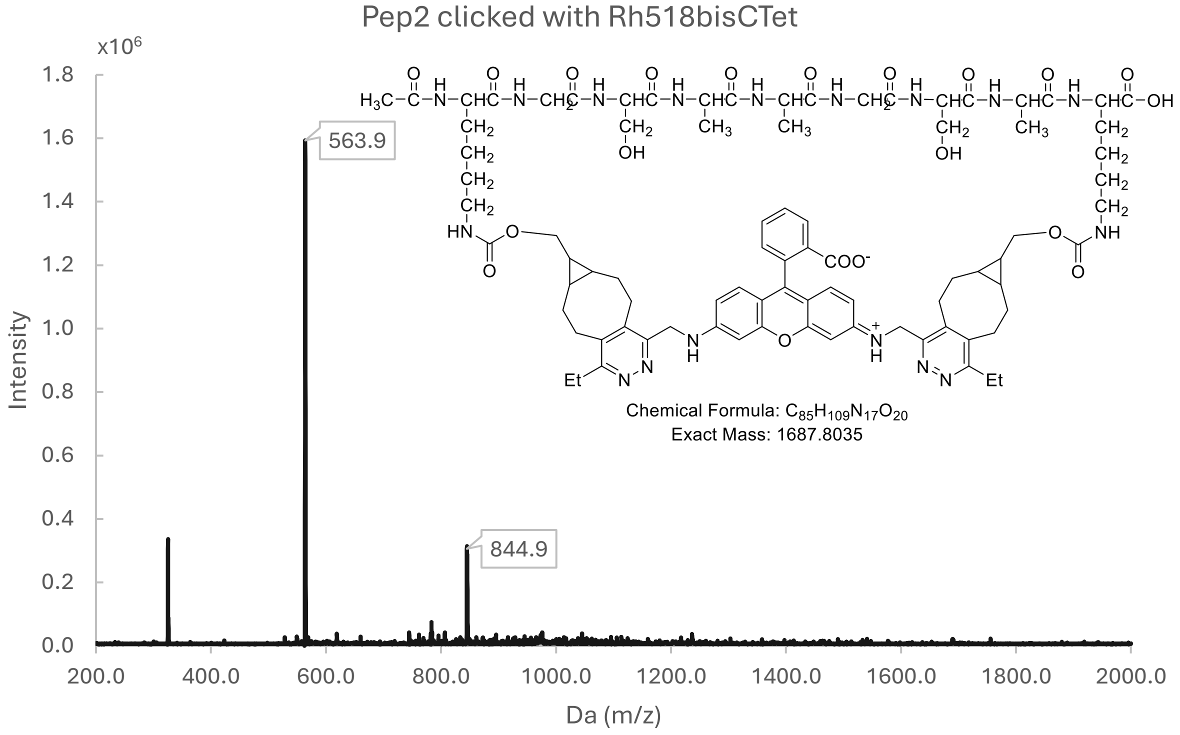


**Figure S16-2.** $ESI^+$ spectra of the Pep2-Rh518bisCTet adduct, as double and triple charged adduct.

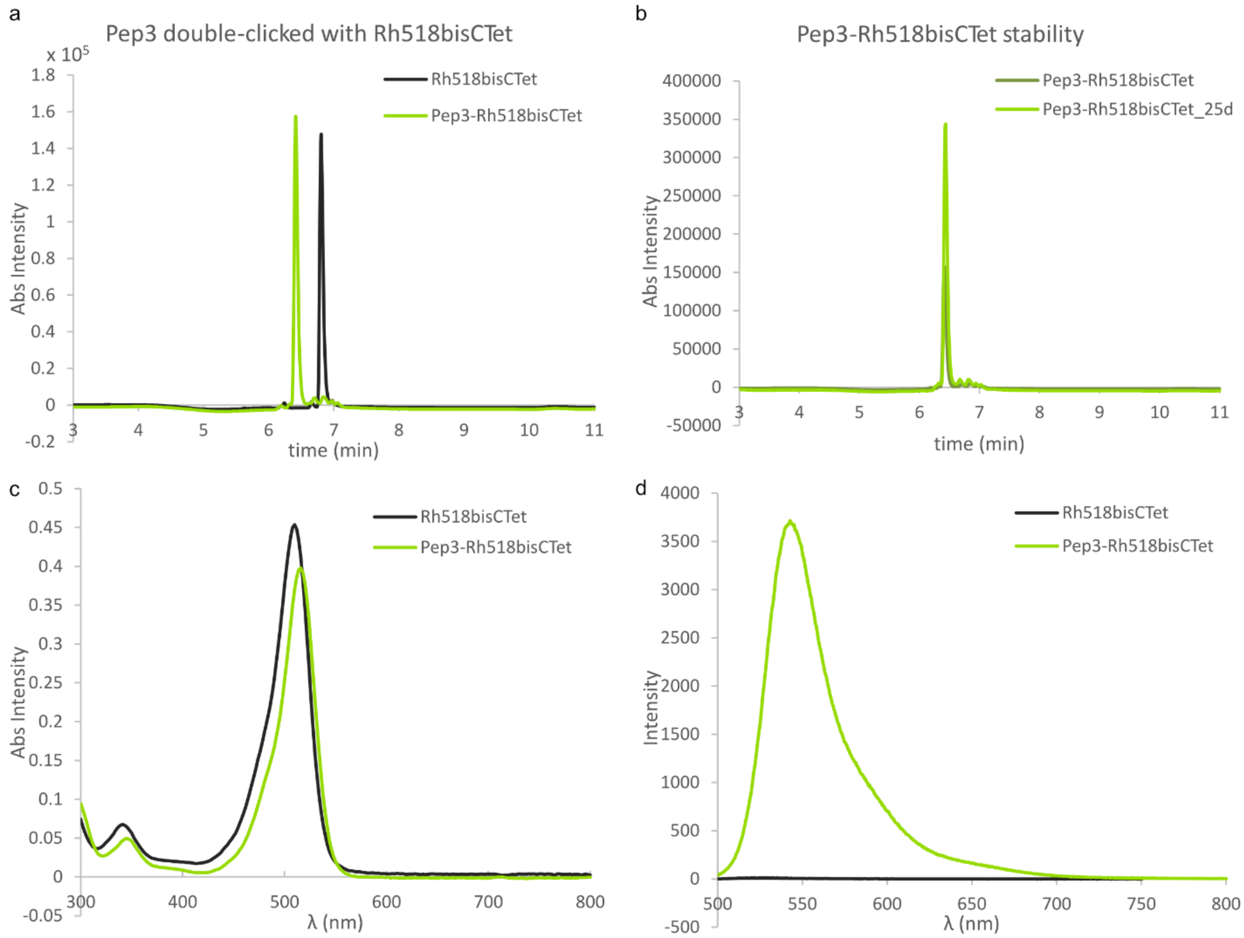


**Figure S17-1.** Peptide cyclization with **Rh518bisCTet**: Peptide3 AcK(BCN)**GSAAEAA**K(BCN)-OH. a) HPLC chromatogram of **Rh518bisCTet** before and after click reaction with Pep3 at 520 nm. b) Stability of Pep3-**Rh518bisCTet** over 25 days in PBS/$CH_3CN$ mixture at ambient temperature, monitored via HPLC coupled with ESI-MS. Retention time of the samples after 25 days, labeled as Pep3-Rh518bisCTet_25d, had to be corrected from 6.40 to 6.44 min, an adjustment by 0.04 seconds (four hundredths). Concentration of Pep3-Rh518bisCTet_25d was higher due to partial evaporation of the solvent. c) Absorbance and d) emission spectra of **Rh518bisCTet** before and after click reaction with Pep3.

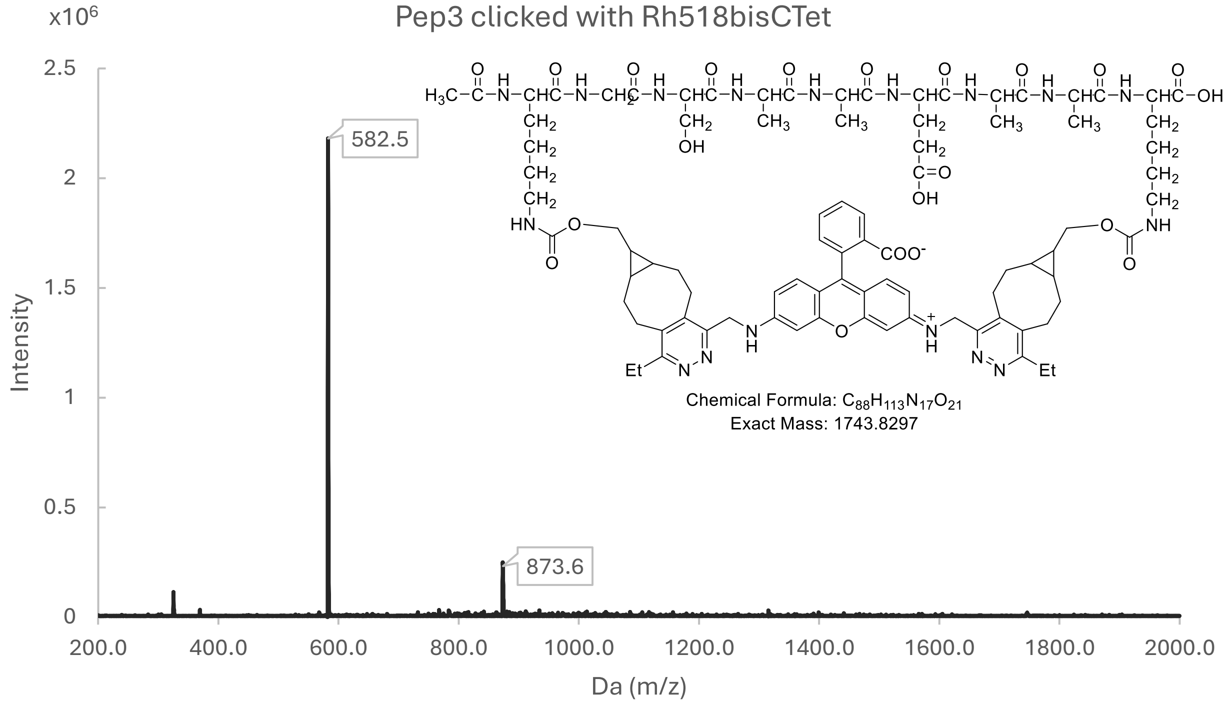


**Figure S17-2.** ESI+ spectra of the Pep3-**Rh518bisCTet** adduct, as double and triple charged adduct.

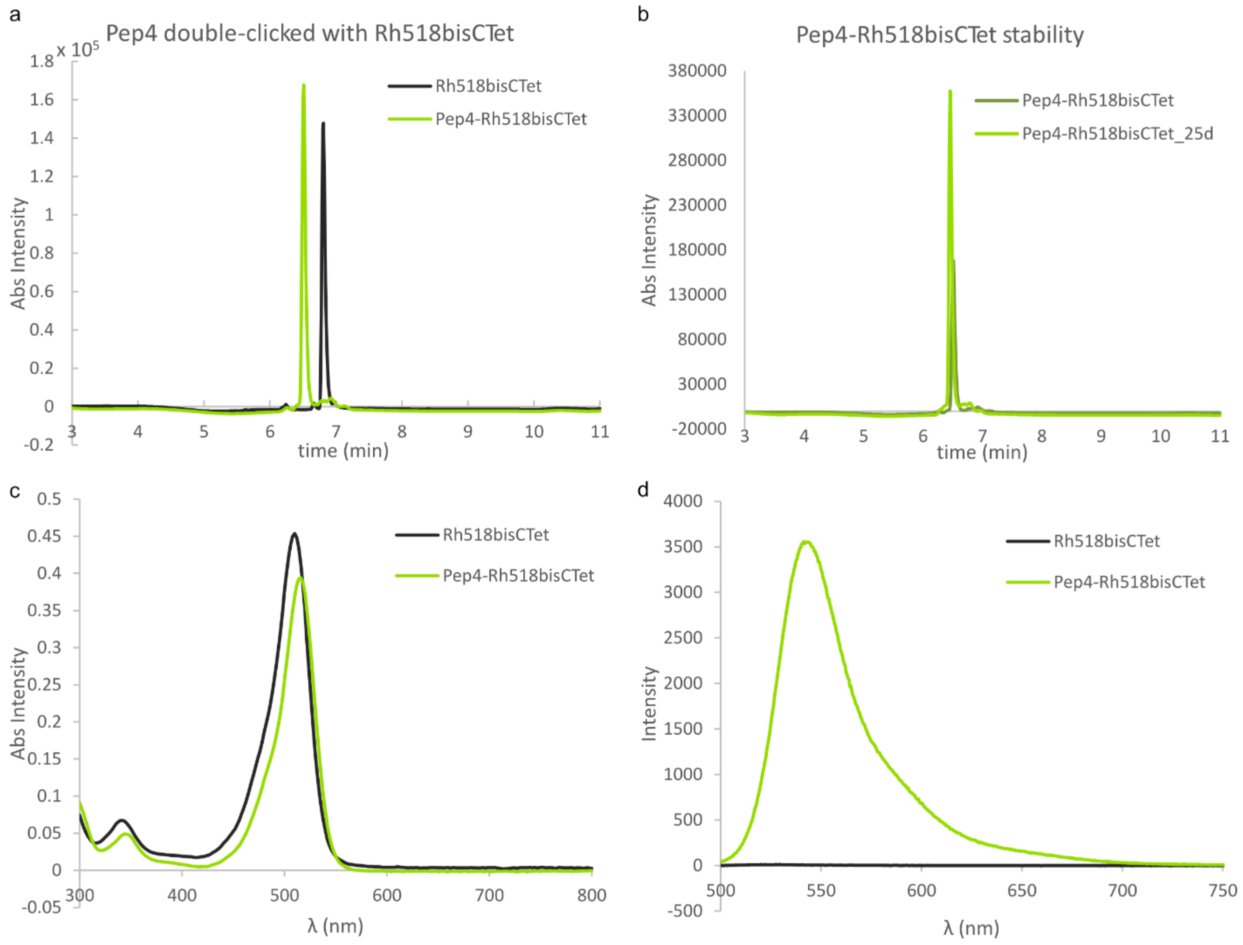


**Figure S18-1.** Peptide cyclization with **Rh518bisCTet**: Peptide4 AcK(BCN)**ADAAEADAAEA**K(BCN)-OH. a) HPLC chromatogram of **Rh518bisCTet** before and after click reaction with Pep4 at 520 nm. b) Stability of Pep4-**Rh518bisCTet** over 25 days in PBS/$CH_3CN$ mixture at ambient temperature, monitored via HPLC coupled with ESI-MS. Retention time of the samples after 25 days, labeled as Pep4-Rh518bisCTet_25d, had to be corrected from 6.35 to 6.46 min, an adjustment by 0.11 seconds (eleven hundredths). Concentration of Pep4-Rh518bisCTet_25d was higher due to partial evaporation of the solvent. c) Absorbance and d) emission spectra of **Rh518bisCTet** before and after click reaction with Pep4.

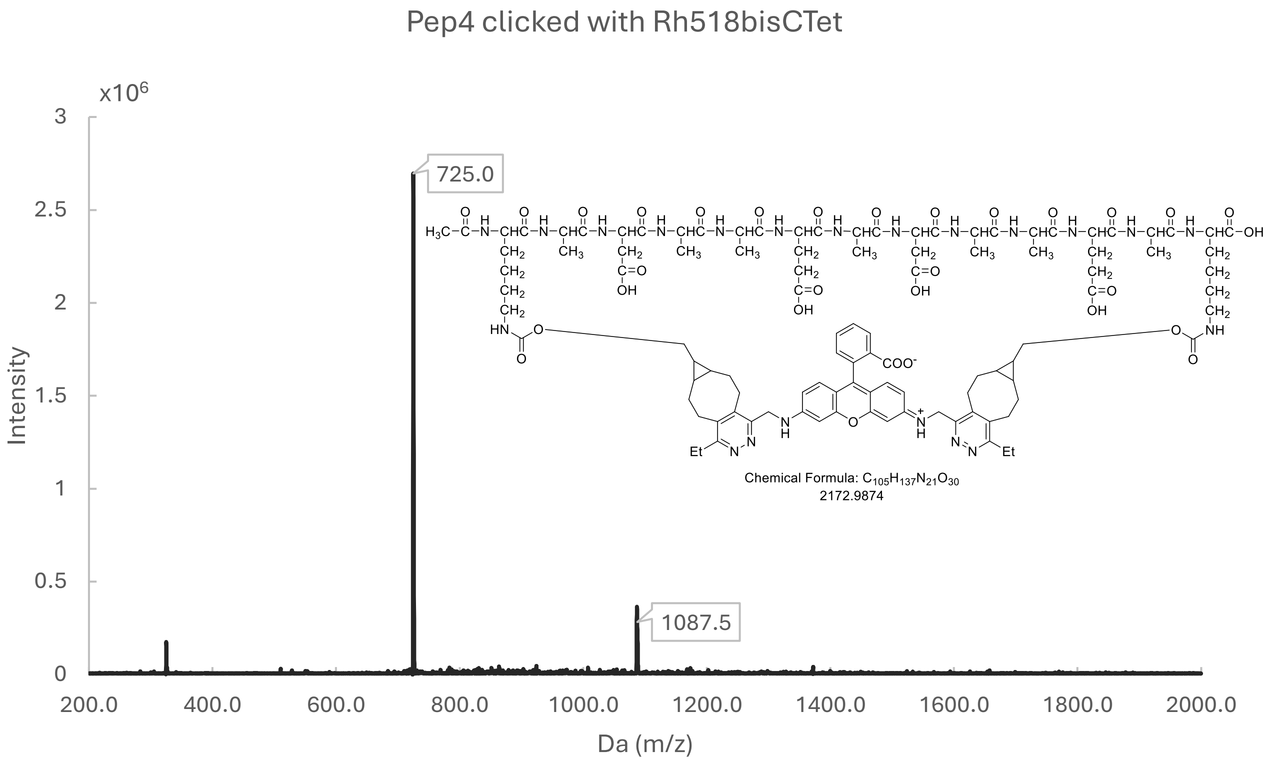


**Figure S18-2.** ESI$^+$ spectra of the Pep4-**Rh518bisCTet** adduct, as double and triple charged adduct.

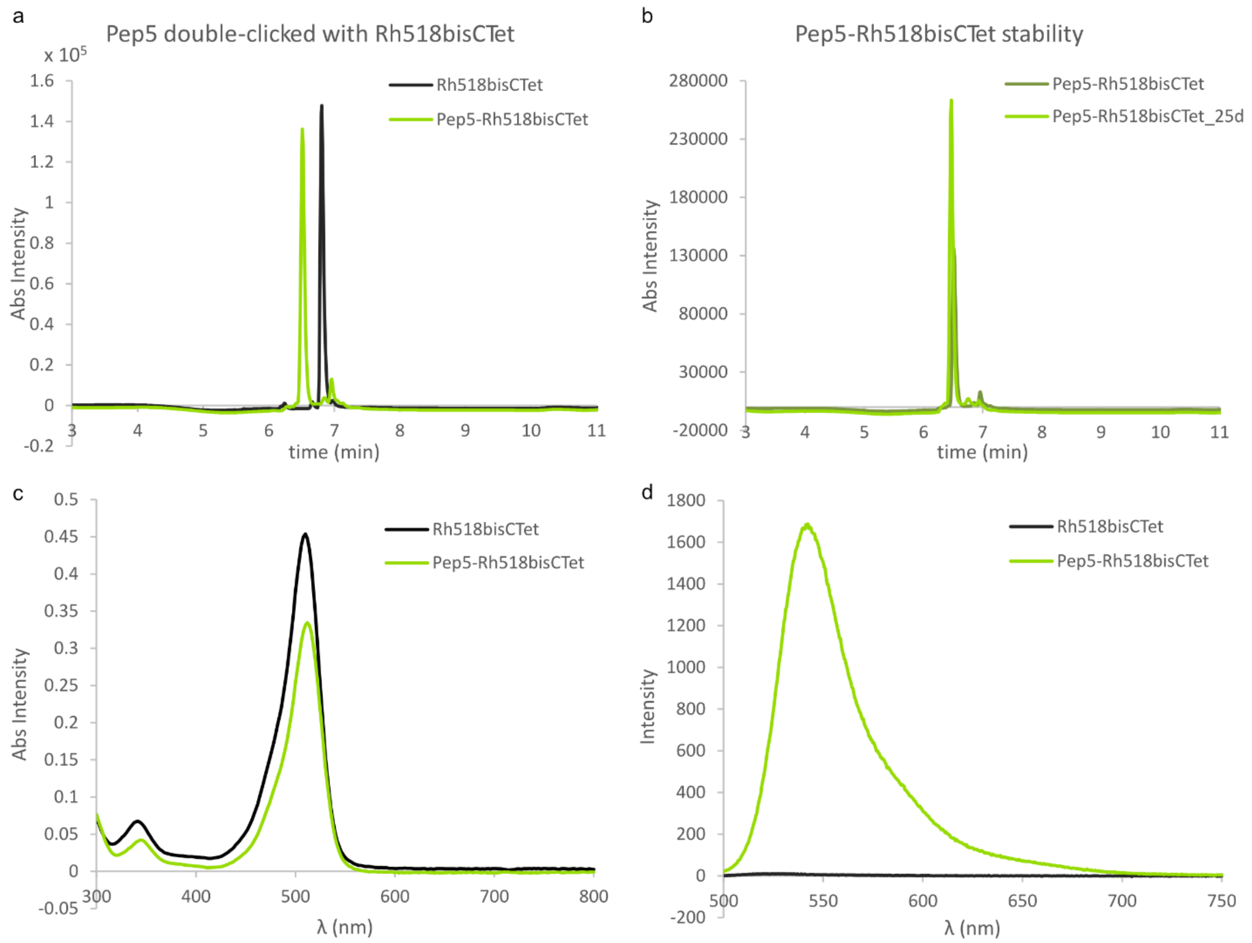


**Figure S19-1.** Peptide cyclization with **Rh518bisCTet**: Peptide5 AcK(BCN)**AAEA**K(BCN)-OH. a) HPLC chromatogram of **Rh518bisCTet** before and after click reaction with Pep5 at 520 nm. b) Stability of Pep5-**Rh518bisCTet** over 25 days in PBS/$CH_3CN$ mixture at ambient temperature, monitored via HPLC coupled with ESI-MS. Retention time of the samples after 25 days, labeled as Pep5-Rh518bisCTet_25d, had to be corrected from 6.40 to 6.47 min, an adjustment by 0.07 seconds (seven hundredths). Concentration of Pep5-Rh518bisCTet_25d was higher due to partial evaporation of the solvent. c) Absorbance and d) emission spectra of **Rh518bisCTet** before and after click reaction with Pep5.

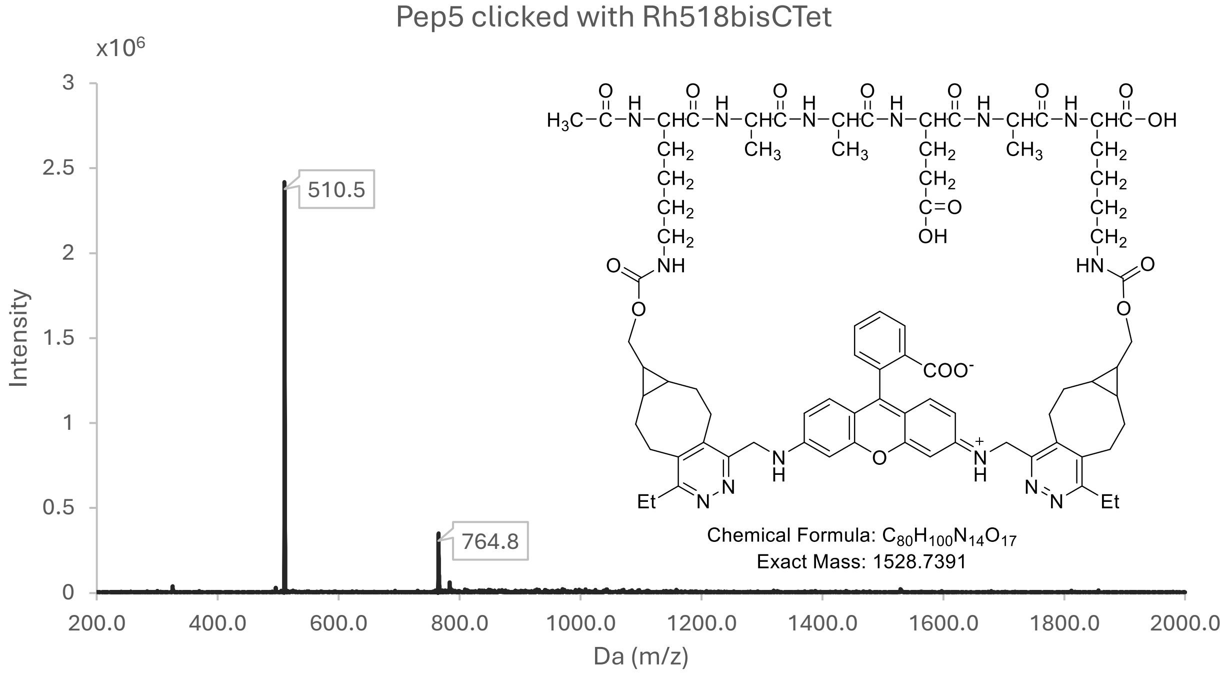


**Figure S19-2.** ESI$^+$ spectra of the Pep5-**Rh518bisCTet** adduct, as double and triple charged adduct.

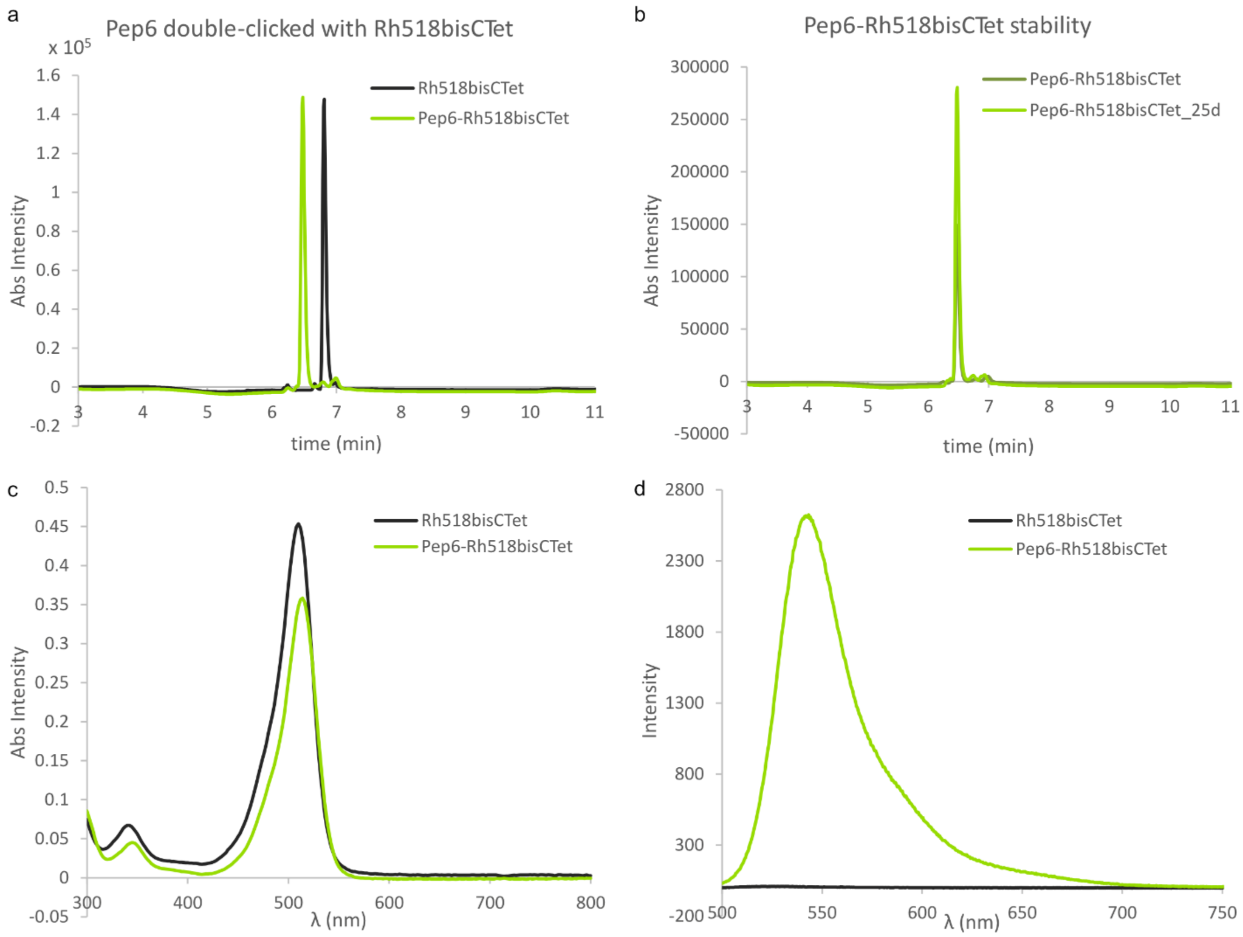


**Figure S20-1.** Peptide cyclization with **Rh518bisCTet**: Peptide6 AcK(BCN)**ADAAEA**K(BCN)-OH. a) HPLC chromatogram of **Rh518bisCTet** before and after click reaction with Pep6 at 520 nm. b) Stability of Pep6-**Rh518bisCTet** over 25 days in PBS/$CH_3CN$ mixture at ambient temperature, monitored via HPLC coupled with ESI-MS. Retention time of the samples after 25 days, labeled as Pep6-Rh518bisCTet_25d, had to be corrected from 6.41 to 6.48 min, an adjustment by 0.07 seconds (seven hundredths). Concentration of Pep6-Rh518bisCTet_25d was higher due to partial evaporation of the solvent. c) Absorbance and d) emission spectra of **Rh518bisCTet** before and after click reaction with Pep6.

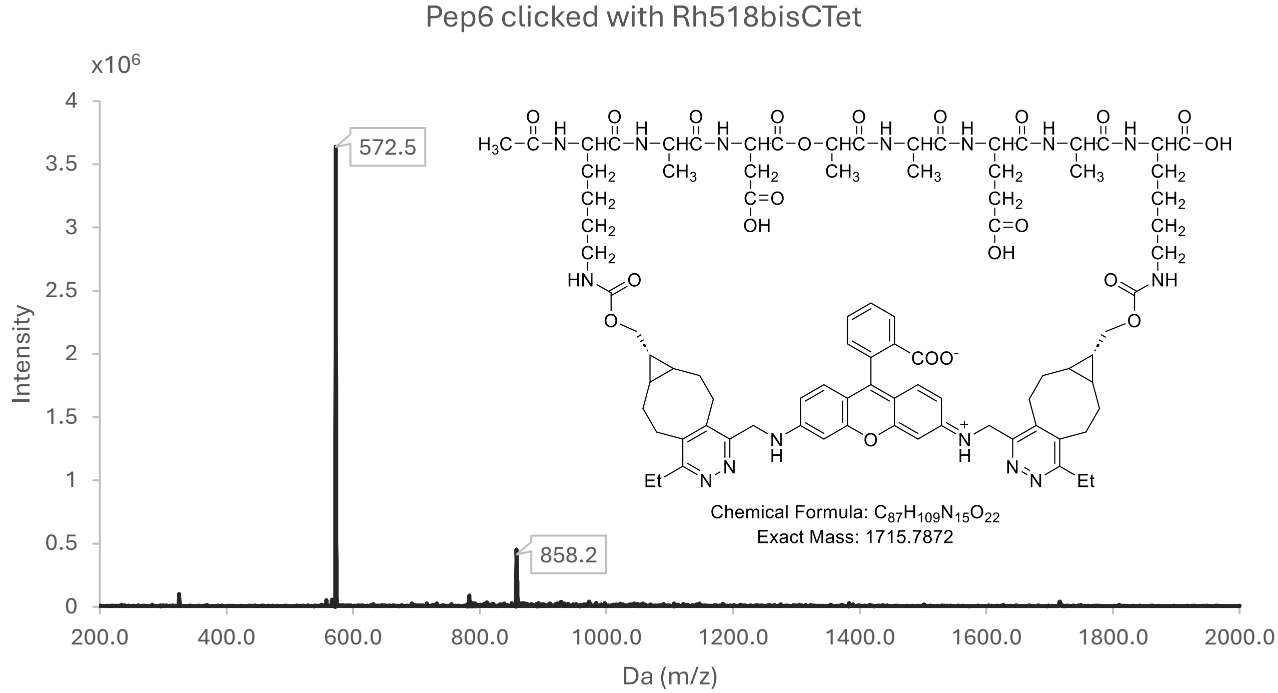


**Figure S20-2.** ESI+ spectra of the Rh518bisCTet-Pep6 adduct, as double and triple charged adduct.

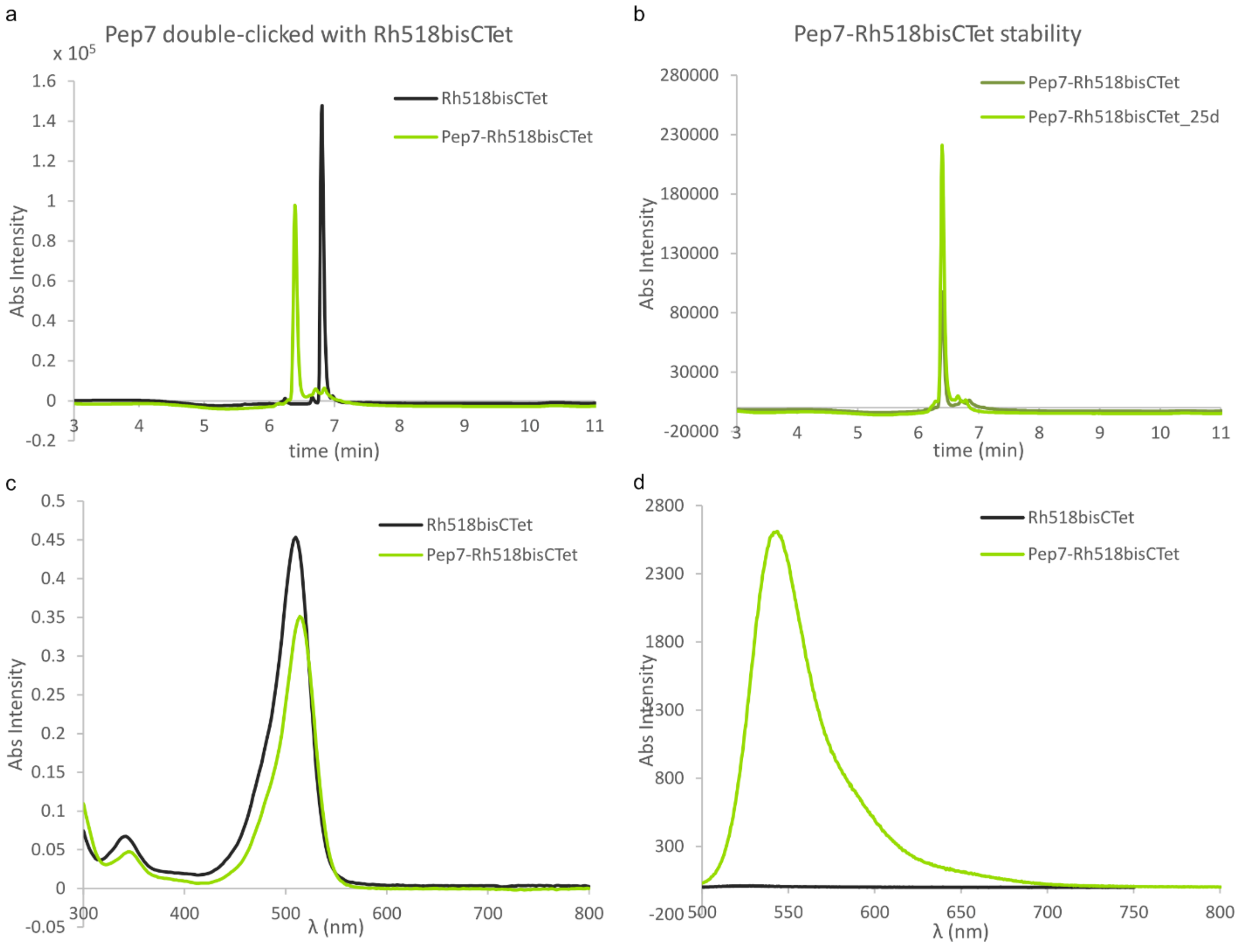


**Figure S21-1.** Peptide cyclization with **Rh518bisCTet**: Peptide7 AcK(BCN)**GGSG**K(BCN)-OH. a) HPLC chromatogram of **Rh518bisCTet** before and after click reaction with Pep7 at 520 nm. b) Stability of Pep7-**Rh518bisCTet** over 25 days in PBS/$CH_3CN$ mixture at ambient temperature, monitored via HPLC coupled with ESI-MS. Retention time of the samples after 25 days, labeled as Pep7-Rh518bisCTet_25d, had to be corrected from 6.32 to 6.39 min, an adjustment by 0.07 seconds (seven hundredths). Concentration of Pep7-Rh518bisCTet_25d was higher due to partial evaporation of the solvent. c) Absorbance and d) emission spectra of **Rh518bisCTet** before and after click reaction with Pep7.

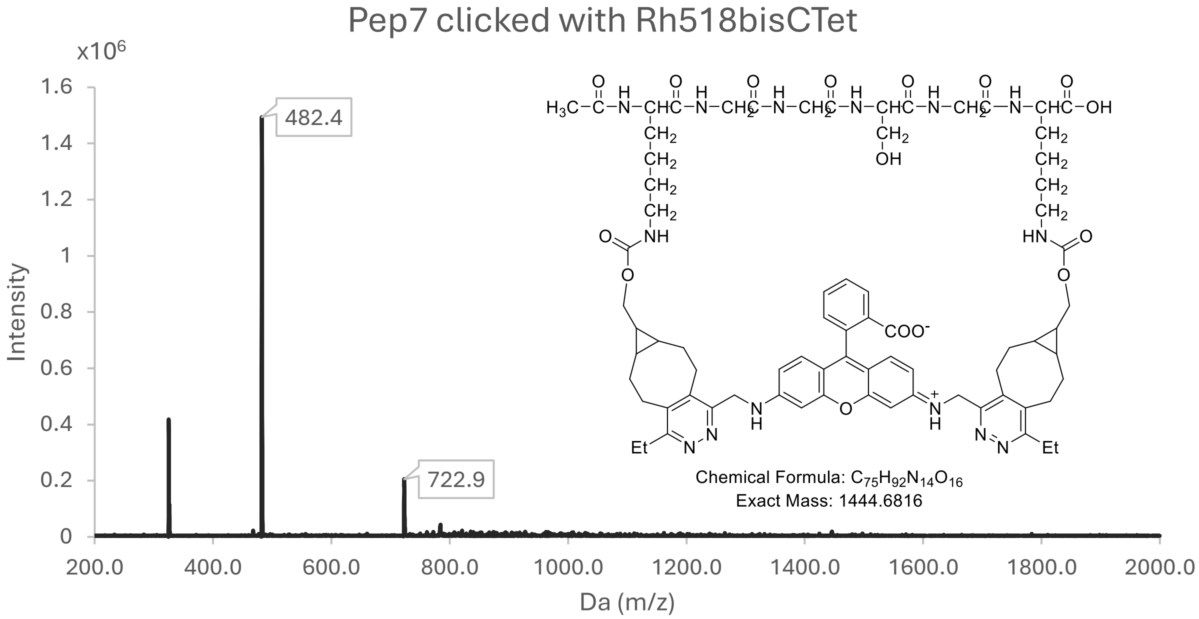


**Figure S21-2.** ESI+ spectra of the Pep7-Rh518bisCTet adduct, as double and triple charged adduct.

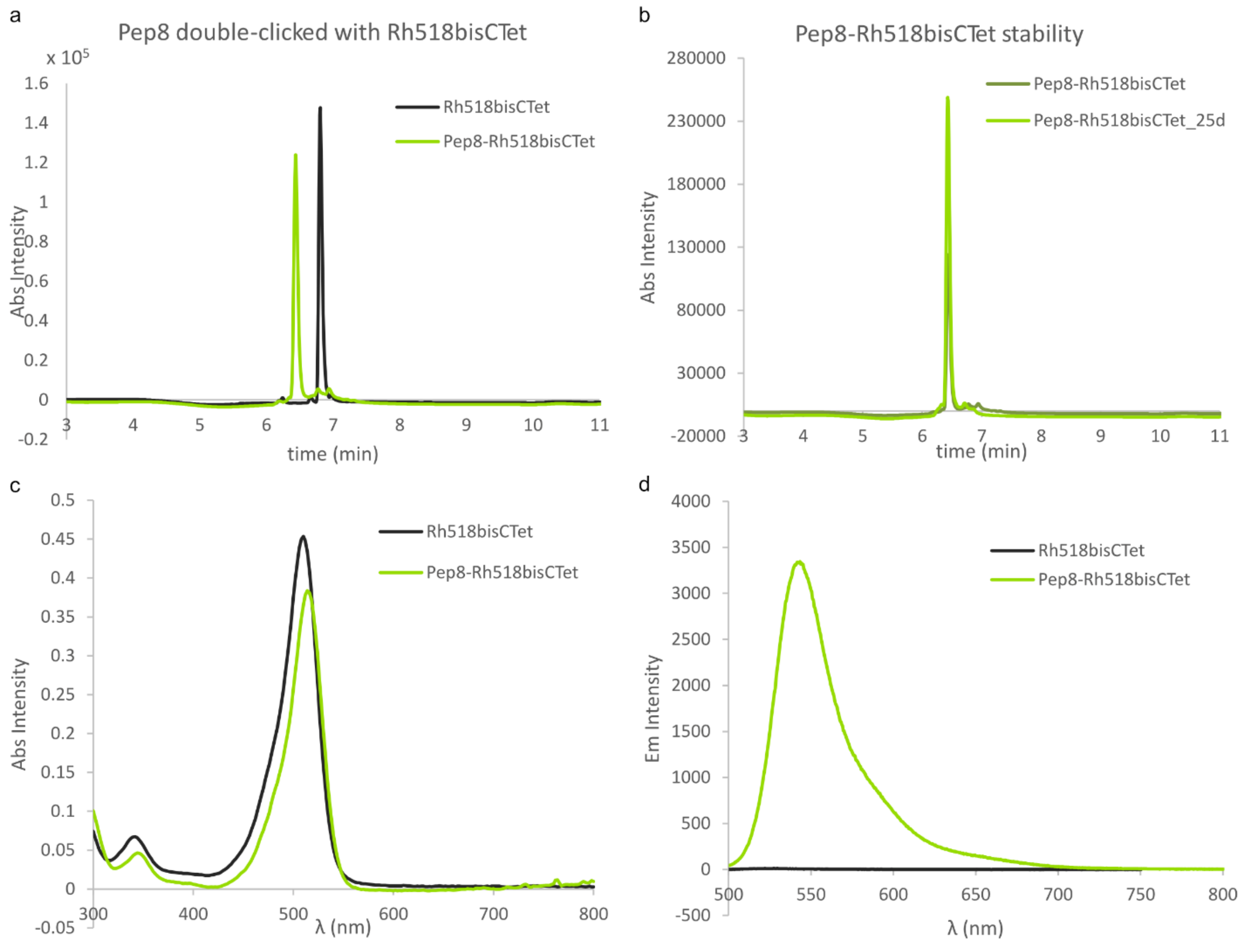


**Figure S22-1.** Peptide cyclization with **Rh518bisCTet**: Peptide8 AcK(BCN)**GSG**K(BCN)-OH. a) HPLC chromatogram of **Rh518bisCTet** before and after click reaction with Pep8 at 520 nm. b) Stability of Pep8-**Rh518bisCTet** over 25 days in PBS/$CH_3CN$ mixture at ambient temperature, monitored via HPLC coupled with ESI-MS. Retention time of the samples after 25 days, labeled as Pep8-Rh518bisCTet_25d, had to be corrected from 6.36 to 6.43 min, an adjustment by 0.07 seconds (seven hundredths). Concentration of Pep8-Rh518bisCTet_25d was higher due to partial evaporation of the solvent. c) Absorbance and d) emission spectra of **Rh518bisCTet** before and after click reaction with Pep8.

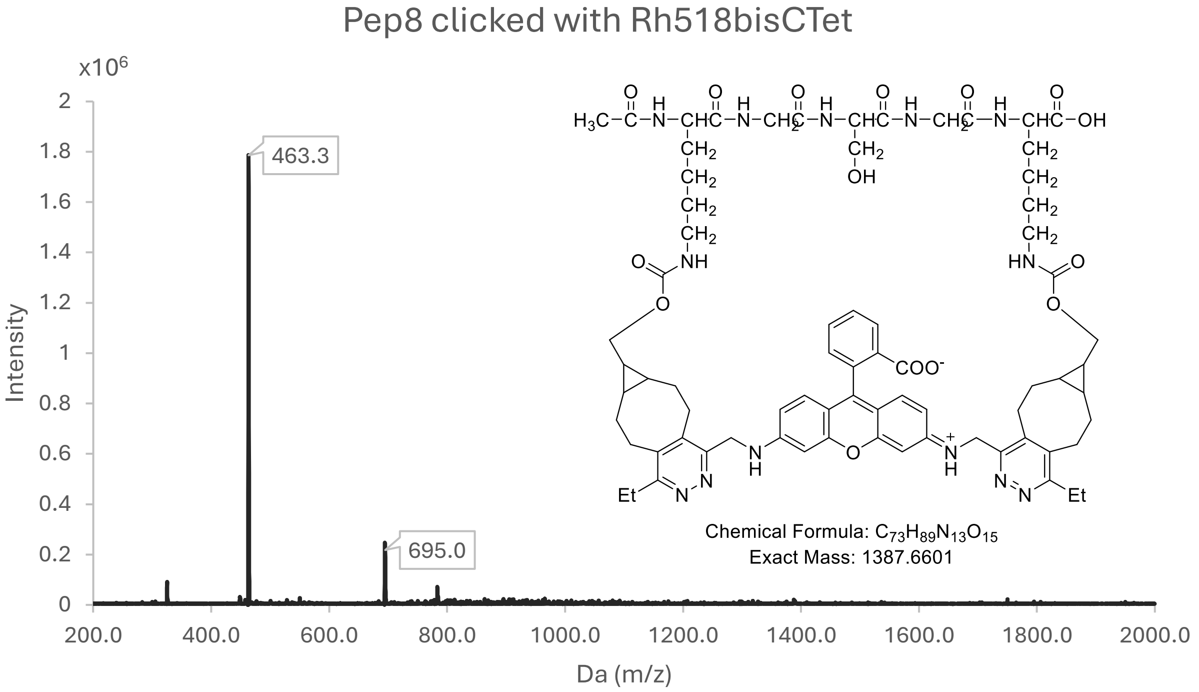


**Figure S22-2.** ESI$^+$ spectra of the Pep8-**Rh518bisCTet** adduct, as double and triple charged adduct.

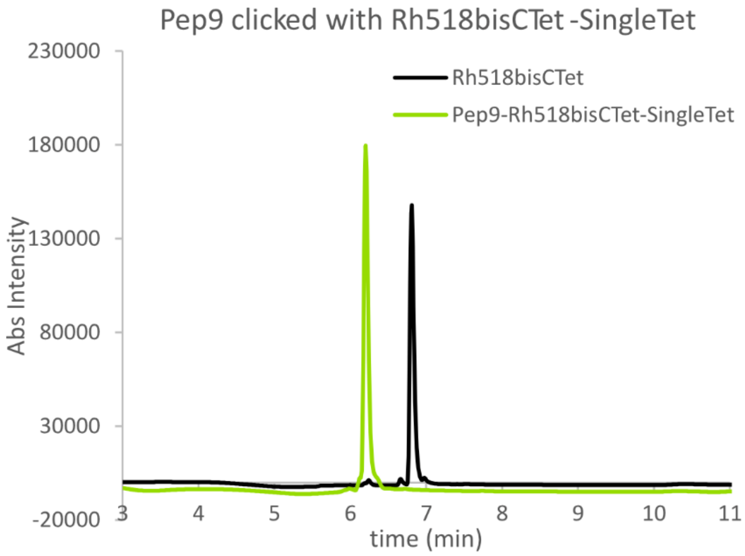


**Figure S23-1.** Peptide cyclization with Rh518bisCTet: Peptide9 AcK(Ac)**AEAADAEAA**K(BCN)-OH. a) HPLC chromatogram of **Rh518bisCTet-SingleTet** after click reaction with Pep9 at 520 nm.

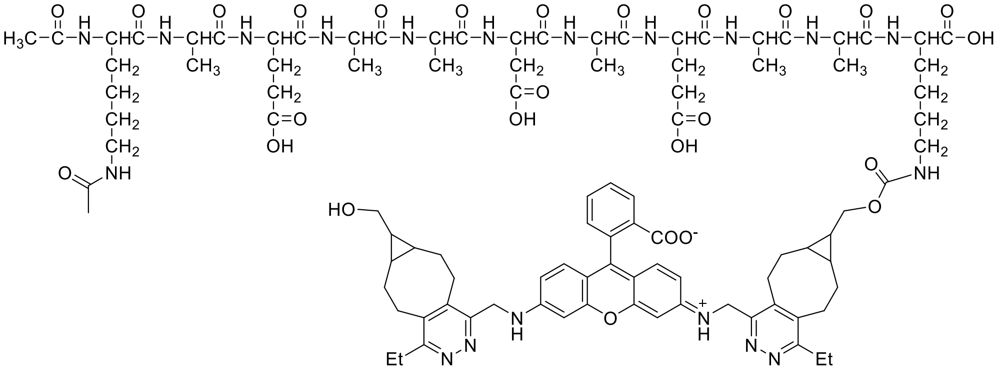

Chemical Formula: $C_{99}H_{131}N_{19}O_{26}$
2002.9546 g/mol

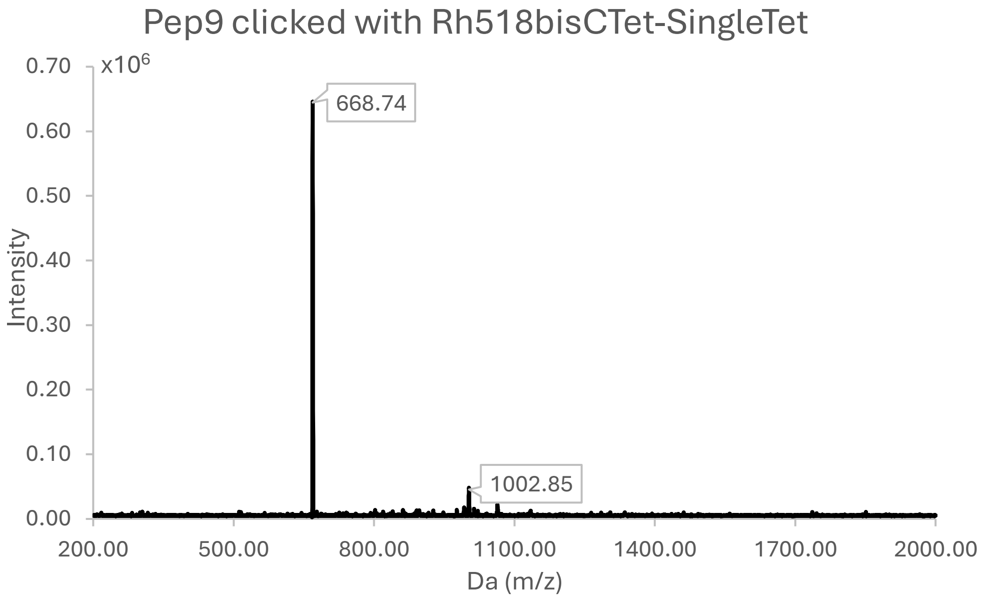


**Figure S23-2.** $ESI^+$ spectra of the Pep9-**Rh518bisCTet-SingleTet** adduct, as single and double charged adduct.

## Kinetics of click reaction

For the determination of the second order rate constant, time-dependent fluorescence measurements were performed. To this end, stock solutions of **Rh518bisCTet** (2 mM in DMSO) *endo*-BCN-lysine in DMSO (50 mM), TCO*A-lysine in DMSO (50 mM) and peptides, Pep1-4, in DMSO (30 mM) were used. The reactions were performed in PBS at 22 °C. After measuring the starting fluorescence intensity value of a 2.5 µM dye solution in 200 µl PBS, TCOAK was added (5, 10, 20, 40, 50, 60 equiv vs total tetrazine reactive sites, respectively). The mixture was immediately mixed thoroughly, and subsequently the fluorescence intensity was read out every 60 s, for 10 minutes. Similarly, for BCNK, to a 2.5 µM dye solution, *endo*-BCNK was added (60, 120, 180, 240, 360 equiv vs total tetrazine reactive sites, respectively). The mixture was immediately mixed thoroughly, and subsequently the fluorescence intensity was read out every 180 s for 60 minutes. For the peptides, to a 3.3 µM dye solution in 120 µl PBS, each peptide was added (5, 10, 20, 40 equiv, respectively). The mixture was immediately mixed thoroughly, and subsequently the fluorescence intensity was read out every 60 s for 20 minutes. For each peptide concentration, triplicate measurements were performed.

Kinetic measurements were performed under conditions in which reagent BCNK and TCOAK were present in large excess relative to the reacting substrate, such that its concentration remained effectively constant over the course of the reaction. Under these conditions, the reaction rate can be approximated by a pseudo-first-order rate law with respect to the substrate. Time-dependent fluorescence traces were therefore analyzed using single-exponential functions to extract observed rate constants ($k_{\mathrm{obs}}$).

The individual curves were fitted using the equation:

$$y = y0 + A(1 - e^{kobsx})$$

where $y$ is the measured signal (fluorescence intensity area), $x$ is the reaction time, $y_0$ is the initial signal at $x$ = 0, $A$ is the amplitude of the signal change, and $k_{\mathrm{obs}}$ is the observed first-order rate constant. The extracted mean $k_{\mathrm{obs}}$ (corresponding to pseudo-first order rate constants) were plotted against dienophile concentrations (BCNK, TCOAK or peptides) and the second order rate constant *k* was obtained as the slope from a linear fit. The validity of this approximation was independently confirmed by the linear dependence of $k_{\mathrm{obs}}$ on the initial concentration of BCNK and TCOAK over the range investigated, with near-zero intercepts, consistent with a bimolecular rate-limiting step involving BCNK (Figure S21a) and TCOAK (Figure S21b). Additionally, for reactions involving BCNK and TCOAK, which proceed through a three-component process, the effective concentrations were adjusted on an equivalent reactive-handle basis to enable meaningful comparison with mono- and bis-functional substrates. This correction prevents systematic underestimation of the apparent rate constants and allows direct comparison of kinetic parameters across different substrates and reaction formats while minimizing biases associated with higher-order reaction kinetics.

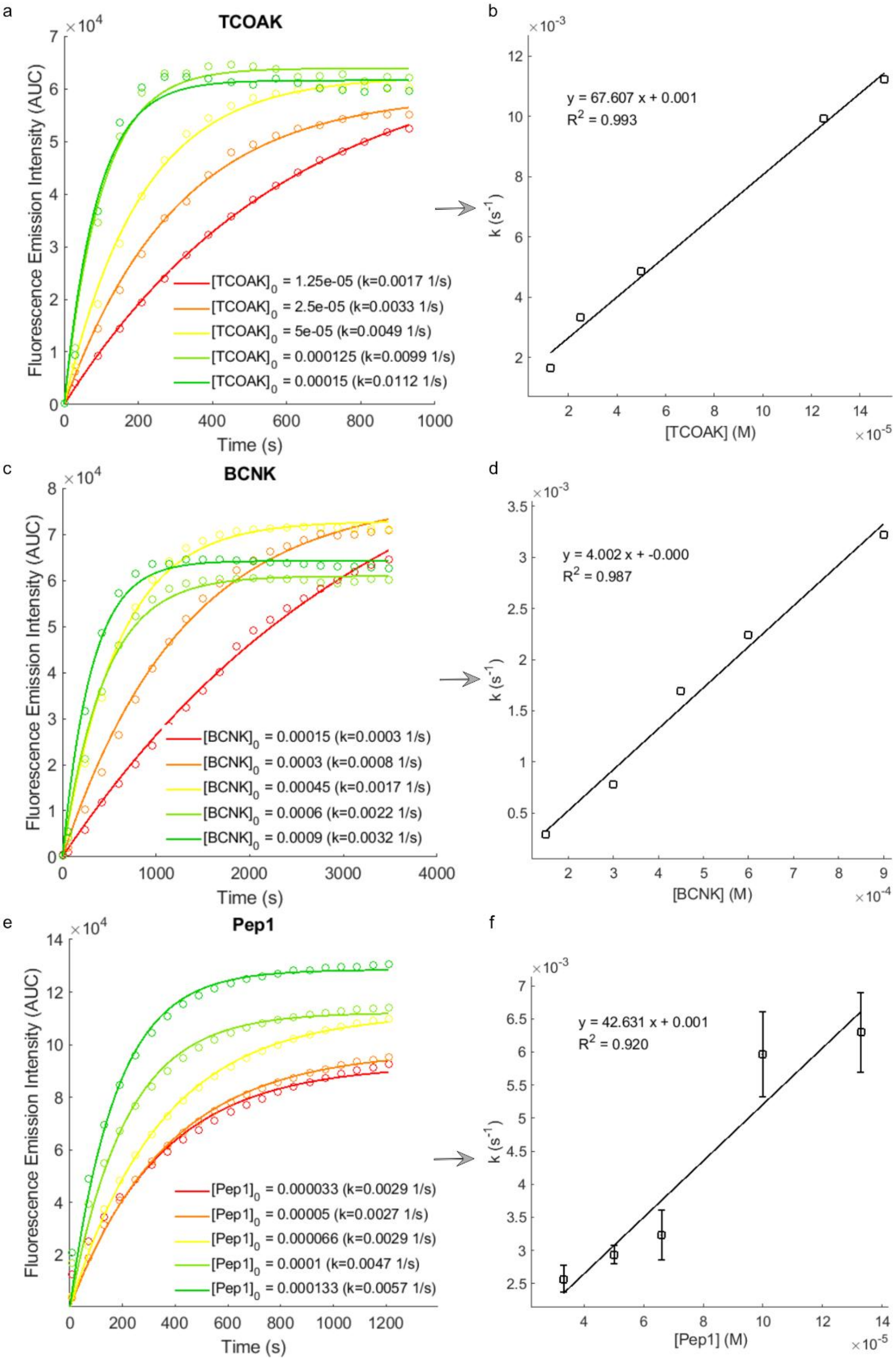


**Figure S24. Kinetic experiment.** (Left) Area under the curve (AUC) of the fluorescence emission intensity as a function of time after the addition of a) TCOA-lysine (TCOAK), c) BCN-lysine (BCNK); e) one representative Pep1 replicate (AcK(BCN)AEAADAEAAK(BCN)-OH). (Right) Linear correlation between the experimentally determined observed rate constants and the concentration of b) TCOAK; d) BCNK; f) Pep1, as a result of the three replicates. For TCOAK and BCNK, concentrations are expressed on an equivalent reactive handle basis (corresponding to half of the real conc.). In the case of the peptide, the concentration calculated on an equivalent reactive handle basis is equivalent to the actual peptide concentration.

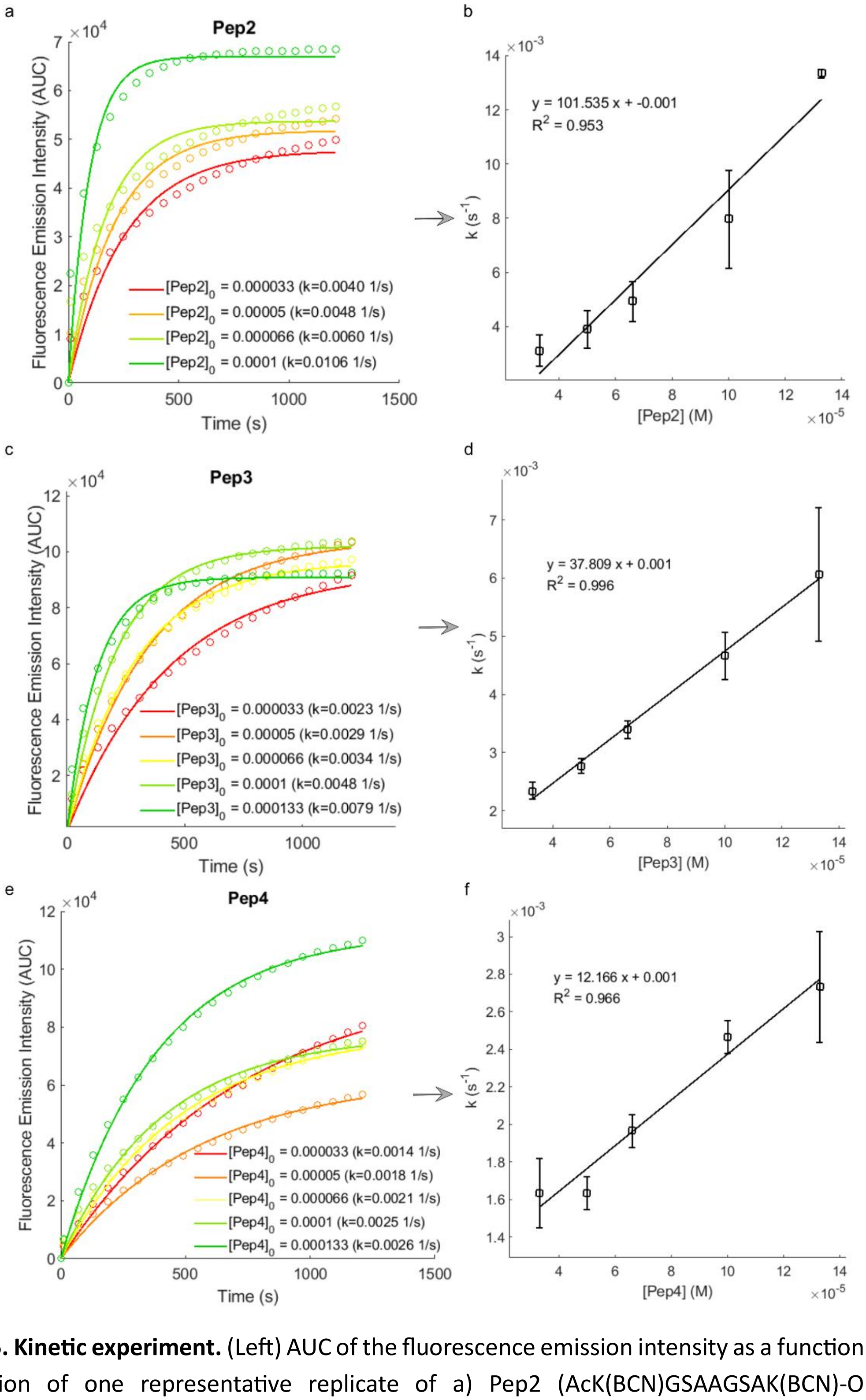


**Figure S25. Kinetic experiment.** (Left) AUC of the fluorescence emission intensity as a function of time after the addition of one representative replicate of a) Pep2 (AcK(BCN)GSAAGSAK(BCN)-OH); c) Pep3 (AcK(BCN)GSAAEAAK(BCN)-OH); e) Pep4 (AcK(BCN)ADAAEADAAEAK(BCN)-OH). (Right) Linear correlation between the experimentally determined observed rate constants and the concentration of b) Pep2, d) Pep3, f) Pep4, as a result of the three replicates. For the peptides, the concentration calculated on an equivalent reactive handle basis is equivalent to the actual peptide concentration.

## Stability monitoring of the peptide-dye conjugate for FC

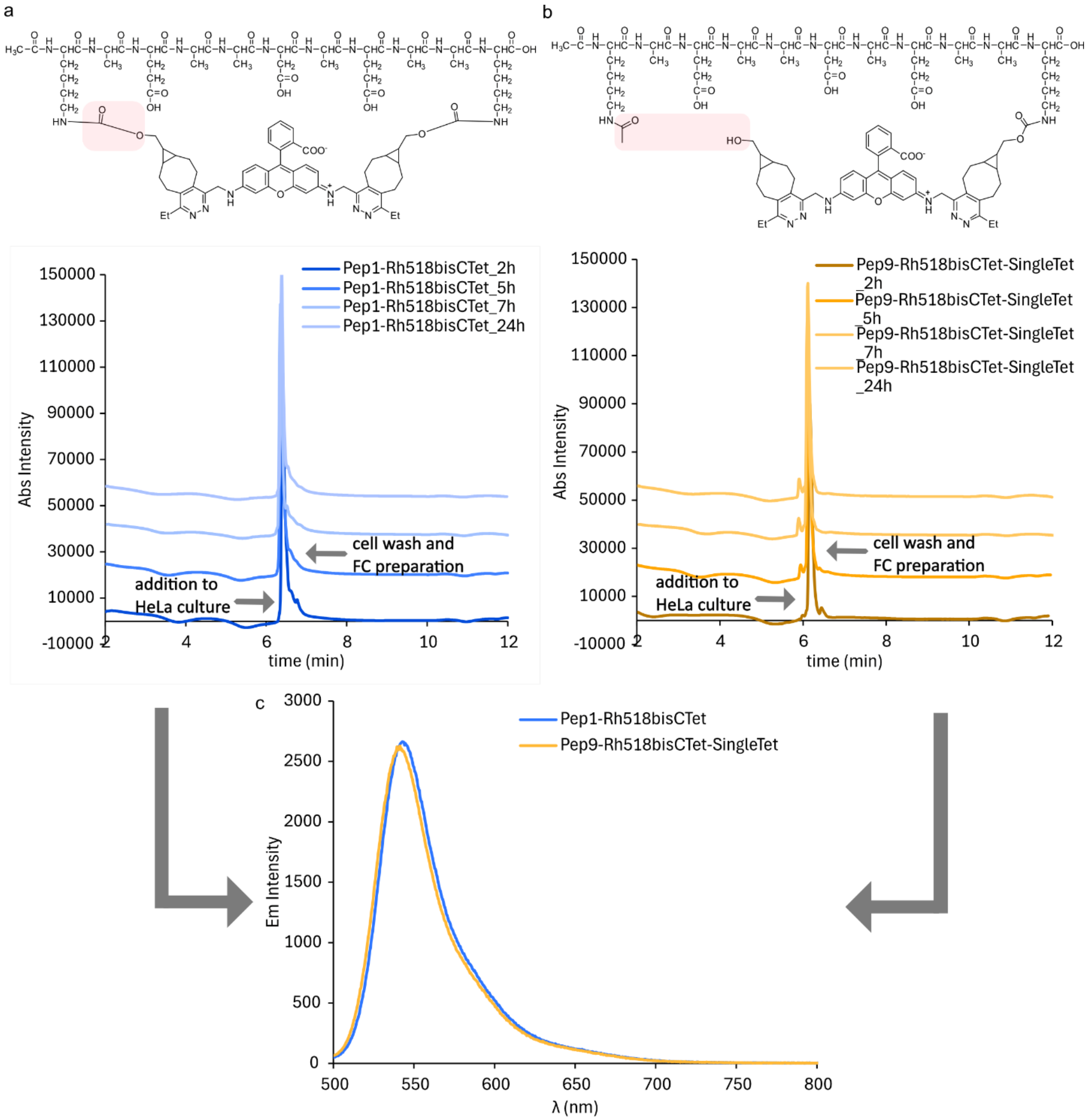


**Figure S26. Chemical structure of Rh518bisCTet-Pep1 and Rh518bisCTet-SingleTet-Pep9 conjugates.** The stability of the preformed peptide-adducts was evaluated under relevant experimental conditions. Samples were prepared by mixing **Rh518bisCTet** and **Rh518bisCTet-SingleTet** (final concentration: 8 µM) with Pep1 and Pep9, respectively, in FluoroBrite DMEM. Formation of the peptide-dye conjugates was monitored by LC-MS, with HPLC chromatograms recorded at 520 nm. After 2 h incubation at RT, the dyes had completely reacted with the peptides, yielding the cyclic peptide conjugate in the case of a) **Rh518bisCTet** with Pep1, and the linear counterpart in the case of b) **Rh518bisCTet-SingleTet** with Pep9. The samples were subsequently incubated at 37 °C for an additional 3 h, after which their stability under these conditions

was assessed, corresponding to the 5 h HPLC chromatogram. To further evaluate probe stability, the samples were maintained at 37 °C for up to 24 h. Fluorescence emission measurements of both samples were also performed to enable a direct and robust comparison between the two systems.

## Flow Cytometry data for HeLa cells

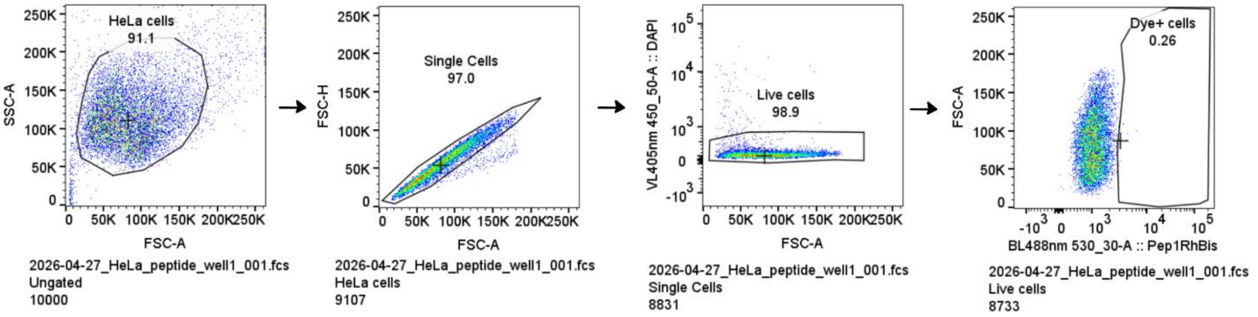


**Figure S27.** Representative gating strategy applied consistently across all samples and measurements in order to get live cells values and median fluorescence intensity (MFI) using FloJo software. In this case, untreated cells are shown. MFI values were calculated using the live-cell gate, whereas the dye-positive cell population is displayed for visualization purposes only.

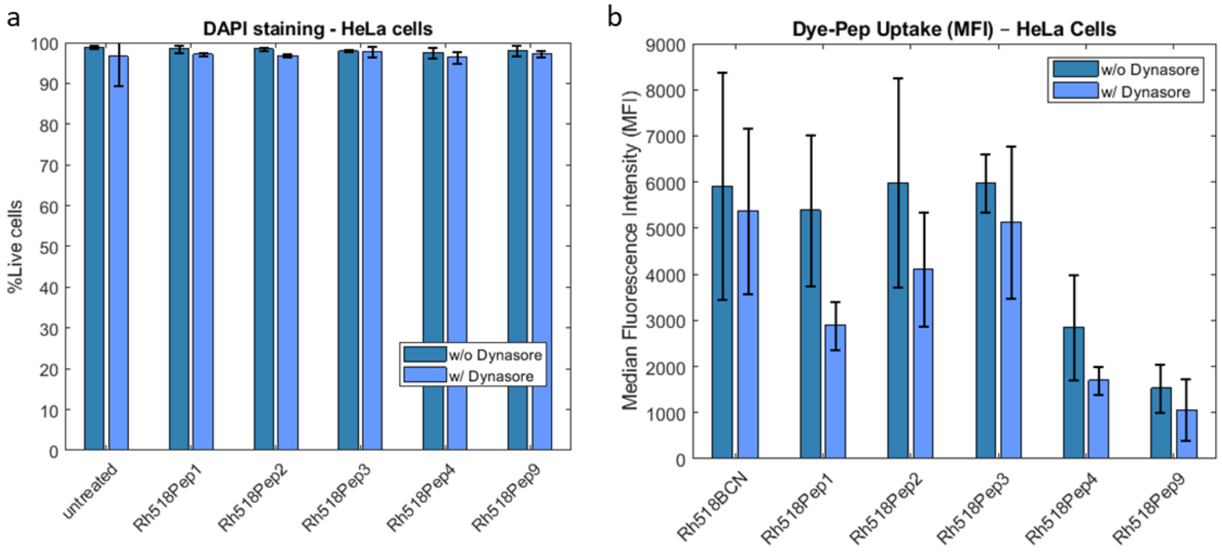


**Figure S28.** Cell viability and cellular uptake of the dye-peptide adduct. a) Cell viability of HeLa cells was tested using DAPI (50 µg/mL), as 1:100 dilution. Percentage of live cells are reported as mean values ± std. b) For the quantification of the dye-peptide uptake in the absence or presence of Dynasore, the MFI was evaluated and reported as median values ± std, after subtraction of background signals from untreated samples and samples treated with Dynasore alone.

a

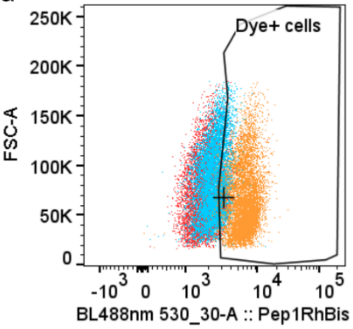


| | Sample Name | Median : BL488nm 530_30-A | Count |
|---|---|---|---|
| | 2026-04-27_HeLa_peptide_well9_009.fcs | 6488 | 8172 |
| | 2026-04-27_HeLa_peptide_well8_008.fcs | 2391 | 8025 |
| | 2026-04-27_HeLa_peptide_well7_007.fcs | 1856 | 9020 |

b

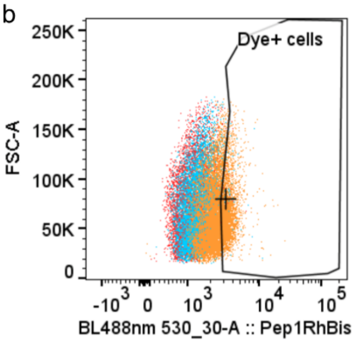


| | Sample Name | Median : BL488nm 530_30-A | Count |
|---|---|---|---|
| | 2026-04-27_HeLa_peptide_well13_013.fcs | 2433 | 9115 |
| | 2026-04-27_HeLa_peptide_well12_012.fcs | 1489 | 9143 |
| | 2026-04-27_HeLa_peptide_well11_011.fcs | 1225 | 9004 |

c

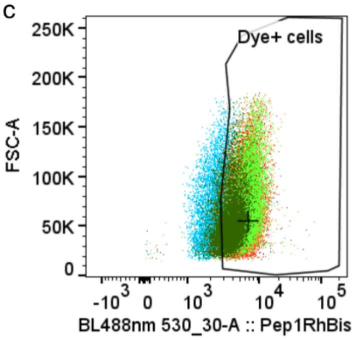


| | Sample Name | Median : BL488nm 530_30-A | Count |
|---|---|---|---|
| | 2026-04-27_HeLa_peptide_well17_017.fcs | 3693 | 9065 |
| | 2026-04-27_HeLa_peptide_well16_016.fcs | 6545 | 8218 |
| | 2026-04-27_HeLa_peptide_well15_015.fcs | 5925 | 8982 |
| | 2026-04-27_HeLa_peptide_well13_013.fcs | 2433 | 9115 |
| | 2026-04-27_HeLa_peptide_well9_009.fcs | 6488 | 8172 |

d

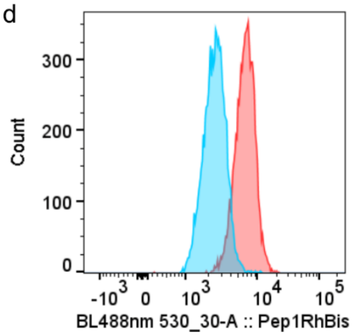


| | Sample Name | Count | Median : BL488nm 530_30-A |
|---|---|---|---|
| | 2026-04-27_HeLa_peptide_well13_013.fcs | 9115 | 2433 |
| | 2026-04-27_HeLa_peptide_well9_009.fcs | 8172 | 6488 |

**Figure S29.** Representative flow cytometry density plots of HeLa cells displaying fluorescence response (x-axis) versus forward scatter area (FSC-A, y-axis) for different concentration (1µM for, 2µM and 5µM) of: a) **Rh518bisCTet**-Pep1 adduct (1µM: well 7; 2µ: well 8; and 5µM: well 9) and b): **Rh518bisCTet**-Pep9 adduct (1µM: well 11; 2µ: well 12; and 5µM: well 13). c) Fluorescence response versus FSC-A for different **Rh518bisCTet**-peptide adducts (Rh518bis-Pep2: well 15; Rh518bis-Pep3: well 16; and Rh518bis-Pep4: well 15), illustrating the adduct-dependent shifts in fluorescence intensity. The dye-positive cell, obtained from the untreated cell sample, is displayed for visualization purposes only (see previous Figure). d) Flow cytometry histograms of the dye-channel showing higher fluorescence intensity for the cyclic peptide

conjugate **Rh518-Pep1** compared to the linear analogue **Rh518-Pep9**, demonstrating the enhanced cellular uptake of the former. Corresponding MFI value for each experimental group is reported in the table and should be subtracted from the untreated controls, which is 1072 for the 488 nm channel relative to this replicate. One representative replicate has been herein reported.

## Protein labeling in vitro

The labeling reaction was performed in a 1 mL Eppendorf tube. 2 µl of protein (20 µM stock solution) was mixed with 0.1 µl of **Rh518bisCTet** (2 mM) in a total 6 µL reaction (1×PBS) for 4 h at 37 °C for the SCO-tagged protein, 30 min for the BCN-tagged protein and 15 min for the TCO-tagged protein. As control for the double labeling, BCN-OH was added after the reaction time has passed (0.2 µl of from 50 mM stock solution) and let it reacted for another 20 min at 37 °C. Each sample was mixed with 5 µL 5×SDS loading dye, boiled for 5 min at 95 °C and loaded on a NuPAGE 4–12 % SDS-PAGE (Invitrogen). After gel electrophoresis in MES (2-(*N*-morpholino)ethanesulfonic acid) buffer (Figure S30a) or in MOPS (3-(*N*-morpholino)propanesulfonic acid) buffer (Figure S30b), the SDS-PAGE was scanned on a fluorescence scanner using green illumination and the 605/50nm emission filter. Finally, the gel was stained with Coomassie blue. Beside the main band corresponding to the MBP (≈ 41 kDa), a minor band at ca. 85 kDa is observed due to the crosslinking reaction between **Rh518bisCTet** and the SCO or BCN of two distinct proteins (red arrow). In the case of BCN-MBP, the ≈85 kDa band was less pronounced and was primarily observed in samples that had not been pre-treated with BCN-OH, suggesting that intermolecular crosslinking may be promoted during the 95 °C heating step, potentially as a result of partial solvent evaporation. (Figure 30b, lane 2-3, 5-6). For the TCO-functionalized protein intermolecular crosslinking is not observed, likely due to the faster kinetics of TCO compared to SCO/BCN or due to a potential cleavage before detection, which could also be promoted by the high temperature required for sample preparation. Moreover, to evaluate the behavior of the system in more complex mixtures, double-labeled BCN-MBP sample was subjected to the click reaction prior to purification (Figure S30b, lanes 7). Only one detectable band was observed.

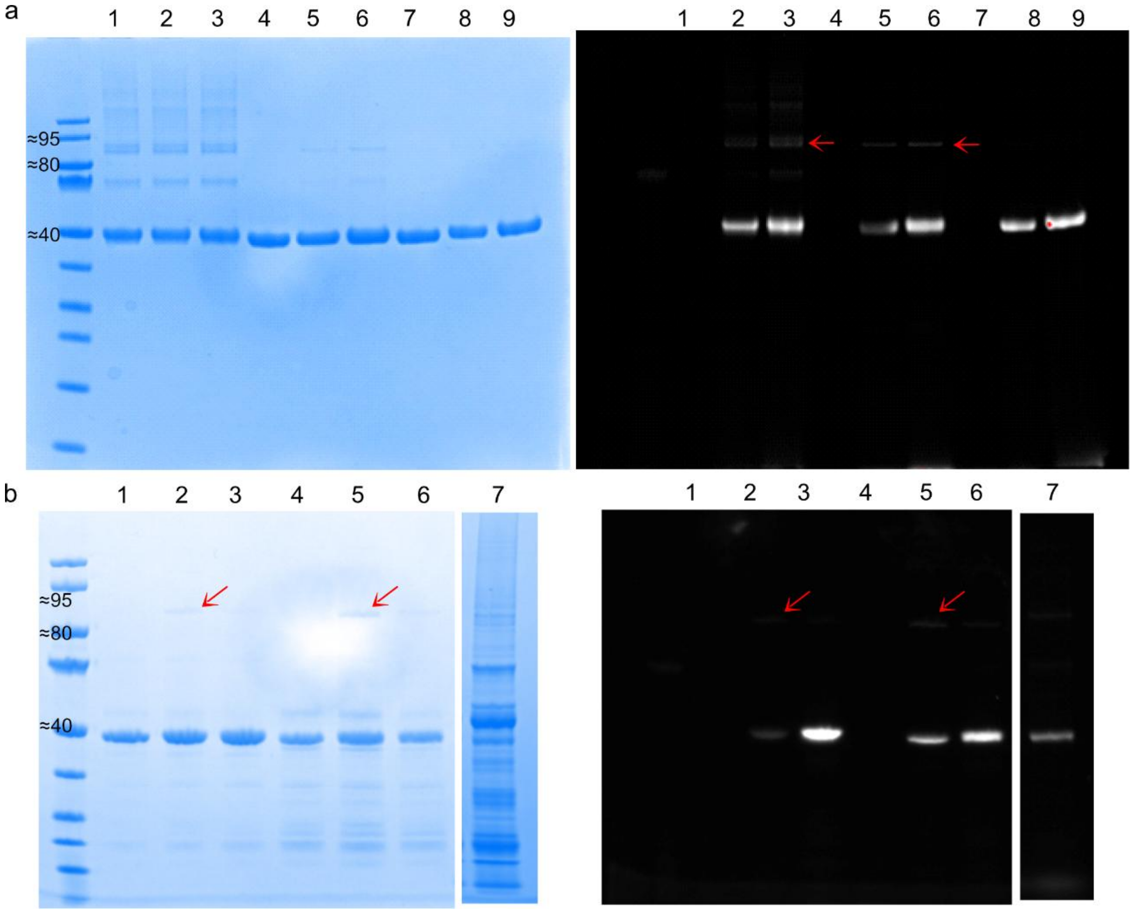


**Figure S30**. SDS-PAGE of MBP. a) Coomassie staining (left) and fluorescence emission (right). a) Lane 1: SCO-peptide-SCO-MBP untreated; Lane 2: SCO-peptide-SCO-MBP with **Rh518bisCTet**; Lane 3: SCO-peptide-SCO-MBP with **Rh518bisCTet** and BCN-OH; Lane 4: K-peptide-SCO-MBP untreated; Lane 5: K-peptide-SCO-MBP with **Rh518bisCTet**; Lane 6: K-peptide-SCO-MBP with **Rh518bisCTet** and BCN-OH; Lane 7: TCO-peptide-TCO-MBP untreated; Lane 8: TCO-peptide-TCO-MBP with **Rh518bisCTet**; Lane 9: TCO-peptide-TCO-MBP with **Rh518bisCTet** and BCN-OH. b) Lane 1: K-peptide-BCN-MBP untreated; Lane 2: K-peptide-BCN-MBP with **Rh518bisCTet**; Lane 3: K-peptide-BCN-MBP with **Rh518bisCTet** and BCN-OH; Lane 4: BCN-peptide-BCN-MBP untreated; Lane 5: BCN-peptide-BCN-MBP with **Rh518bisCTet**; Lane 6: BCN-peptide-BCN-MBP with **Rh518bisCTet** and BCN-OH; Lane 7: BCN-peptide-BCN-MBP before SEC purification with **Rh518bisCTet**.

## Anisotropy data

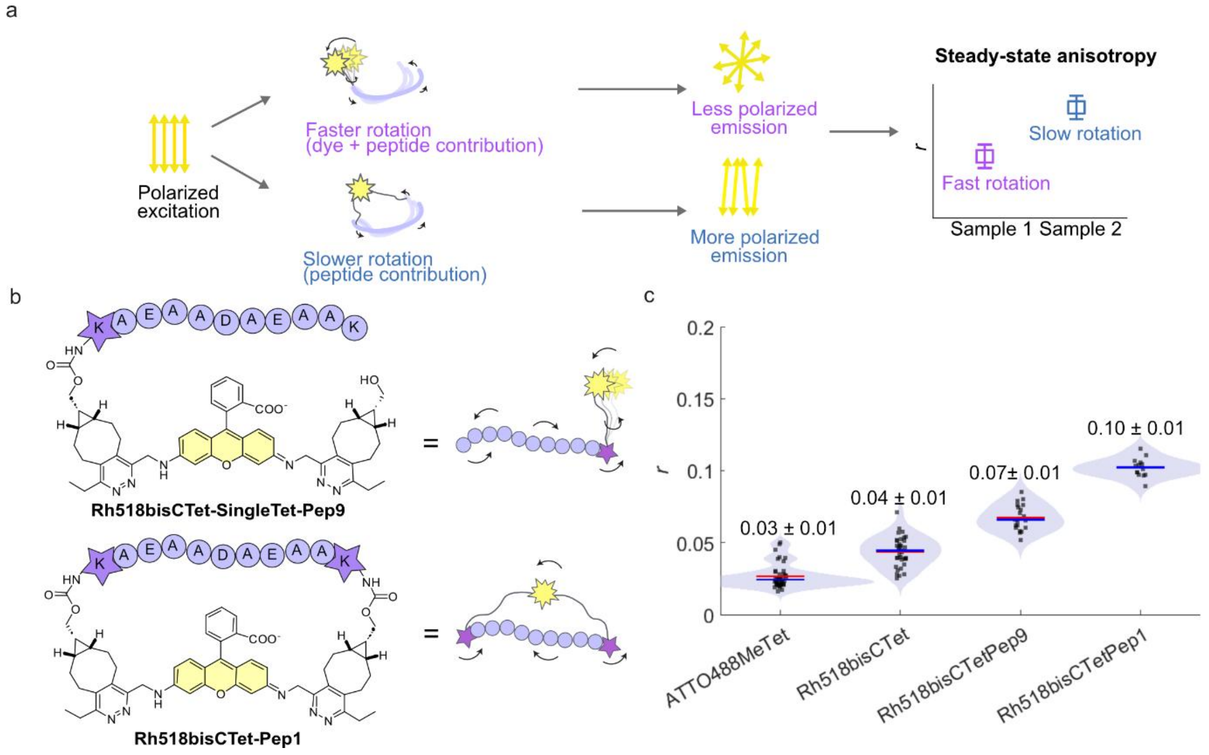


**Figure S31**. a) Schematic illustrations of the contribution of fluorophore rotational freedom to the measured peptide rotation in fluorescence anisotropy experiments with single- and dual-site attachment. b) Steady-state anisotropy of mono- and bis-functionally labeled peptides. Median values are reported as red lines, while mean values are reported as blue lines.

## Click-to-release: doxorubicin as a drug model

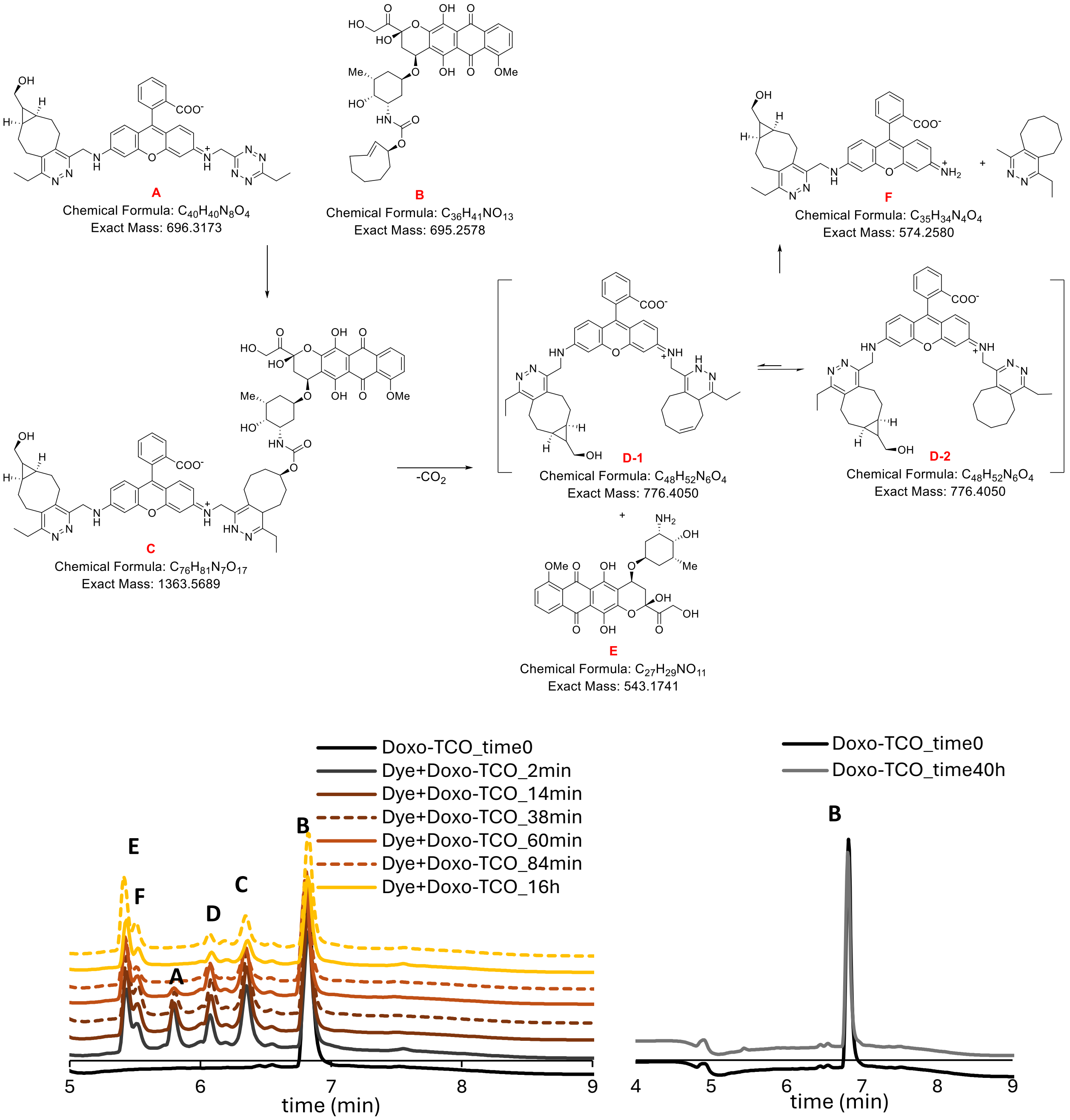


**Figure S32.** Click-to-release between **Rh518bisCTet-SingleTet** and Doxo-TCO. The reaction was performed using 16 µM dye in the presence of 2 equiv of Doxo-TCO (32 µM) in PBS at pH 6.6 and monitored via LC-MS. (Top): Chemical structure of **Rh518bisCTet-SingleTet** (A) and Doxo-TCO (B) and of their clicked product (C) with proposed release pathways. (Bottom, left) HPLC chromatograms showcasing the drug release and the corresponding release profile over time (from 2 min to 24 h, absorbance at 250 nm). (Bottom, right) HPLC chromatogram of Doxo-TCO in PBS at pH 6.6 for 48 h, showcasing its stability in the conditions tested (absorbance at 250 nm). LC-MS settings: eluent A (0.1% formic acid in water) and eluent B ($CH_3CN$) was

used at a flow rate of 0.3 mL/min at 40 °C, injection volume 10 µl. Linear gradient elution was used: 2 min 10% B, 6 min 90%, 8 min 90% B, 8.50 min 10% B.

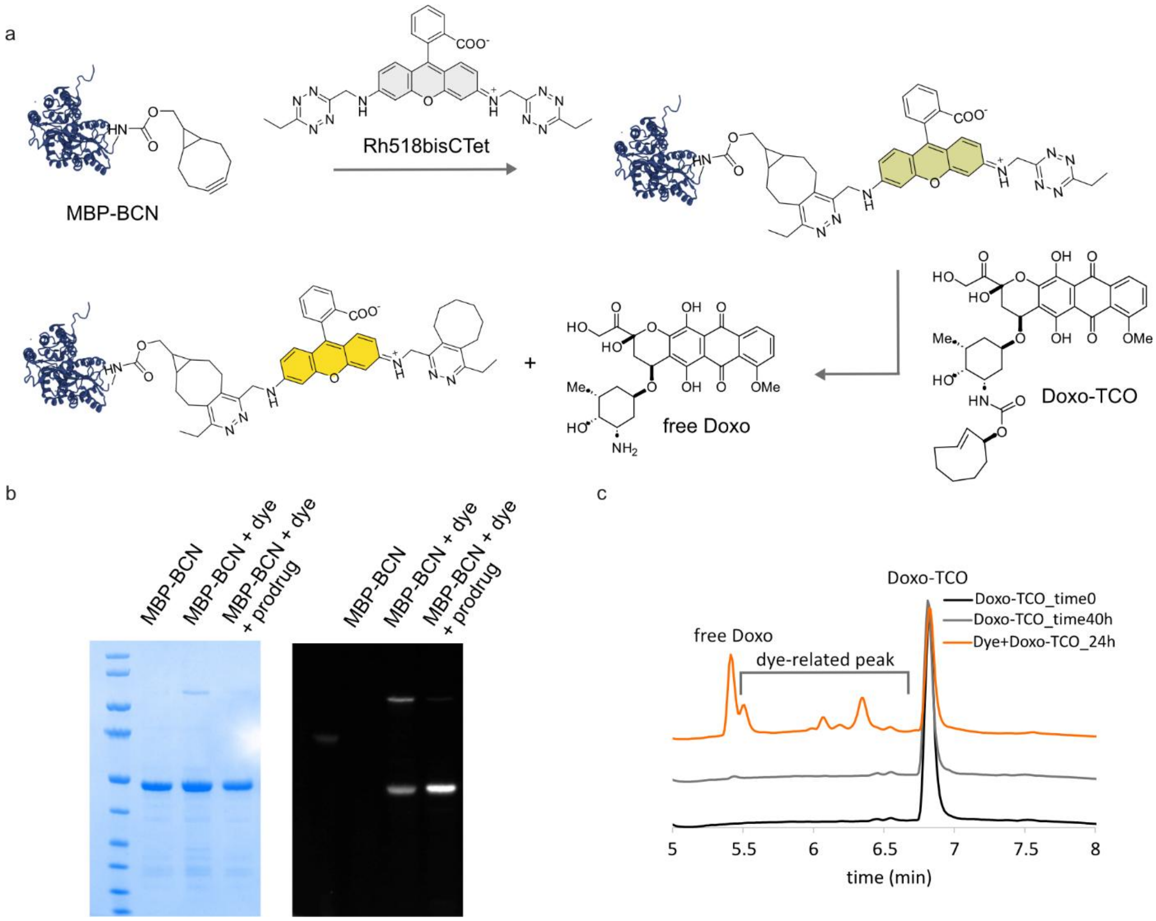


**Figure S33.** a) Schematic representation of K-peptide-BCN-MBP labeling with **Rh518bisCTet** and Doxo-TCO. b) SDS-PAGE of MBP in MOPS buffer: Coomassie staining (left) and fluorescence emission using green illumination and the 605/50nm emission filter (right). Lane 1: K-peptide-BCN-MBP untreated (concentration 2 µM); Lane 2: K-peptide-BCN-MBP with **Rh518bisCTet** (concentration 2 µM, 10 µM, respectively); Lane 3: K-peptide-BCN-MBP with **Rh518bisCTet** and Doxo-TCO (concentration 2 µM, 10 µM, 26 µM, respectively). c) HPLC chromatograms acquired at LC-MS from Figure S32, showcasing the stability of Doxo-TCO before clicking and the corresponding release of free Doxo after 24 h incubation at RT in the presence of **Rh518bisCTet-SingleTet.**

## Live cell data

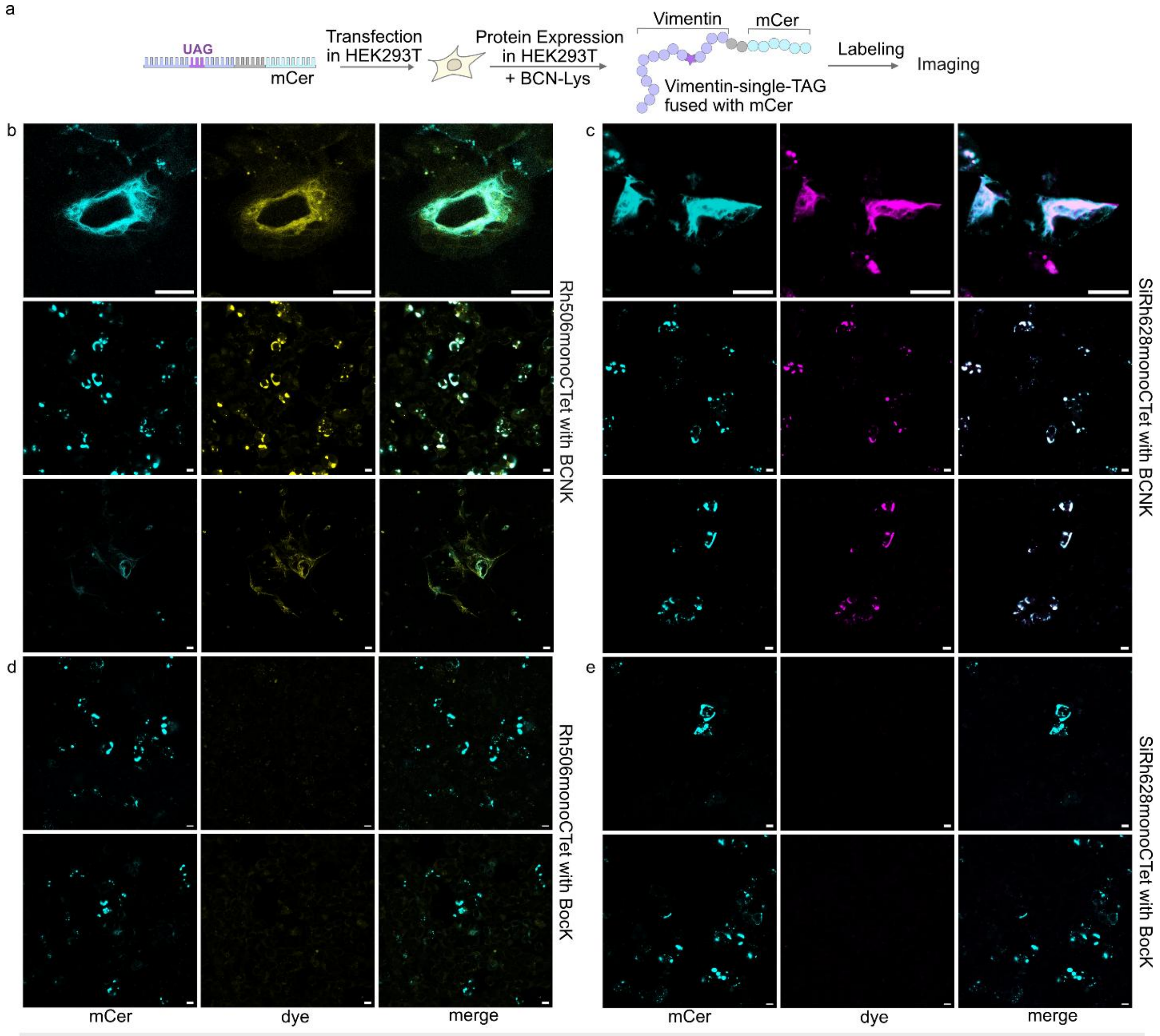


**Figure S34**. a) Single-amber GCE for site-specific incorporation of ncAA into the Vim-mCer construct. b-d) Confocal microscopy images of single-amber Vim-mCer protein in live HEK293T cells treated with BCNK (b, c) and BocK (d, e), as blank control for GCE experiments, and the mono-functional dyes: **Rh506monoCTet** (b, d); and **SiRh628monoCTet** (c, e). The images were acquired under the same microscope conditions as at least three replicates (different days, different cell passages): 514 nm laser, emission filter 525-625 nm, laser power 20% for **Rh506monoCTet** and 633 nm laser, emission filter 643-800 nm, laser power 15%. Brightness and contrast have been consistently adjusted. Scale bar 10 µm.

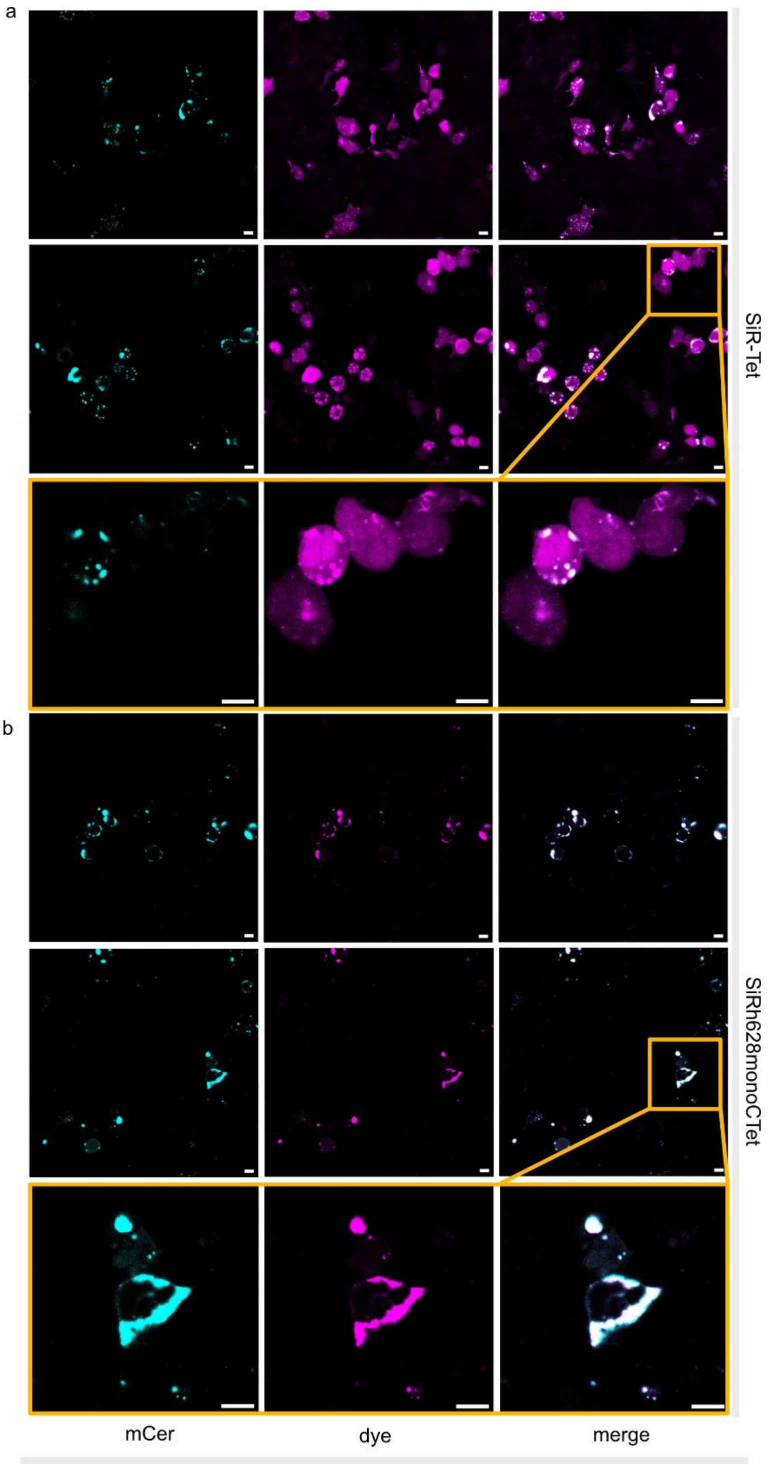


**Figure S35**. Confocal microscopy image of single-amber Vim-mCer protein in live HEK293T cells treated with (a) the commercial SiR-Tet (from Spirochrome) and (b) **SiRh628monoCTet**, as comparison. The images were acquired on the same day under the same microscope conditions and as at least two replicates (different day, different cell passages): 633 nm laser, emission filter 643-800 nm, laser power 15%. Scale bar 10 µm.

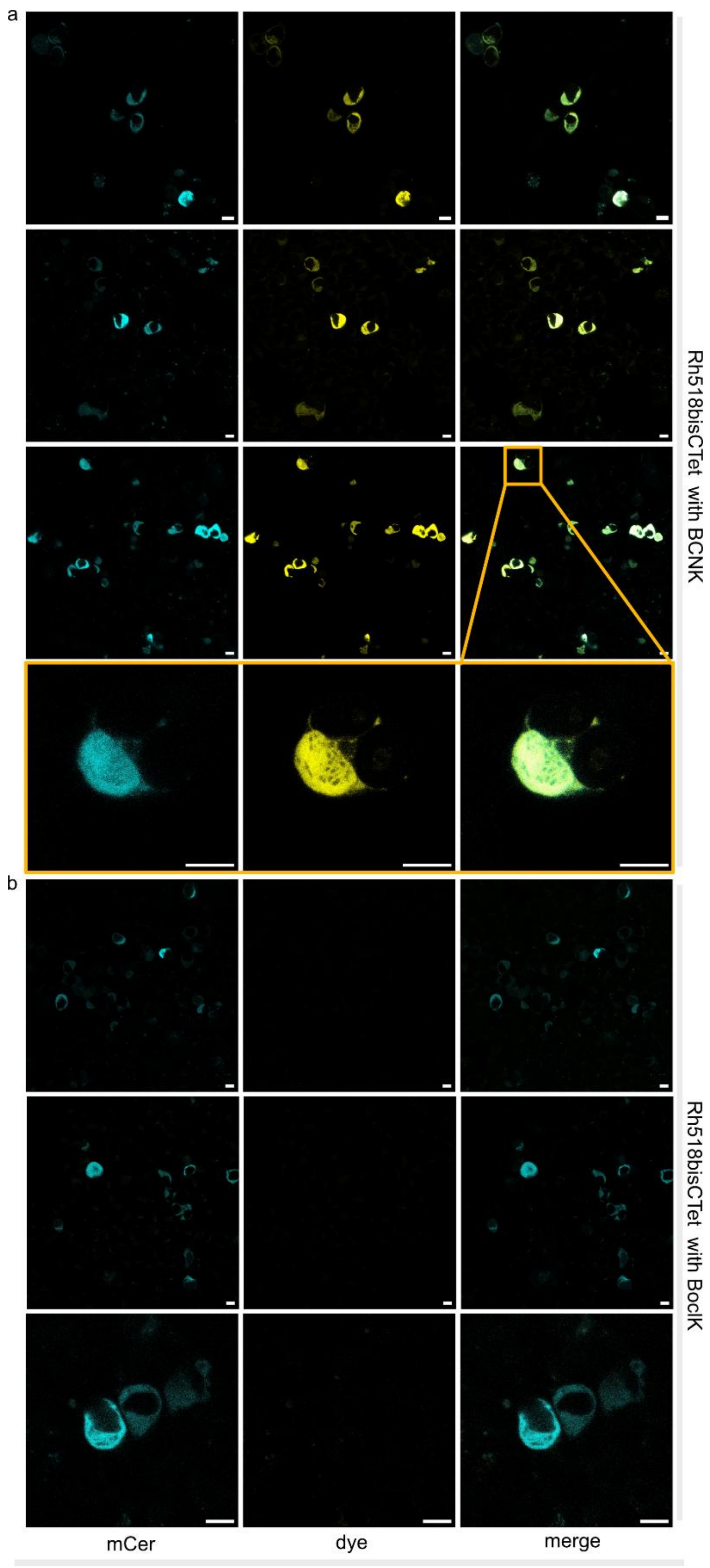


**Figure S36**. Confocal microscopy image of double-amber Vim-mCer protein in live HEK293T cells treated with (a) BCNK or (b) BocK, as blank control for GCE experiments, in the presence of the bis-functional dye, **Rh518bisCTet**. The images were acquired on the same day under the same microscope conditions, as at

least three replicates (different day, different cell passages): 514 nm laser, emission filter 525-625 nm, laser power 20%. Brightness and contrast have been consistently adjusted. Scale bar 10 µm.

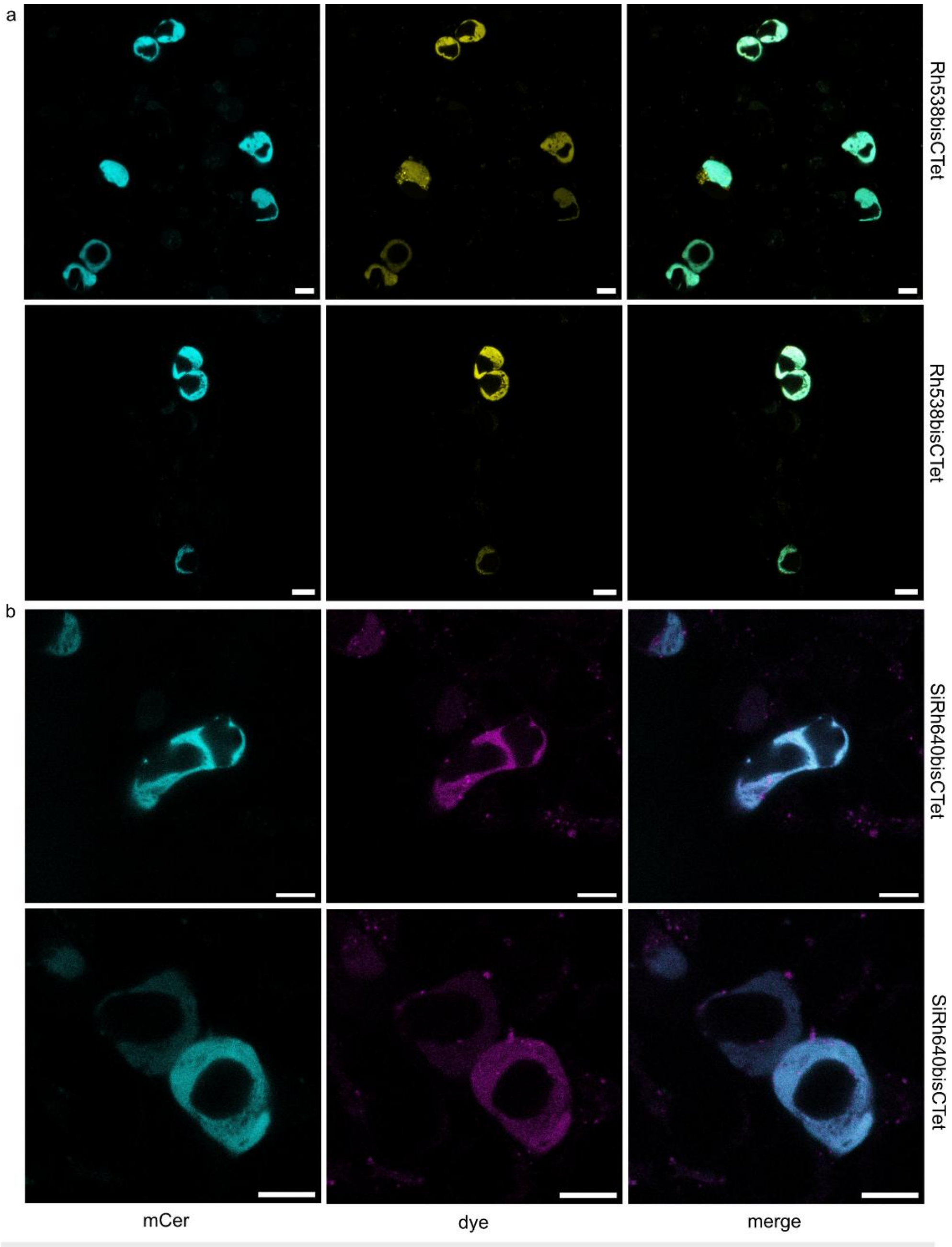


**Figure S37**. Confocal microscopy image of double-amber Vim-mCer protein in live HEK293T cells treated with BCNK as ncAA. a) Labeling with **Rh538bisCTet**. b) Labeling with **SiRh640bisCTet**. With **SiRh640bisCTet** poor brightness is observed due to the prevalence of the non-fluorescent closed form at physiological pH (see Figure S8). Microscope settings for **Rh538bisCTet**: 514 nm laser, emission filter 560-640 nm, laser power 20%; for **SiRh540bisCTet**: 633 nm laser, emission filter 643-800 nm, laser power 30%. Brightness and contrast have been consistently adjusted. Scale bar 10 µm.

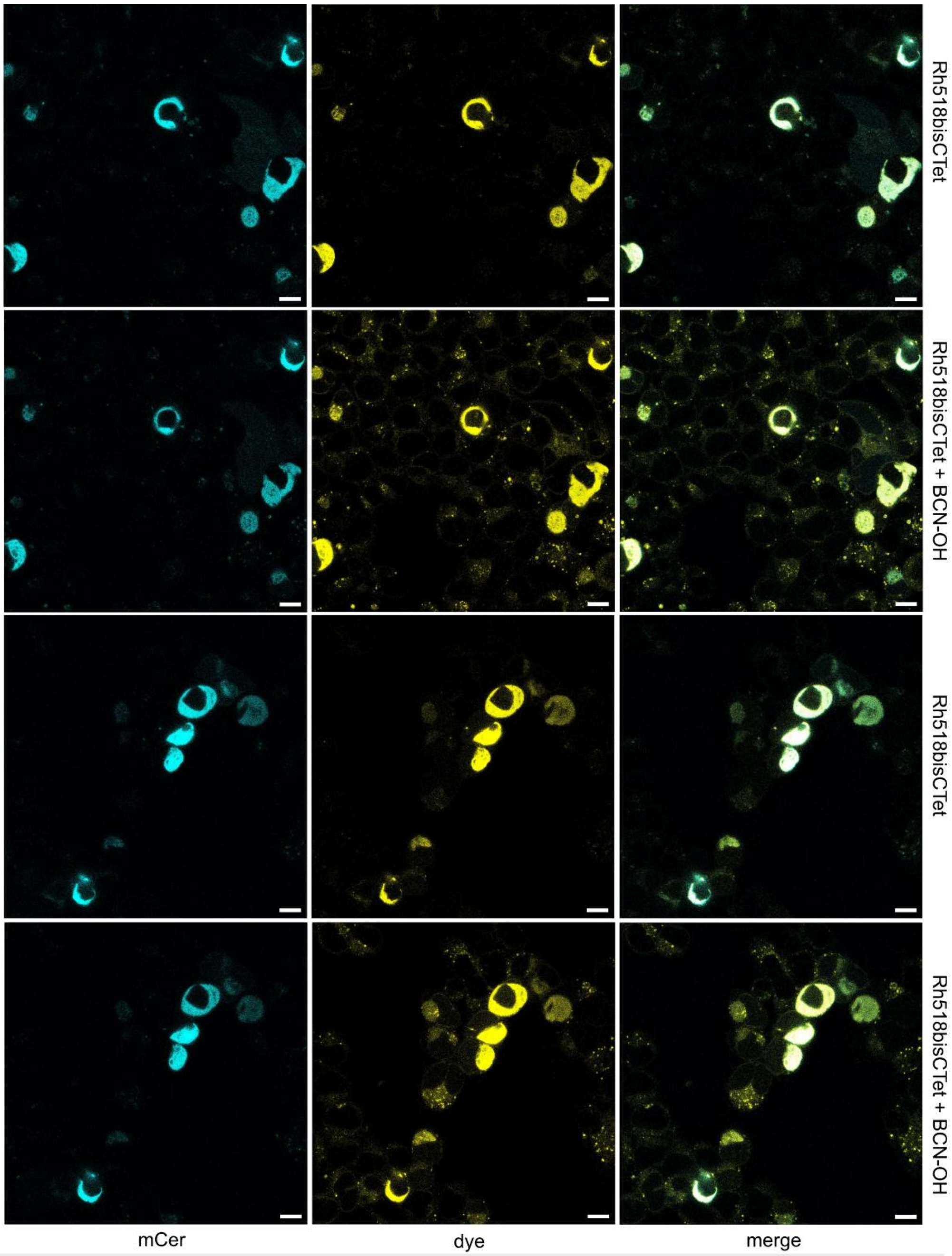


**Figure S38.** Confocal microscopy images of Vim-mCer in live HEK293T cells, using BCNK as ncAA and **Rh518bisCTet** for fluorescent labeling. Same well and images were acquired before and after addition of BCN-OH. Media was replaced with fresh FluoroBrite DMEM containing 5 µM of BCN-OH and images were acquired after 20 min at RT. Scale bar 10 µm. Brightness and contrast have been adjusted consistently for better visualization between +/- BCN-OH.

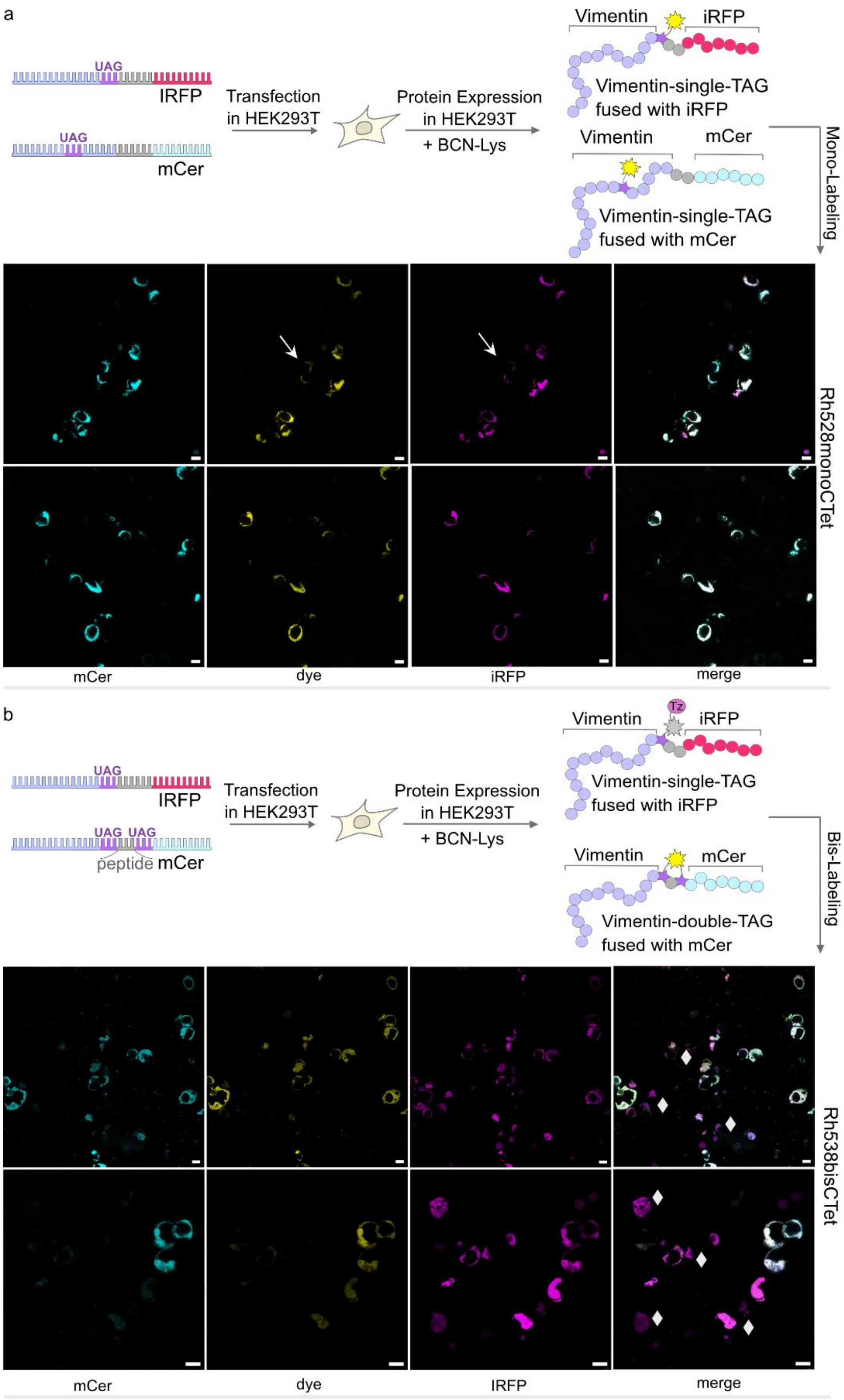


**Figure S39.** Confocal microscopy images of HEK293T cells co-transfected with either (a) two single-amber constructs (single-TAG-Vim-mCer and single-TAG-Vim-iRFP) and labeled with the mono-functional dye **Rh528monoCTet**, or (b) a combination of single- and double-amber constructs (single-TAG-Vim-iRFP and double-TAG-Vim-mCer) and labeled with the bis-functional dye **Rh538bisCTet**. White arrows in (a) highlight

stochastic labeling arising from non-selective labeling of both single-TAG proteins by the mono-functional dye. In contrast, diamonds in (b) indicate the absence/reduce labeling on the single-TAG protein when using the bis-functional dye. Consequently, a higher degree of colocalization between the dye and mCer channels is observed specifically for the double-TAG protein. Microscope settings for **Rh528monoCTet**: 405 nm laser, emission filter 420-480 nm, laser power 5%; 514 nm laser, emission filter 560-640 nm, laser power 10%; for **Rh528bisCTet**: 405 nm laser, emission filter 420-480 nm, laser power 10%; 514 nm laser, emission filter 560-640 nm, laser power 20%. For single-amber GCE in (a), the laser power had to be reduced in both channels due to the higher expression. Scale bar 10 µm.

## Flow Cytometry data for HEK293T cells

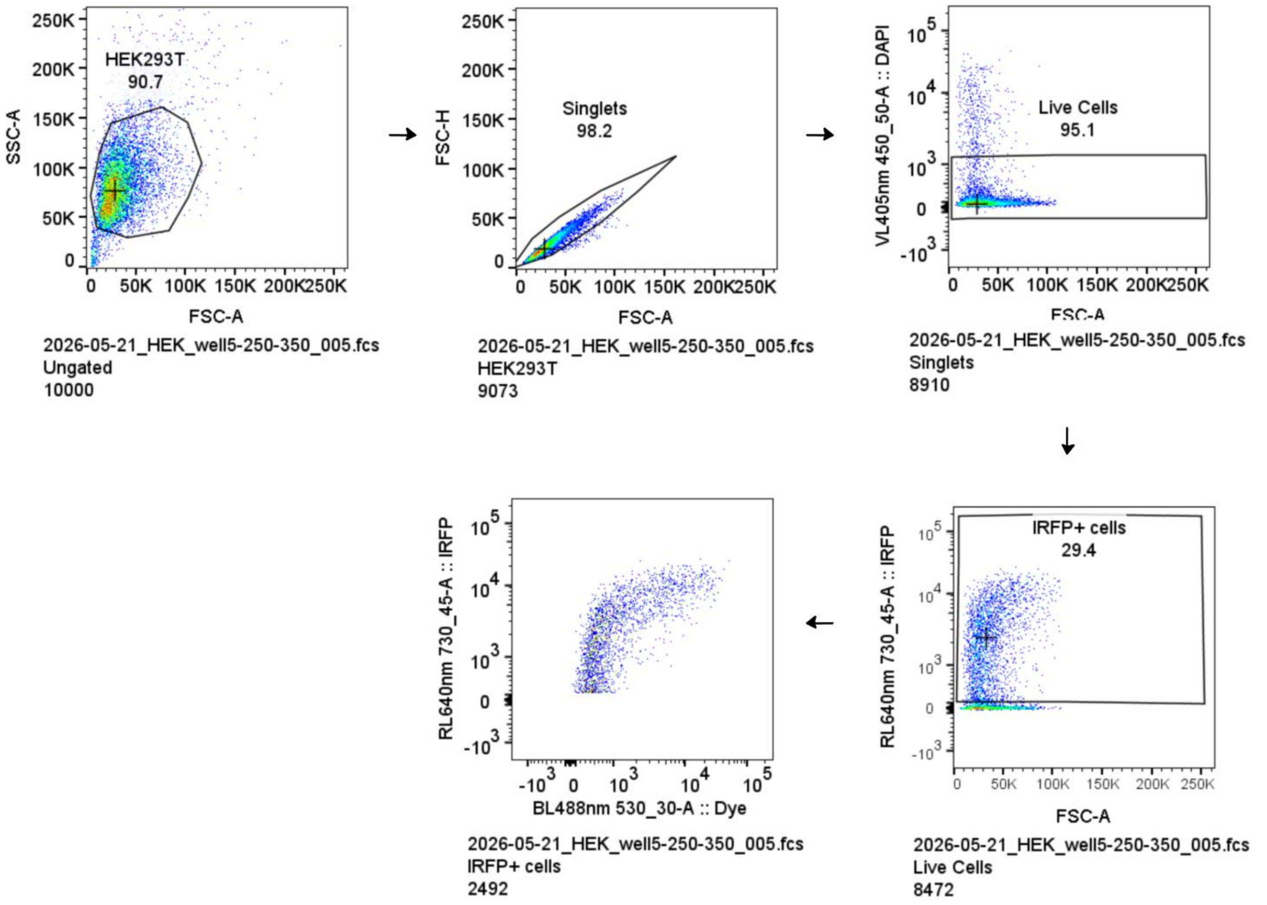


**Figure S40.** Representative gating strategy applied consistently across all samples and measurements in order to get live cells values and MFI intensity using FloJo software. In this case, cells treated with BCNK and **Rh506monoCTet** are shown for illustrative purposes. MFI values were calculated using the iRFP positive cell gate.

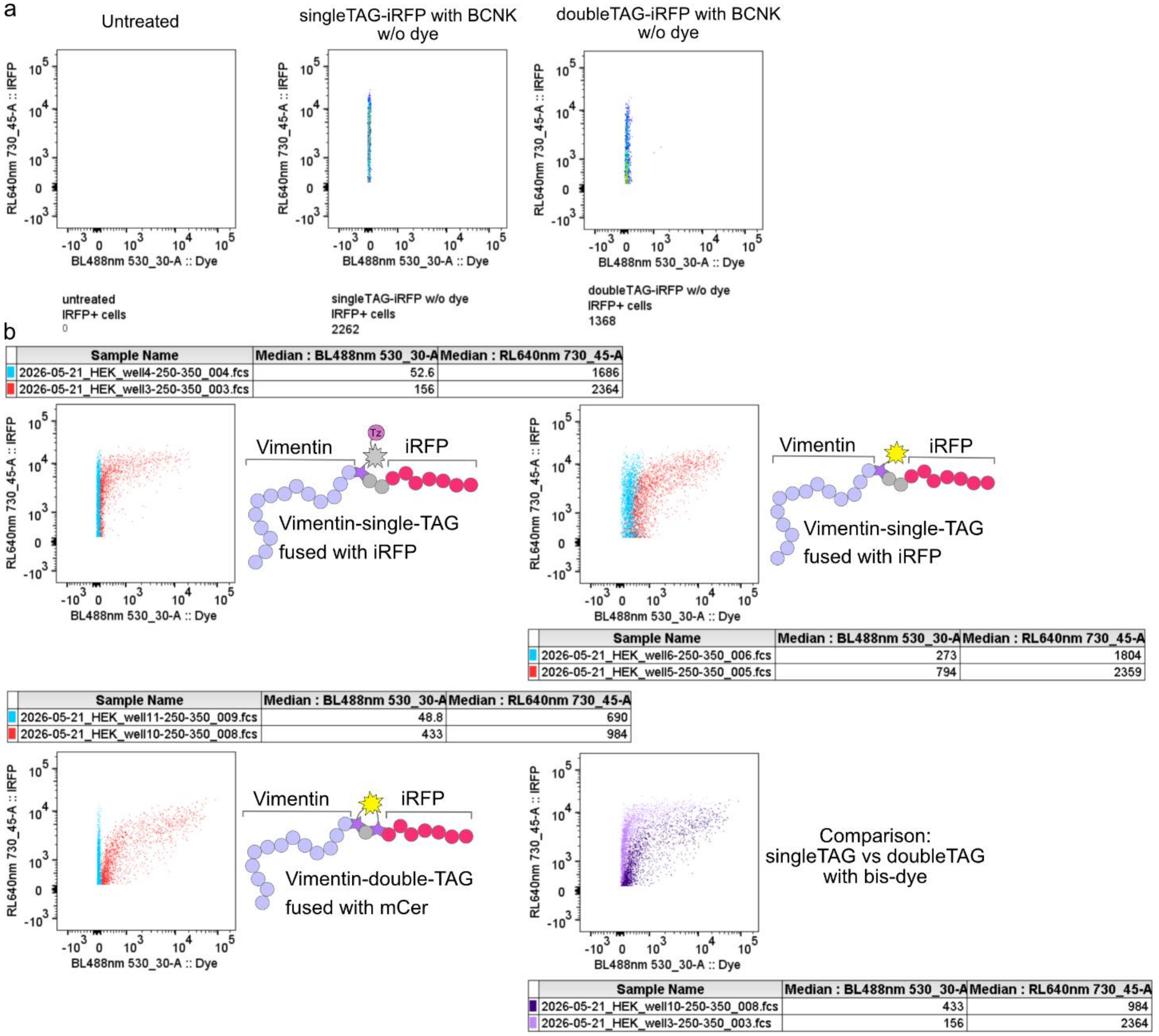


| Sample Name | Median : BL488nm 530_30-A | Median : RL640nm 730_45-A |
|---|---|---|
| 2026-05-21_HEK_well4-250-350_004.fcs | 52.6 | 1686 |
| 2026-05-21_HEK_well3-250-350_003.fcs | 156 | 2364 |

| Sample Name | Median : BL488nm 530_30-A | Median : RL640nm 730_45-A |
|---|---|---|
| 2026-05-21_HEK_well6-250-350_006.fcs | 273 | 1804 |
| 2026-05-21_HEK_well5-250-350_005.fcs | 794 | 2359 |

| Sample Name | Median : BL488nm 530_30-A | Median : RL640nm 730_45-A |
|---|---|---|
| 2026-05-21_HEK_well11-250-350_009.fcs | 48.8 | 690 |
| 2026-05-21_HEK_well10-250-350_008.fcs | 433 | 984 |

| Sample Name | Median : BL488nm 530_30-A | Median : RL640nm 730_45-A |
|---|---|---|
| 2026-05-21_HEK_well10-250-350_008.fcs | 433 | 984 |
| 2026-05-21_HEK_well3-250-350_003.fcs | 156 | 2364 |

**Figure S41.** FC analysis of iRFP-positive cells showing fluorescence intensity in the red channel (640 nm laser, y-axis) versus fluorescence in the green channel (488 nm laser, x-axis) across untreated (a, left), single-amber-Vim-iRFP treated cells in absence of dye (a, middle), double-amber-Vim-iRPF treated cells in absence of dye (a, left) and dye-treated conditions (b). Cells were labeled with **Rh506monoCTet** [in the presence of BCNK (well 5) or in the presence of BocK (well 6)] or **Rh518bisCTet** [single-amber-Vim-iRFP: in the presence of BCNK (well 3) or in the presence of BocK (well 4); double-amber-Vim-iRFP: in the presence of BCNK (well 10) or in the presence of BocK (well 11)]. Populations were gated on iRFP-expressing cells, and mean fluorescence intensity (MFI) in the green channel was quantified for each condition as described in the Fig. S40. Untreated and no-dye controls established baseline fluorescence, while samples treated with different dyes under distinct conditions showed variable shifts in green fluorescence intensity. Corresponding MFI values for each experimental group are reported in the table and should be subtracted from the untreated controls, which are 14.6 for the green channel and 1.28 for the red channel relative to this replicate. One representative replicate has been herein reported.

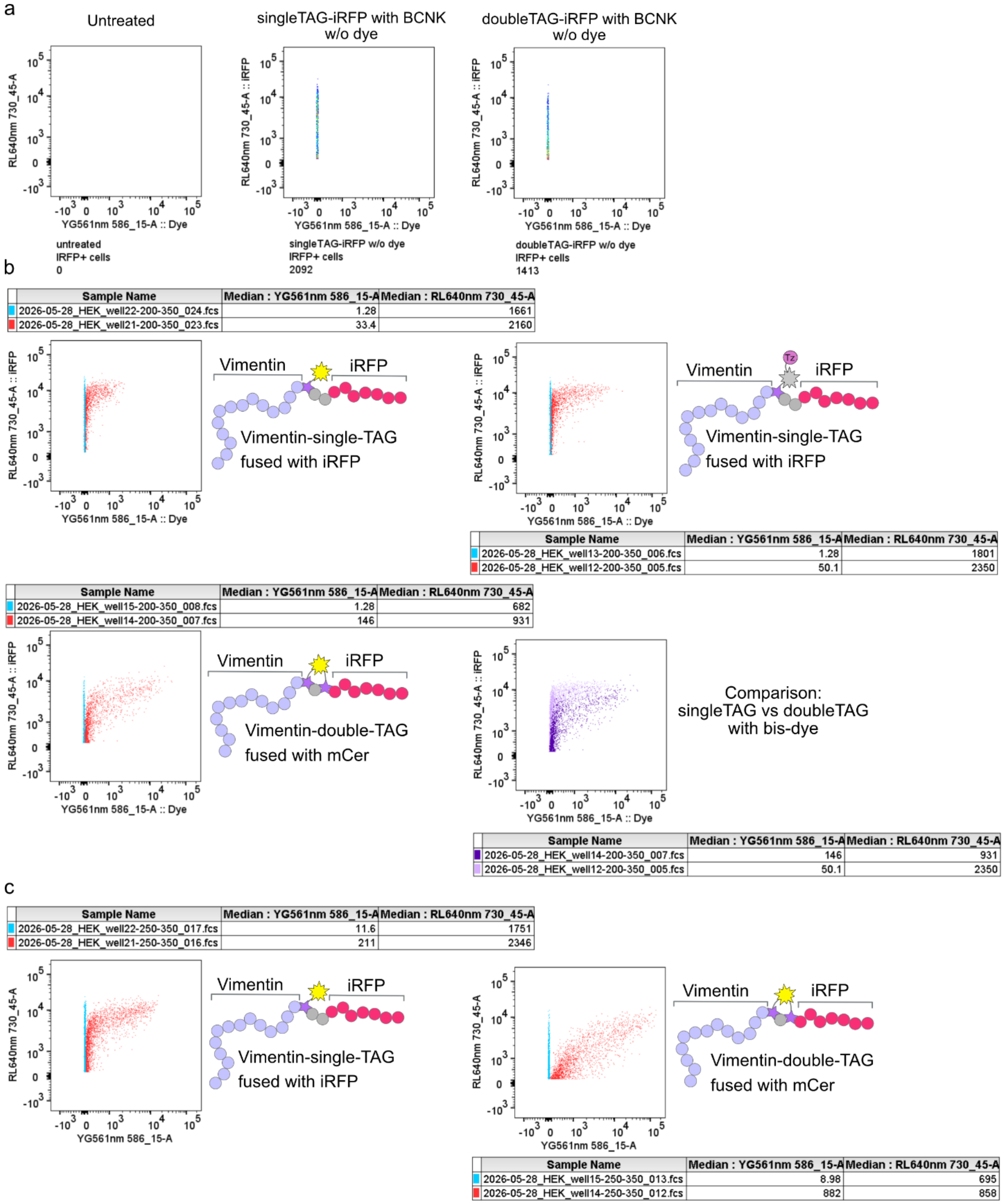


| Sample Name | Median : YG561nm 586_15-A | Median : RL640nm 730_45-A |
|---|---|---|
| 2026-05-28_HEK_well22-200-350_024.fcs | 1.28 | 1661 |
| 2026-05-28_HEK_well21-200-350_023.fcs | 33.4 | 2160 |

| Sample Name | Median : YG561nm 586_15-A | Median : RL640nm 730_45-A |
|---|---|---|
| 2026-05-28_HEK_well13-200-350_006.fcs | 1.28 | 1801 |
| 2026-05-28_HEK_well12-200-350_005.fcs | 50.1 | 2350 |

| Sample Name | Median : YG561nm 586_15-A | Median : RL640nm 730_45-A |
|---|---|---|
| 2026-05-28_HEK_well15-200-350_008.fcs | 1.28 | 682 |
| 2026-05-28_HEK_well14-200-350_007.fcs | 146 | 931 |

| Sample Name | Median : YG561nm 586_15-A | Median : RL640nm 730_45-A |
|---|---|---|
| 2026-05-28_HEK_well14-200-350_007.fcs | 146 | 931 |
| 2026-05-28_HEK_well12-200-350_005.fcs | 50.1 | 2350 |

| Sample Name | Median : YG561nm 586_15-A | Median : RL640nm 730_45-A |
|---|---|---|
| 2026-05-28_HEK_well22-250-350_017.fcs | 11.6 | 1751 |
| 2026-05-28_HEK_well21-250-350_016.fcs | 211 | 2346 |

| Sample Name | Median : YG561nm 586_15-A | Median : RL640nm 730_45-A |
|---|---|---|
| 2026-05-28_HEK_well15-250-350_013.fcs | 8.98 | 695 |
| 2026-05-28_HEK_well14-250-350_012.fcs | 882 | 858 |

**Figure S42.** FC analysis of iRFP-positive cells showing fluorescence intensity in the red channel (640 nm laser, y-axis) versus fluorescence in the green channel (561 nm laser, x-axis) across untreated (a, left), single-amber-Vim-iRFP treated cells in absence of dye (a, middle), double-amber-Vim-iRPF treated cells in absence of dye (a, left) and dye-treated conditions (b). Cells were labeled with **Rh506monoCTet** [in the presence of BCNK (well 21) or in the presence of BocK (well 22)] or with **Rh538bisCTet** [single-amber-Vim-

iRFP: in the presence of BCNK (well 12) or in the presence of BocK (well 13); double-amber-Vim-iRFP: in the presence of BCNK (well 14) or in the presence of BocK (well 15)]. Given the weak excitation of **Rh528monoCTet** with the 561 nm laser, a higher voltage was used for illustrative purposes in (c), with the yellow laser power set to 250 instead of 200. Under these conditions, the signal from **Rh538bisCTet** was close to saturation, resulting in potential signal loss. Populations were gated on iRFP-expressing cells, and MFI in the yellow channel was quantified for each condition as described in Figure S40. Corresponding MFI values for each experimental group are reported in the table and should be subtracted from the untreated controls, which for the yellow channel are 0 under the 200 V yellow power laser and 5.13 under the 250 V yellow power laser, and 1.28 for the red channel relative to this replicate. One representative replicate has been herein reported.

a

b

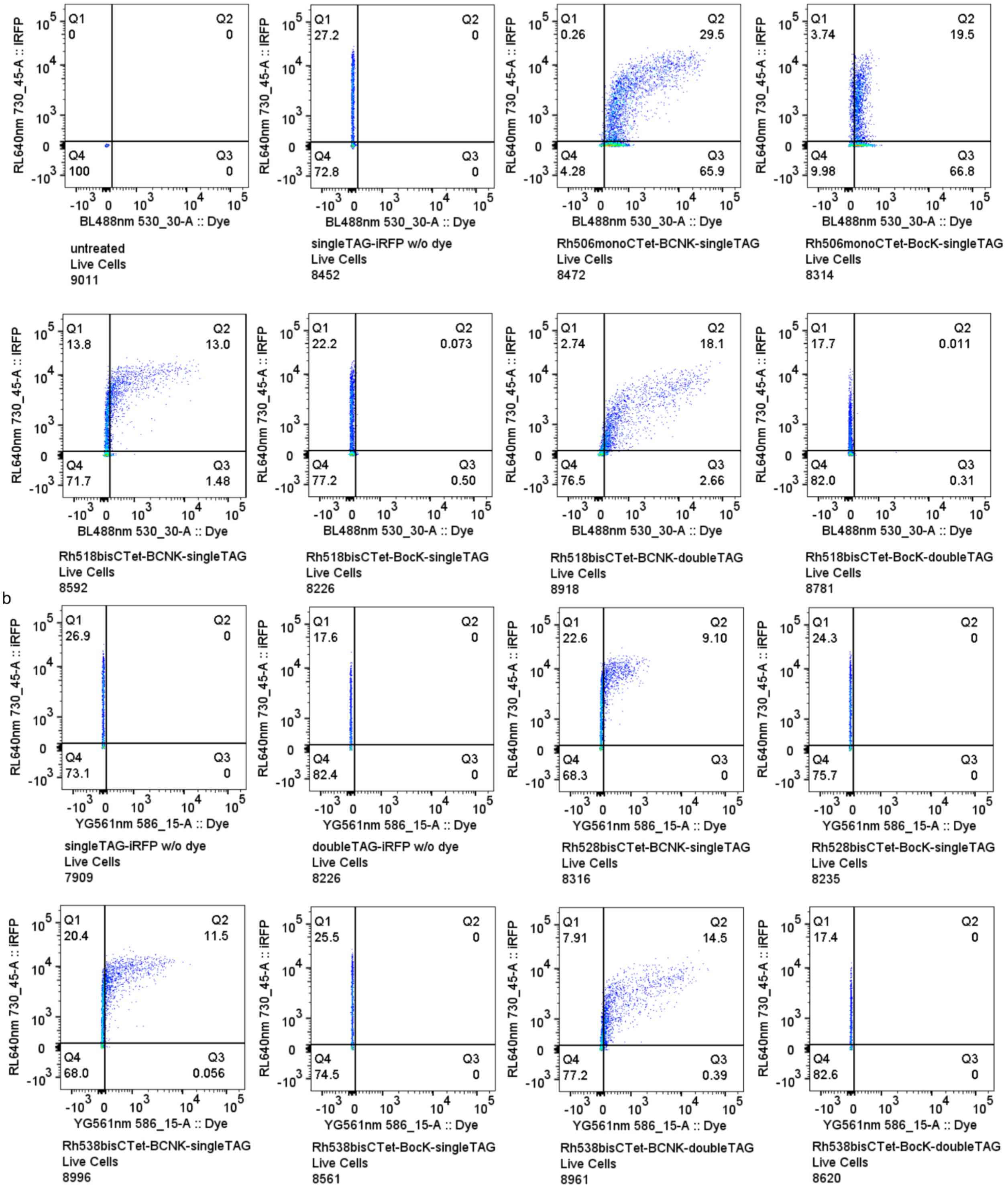


**Figure S43.** FC analysis of live HEK293T cells prior to gating for iRFP-positive cells, included for transparency. The iRFP-positive gated population corresponds to quadrants Q1 and Q2, with Q2 representing the dye-positive population. Red fluorescence (640 nm laser, y-axis) is plotted against green fluorescence (488 nm laser, x-axis) for the indicated samples. Gates were set at 130 fluorescence intensity units for the iRFP channel, 160 for the green channel, and 100 for the yellow channel.

## Summary of synthetic steps

**1. Synthetic route to bromomethyl tetrazine**

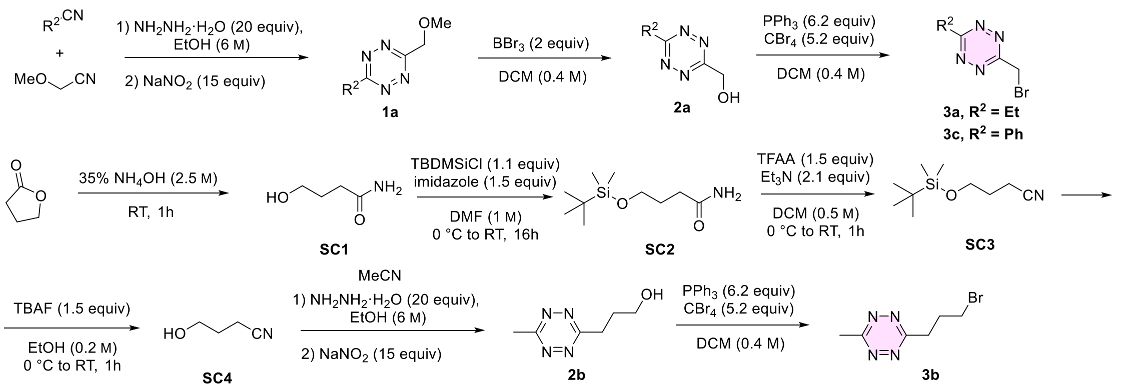


**2. Synthetic route to rhodamine precursors**

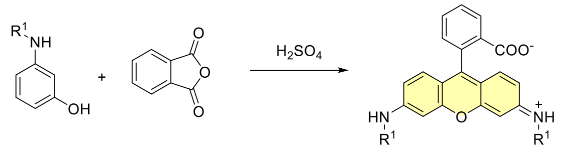


**3. Synthetic route to silicon rhodamine precursors**

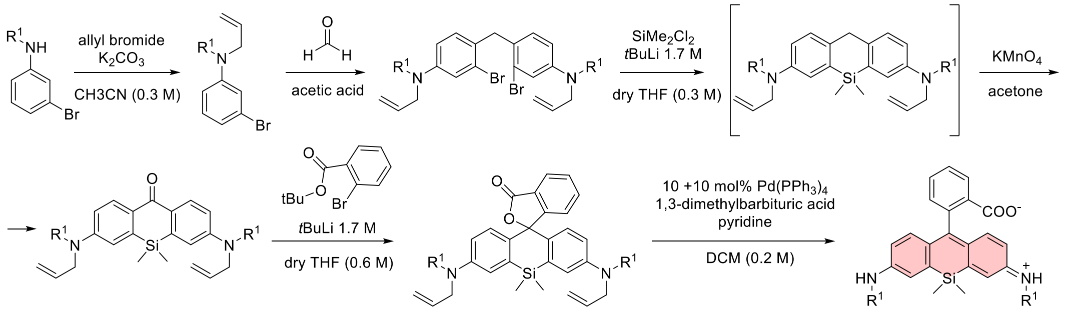


**4. Key late-stage functionalization with bromomethyl tetrazine derivatives**

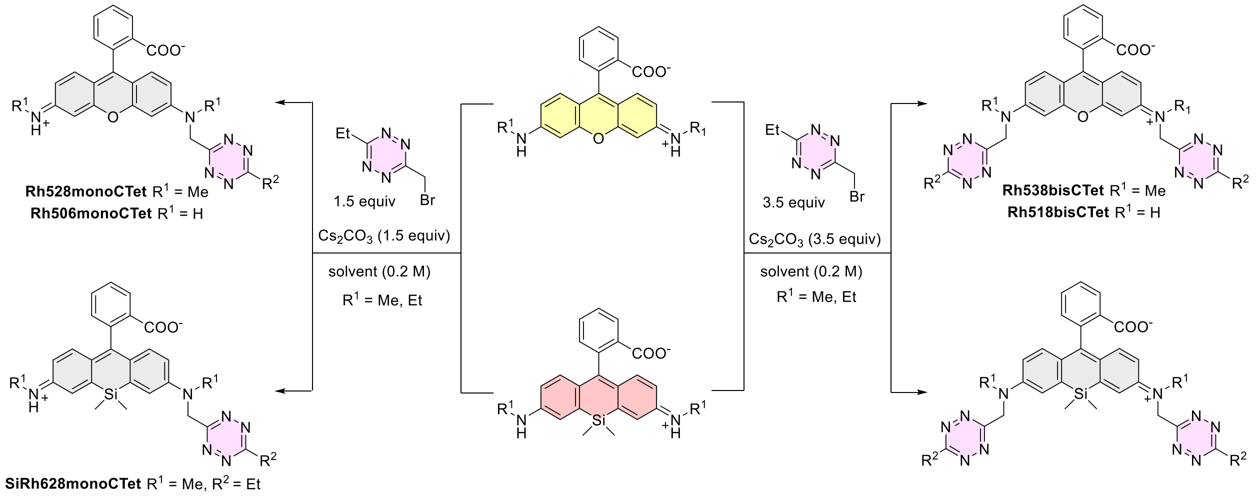

### Synthesis of 3-ethyl-6-(methoxymethyl)-1,2,4,5-tetrazine (compound 1a)

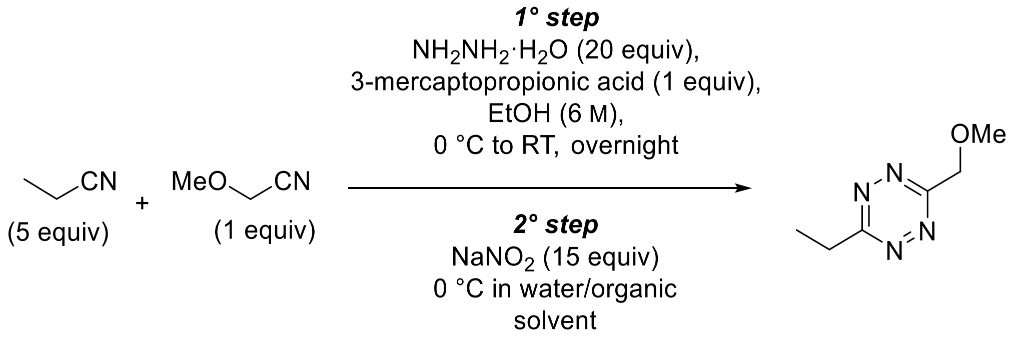


According to the literature,[4] a solution of proprionitrile (1.41 mL, 20.00 mmol, 5 equiv), methoxyacetonitrile (0.59 mL, 4.00 mmol, 1 equiv), and 3-mercaptopropionic acid (347 µL, 4.00 mmol, 1 equiv) in ethanol (0.7 mL, 6 M) was cooled to 0 °C. To this mixture was added dropwise hydrazine hydrate 65% in $H_2O$ (3.3 mL, 80 mmol, 20 equiv). The reaction mixture was stirred vigorously at RT for 18 h. Upon completion, the reaction solution was cooled with ice water, and DCM was added as cosolvent. An ice water solution of sodium nitrite (4.14 g, 60 mmol, 15 equiv) was slowly added to the reaction mixture, followed by slow addition of 1M HCl under vigorous stirring. The mixture turned bright red and gas evolved. Addition of 1M HCl was continued until gas evolution ceased (pH = 4–5). Then, the reaction mixture was extracted with DCM (3 x 40 mL). The combined organic layers were dried over anhydrous $MgSO_4$, filtered, and the solvent was removed under reduced pressure. The crude reaction mixture was purified via column chromatography (silica, n-Hex/EtOAc 4:1 to 2:1) to afford the desired **compound 1a** as a pinkish oil (530.0 mg, 3.44 mmol, 86%).

$^1$H NMR (400 MHz, $CDCl_3$) δ 5.01 (s, 2H), 3.57 (d, $J$ = 0.6 Hz, 3H), 3.36 (q, $J$ = 7.6 Hz, 2H), 1.50 (t, $J$ = 7.6 Hz, 3H).

$^{13}$C NMR (101 MHz, $CDCl_3$) δ 172.05 (s), 166.25 (s), 72.14 (t), 59.59 (q), 28.44 (t), 12.24 (q).

HRMS-ESI $m/z$ for $C_6H_{10}N_4O$: calc. 155.0927 $[M+H]^+$; found 155.0914 $[M+H]^+$ (70%).

### Synthesis of (6-ethyl-1,2,4,5-tetrazin-3-yl)methanol (compound 2a)

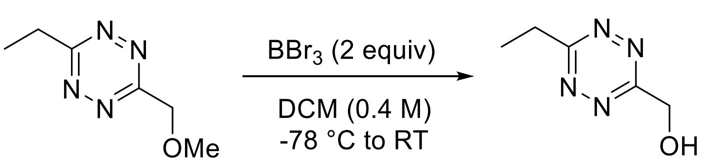


Adapted from the literature,[5] **compound 1a** (500 mg, 3.20 mmol, 1 equiv) was dissolved in DCM (8.0 mL, 6 M) and cooled to -78 °C. 1M solution of $BBr_3$ in DCM (6.40 mL, 6.40 mmol, 2 equiv) was added dropwise. The solution was stirred at -78 °C for 1 h and then warmed to RT. Upon completion of the reaction as indicated by TLC, water (2 mL) was added and the reaction was stirred vigorously at open air for 20 min. Afterwards, the aqueous phase was extracted with DCM (1 x 10 mL) and EtOAc (the formed alcohol product is rarely soluble in DCM but soluble in EtOAc). The combined organic layers were dried over $MgSO_4$ and

concentrated under reduced pressure. The crude reaction mixture was purified via column chromatography (silica, n-Hex/EtOAc 1:1) to afford the desired **compound 2a** as a pinkish oil (337.4 mg, 2.41 mmol, 75%).

$^{1}H$ NMR (400 MHz, $CDCl_3$) δ 5.29 (s, 2H), 3.40 (q, *J* = 7.6 Hz, 2H), 1.53 (t, *J* = 7.6 Hz, 3H).

$^{13}C$ NMR (101 MHz, $CDCl_3$) δ 172.30 (s), 167.59 (s), 62.70 (t), 28.46 (t), 12.30 (q).

HRMS-ESI *m/z* for $C_5H_8N_4O$: calc. 141.0771 $[M+H]^+$; found 141.0770 $[M+H]^+$ (100%).

## Synthesis of 3-(bromomethyl)-6-ethyl-1,2,4,5-tetrazine (compound 3a)

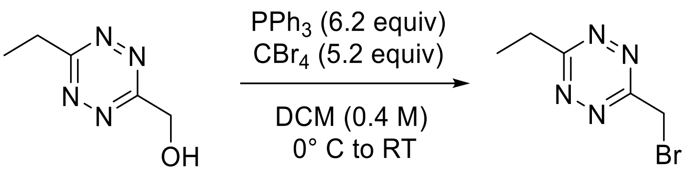


Adapted from the literature,[5] $PPh_3$ (496 mg, 1.89 mmol, 3.3 equiv) was dissolved in DCM (2 mL, 1 M) and cooled to 0 °C. $CBr_4$ (416 mg, 1.25 mmol, 2.2 equiv) was diluted in DCM (2 mL) and added dropwise. The mixture was stirred for 30 min. Then, **compound 2a** (80 mg, 0.57 mmol, 1.0 equiv), dissolved in DCM (1 mL), was added dropwise. The ice bath was removed and the reaction mixture was stirred for 1 h at RT. Subsequently, the reaction mixture was adsorbed on silica and directly subjected to flash column chromatography (dry loading). DCM was used as eluent, since the resulting brominated compound is volatile (<200mbar, 40 °C). Upon evaporation of the solvent, **compound 3a** was obtained as pinkish oil (115 mg, 0.52 mmol, 91%).

**Note:** We recommend to prepare **compound 3a** freshly and using it immediately or within one to two days, owing to its limited stability.

$^{1}H$ NMR (400 MHz, $CDCl_3$) δ 4.95 (s, 2H), 3.41 (q, *J* = 7.6 Hz, 2H), 1.55 (t, *J* = 7.6 Hz, 3H).

$^{13}C$ NMR (101 MHz, $CDCl_3$) δ 171.34 (s), 167.43 (s), 28.48 (t), 27.58 (t), 12.18 (q).

HRMS-ESI *m/z* for $C_5H_7BrN_4$: calc. 202.9927 $[M+H]^+$; found 202.9926 $[M+H]^+$ , and calc. 204.9907 $[M+H]^+$; found 204.9915 $[M+H]^+$ .

## Synthesis of 4-hydroxybutanamide (SI1)

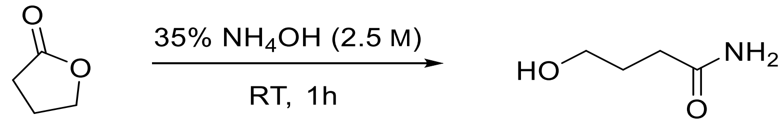


According to the literature,[6] in a round-bottom flask loaded with 35% $NH_4OH_{aq}$ (40 mL), γ-butyrolactone (7.7 mL, 8.6 g, 100 mmol, 1.0 eq) was added at rt and the resulting solution was stirred at RT for 16 h. The solvent was evaporated under reduced pressure to give the crude product. Purification by recrystallisation from hot acetone gave the corresponding amide **SI1** (7.3 g, 71 mmol, 71%) as a colorless solid.

$^1$H NMR (300 MHz, $CDCl_3$) δ 7.85 – 7.38 (m, 1H), 6.95 (s, 1H), 5.01 (s, 3H), 3.57 (s, 2H), 2.29 (s, 2H). Spectroscopic data consistent with those reported in the literature.[6]

### Synthesis of 4-((tert-butyldimethylsilyl)oxy)butanamide (SI2)

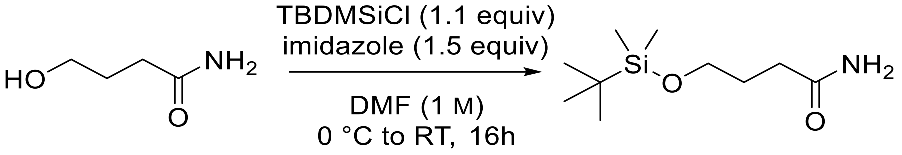


Adapted from the literature,[6] TBDMSiCl (8.2 g, 55 mmol, 1.1 eq) was added dropwise to a stirred solution of amide **SI1** (5.2 g, 50 mmol, 1.0 eq) and imidazole (5.1 g, 75 mmol, 1.5 eq) in DMF (40 mL, 1 M) at 0 °C and the resulting solution was allowed to warm to rt and stirred for 16 h. Water (40 mL) and EtOAc (40 mL) were added and the two layers were separated. The aqueous layer was extracted with EtOAc (40 mL × 2). The combined organic phases were washed with 1 M $HCl_{aq}$ (30 mL) and brine (30 mL), dried over $MgSO_4$, and evaporated under reduced pressure to give the crude product. The crude reaction mixture was purified via column chromatography (silica, n-Hex/EtOAc 1:1 to 1:9) to afford the desired product **SI2** as a colorless solid (3.72 g, 17.2 mmol, 34%).

$^1$H NMR (300 MHz, $CDCl_3$) δ 3.72 (t, *J* = 5.8 Hz, 2H), 2.45 (t, *J* = 6.7 Hz, 2H), 1.96 – 1.87 (m, 2H), 0.92 (s, 9H), 0.10 (s, 6H). Spectroscopic data consistent with those reported in the literature.[6]

### Synthesis of 4-((*tert*-butyldimethylsilyl)oxy)butanenitrile (SI3)

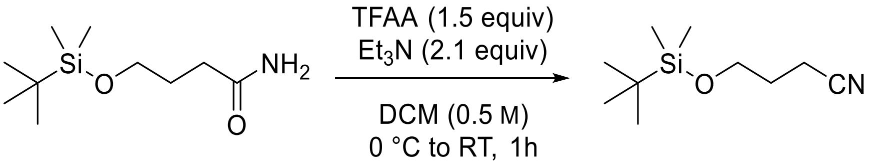


Adapted from the literature,[6] trifluoroacetic anhydride (TFAA) (1.59 mL, 2.40 g, 11.5 mmol, 2.5 eq), was added dropwise to a solution of amide **SI2** (1.0 g, 4.6 mmol, 1.0 eq) and triethylamine (0.71 mL, 0.975 g, 9.66 mmol, 2.1 eq) in DCM (10 mL, 0.5 M) at 0 °C. The resulting solution was allowed to warm to RT and stirred for 1 h. Water was added and the two layers were separated. The aqueous layer was extracted with DCM (15 mL × 3). The combined organic phases were dried over $MgSO_4$ and evaporated under reduced pressure to give the crude product. The crude reaction mixture was purified on via column chromatography (silica, n-Hex/EtOAc 9:1) to afford the desired product **SI3** as a colorless oil (0.756 g, 3.82 mmol, 83%).

$^1$H NMR (300 MHz, $CDCl_3$) δ 3.74 (t, *J* = 5.6 Hz, 2H), 2.48 (t, *J* = 7.1 Hz, 2H), 1.87 (tt, *J* = 7.1, 5.6 Hz, 2H), 0.92 (s, 8H), 0.09 (s, 6H). Spectroscopic data consistent with those reported in the literature.[7]

### Synthesis of 4-hydroxybutanenitrile (SI4)

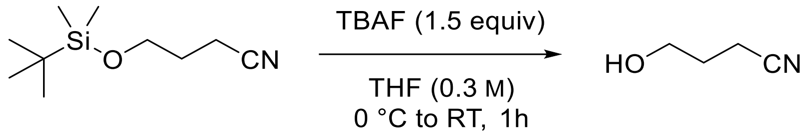


According to the literature,[6] a 1 M solution of TBAF in THF (7.8 mL, 7.8 mmol, 1.3 eq) was added dropwise to a stirred solution of nitrile **SI3** (1.2 g, 6.0 mmol, 1.0 eq) in THF (20 mL, 0.3 M) at 0 °C. The resulting solution was allowed to warm to RT and stirred for 1 h. Detection of the product via TLC was achieved by staining with $KMnO_4$ or MolybD, followed by heating. Saturated $NH_4Cl_{aq}$ was added and the two layers were separated. The aqueous layer was extracted with EtOAc (30 mL × 3). The combined organic phases were dried over $MgSO_4$ and evaporated under reduced pressure to give the crude product. The crude reaction mixture was purified via column chromatography (silica, DCM to DCM/MeOH 20:1) to afford the desired product **SI4** as a colorless oil (445 g, 5.2 mmol, 84%).

$^1$H NMR (300 MHz, $CDCl_3$) δ 3.81 (t, *J* = 5.8 Hz, 2H), 2.53 (t, *J* = 7.1 Hz, 2H), 1.93 (t, *J* = 5.9 Hz, 2H). Spectroscopic data consistent with those reported in the literature.[8]

### Synthesis of 3-(6-methyl-1,2,4,5-tetrazin-3-yl)propan-1-ol (compound 2b)

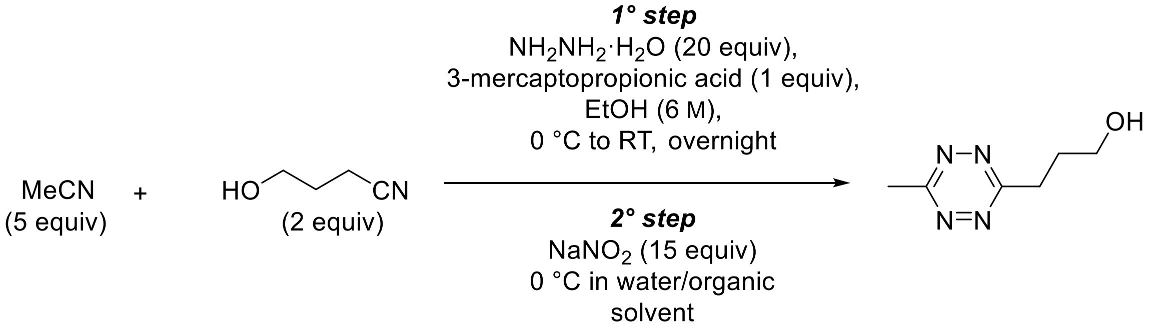


Adapted from literature,[4] a solution of acetonitrile (662 µL, 12.9 mmol, 5 equiv), **SI4** (440 mg, 5.17 mmol, 1 equiv), and 3-mercaptopropionic acid (224 µL, 2.58 mmol, 1 equiv) in ethanol (0.43 mL, 6 M) was cooled to 0 °C. To this mixture was added dropwise hydrazine hydrate 65% in $H_2O$ (2.1 mL, 51.6 mmol, 20 equiv). The reaction mixture was stirred vigorously at RT for 18 h. Upon completion, the reaction solution was cooled with ice water, and DCM was added as cosolvent. An ice-cold aqueous solution of sodium nitrite (2.66 g, 38.7 mmol, 15 equiv) was slowly added to the reaction mixture, followed by the slow addition of 1m HCl under vigorous stirring. the solution turned bright red and gas evolved. Addition of 1 M HCl continued until gas evolution ceased (pH = 4-5). Then, the reaction mixture was extracted with EtOAc (3 x 40 mL). The combined organic phases were dried over anhydrous $MgSO_4$, filtered, and the solvent was removed under reduced pressure. The crude reaction mixture was purified via column chromatography (silica, DCM to DCM/MeOH 20:1) to afford the desired **compound 2b** as a pinkish oil (374 mg, 2.42 mmol, 47%).

$^{1}H$ NMR (400 MHz, $CDCl_3$) δ 3.82 (t, $J$ = 6.1 Hz, 2H), 3.45 (t, $J$ = 7.4 Hz, 2H), 3.06 (s, 3H), 2.24 (tt, $J$ = 7.3, 6.1 Hz, 2H).

$^{13}C$ NMR (101 MHz, $CDCl_3$) δ 169.83 (s), 167.42 (s), 61.74 (t), 31.42 (t), 30.65 (t), 21.10 (q).

HRMS-ESI *m/z* for: calc. 155.0927 $[M+H]^+$; found 155.0928 $[M+H]^+$ .

### Synthesis of 3-(3-bromopropyl)-6-methyl-1,2,4,5-tetrazine (compound 3b)

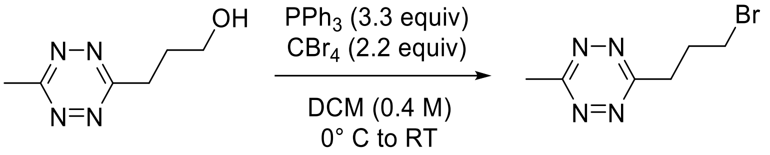


Adapted from the literature,[5] $PPh_3$ (496 mg, 1.89 mmol, 3.3 equiv) was dissolved in DCM (2 mL, 1 M) and cooled to 0 °C. $CBr_4$ (416 mg, 1.25 mmol, 2.2 equiv) was diluted in DCM (2 mL) and added dropwise. The reaction mixture was stirred for 30 min at the same temperature. Then substrate **2b** (90 mg, 0.58 mmol, 1.0 equiv) was dissolved in DCM (1 mL) and added dropwise. The ice bath was removed after complete addition and the reaction mixture was stirred for 1 h at RT. Subsequently it was adsorbed on silica and was purified by flash column chromatography, DCM was used as the only eluent, since the resulting brominated compound could be volatile. After evaporation, the **compound 3b** was obtained as pink liquid (103 mg, 0.48 mmol, 83%).

$^{1}H$ NMR (400 MHz, $CDCl_3$) δ 3.58 (t, $J$ = 6.5 Hz, 2H), 3.57 – 3.45 (m, 2H), 3.07 (s, 3H), 2.61 – 2.49 (m, 2H).

$^{13}C$ NMR (101 MHz, $CDCl_3$) δ 168.87 (s), 167.64 (s), 33.21 (t), 32.22 (t), 30.37 (t), 21.13 (q).

HRMS-ESI *m/z* for: calc. 416.0717 $[M+H]^+$; found 416.0719 $[M+H]^+$, calc. 418.0698 $[M+H]^+$; found 418.0703 $[M+H]^+$.

### Synthesis of 3-(methoxymethyl)-6-phenyl-1,2,4,5-tetrazine (compound 1c)

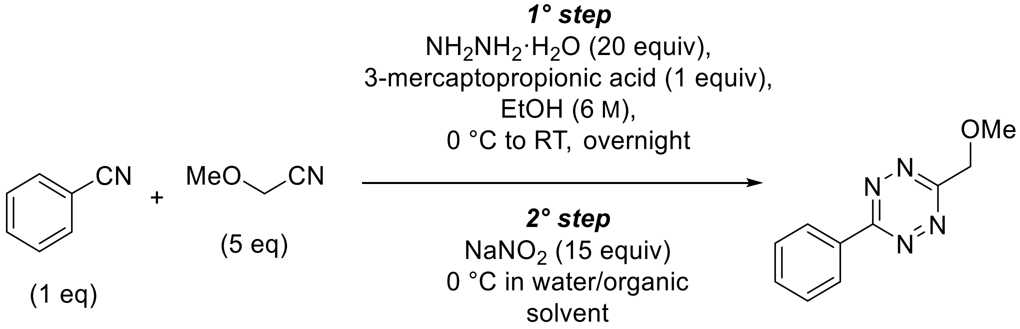


According to the literature,[4] a solution of benzonitrile (407 µL, 4.00 mmol, 1 equiv), methoxyacetonitrile (742 µL, 20.00 mmol, 5 equiv), and 3-mercaptopropionic acid (173 µL, 2.00 mmol, 0.5 equiv) in ethanol (0.7 mL, 6 M) was cooled to 0 °C. To this mixture was added dropwise hydrazine hydrate 65% in $H_2O$ (3.3

mL, 80 mmol, 20 equiv). The reaction mixture was stirred vigorously at RT for 18 h. Upon completion, the reaction solution was cooled with ice water, and DCM was added as cosolvent. An ice-cold water solution of sodium nitrite (4.14 g, 60 mmol, 15 equiv) was slowly added to the reaction mixture, followed by slow addition of 1 M HCl during which the solution was stirred intensely and turned bright red, and gas evolution was observed. Addition of 1 M HCl continued until gas evolution ceased and the pH value was 4-5. Then, the reaction mixture was extracted with DCM (3 x 40 mL). The combined organic layers were dried over anhydrous $MgSO_4$, filtered, and the solvent was removed under reduced pressure. The crude reaction mixture was purified via column chromatography (silica, n-Hex/EtOAc 3:1) to afford the desired **compound 1c** as a pink solid (646 mg, 3.2 mmol, 80%).

$^1H$ NMR (300 MHz, $CDCl_3$) δ 8.87 – 8.33 (m, 2H), 7.74 – 7.57 (m, 3H), 5.13 (s, 2H), 3.67 (s, 3H).

### Synthesis of (6-phenyl-1,2,4,5-tetrazin-3-yl)methanol (compound 2c)

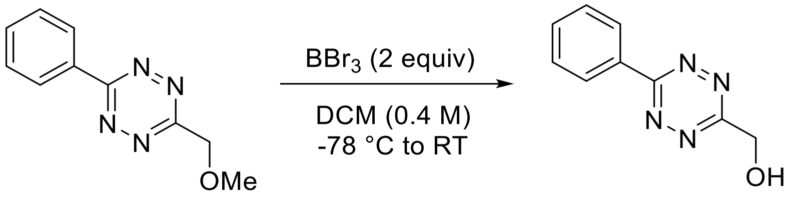


Adapted from the literature,[5] **compound 1c** (323 mg, 1.6 mmol, 1 equiv) was dissolved in DCM (8.0 mL, 6 M) and cooled to -78 °C. 1 M solution of $BBr_3$ in DCM (3.20 mL, 3.20 mmol, 2 equiv) was added dropwise. The solution was stirred at -78 °C for 1 h and then warmed to RT, once reached RT, the reaction was monitored via TLC to check if all the starting material has been consumed. Then water (2 mL) was added, and the reaction was stirred vigorously at open air for 20 min. Afterwards the aqueous layer was extracted with DCM (3 x 10 mL). Combined organic layers were dried over $MgSO_4$ and concentrated under reduced pressure. The crude reaction mixture was purified on column chromatography (silica, n-Hex/EtOAc 3:1 to 1:1) to afford the desired **compound 2c** as a pink solid (217 mg, 1.15 mmol, 72%).

$^1H$ NMR (300 MHz, $CDCl_3$) δ 8.71 – 8.60 (m, 2H), 7.75 – 7.58 (m, 3H), 5.36 (s, 2H).

$^{13}C$ NMR (101 MHz, $CDCl_3$) δ 167.39 (s), 165.38 (s), 133.03 (d), 131.41 (s), 129.37 (d), 128.22 (d), 62.82 (t).

HRMS-ESI *m/z* for $C_9H_8N_4O$: calc. 188.0698 $[M+H]^+$; found 189.0771 $[M+H]^+$ (100%).

### Synthesis of 3-(bromomethyl)-6-phenyl-1,2,4,5-tetrazine (compound 3c)

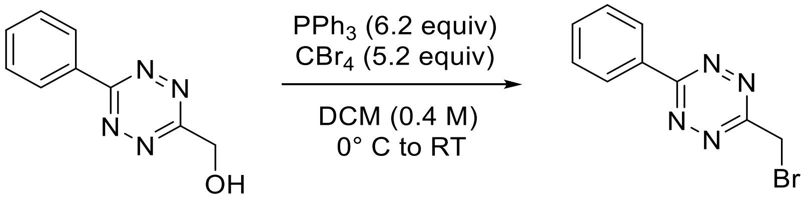

Adapted from the literature,[5] $PPh_3$ (496 mg, 1.89 mmol, 3.3 equiv) was dissolved in DCM (2 mL, 1 M) and cooled to 0 °C. $CBr_4$ (416 mg, 1.25 mmol, 2.2 equiv) was diluted in DCM (2 mL) and added dropwise. The reaction mixture was stirred for 30 min. Then, **compound 2c** (107 mg, 0.57 mmol, 1.0 equiv) was dissolved in DCM (1 mL) and added dropwise. The ice bath was removed, and the reaction mixture was stirred for 1 h at RT. Subsequently, the reaction mixture was loaded on silica and purified via column chromatography (silica, 100% DCM) to afford the desired **compound 3c** as a pink solid (126.3 mg, 0.51 mmol, 89%).

$^1$H NMR (300 MHz, $CDCl_3$) δ 8.91 – 8.35 (m, 1H), 8.03 – 7.49 (m, 1H), 5.02 (s, 1H).

$^{13}$C NMR (101 MHz, $CDCl_3$) δ 167.29 (s), 164.12 (s), 133.28 (d), 131.21 (s), 129.42 (d), 128.47 (d), 27.65 (t).

**Note**: This synthetic approach can be extended to generate a toolbox of various functionalized bromomethyl tetrazine derivatives. However, the use of **compound 3c** in combination with **compound 11** yields a poorly water-soluble compound. For this reason, no further investigations using **compound 3c** have been undertaken.

### Synthesis of 3-(methylamino)phenol (compound 4)

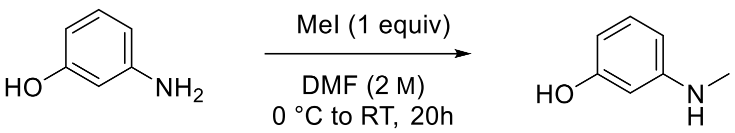


In a round-bottom flask equipped with a condenser, 3-aminophenol (4.0 g, 37.0 mmol, 1 equiv) was dissolved in DMF (20 mL, 2 M), followed by the addition of potassium carbonate (5.1 g, 36.0 mmol, 1 equiv). The reaction was cooled at 0 °C and methyl iodide (2.2 mL, 36.2 mmol, 0.98 equiv) was added dropwise. The reaction was heated to 100 °C and stirred for 2 hours at the same temperature.

After the reaction was completed (TLC monitoring), the mixture was quenched with water (15 mL) and extracted with EtOAc (4 x 20 mL). The combined organic layers were washed successively with water (20 mL) and brine (2 x 20 mL), and then dried over $MgSO_4$. The filtered solution was concentrated under reduced pressure, and the crude residue was purified by flash chromatography (silica, 4:1 n-Hex:EtOAc) to provide *N*-methyl-3-aminophenol, **compound 4**, as a white solid (3.5 g, 28.0 mmol, 77%).

$^1$H NMR (300 MHz, $CDCl_3$) δ 7.05 (t, $J$ = 8.0 Hz, 1H), 6.32 – 6.08 (m, 3H), 2.84 (s, 3H).

Spectroscopic data are consistent with those reported in the literature.[9]

## Synthesis of 2-(6-(methylamino)-3-(methyliminio)-3H-xanthen-9-yl)benzoate (compound 5a)

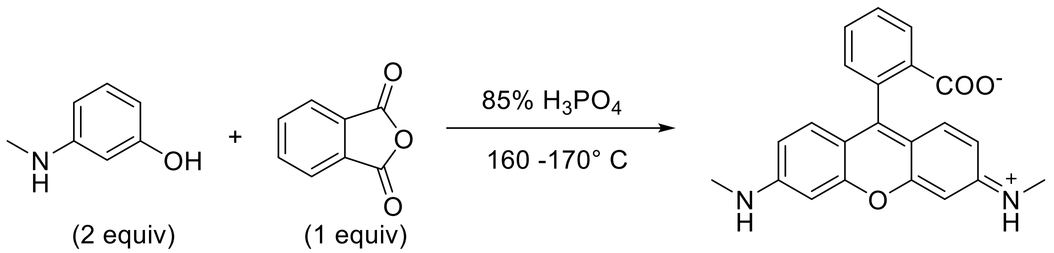


According to the literature,[10] a mixture of powdered phthalic anhydride (1.23 g, 8.31 mmol) and **compound 4** (1.08 g, 5.65 mmol) was heated with stirring under Ar at 160–170 °C for 3 h. Then the second portion of **compound 4** (0.86 g, 4.51 mmol) and 5 mL of 85% $H_3PO_4$ were added, and the heating at 160–170°C was continued for additional 3 h. After cooling to RT, MeOH (10 mL) and water (2 mL) were added, and the mixture was stirred at room temperature overnight. The red solid was filtrated and recrystallized from MeOH to yield **compound 5a** (686 mg, 1.9 mmol, 23%) as a bright red solid.

**Note**: If precipitation does not occur, even upon cooling, purify via reverse-phase column chromatography (Biotage, C18 packed column).

$^1$H NMR (600 MHz, $CD_3CN$) δ 8.03 (d, *J* = 7.7 Hz, 1H), 7.76 (td, *J* = 7.5, 1.2 Hz, 1H), 7.70 (td, *J* = 7.5, 1.1 Hz, 1H), 7.23 (dt, *J* = 7.6, 0.9 Hz, 1H), 6.61 (d, *J* = 8.7 Hz, 2H), 6.48 – 6.41 (m, 4H), 5.41 – 5.05 (br s, 2H), 2.85 (s, 6H).

HRMS-ESI *m/z* for: calc. 359.1384 $[M+H]^+$; found 359.1390 $[M+H]^+$.

Spectroscopic data are consistent with those reported in the literature.[10]

## Synthesis of 2-(6-amino-3-iminio-3H-xanthen-9-yl)benzoate (compound 5b)

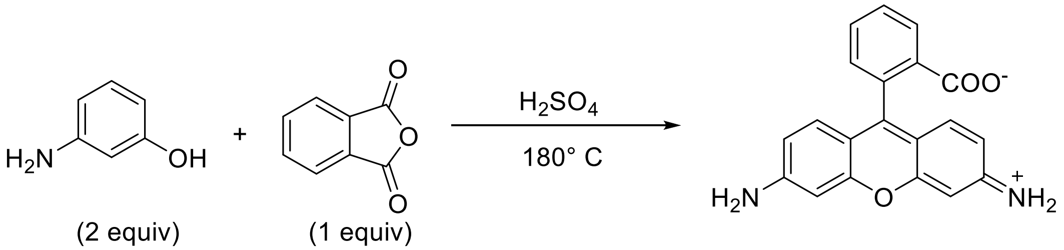


According to the literature,[11] phthalic anhydride (2.5 g, 16.7 mmol, 1.0 equiv) and 3-aminophenol (3.12 g, 28.6 mmol, 1.7 equiv) were suspended in conc. sulfuric acid (12.5 mL) and heated to 180° C for 5 hours. After cooling to RT, the viscous black liquid was carefully added to crushed ice (50 g) and divided into 2 centrifuge tubes. The brownish solid was separately washed with water (7.5 mL) followed by addition of water (10 mL) and conc. ammonia solution (2.5 mL). This mixture was allowed to stand for 12 hours at 4 °C. After centrifugation the solid (about 7 g) was combined and refluxed for 5 minutes in hydrochloric acid

(200 mL, 0.3 M) and hot filtrated. The crude product was allowed to stand for 48 hours at 4 °C. The brownish-red solid filtered and dried to yield **compound 5b** (1.27 g, 3.8 mmol, 23%)

Spectroscopic data are consistent with those reported in the literature.[12]

$^1$H NMR (400 MHz, DMSO) δ 7.97 – 7.91 (m, 1H), 7.78 (td, *J* = 7.5, 1.2 Hz, 1H), 7.68 (td, *J* = 7.4, 1.0 Hz, 1H), 7.23 (dt, *J* = 7.7, 0.9 Hz, 1H), 6.39 (d, *J* = 1.9 Hz, 2H), 6.36 – 6.17 (m, 4H), 5.60 (s, 4H).

$^{13}$C NMR (101 MHz, DMSO) δ 169.39 (s), 166.01 (s), 152.98 (s), 152.71 (s), 151.58 (s), 151.52 (s), 135.69 (d), 130.18 (d), 128.87 (d), 127.32 (s), 124.80 (d), 124.50 (d), 111.24 (d), 106.37 (s), 99.63 (d), 85.64 (s).

HRMS-ESI *m/z* for: calc. 331.1078 $[M+H]^+$; found 331.1077 $[M+H]^+$.

## Synthesis of *tert*-butyl 3-bromobenzoate (compound 6)

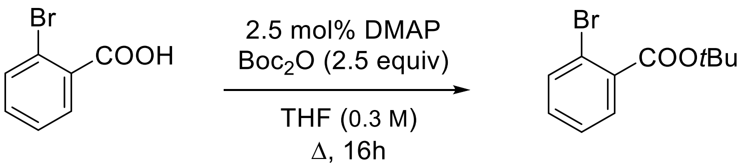


According to the literature,[13] 3-bromo-4-methylbenzoic acid (2.0 g, 10 mmol, 1 equiv), $Boc_2O$ (5.88 g, 27 mmol, 2.7 equiv) and DMAP (30.0 mg, 0.25 mmol, 2.5 mol%) were dissolved in anhydrous THF (33 mL, 0.3 M). The solution was refluxed overnight, then allowed to cool to RT, and evaporated to dryness. The residue was dissolved in EtOAc. The resulting solution was washed with sat. $NaHCO_3$ aq. and brine, dried over anhydrous $MgSO_4$, and filtered. The filtrate was evaporated to dryness, and the residue was purified by column chromatography (silica, n-Hex to n-Hex:EtOAc 100:1) to afford *tert*-butyl 3-bromo-4-methylbenzoate (2.4 g, 9.3 mmol, 93%).

$^1$H NMR (300 MHz, $CDCl_3$) δ 7.71 (dd, *J* = 7.3, 2.2 Hz, 1H), 7.64 (dd, *J* = 7.9, 1.4 Hz, 1H), 7.36 (td, *J* = 7.5, 1.5 Hz, 1H), 7.30 (td, *J* = 7.6, 2.0 Hz, 2H), 1.64 (s, 9H).

Spectroscopic data consistent with those reported in the literature.[14]

## Synthesis of 3-bromo-*N*-methylaniline

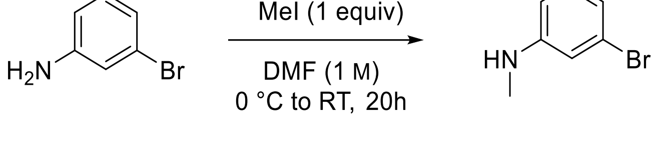


According to the literature,[15] to a stirred solution of 3-bromoaniline (5.67 mL, 52.2 mmol, 1.0 equiv) in DMF (55 mL) was added potassium hydroxide (3.23 g, 57.6 mmol, 1.1 equiv), followed by the dropwise addition of methyl iodide (3.25 mL, 52.2 mmol, 1.0 equiv). After stirring for 20 h at RT, the reaction mixture was quenched with water (30 mL) and extracted with EtOAc (4 x 30 mL). The combined organic layers were washed successively with water (20 mL) and brine (2 x 20 mL) and then dried over $MgSO_4$. The filtered solution was concentrated under reduced pressure, and the crude residue was purified by column

chromatography (silica, n-Hex to n-Hex:EtOAc 100:1) to afford *N*-methyl-3-bromoaniline (4.05 g, 21.6 mmol, 41% yield) as a pale-yellow oil. Spectroscopic data consistent with those reported in the literature.[16]

### Synthesis of *N*-allyl-3-bromo-*N*-methylaniline (compound 7)

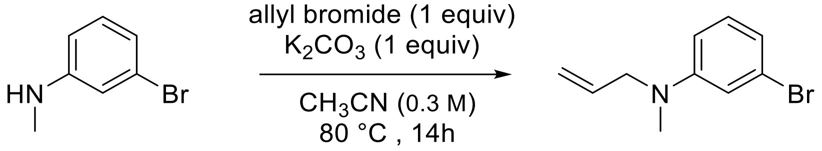


To suspension of $K_2CO_3$ (2.90 g, 21 mmol, 1.0 equiv) in $CH_3CN$, *N*-methyl 3-bromo-aniline was added (4.0 g, 21 mmol, 1.0 equiv). The reaction was cooled to 0 °C and allyl bromide (2.7 mL, 31.5 mmol, 1.5 equiv) was added dropwise and the mixture stirred at 80 °C for 14 h. After cooling, the reaction mixture was filtered through a celite pad, washed with EtOAc and evaporated to dryness. The residue was purified by column chromatography (silica, n-Hex:EtOAc 40:1) to afford **compound 7** (3.9 g, 17.2 mmol, 82% yield) as an oil. For spectroscopic data refer to those reported in the literature.[17]

### Synthesis of 4,4'-methylenebis(*N*-allyl-3-bromo-*N*-methylaniline) (compound 8)

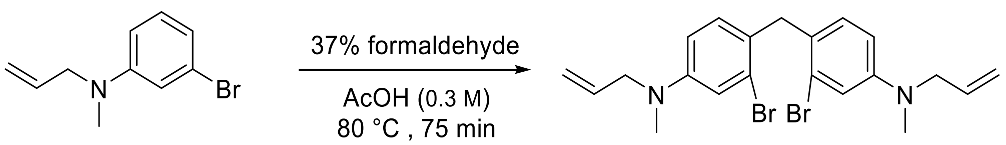


Adapted from literature,[18] to a solution of **compound 7** (3.8 g, 16.7 mmol, 1 equiv) in AcOH (50 mL, 0.3 M) was added 37% formaldehyde (2.4 mL), and the mixture was stirred at 80 °C for 75 min. After cooling to RT, the reaction mixture was neutralized with saturated $NaHCO_3$ aq. and NaOH aq. and extracted with DCM. The organic layer was washed with brine, dried over $Mg_2SO_4$ and concentrated under reduced pressure. The crude reaction mixture was purified on column chromatography (silica, n-Hex/EtOAc 30:1) to afford the desired product **compound 8** as a colorless oil (3.1 g, 6.5 mmol, 77%). For spectroscopic data refer to those reported in the literature.[17]

### Synthesis of 3,7-bis(allyl(methyl)amino)-5,5-dimethyldibenzo[*b,e*]silin-10(5H)-one (compound 9)

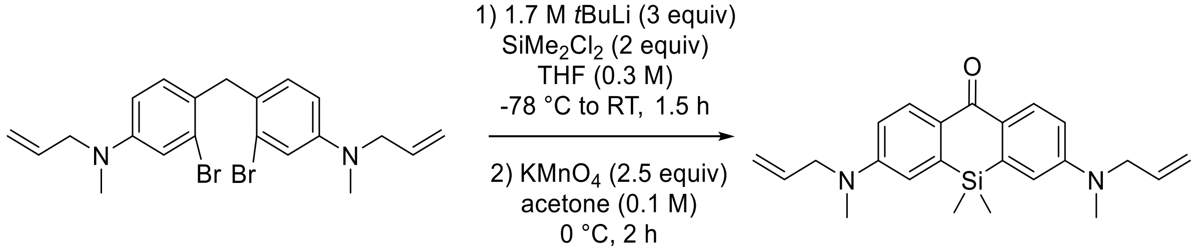

Adapted from literature,[17] to a flame-dried flask flushed with argon, **compound 8** (2.6 g, 5.6 mmol, 1 equiv) and anhydrous THF (20 mL, 0.3 M) were added. The solution was cooled to –78 °C, 1.7 M *t*BuLi (10.0 mL, 20 mmol, 3 equiv) was added, and the mixture was stirred for 30 min. At the same temperature, a solution of $SiMe_2Cl_2$ (1.35mL, 11.2 mmol, 2 equiv) in anhydrous THF (6 mL) was slowly added, afterwards the mixture was warmed to room temperature and stirred for 1 h. The reaction was quenched by addition of water and saturated $NH_4Cl$ (keep the pH around 5-6). The mixture was neutralized with sat. aq. $NaHCO_3$ and extracted with DCM. The organic layer was washed with brine, dried over $Mg_2SO_4$, and concentrated under reduced pressure. The residue was dissolved in acetone (56 mL, 0.1 m), and the solution was cooled to 0 °C. To this solution, $KMnO_4$ (2.2 g, 14.0 mmol, 2.5 equiv) was added in small portions over a period of 2 h while stirring. The mixture was stirred for another 1 h, keeping the temperature at 0 °C, then diluted with DCM (100 mL), filtered under vacuum through paper filter. The filtrate was concentrated under reduced pressure. The crude reaction mixture was purified via column chromatography (silica, 100% DCM) to afford the desired product **compound 9** as a yellowish oil (673 mg, 1.78 mmol, 31%).[17]

**Note:** the silylation step is quite critical, be careful to any water content as well as the pH after quenching the reaction and during the work up.

$^1$H NMR (400 MHz, $CDCl_3$) δ 8.55 – 8.31 (m, 2H), 6.98 – 6.74 (m, 4H), 5.89 (ddt, *J* = 17.0, 10.2, 5.0 Hz, 2H), 5.34 – 5.10 (m, 4H), 4.06 (dt, *J* = 5.1, 1.8 Hz, 4H), 3.11 (s, 6H), 0.47 (s, 6H).

$^{13}$C NMR (101 MHz, $CDCl_3$) δ 185.11, 150.38, 140.57, 132.47, 131.67, 130.17, 129.81, 116.94, 114.89, 113.60, 54.99, 38.23, -1.09.

HRMS-ESI *m/z* for $C_{23}H_{28}N_2OSi$: calc. 377.2044 $[M+H]^+$; found 377.2039 $[M+H]^+$.

### Synthesis of 3,7-bis(allyl(methyl)amino)-5,5-dimethyl-3'H,5H-spiro[dibenzo[*b*,*e*]siline-10,1'-isobenzofuran]-3'-one (compound 10)

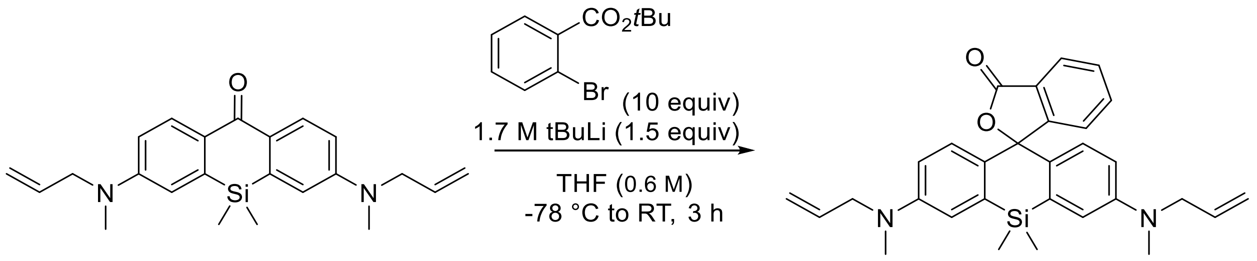


In a flame-dried Schlenk flask flushed with argon *tert*-Butyl 2-bromo-benzoate (627 mg, 2.31 mmol) was dissolved in anhydrous THF (5.0 mL). The solution was cooled to –78 °C, and 1.7 M *t*BuLi in *n*-Hex (1.2 mL, 2 mmol, 1.5 equiv) was added dropwise. The mixture was stirred for 35 min at the same temperature. Then, **compound 9** (86.0 mg, 0.223 mmol) dissolved in anhydrous THF (12.0 mL) was added slowly. The reaction mixture was warmed to room temperature and stirred for 2.5 h. Then, 2N HCl aq. was added and stirred for 20 min. The reaction was neutralized with sat. aq. $NaHCO_3$ to obtain the closed spirolactone form. The aqueous phase was extracted with DCM. The organic layer was washed with brine, dried over $Mg_2SO_4$ and evaporated in vacuo. The crude reaction mixture was purified on silica gel column

chromatography (nHex/EtOAc/DCM 6:1:1) to afford the desired **compound 10** as a colorless oil (56 mg, 0.116 mmol, 52%).

$^1$H NMR (600 MHz, $CD_3CN$) δ 8.01 (d, *J* = 7.8 Hz, 1H), 7.75 (dd, *J* = 7.5, 1.2 Hz, 1H), 7.66 (dd, *J* = 7.5, 1.0 Hz, 1H), 7.27 (dt, *J* = 7.7, 0.9 Hz, 1H), 7.15 (d, *J* = 2.9 Hz, 2H), 6.80 (d, *J* = 9.1 Hz, 2H), 6.67 (dd, *J* = 9.1, 2.9 Hz, 2H), 5.87 (ddt, *J* = 17.1, 10.3, 5.1 Hz, 2H), 5.28 – 5.02 (m, 4H), 4.16 – 3.89 (m, 4H), 3.06 (s, 6H), 0.63 (s, 3H), 0.53 (s, 3H).

$^{13}$C NMR (151 MHz, $CD_3CN$) δ 169.21, 159.33, 159.09, 149.31, 134.33, 133.95, 132.87, 130.93, 129.11, 128.93, 128.19, 126.85, 125.28, 122.47, 117.38, 116.20, 115.02, 114.03, 54.63, 37.95, -1.09, -2.41.

HRMS-ESI *m/z* for $C_{28}H_{31}N_5OSi$: calc. 481.2292 $[M+H]^+$; found 481.2296 $[M+H]^+$.

### Synthesis of 5,5-dimethyl-3,7-bis(methylamino)-3'H,5H-spiro[dibenzo[*b,e*]siline-10,1'-isobenzofuran]-3'-one (compound 11)

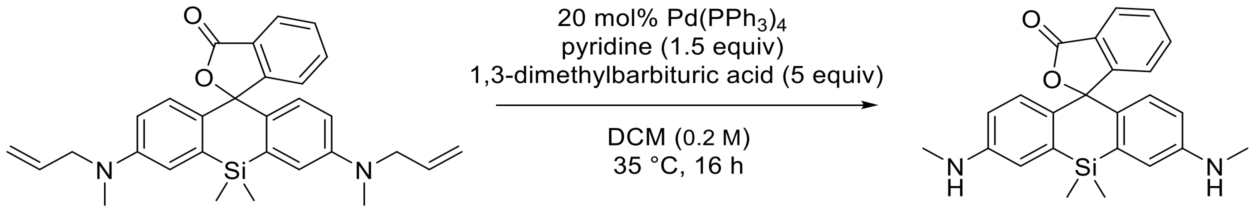


In a flame-dried Schlenk-flask flushed with argon, $Pd(PPh_3)_4$ (53 mg, 0.046 mmol, 20 mol%) and pyridine (27 µl, 0.35 mmol, 1.5 eq) were added. **Compound 10** (109 mg, 0.23 mmol, 1 equiv) dissolved in DCM (10 mL) was added, followed by 1,3-dimethylbarbituric acid (179 mg, 1.1 mmol, 5.0 equiv). The reaction was stirred at 35° C for 24 h, since the reaction was not complete after 8 hours, another 10 mol% $Pd(PPh_3)_4$ was added. Once the reaction was completed, it was evaporated to dryness. The residue was suspended in saturated $Na_2CO_3$ aq. and extracted with DCM. The organic layer was washed with brine, dried over $MgSO_4$ and evaporated in vacuo. Purification via preparative reverse-phase HPLC (Phase A: water 0.1% TFA, Phase B: $CH_3CN$; Phase B 5% to 90%) afforded the title **compound 11** as a blue solid and trifluoroacetate salt (47 mg, 0.10 mmol, 40%). Alternatively, the title compound was also purified via column chromatography (silica, DCM/MeOH 200:1 to 100:1).

$^1$H NMR (600 MHz, $CD_3CN$) δ 7.93 (dt, *J* = 7.7, 1.0 Hz, 1H), 7.73 (td, *J* = 7.5, 1.1 Hz, 2H), 7.63 (td, *J* = 7.5, 0.9 Hz, 1H), 7.26 (dt, *J* = 7.7, 0.9 Hz, 1H), 6.94 (d, *J* = 2.7 Hz, 2H), 6.68 (d, *J* = 8.7 Hz, 2H), 6.49 (dd, *J* = 8.7, 2.7 Hz, 2H), 2.78 (s, 6H), 0.61 (s, 3H), 0.53 (s, 3H).

$^{13}$C NMR (151 MHz, $CD_3CN$) δ 170.32 (s), 161.80(s), 154.85 (s), 149.10 (s), 136.46 (s), 134.30 (d), 131.66 (s), 128.99 (d), 128.14 (d), 126.13 (s), 125.24 (d), 124.27 (d), 117.38 (s), 116.44 (d), 113.22 (d), 29.35 (t), -1.05 (q), -2.38 (q).

HRMS-ESI *m/z* for $C_{24}H_{24}N_2O_2Si$: calc. 401.1680 $[M+H]^+$; found 401.1671 $[M+H]^+$.

## Synthesis of 3'-amino-6'-(((6-ethyl-1,2,4,5-tetrazin-3-yl)methyl)amino)-3H-spiro[isobenzofuran-1,9'-xanthen]-3-one (Rh506monoCTet)

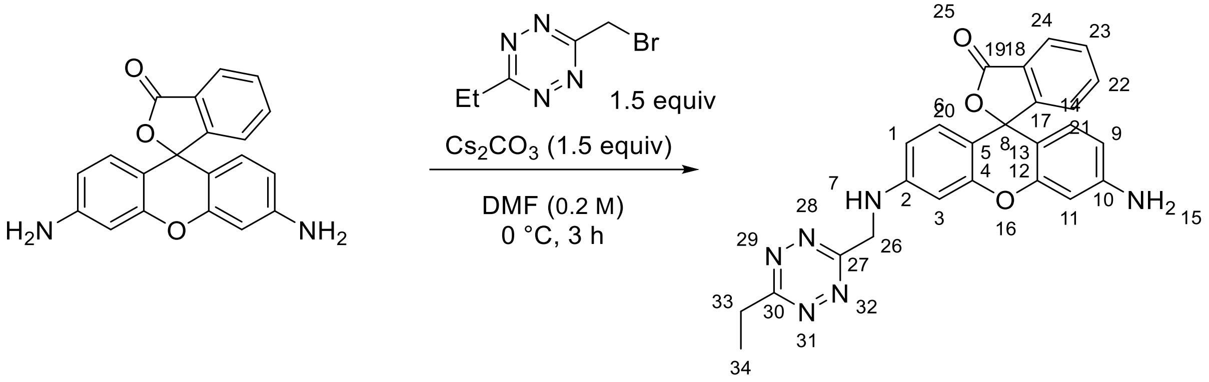


In a round bottom flask charged with 4 Å molecular sieves under Argon, **5b** (20 mg, 60 µmol, 1 equiv) was dissolved in anhydrous dimethylformamide (0.2 mL, 0.2 M) and cooled down to 0 °C. Then, $Cs_2CO_3$ (29.2 mg, 90 µmol, 1.5 equiv) was added at 0 °C. After 5 min, **3a** (18.2 mg, 90.0 µmol, 1.5 equiv), dissolved in anhydrous dimethylformamide (0.1 mL), was added dropwise. The resulting mixture was stirred for 3 h at < 15 °C. Conversion of **5b** to the resulting product was monitored by LCMS. Longer reactions times and higher temperatures led to the degradation of the product, due to the instability of the tetrazine under basic conditions. Next, the reaction was quenched with 3 ml of $CH_3CN$ (0.1 % TFA), loaded onto a C18 column (Biotage Sfär C18D, 12g) and subjected to crude purification and desalting by reverse-phase Biotage purification using phase A (water with 0.1% TFA) and phase B ($CH_3CN$) under a gradient of 5-90% phase B. The crude mixture could also be purified on column chromatography, (silica, DCM/MeOH 20:1 to 4:1), by replacing $CH_3CN$ with 3 mL of DCM containing 0.1% TFA; however, this approach resulted in lower yields. As the final purification step, the product was purified via preparative reverse-phase HPLC (Phase A: water 0.1% TFA, Phase B: $CH_3CN$; Phase B 5% to 80%) afforded the title product **Rh506monoCTet** as a bright red solid and as TFA salt (3.4 mg, 6 µmol, 9%).

$^{1}$H NMR (600 MHz, MeOD) δ 8.35 (dd, *J* = 7.9, 1.3 Hz, 1H, H-24), 7.86 (td, *J* = 7.5, 1.4 Hz, 1H, H-22), 7.81 (td, *J* = 7.7, 1.3 Hz, 1H, H-23), 7.41 (dd, *J* = 7.5, 1.2 Hz, 1H, H-21), 7.13 – 7.09 (m, 2H), 7.06 (d, *J* = 2.2 Hz, 1H), 7.00 (dd, *J* = 9.2, 2.2 Hz, 1H), 6.87 (d, *J* = 2.1 Hz, 1H), 6.85 (s, 1H), 5.25 (s, 2H, H-26), 3.33 (p, *J* = 1.7 Hz, 2H, H-33), 1.51 (t, *J* = 7.6 Hz, 3H, H-34). In MeOD as open zwitterionic form.

$^{13}$C NMR (151 MHz, MeOD) δ 171.89 (s), 166.77 (s), 166.36 (s), 161.36 (s), 160.41 (s), 158.68 (s), 157.74 (s), 133.85 (s), 132.46 (d), 131.69 (d), 131.12 (d), 130.84 (s), 130.08 (d), 129.97 (d), 117.06 (d), 114.18 (s), 113.98 (s), 96.89 (d), 45.32 (t), 27.85 (t), 10.92 (q).

HRMS-ESI *m/z* for: calc. 453.1670 $[M+H]^+$; found 453.1675 $[M+H]^+$ .

**Synthesis of 3',6'-bis(((6-ethyl-1,2,4,5-tetrazin-3-yl)methyl)amino)-3H-spiro[isobenzofuran-1,9'-xanthen]-3-one (Rh518bisCTet)**

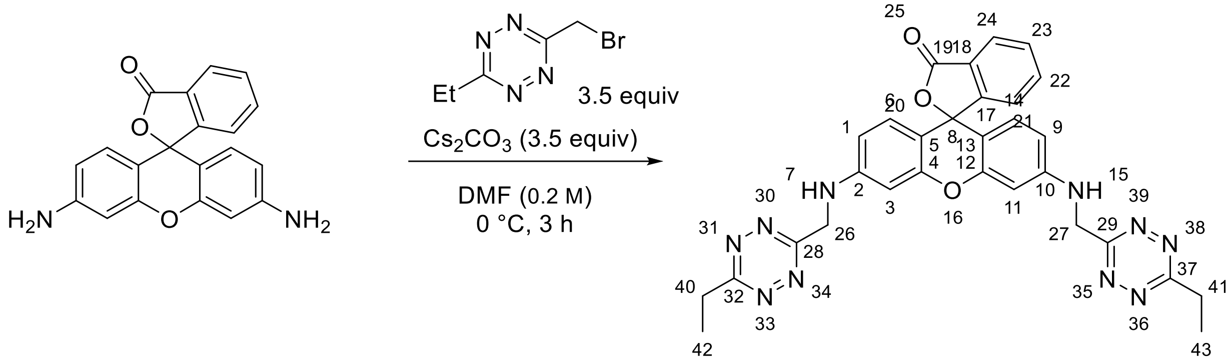


In a round bottom flask charged with 4 Å molecular sieves under Argon, **5b** (12 mg, 37 µmol, 1 equiv) was dissolved in anhydrous dimethylformamide (0.1 mL, 0.2 M) and cooled down to 0 °C. Then, $Cs_2CO_3$ (42 mg, 129 µmol, 3.5 equiv) was added at 0 °C. After 5 min, **3a** (26 mg, 129 µmol, 3.5 equiv), dissolved in anhydrous dimethylformamide (0.1 mL), was added dropwise. The resulting mixture was stirred for 3 h at < 15 °C. Conversion of **5b** to the resulting product was monitored by LCMS. Longer reactions times and higher temperatures led to the degradation of the product, due to the instability of the tetrazine under basic conditions. Next, the reaction was quenched with 3 ml of $CH_3CN$ (0.1 % TFA), loaded onto a C18 column (Biotage Sfär C18D, 12g) and subjected to crude purification and desalting by reverse-phase Biotage purification using phase A (water with 0.1% TFA) and phase B ($CH_3CN$) under a gradient of 5-90% phase B. The crude mixture could also be purified on column chromatography (silica, DCM/MeOH 20:1 to 6:1), by replacing $CH_3CN$ with 3 mL of DCM containing 0.1% TFA; however, this approach resulted in lower yields. As the final purification step, the product was purified via preparative reverse-phase HPLC (Phase A: water 0.1% TFA, Phase B: $CH_3CN$; Phase B 5% to 90%) afforded the title product **Rh518bisCTet** as a bright red solid and as TFA salt (3.7 mg, 5.4 µmol, 15%).

$^{1}H$ NMR (600 MHz, $CD_3CN$) δ 8.22 (dd, $J$ = 7.8, 1.3 Hz, 1H, H-24), 7.82 (td, $J$ = 7.5, 1.3 Hz, 1H, H-22), 7.77 (td, $J$ = 7.6, 1.3 Hz, 1H, H-23), 7.32 (d, $J$ = 6.7 Hz, 1H, H-21), 7.00 (d, $J$ = 9.5 Hz, 2H, H-6, H-14), 6.90 – 6.85 (m, 4H, H-1, H-3, H-11, H-9), 5.14 (s, 4H, H-26, H-27), 3.34 (q, $J$ = 7.6 Hz, 4H, H-40, H-41), 1.48 (t, $J$ = 7.6 Hz, 6H, H-42, H-43).

$^{13}C$ NMR (151 MHz, $CD_3CN$) δ 171.93 (s), 166.89 (s), 166.22 (s), 159.67 (s), 159.44 (s), 156.76 (s), 155.78 (s), 133.44 (d), 131.46 (s), 130.65 (d), 130.23 (d), 130.20 (d), 129.51 (d), 128.20 (d), 115.57 (d), 113.00 (d), 102.41 (d), 96.79 (d), 45.67 (t), 28.08 (t), 11.32 (q).

HRMS-ESI $m/z$ for: calc. 575.2262 $[M+H]^+$; found 575.2258$[M+H]^+$.

## Synthesis of 3'-(((6-ethyl-1,2,4,5-tetrazin-3-yl)methyl)(methyl)amino)-6'-(methylamino)-3H-spiro[isobenzofuran-1,9'-xanthen]-3-one (Rh528monoCTet)

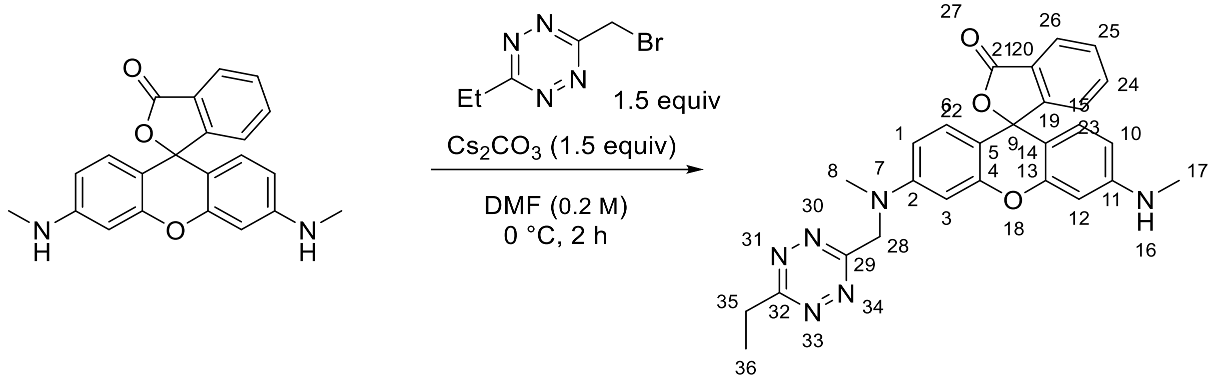


In a round bottom flask charged with 4 Å molecular sieves under Argon, **5a** (5 mg, 14 µmol, 1 equiv) was dissolved in anhydrous dimethylformamide (0.05 mL, 0.2 M) and cooled down to 0 °C. Then, $Cs_2CO_3$ (6.8 mg, 20 µmol, 1.5 equiv) was added at 0 °C. After 5 min, **3a** (5 mg, 20 µmol, 1.5 equiv), dissolved in anhydrous dimethylformamide (0.02 mL), was added. The resulting mixture was stirred for 2 h at the same temperature. Conversion of **5a** to the resulting product was monitored by LCMS. Longer reactions times and higher temperatures led to the degradation of the product, due to the instability of the tetrazine under basic conditions. Next, the reaction was quenched with 2 ml of $CH_3CN$ (0.1 % TFA), loaded onto a C18 column (Biotage Sfär C18D, 12g) and subjected to crude purification and desalting by reverse-phase Biotage purification using phase A (water with 0.1% TFA) and phase B ($CH_3CN$) under a gradient of 5-90% phase B. The crude mixture could also be purified on column chromatography (silica, DCM/MeOH 20:1 to 4:1), by replacing $CH_3CN$ with 2 mL of DCM containing 0.1% TFA; however, this approach resulted in lower yields. As the final purification step, the product was purified via preparative reverse-phase HPLC (Phase A: water 0.1% TFA, Phase B: $CH_3CN$; Phase B 5% to 90%) afforded the title product **Rh528monoCTet** as a bright red solid and as TFA salt (2.3 mg, 3.8 µmol, 25%).

$^1$H NMR (600 MHz, $CD_3CN$) δ 8.66 – 8.57 (m, 1H, H-26), 8.44 – 8.37 (m, 1H, H-24), 8.34 (d, *J* = 7.3 Hz, 1H, H-25), 7.86 (d, *J* = 7.7 Hz, 1H, H-23), 7.33 (d, *J* = 1.5 Hz, 1H, H-6), 7.24 (d, *J* = 1.8 Hz, 2H, H-1, H-3), 7.17 (d, *J* = 8.6 Hz, 1H, H-15), 7.04 (d, *J* = 2.4 Hz, 1H, H-12), 7.01 (dd, *J* = 8.6, 2.4 Hz, 1H, H-10), 5.84 (s, 2H, H-28), 5.48 (d, *J* = 5.1 Hz, 1H, NH), 3.97 (q, *J* = 7.6 Hz, 2H, H-35), 3.93 (s, 3H, H-8), 3.46 (d, *J* = 5.0 Hz, 3H, H-17), 2.13 (t, *J* = 7.6 Hz, 3H, H-36).

$^{13}$C NMR (151 MHz, $CD_3CN$) δ 172.40 (s), 169.97 (s), 167.65 (s), 153.78 (s), 153.56 (s), 153.26 (s), 152.74 (s), 151.44 (s), 135.72 (d), 130.28 (d), 129.38 (d), 129.23 (d), 127.83 (s), 125.08 (d), 124.52 (d), 110.48 (d), 109.65 (d), 108.25 (s), 107.09 (s), 99.38 (d), 97.47 (d), 85.18 (s), 55.07 (t), 39.65 (q), 29.99 (q), 28.71 (t), 11.95 (q).

HRMS-ESI *m/z* for: calc. 481.1983 $[M+H]^+$; found 481.1984 $[M+H]^+$.

## Synthesis of 3',6'-bis(((6-ethyl-1,2,4,5-tetrazin-3-yl)methyl)(methyl)amino)-3H-spiro[isobenzofuran-1,9'-xanthen]-3-one (Rh538bisCTet)

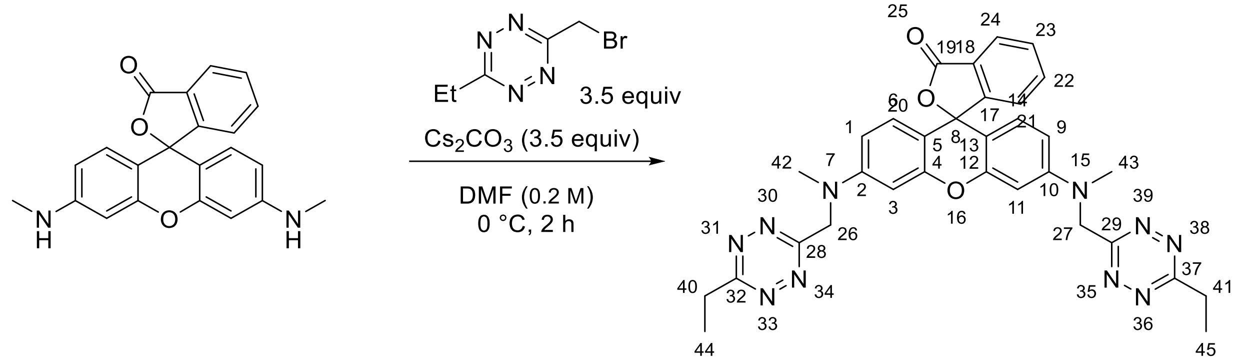


In a round bottom flask charged with 4 Å molecular sieves under Argon, **5a** (5 mg, 14 µmol, 1 equiv) was dissolved in anhydrous dimethylformamide (0.05 mL, 0.2 M) and cooled down to 0 °C. Then, $Cs_2CO_3$ (16 mg, 49 µmol, 3.5 equiv) was added at 0 °C. After 5 min, **3a** (10 mg, 49 µmol, 3.5 equiv), dissolved in anhydrous dimethylformamide (0.01 mL), was added. The resulting mixture was stirred for 2 h at the same temperature. Conversion of **5a** to the resulting product was monitored by LCMS. Longer reactions times and higher temperatures led to the degradation of the product, due to the instability of the tetrazine under basic conditions. Next, the reaction was quenched with 2 ml of $CH_3CN$ (0.1 % TFA), loaded onto a C18 column (Biotage Sfär C18D, 12g) and subjected to crude purification and desalting by reverse-phase Biotage purification using phase A (water with 0.1% TFA) and phase B ($CH_3CN$) under a gradient of 5-90% phase B. As the final purification step, the product was purified via preparative reverse-phase HPLC (Phase A: water 0.1% TFA, Phase B: $CH_3CN$; Phase B 5% to 90%) afforded the title product **Rh538bisCTet** as a bright red solid and as TFA salt (1.6 mg, 2.3 µmol, 16%).

$^1$H NMR (600 MHz, $CD_3CN$) δ 8.60 (d, *J* = 7.6 Hz, 1H, H-24), 8.38 (t, *J* = 7.5 Hz, 1H, H-22), 8.32 (t, *J* = 7.5 Hz, 1H, H-23), 7.83 (d, *J* = 7.6 Hz, 1H, H-21), 7.40 – 7.28 (m, 2H, H-6, H-14), 7.23 (s, 4H, H-1, H-3, H-9, H-11), 5.82 (s, 4H, H-26, H-27), 3.96 (q, *J* = 7.6 Hz, 4H, H-40, H-41), 3.90 (s, 6H, H-42, H-43), 2.11 (t, *J* = 7.6 Hz, 6H, H-44, H-45).

$^{13}$C NMR (151 MHz, $CD_3CN$) δ 198.42 (s), 172.38 (s), 171.46 (s), 167.61 (s), 153.23 (s), 151.49 (s), 135.76 (d), 130.33 (d), 129.39 (d), 127.75 (s), 125.13 (d), 124.47 (d), 109.70 (d), 108.08 (s), 99.34 (d), 83.03 (s), 55.05 (t), 39.63 (q), 28.69 (t), 11.94 (q).

HRMS-ESI *m/z* for: calc. 603.2575 $[M+H]^+$; found 603.2572 $[M+H]^+$ .

**Synthesis of 3-(((6-ethyl-1,2,4,5-tetrazin-3-yl)methyl)(methyl)amino)-5,5-dimethyl-7-(methylamino)-3'H,5H-spiro[dibenzo[b,e]siline-10,1'-isobenzofuran]-3'-one (SiRh628monoCTet)**

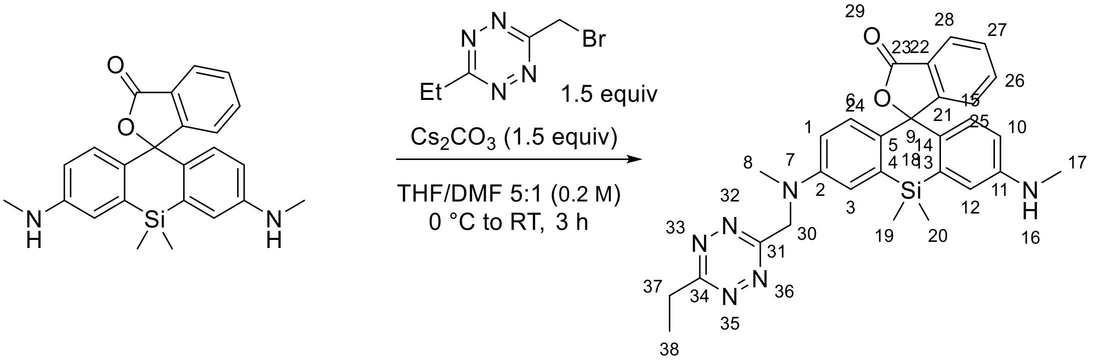


In a round bottom flask charged with 4 Å molecular sieves under Argon, **compound 11** (6 mg, 15.0 µmol, 1 equiv) was dissolved in a 1:5 mixture of anhydrous dimethylformamide and anhydrous tetrahydrofuran (0.2 mL, 0.2 M) and cooled down to 0 °C. Then, $Cs_2CO_3$ (7.5 mg, 22.5 µmol, 1.5 equiv) was added at 0 °C. After 5 min, **3a** (4.5 mg, 22.5 µmol, 1.5 equiv), dissolved in anhydrous dimethylformamide (0.05 mL), was added dropwise. The resulting mixture was stirred for 3 h until reaching room temperature. Conversion of **compound 11** to the resulting product was monitored by LCMS. Longer reactions times and higher temperatures led to the degradation of the product, due to the instability of the tetrazine under basic conditions. Next, the reaction was quenched with 1 ml of $CH_3CN$ (0.1 % TFA), loaded onto a C18 column (Biotage Sfär C18D, 12g) and subjected to crude purification and desalting by reverse-phase Biotage purification using phase A (water with 0.1% TFA) and phase B ($CH_3CN$) under a gradient of 5-90% phase B. The crude mixture could also be purified on column chromatography (silica, DCM to DCM/MeOH 100:1), by replacing $CH_3CN$ with 1 mL of DCM containing 0.1% TFA; however, this approach resulted in lower yields. As the final purification step, the product was purified via preparative reverse-phase HPLC (Phase A: water 0.1% TFA, Phase B: $CH_3CN$; Phase B 5% to 90%) afforded the title product **SiRh628monoCTet** as a blue solid and as a TFA salt (1.8 mg, 2.8 µmol, 17%).

$^1$H NMR (600 MHz, $CD_3CN$) δ 7.91 (t, *J* = 1.0 Hz, 1H, H-28), 7.72 (td, *J* = 7.5, 1.1 Hz, 1H, H-26), 7.62 (td, *J* = 7.5, 0.9 Hz, 1H, H-27), 7.24 (dt, *J* = 7.7, 0.9 Hz, 1H, H-25), 7.16 (dd, *J* = 2.3, 1.1 Hz, 1H, H-6), 6.92 (d, *J* = 2.7 Hz, 1H, H-12), 6.77 – 6.69 (m, 2H, H-1, H-3), 6.68 (d, *J* = 8.7 Hz, 1H, H-15), 6.48 (dd, *J* = 8.7, 2.7 Hz, 1H, H-10), 5.17 (s, 2H, H-30), 3.29 (q, *J* = 7.6 Hz, 2H, H-37), 3.25 (s, 3H, H-8), 2.77 (s, 3H, H-17), 1.44 (t, *J* = 7.6 Hz, 3H, H-38), 0.58 (s, 3H, H-20), 0.50 (s, 3H, H-19).

$^{13}$C NMR (151 MHz, $CD_3CN$) δ 197.88 (s), 170.27 (s), 167.32 (s), 159.44 (s), 154.82 (s), 149.12 (s), 147.79 (s), 136.48 (s), 134.37 (d), 132.32 (s), 131.39 (s), 129.04 (d), 128.11 (d), 127.98 (d), 126.01 (s), 125.25, (d) 124.16 (d), 116.76 (d), 116.35 (d), 113.70 (d), 113.25 (d) , 88.51 (s), 54.37 (t), 38.85 (q), 29.31 (q), 28.02 (t), 11.31 (q), -0.89 (q), -2.33 (q).

HRMS-ESI *m/z* for: calc. 523.2269 $[M+H]^+$; found 523.2272 $[M+H]^+$ .

**Synthesis of 3,7-bis(allylamino)-5,5-dimethyl-3'H,5H-spiro[dibenzo[*b,e*]siline-10,1'-isobenzofuran]-3'-one (SiRh640bisCTet)**

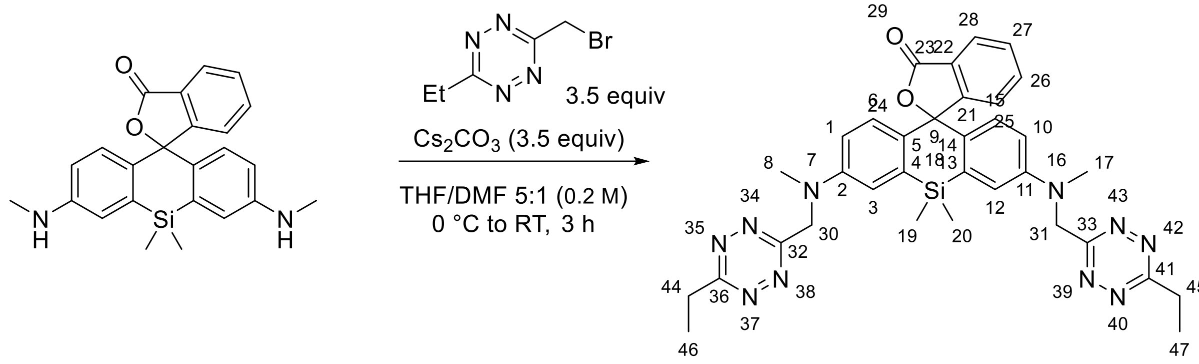


In a round bottom flask charged with 4 Å molecular sieves under Argon, **compound 11** (5 mg, 13 µmol, 1 equiv) was dissolved in a 1:5 mixture of anhydrous dimethylformamide and anhydrous tetrahydrofuran (0.2 mL, 0.2 M) and cooled down to 0 °C. Then, $Cs_2CO_3$ (15 mg, 45.5 µmol, 3.5 equiv) was added at 0 °C. After 5 min, **3a** (9.0 mg, 45.5 µmol, 3.5 equiv), dissolved in anhydrous tetrahydrofuran (0.1 mL), was added dropwise. The resulting mixture was stirred for 3 h until reaching room temperature. Conversion of **compound 11** to the resulting product was monitored by LCMS. Longer reactions times and higher temperatures led to the degradation of the product, due to the instability of the tetrazine under basic conditions. Next, the reaction was quenched with 1 ml of $CH_3CN$ (0.1 % TFA), loaded onto a C18 column (Biotage Sfär C18D, 12g) and subjected to crude purification and desalting by reverse-phase Biotage purification using phase A (water with 0.1% TFA) and phase B ($CH_3CN$) under a gradient of 5-90% phase B. As the final purification step, the product was purified via preparative reverse-phase HPLC (Phase A: water 0.1% TFA, Phase B: $CH_3CN$; Phase B 5% to 90%) afforded the title product **SiRh640bisCTet** as a blue solid and as a TFA salt (1.1 mg, 1.6 µmol, 12%).

$^1$H NMR (600 MHz, $CD_3CN$) δ 7.91 (dt, *J* = 7.6, 1.0 Hz, 1H, H-28), 7.71 (td, *J* = 7.6, 1.1 Hz, 1H, H-26), 7.61 (td, *J* = 7.5, 0.9 Hz, 1H, H-27), 7.21 (dt, *J* = 7.7, 0.9 Hz, 1H, H-25), 7.14 (t, *J* = 1.7 Hz, 2H, H-6, H-15), 6.71 (d, *J* = 1.6 Hz, 4H, H-1, H-3, H-10, H-12), 5.17 (s, 4H, H-30, H-31), 3.28 (q, *J* = 7.6 Hz, 4H, H-44, H-45), 3.25 (s, 6H, H-8, H-17), 1.44 (t, *J* = 7.6 Hz, 6H, H-46, H-47), 0.55 (s, 3H, H-20), 0.47 (s, 3H, H-19).

$^{13}$C NMR (151 MHz, $CD_3CN$) δ 171.64 (s), 170.20 (s), 167.30 (s), 154.70 (s), 147.81 (s), 136.35 (s), 134.44 (d), 132.06 (s), 129.09 (d), 128.00 (d), 125.90 (s), 125.30 (d), 124.07 (d), 116.74 (d), 113.74 (d), 90.95 (s), 54.34 (t), 38.84 (q), 28.02 (t) , 11.32 (q), -0.94 (q), -2.28 (q).

HRMS-ESI *m/z* for: calc. 645.2865 $[M+H]^+$; found 645.2865$[M+H]^+$.

**Synthesis of 3-(dimethylamino)-5,5-dimethyl-7-(methyl(3-(6-methyl-1,2,4,5-tetrazin-3-yl)propyl)amino)-3'H,5H-spiro[dibenzo[*b,e*]siline-10,1'-isobenzofuran]-3'-one (SiRh634monoC3Tet)**

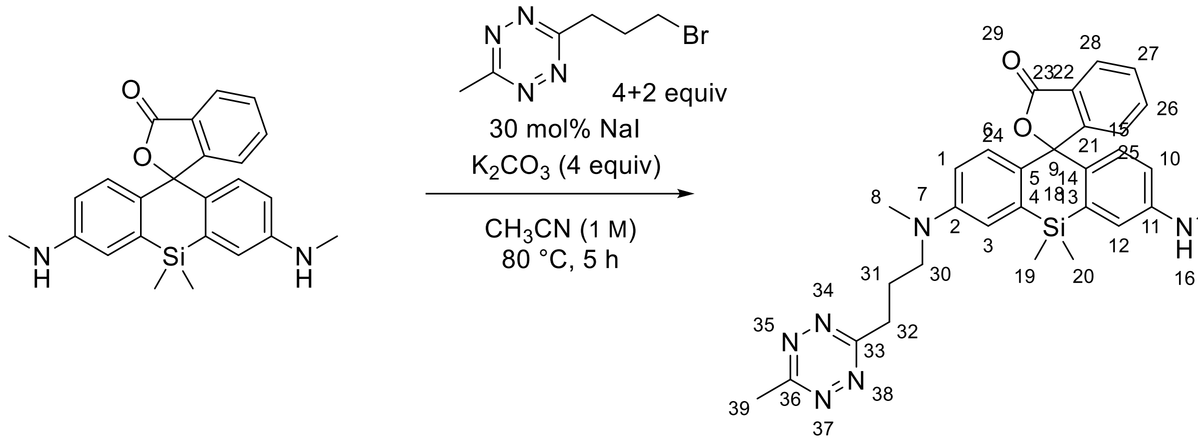


In a round bottom flask charged with 4 Å molecular sieves under Argon, **compound 11** (14 mg, 35.0 µmol, 1 equib) was dissolved in anhydrous acetonitrile (35.0 µL, 1 M) and then $K_2CO_3$ (19 mg, 140.0 µmol, 4 equiv) and sodium iodide (1.48 mg, 10.5 µmol, 30 mol%) were added. After 5 min, **compound 3b** (30 mg, 140.0 µmol, 4 eq), dissolved in anhydrous acetonitrile (35.0 µL), was added dropwise. The resulting mixture was stirred at 80 °C for 5 hours. Conversion of **compound 11** to the resulting product was monitored by LCMS. Since poor conversion was observed, other 2 equiv of **compound 3b** were added after 3 h. Next, the reaction was quenched with 3 ml of DCM (0.1 % TFA) and loaded on a short pad of silica gel. The crude mixture was purified on column chromatography (silica, DCM/MeOH 200:1 to 20:1). As the final purification step, the product was purified via preparative reverse-phase HPLC (Phase A: water 0.1% TFA, Phase B: $CH_3CN$; Phase B 5% to 90%), affording the title product **SiRh634monoC3Tet** as a deep blue solid and as a TFA salt (0.7 mg, 1.3 µmol, 3.7%).

$^1$H NMR (600 MHz, $CD_3CN$) δ 7.94 (dt, *J* = 7.7, 1.0 Hz, 1H, H-28), 7.74 (td, *J* = 7.5, 1.2 Hz, 1H, H-26), 7.63 (td, *J* = 7.5, 0.9 Hz, 1H, H-27), 7.26 (dt, *J* = 7.7, 0.9 Hz, 1H, H-25), 7.03 (d, *J* = 2.9 Hz, 1H, C-3), 6.95 (d, *J* = 2.7 Hz, 1H, C-15), 6.69 (dd, *J* = 8.8, 8.8 Hz, 2H, C-6, C-15), 6.60 (dd, *J* = 9.0, 2.9 Hz, 1H, C-1), 6.49 (dd, *J* = 8.8, 2.7 Hz, 1H, C-10), 3.54 (dd, *J* = 8.1, 6.6 Hz, 2H, H-30), 3.30 (t, *J* = 7.4 Hz, 2H, H-32), 2.97 (s, 3H, H-8), 2.92 (s, 3H, H-39), 2.78 (s, 3H, C-17), 2.21 (p, *J* = 7.4 Hz, 2H, C-31), 0.63 (s, 3H, C-20), 0.54 (s, 3H, C-19).

HRMS-ESI *m/z* for: calc. 537.2429 $[M+H]^+$; found 537.2441$[M+H]^+$.

**Synthesis of (*S,E*)-cyclooct-2-en-1-yl ((1*S*,2*R*,3*R*,5*R*)-2-hydroxy-3-methyl-5-(((2*R*,4*S*)-2,5,12-trihydroxy-2-(2-hydroxyacetyl)-7-methoxy-6,11-dioxo-3,4,6,11-tetrahydro-2H-naphtho[2,3-g]chromen-4-yl)oxy)cyclohexyl)carbamate (compound 12)**

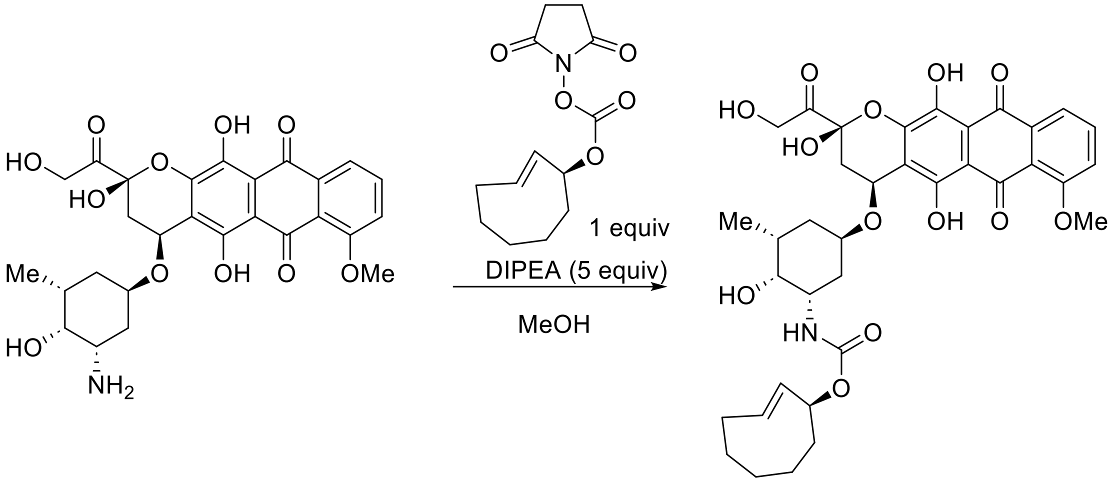


Doxorubicin hydrochloride (29 mg, 0.05 mmol, 1.0 equiv) was dissolved in MeOH (1 mL) and cooled to 0 °C. Then, TCO-NHS ester [(*E*)-cyclooct-2-en-1-yl (2,5-dioxopyrrolidin-1-yl) carbonate axial diastereoisomer from BLDpharm (BD01357271)] (13.5 mg, 0.05 mmol, 1.0 equiv) and ethyldiisopropylamine (DIPEA; 42 µL, 0.25 mmol, 5.0 equiv) were added successively. The reaction mixture was stirred at room temperature for 3 h. Upon completion, the mixture was quenched with water and extracted with DCM (3x10 mL). The combined organic layers were washed with brine (10 mL × 1), dried over $MgSO_4$, filtered, and concentrated under reduced pressure. The residue was purified via column chromatography (silica, EtOAc to EtOAc/MeOH 20:1) to afford **compound 12** as a red solid (18 mg, 26 µmol, 52%).

$^1H$ NMR (400 MHz, $CDCl_3$) δ 13.93 (s, 1H), 13.19 (s, 1H), 8.03 (d, *J* = 7.8, 1H), 7.82 (t, *J* = 8.1 Hz, 1H), 7.42 (d, *J* = 8.2 Hz, 1H), 5.82 (m, 1H), 5.55 (m, 2H), 5.45 – 5.25 (m, 2H), 4.82 (d, *J* = 11.1 Hz, 2H), 4.10 (s, 3H), 3.83 – 3.65 (m, 2H), 3.26 (d, *J* = 18.7, 1H), 3.04 – 2.89 (m, 2H), 2.55 – 2.36 (m, 2H), 2.23 – 0.98 (m, 13H), 0.98 – 0.69 (m, 2H).

HRMS-ESI *m/z* for $C_{36}H_{41}NO_{13}$: calc. 718.2470 $[M+Na]^+$; found 718.2459 $[M+Na]^+$ .

Spectroscopic data are consistent with those reported in the literature.[19]

## Synthesis of the Rh518bisCTet-Pep1 adduct

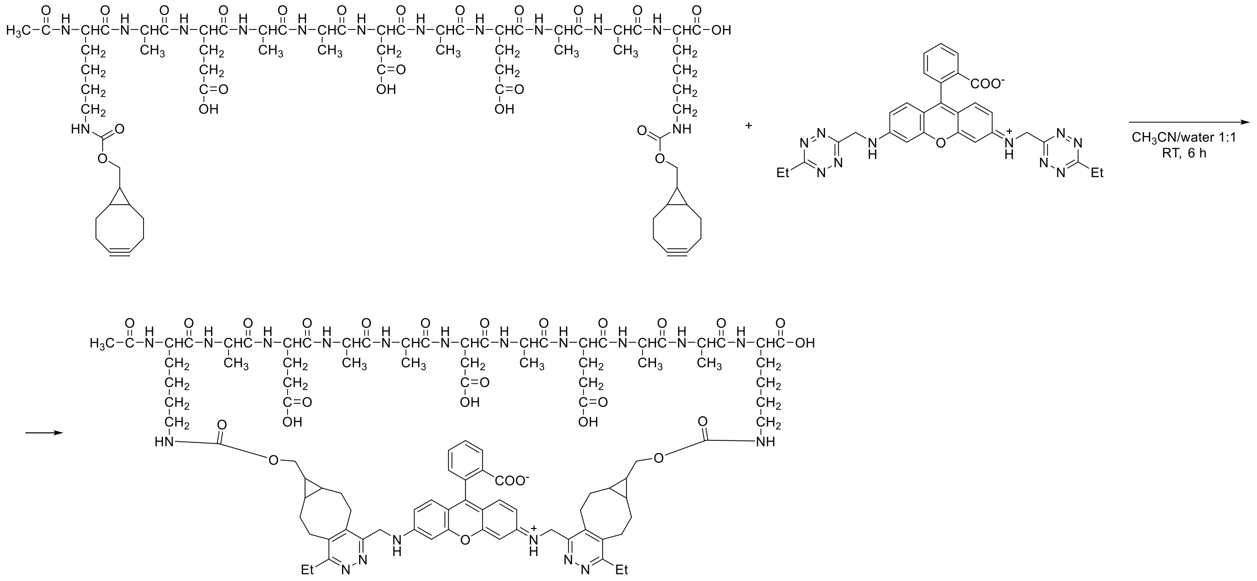


In a brown-glass HPLC vial, **Rh518bisCTet** (0.5 mg, 0.85 µmol, 1 equiv) was dissolved in a mixture acetonitrile/water 1:1 (170 µL, 0.005 M) and then Pep1 (1.5 mg, 0.94 µmol, 1.1 equiv) from a 30 mM stock solution (31 µL) was added. The resulting mixture was shaken at RT for 6 hours. Conversion of **Rh518bisCTet** to the resulting product was monitored by LCMS. Afterwards, the resulting crude residue was purified via preparative reverse-phase HPLC (Phase A: water 0.1% TFA, Phase B: $CH_3CN$; Phase B 5%, 0 to 1 min, Phase B 90%, 1 min to 30 min, product elution: 17.47 min), affording the title product **Rh518bisCTet-Pep1** as TFA salt (1.1 mg, 0.53 µmol, 62%).

HRMS-ESI *m/z* for $C_{98}H_{128}N_{19}O_{26}$: calc. 1986.9227 $[M+H]^+$; found 1986.9230 $[M+H]^+$ . Because of $C^{13}$ isotope also 1987.9264 $[M+H]^+$ is detected as major signal together with its double, 994.4670 $[M+2H]^{2+}$, and triple charged 663.3136 $[M+3H]^{3+}$.

### X-Ray Crystallography

Crystallization method: red brick crystals were grown by vapor diffusion of n-hexane/DCM into a solution of **Rh538bisCTet** (~1.5 mg) in MeOH (100 µL) at RT.

Single crystal X-ray diffraction analysis of the compound of **Rh538bisCTet** (data from Dr. Dieter Schollmeyer)

Table S1. Crystal and refinement data of **Rh538bisCTet**

| | |
|---|---|
| Substance name | Rh538bisCTet |
| Empirical formula | $C_{33.55}H_{31.68}F_{2.32}N_{10}O_5$ |
| Molecular formula | $C_{32}H_{30}N_{10}O_3$, 0.774($CF_3COOH$), 0.4($H_2O$) |
| Formula weight | 699.02 |
| Temperature | 20(2) K |
| Wavelength, radiation type | 1.54186Å, CuKα |
| Diffractometer | STOE STADIVARI |
| Cristall system | Orthorhombic |
| Space group (name, number) | P $2_12_12_1$, (19) |
| Unit cells parameters | a = 9.8130(3) Å α = 90° |
| | b = 15.6020(4) Å β = 90° |
| | c = 21.2496(8) Å γ = 90° |
| Volume | 3253.37(18) $Å^3$ |
| Number of reflection and | 9067 |
| range for unit cells determination | 4.16° <=Θ<= 68.74° |
| Z | 4 |
| Density (calculated) | 1.427 $Mg/m^3$ |
| Absorption coefficient | 0.915 $mm^{-1}$ |
| Absorption correction method | None |
| F(000) | 1455 |
| Crystal size, shape and color | 0.033 x 0.078x0.097 $mm^3$, red block |
| Theta range for data collection | 3.514 bis 67.986°. |
| Index ranges | -11<=h<=10, -18<=k<=18, -25<=l<=25 |

| | |
|---|---|
| Number of reflections: | |
| measured | 23458 |
| independent | 5822 [$R_{int}$ = 0.0656] |
| observed [I>2sigma(I)] | 2699 |
| Completeness to Θmax= 67.7° | 99.4 % |
| Refinement method | Full-matrix least-squares on $F^2$ |
| Reflections / restraints / parameters | 5822 / 108 / 504 |
| Goodness-of-fit on $F^2$ | 0.885 |
| Final R indices [I>2sigma(I)] | R1 = 0.0810, wR2 = 0.2008 |
| R indices (all data) | R1 = 0.1494, wR2 = 0.2306 |
| Absolute structure parameter | 0.5(3) |
| Largest diff. peak and hole | 0.703 und -0.255 eÅ$^{-3}$ |

Deposition number (CCDC): 2548790

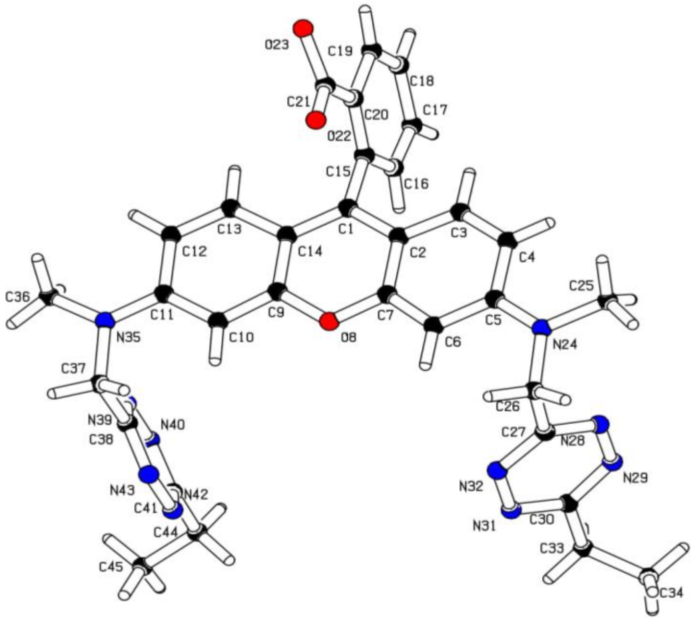


**Figure S44**. Structure of **Rh538bisCTet**, with the atom-numbering scheme. Atoms are represented with the usual CPK colour code (C: black; N: blue; O: red; H: white)

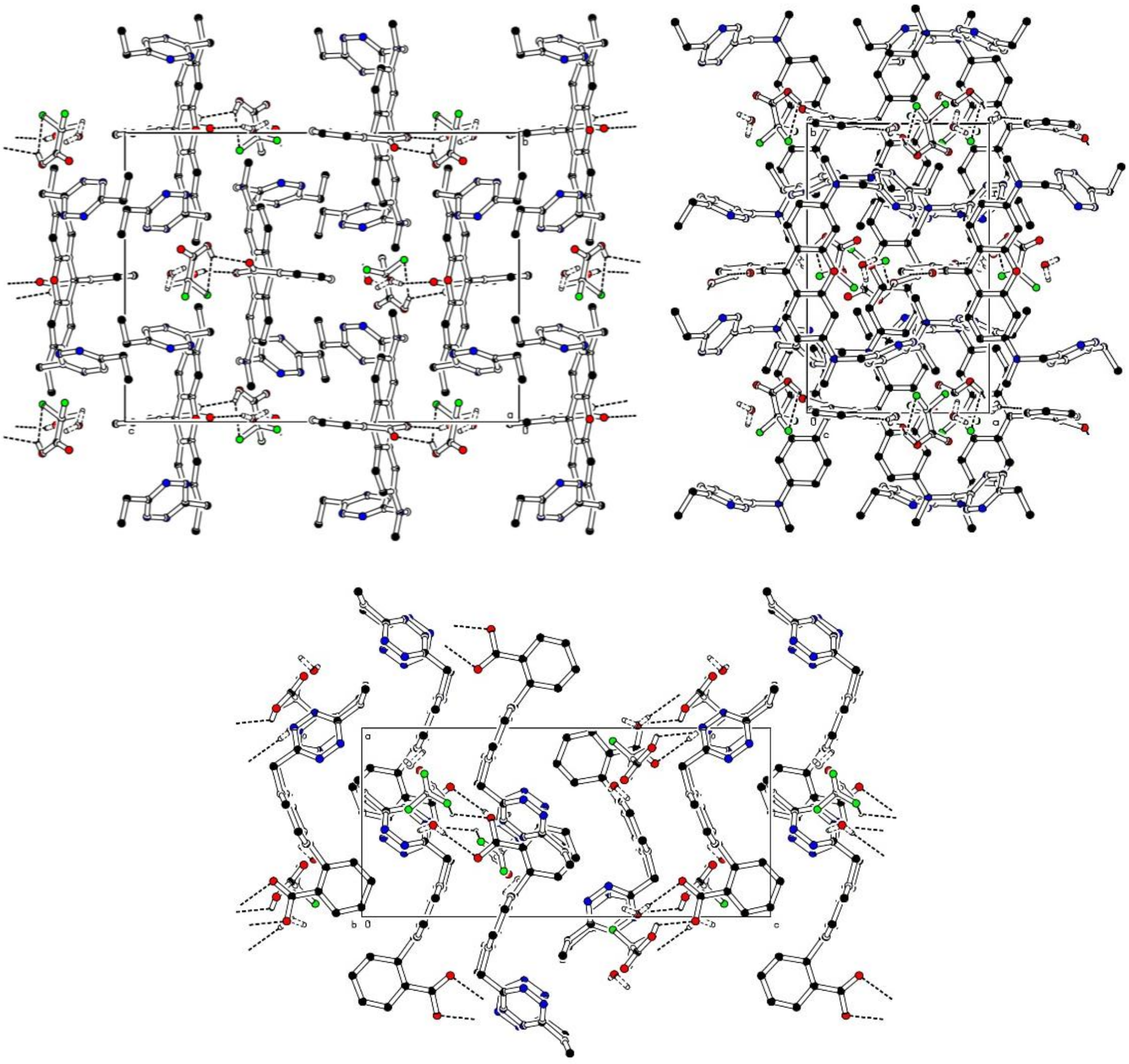

**Figure S45**. Ball-and-stick representation of the crystal packing of **Rh538bisCTet** at RT, as seen along the a (a), b (b) and c (c) cell axes. Color code as in Figure S44.

## References


(1) Zhang, X.-F.; Zhang, Y.; Liu, L. Fluorescence Lifetimes and Quantum Yields of Ten Rhodamine Derivatives: Structural Effect on Emission Mechanism in Different Solvents. *Journal of Luminescence* **2014**, *145*, 448–453. https://doi.org/10.1016/j.jlumin.2013.07.066.

(2) Schrimpf, W.; Barth, A.; Hendrix, J.; Lamb, D. C. PAM: A Framework for Integrated Analysis of Imaging, Single-Molecule, and Ensemble Fluorescence Data. *Biophysical Journal* **2018**, *114* (7), 1518–1528. https://doi.org/10.1016/j.bpj.2018.02.035.

(3) Kormos, A.; Koehler, C.; Fodor, E. A.; Rutkai, Z. R.; Martin, M. E.; Mező, G.; Lemke, E. A.; Kele, P. Bistetrazine-Cyanines as Double-Clicking Fluorogenic Two-Point Binder or Crosslinker Probes. *Chemistry – A European Journal* **2018**, *24* (35), 8841–8847. https://doi.org/10.1002/chem.201800910.

(4) Mao, W.; Shi, W.; Li, J.; Su, D.; Wang, X.; Zhang, L.; Pan, L.; Wu, X.; Wu, H. Organocatalytic and Scalable Syntheses of Unsymmetrical 1,2,4,5-Tetrazines by Thiol-Containing Promotors. *Angewandte Chemie International Edition* **2019**, *58* (4), 1106–1109. https://doi.org/10.1002/anie.201812550.

(5) Werther, P.; Yserentant, K.; Braun, F.; Grußmayer, K.; Navikas, V.; Yu, M.; Zhang, Z.; Ziegler, M. J.; Mayer, C.; Gralak, A. J.; Busch, M.; Chi, W.; Rominger, F.; Radenovic, A.; Liu, X.; Lemke, E. A.; Buckup, T.; Herten, D.-P.; Wombacher, R. Bio-Orthogonal Red and Far-Red Fluorogenic Probes for Wash-Free Live-Cell and Super-Resolution Microscopy. *ACS Cent. Sci.* **2021**, *7* (9), 1561–1571. https://doi.org/10.1021/acscentsci.1c00703.

(6) Wong, J. Y. F.; Thomson, C. G.; Vilela, F.; Barker, G. Flash Chemistry Enables High Productivity Metalation-Substitution of 5-Alkyltetrazoles. *Chem. Sci.* **2021**, *12* (40), 13413–13424. https://doi.org/10.1039/D1SC04176B.

(7) Nakao, Y.; Yada, A.; Hiyama, T. Heteroatom-Directed Alkylcyanation of Alkynes. *J. Am. Chem. Soc.* **2010**, *132* (29), 10024–10026. https://doi.org/10.1021/ja1017078.

(8) Fukuyama, T.; Yamada, K.; Nishikawa, T.; Ravelli, D.; Fagnoni, M.; Ryu, I. Site-Selectivity in TBADT-Photocatalyzed C(Sp3)–H Functionalization of Saturated Alcohols and Alkanes. *Chemistry Letters* **2018**, *47* (2), 207–209. https://doi.org/10.1246/cl.171068.

(9) Tsai, C.-S.; Liu, P.-Y.; Yen, H.-Y.; Hsu, T.-L.; Wong, C.-H. Development of Trifunctional Probes for Glycoproteomic Analysis. *Chem. Commun.* **2010**, *46* (30), 5575. https://doi.org/10.1039/c0cc00345j.

(10) Mitronova, G. Y.; Belov, V. N.; Bossi, M. L.; Wurm, C. A.; Meyer, L.; Medda, R.; Moneron, G.; Bretschneider, S.; Eggeling, C.; Jakobs, S.; Hell, S. W. New Fluorinated Rhodamines for Optical Microscopy and Nanoscopy. *Chemistry* **2010**, *16* (15), 4477–4488. https://doi.org/10.1002/chem.200903272.

(11) Zhang, T.; Yang, Z.; He, X.; Guo, L.; Wang, J.; Jiang, X.; Shen, R.; Lu, X. A Ratiometric Fluorescent Dye for Detection of Lys and Arg and Its Bioimaging in Live Cells and Zebrafish Larvae. *Anal. Methods* **2023**, *15* (6), 703–708. https://doi.org/10.1039/D2AY01740G.

(12) Hammler, D.; Marx, A.; Zumbusch, A. Fluorescence-Lifetime-Sensitive Probes for Monitoring ATP Cleavage. *Chemistry* **2018**, *24* (57), 15329–15335. https://doi.org/10.1002/chem.201803234.

(13) Numasawa, K.; Hanaoka, K.; Saito, N.; Yamaguchi, Y.; Ikeno, T.; Echizen, H.; Yasunaga, M.; Komatsu, T.; Ueno, T.; Miura, M.; Nagano, T.; Urano, Y. A Fluorescent Probe for Rapid, High-Contrast Visualization of Folate-Receptor-Expressing Tumors In Vivo. *Angew Chem Int Ed Engl* **2020**, *59* (15), 6015–6020. https://doi.org/10.1002/anie.201914826.

(14) Wiseman, R. L.; Johnson, S. M.; Kelker, M. S.; Foss, T.; Wilson, I. A.; Kelly, J. W. Kinetic Stabilization of an Oligomeric Protein by a Single Ligand Binding Event. *J Am Chem Soc* **2005**, *127* (15), 5540–5551. https://doi.org/10.1021/ja042929f.

(15) Hie, L.; Fine Nathel, N. F.; Hong, X.; Yang, Y.-F.; Houk, K. N.; Garg, N. K. Nickel-Catalyzed Activation of Acyl C−O Bonds of Methyl Esters. *Angewandte Chemie International Edition* **2016**, *55* (8), 2810–2814. https://doi.org/10.1002/anie.201511486.

(16) González, I.; Mosquera, J.; Guerrero, C.; Rodríguez, R.; Cruces, J. Selective Monomethylation of Anilines by $Cu(OAc)_2$ -Promoted Cross-Coupling with $MeB(OH)_2$. *Org. Lett.* **2009**, *11* (8), 1677–1680. https://doi.org/10.1021/ol802882k.
(17) Umezawa, K.; Yoshida, M.; Kamiya, M.; Yamasoba, T.; Urano, Y. Rational Design of Reversible Fluorescent Probes for Live-Cell Imaging and Quantification of Fast Glutathione Dynamics. *Nat Chem* **2017**, *9* (3), 279–286. https://doi.org/10.1038/nchem.2648.
(18) Egawa, T.; Koide, Y.; Hanaoka, K.; Komatsu, T.; Terai, T.; Nagano, T. Development of a Fluorescein Analogue, TokyoMagenta, as a Novel Scaffold for Fluorescence Probes in Red Region. *Chem. Commun.* **2011**, *47* (14), 4162–4164. https://doi.org/10.1039/C1CC00078K.
(19) Versteegen, R. M.; Rossin, R.; ten Hoeve, W.; Janssen, H. M.; Robillard, M. S. Click to Release: Instantaneous Doxorubicin Elimination upon Tetrazine Ligation. *Angewandte Chemie International Edition* **2013**, *52* (52), 14112–14116. https://doi.org/10.1002/anie.201305969.

## HRMS Spectra

### Compound Rh506monoCTet

+ Scan (rt: 0.211-0.343 min) Sub

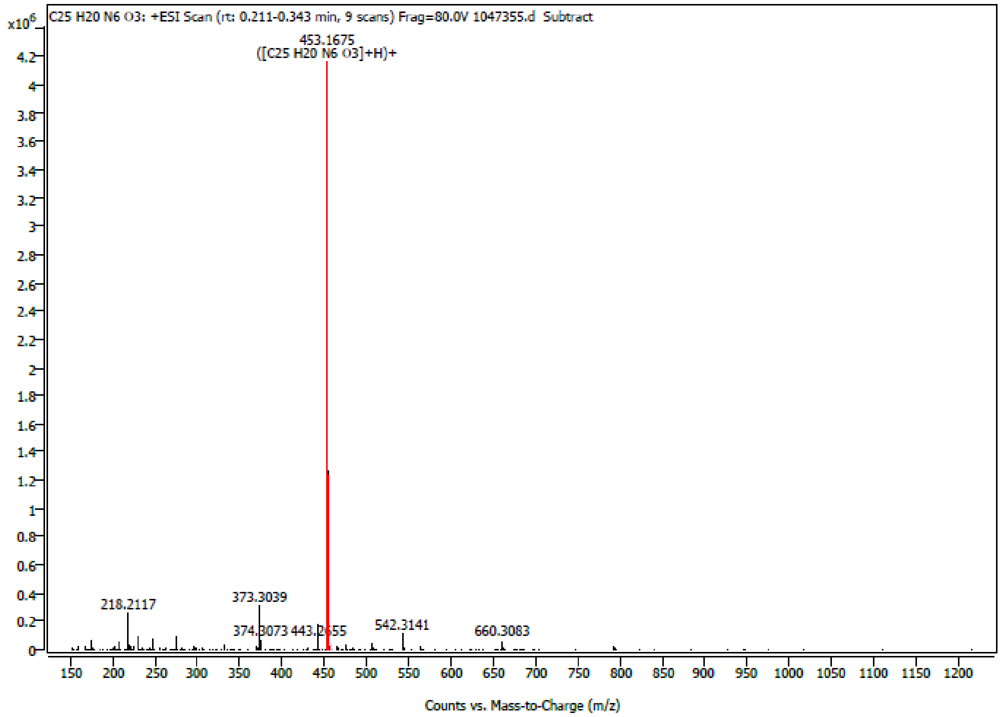

## Compound Rh518bisCTet

+ Scan (rt: 0.203-0.319 min) Sub

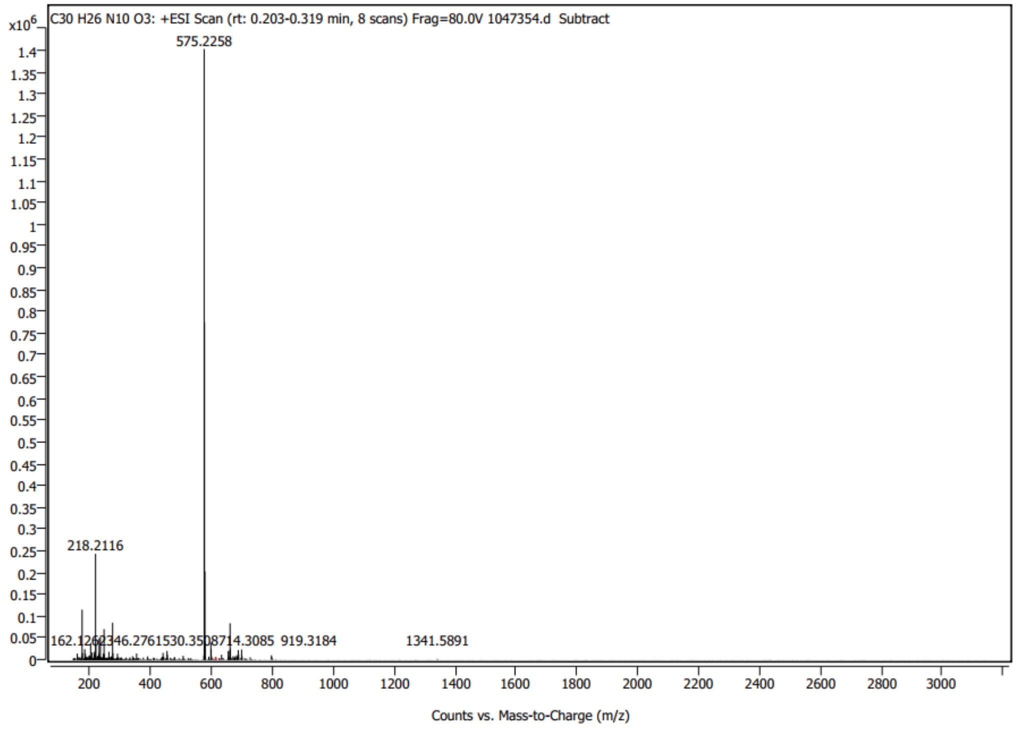


## Compound Rh528bisCTet

+ Scan (rt: 0.192-0.340 min) Sub

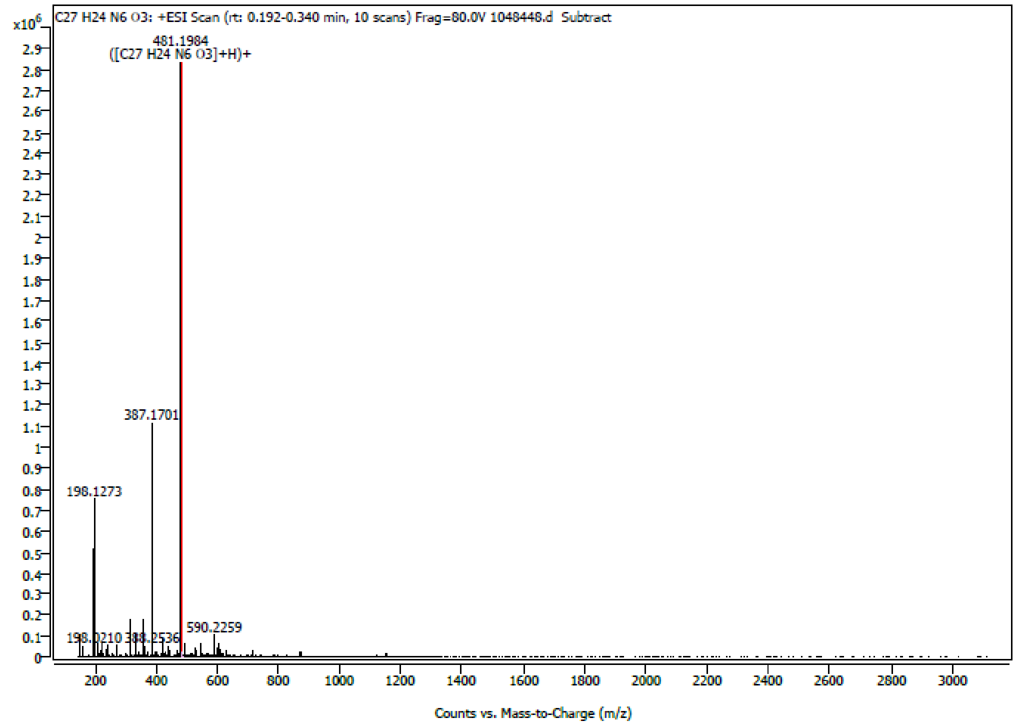

## Compound Rh538bisCTet

+ Scan (rt: 0.193-0.326 min) Sub

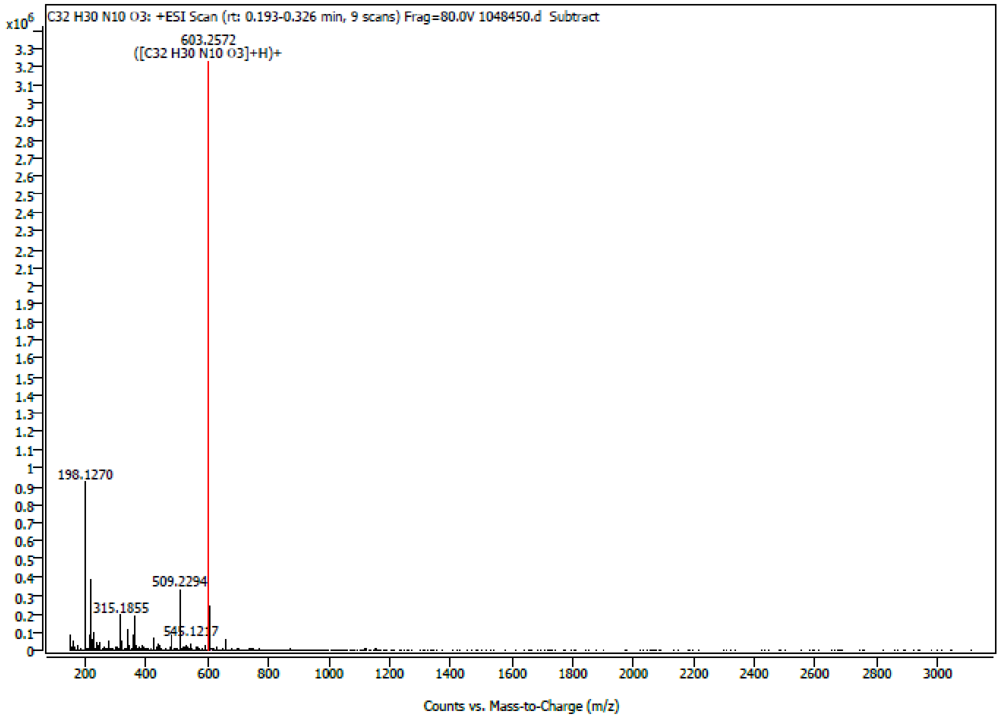


## Compound SiRh628bisCTet

+ Scan (rt: 0.190-0.338 min) Sub

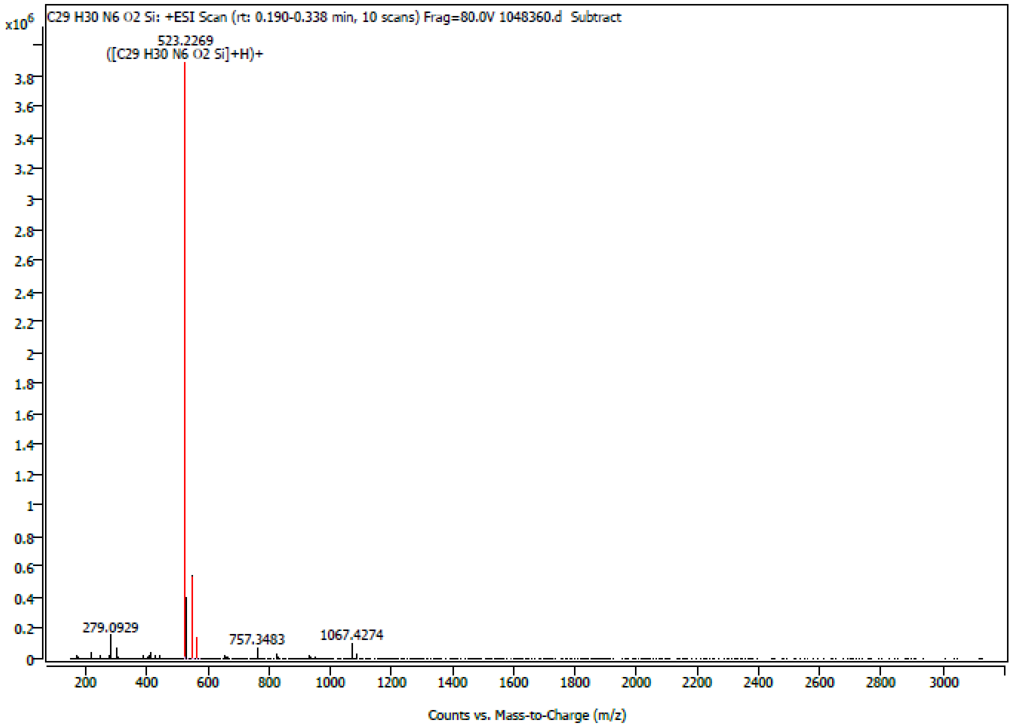

**Compound SiRh640bisCTet**

+ Scan (rt: 0.204-0.369 min) Sub

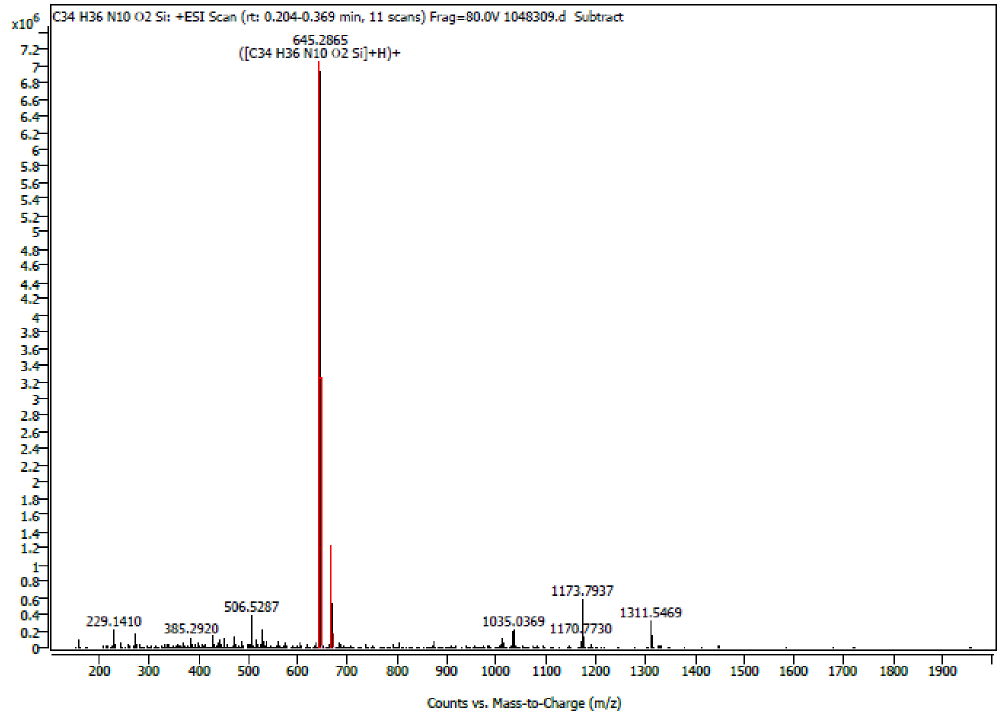


**Compound SiRh634monoC3Tet**

+ Scan (rt: 0.265-0.497 min) Sub

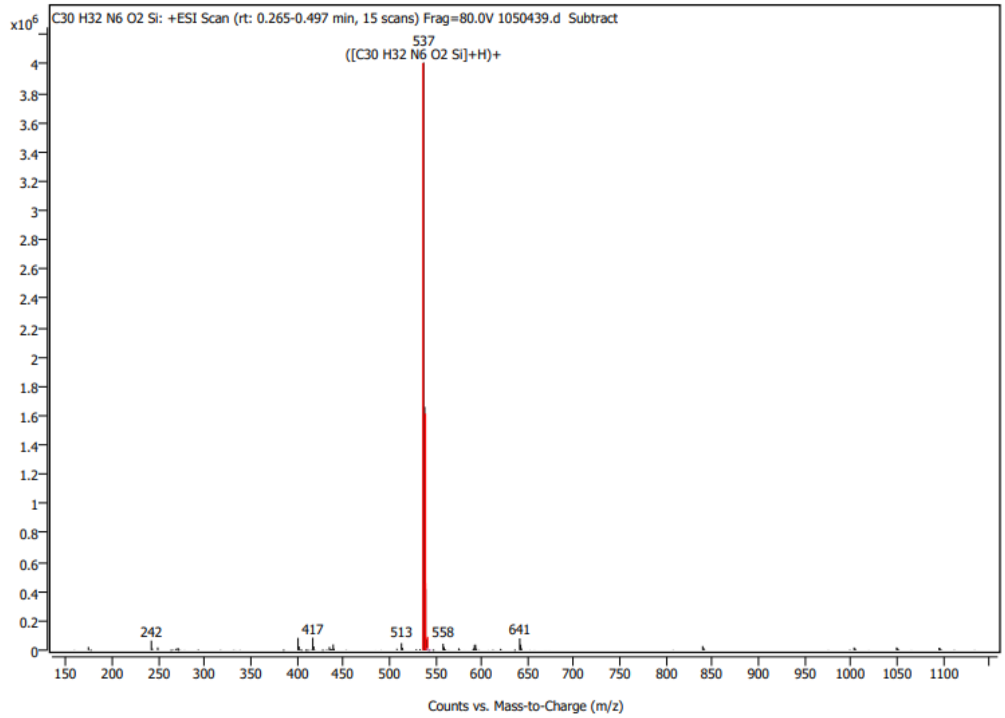

## Compound Rh518bisCTet-SingleTet

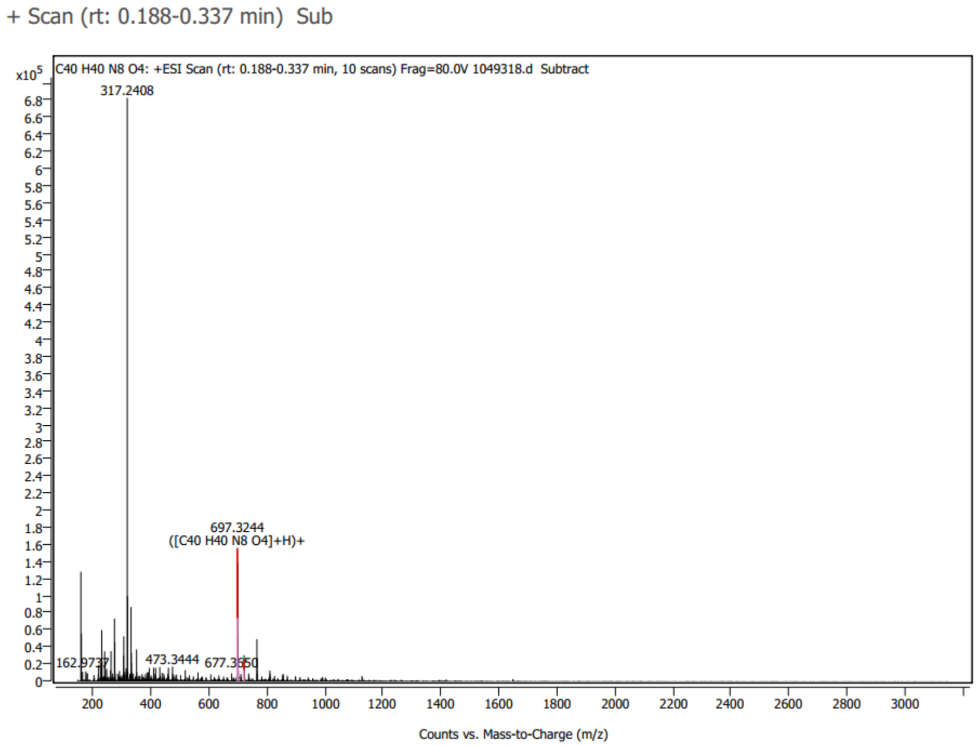


## Compound Rh518bisCTet-Pep1

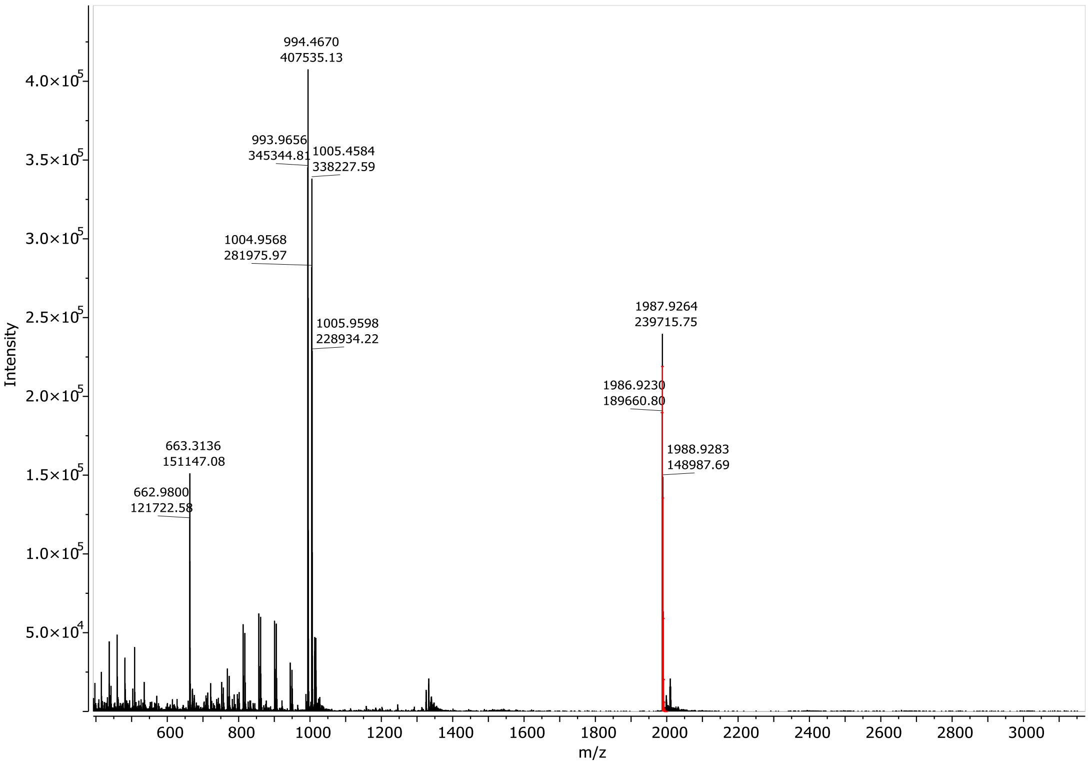

## NMR Spectra

### $^{1}$H NMR compound 1a

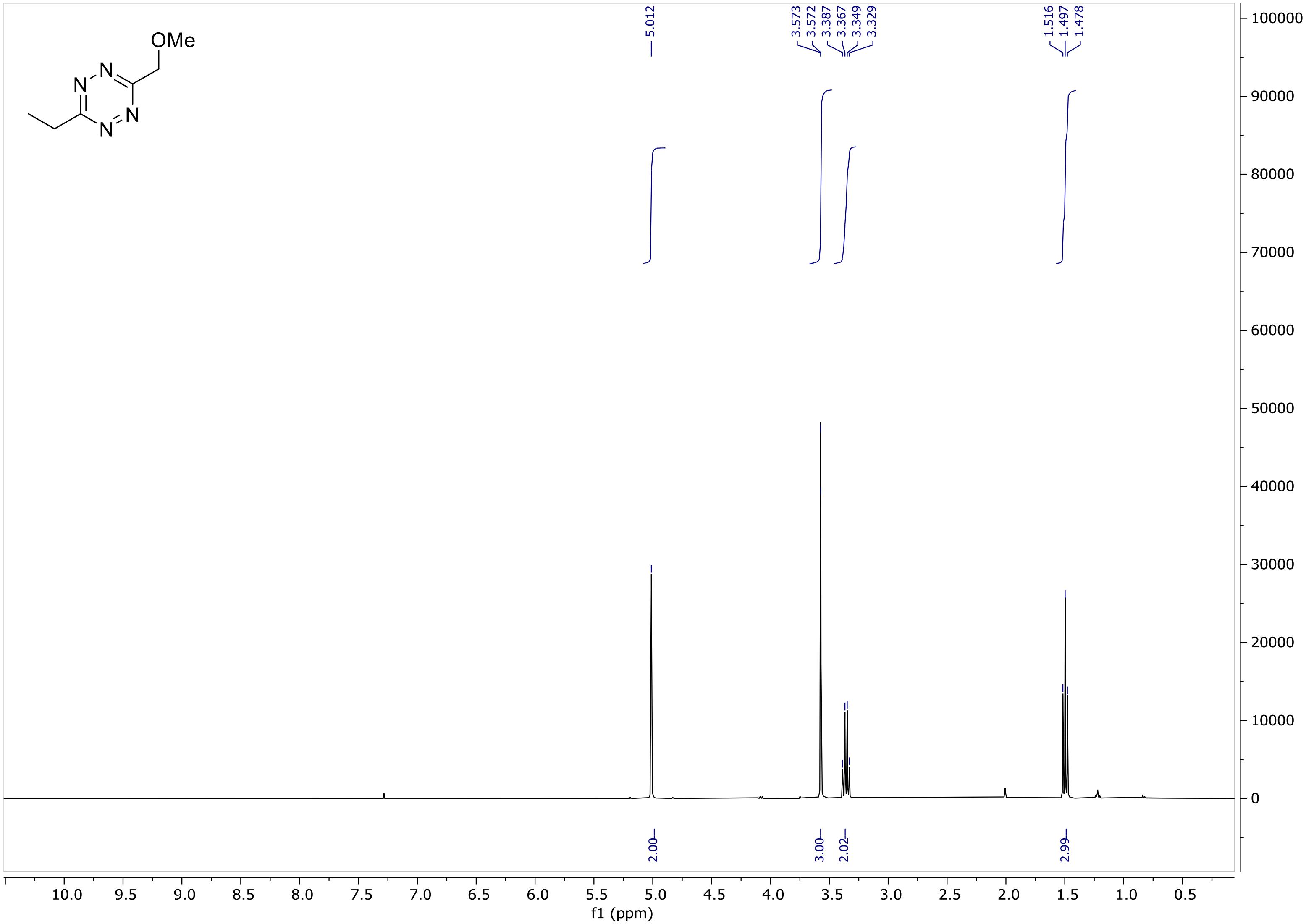

**$^{13}$C NMR compound 1a**

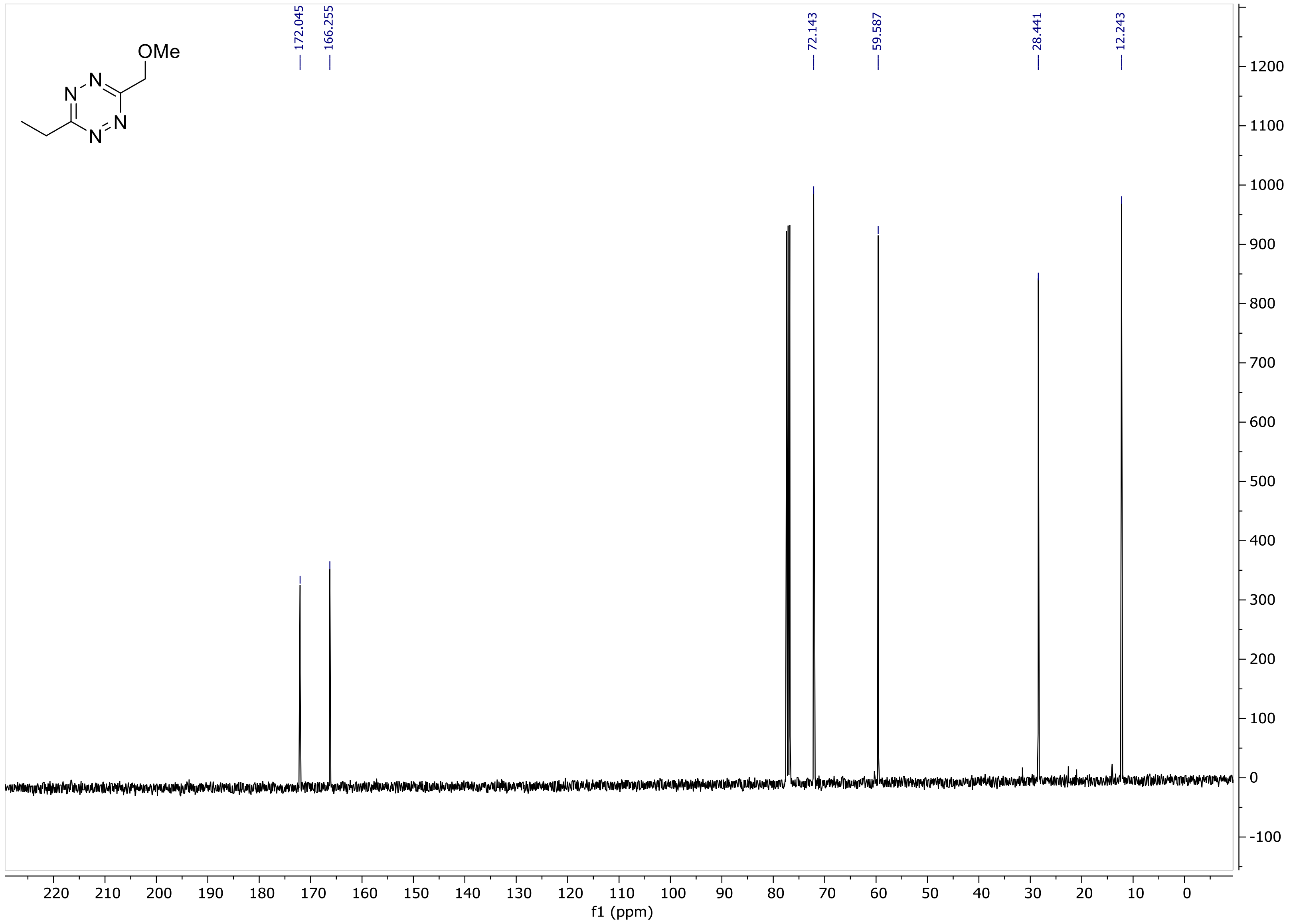

**$^{1}$H NMR compound 2a**

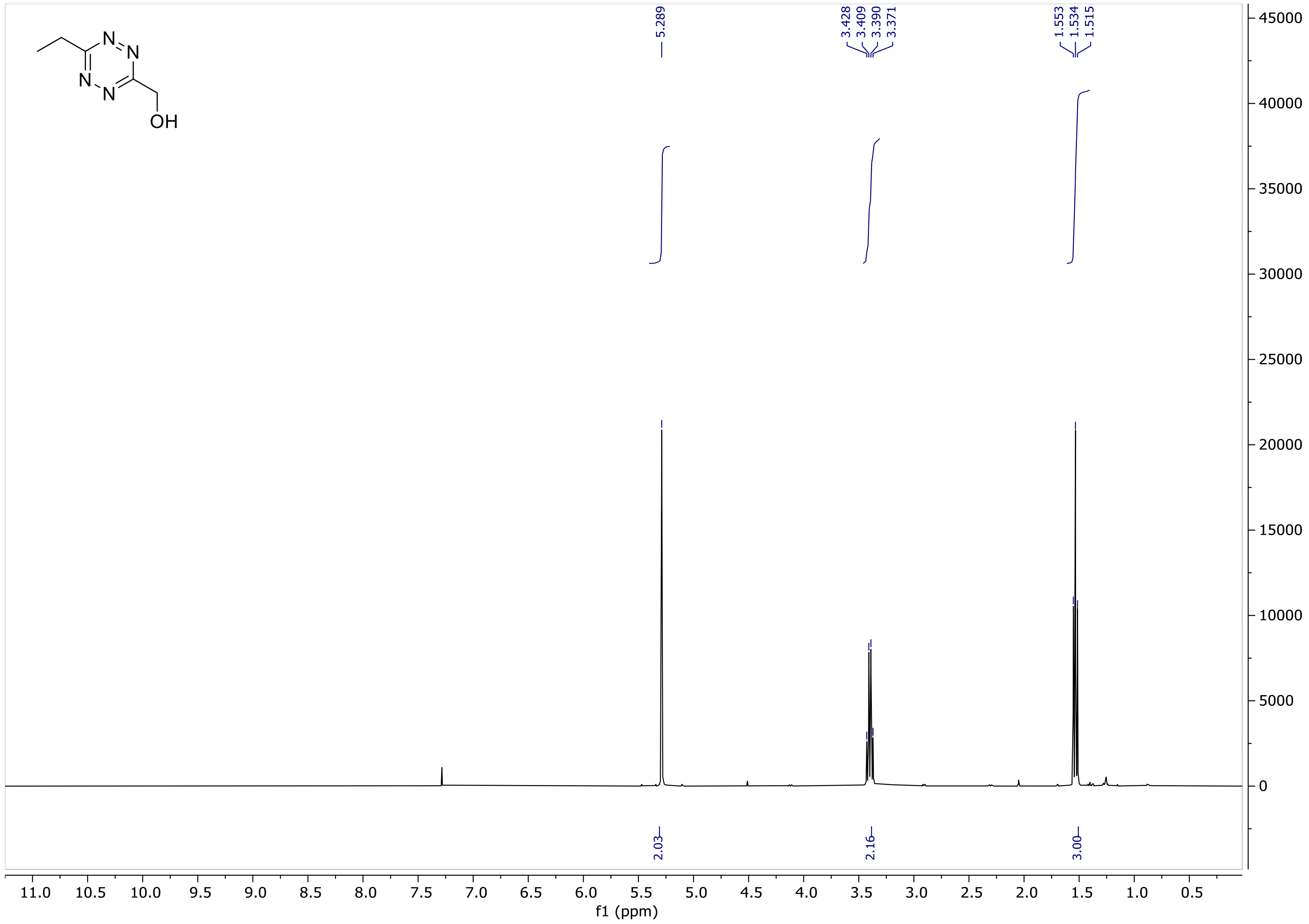

**$^{13}$C NMR compound 2a**

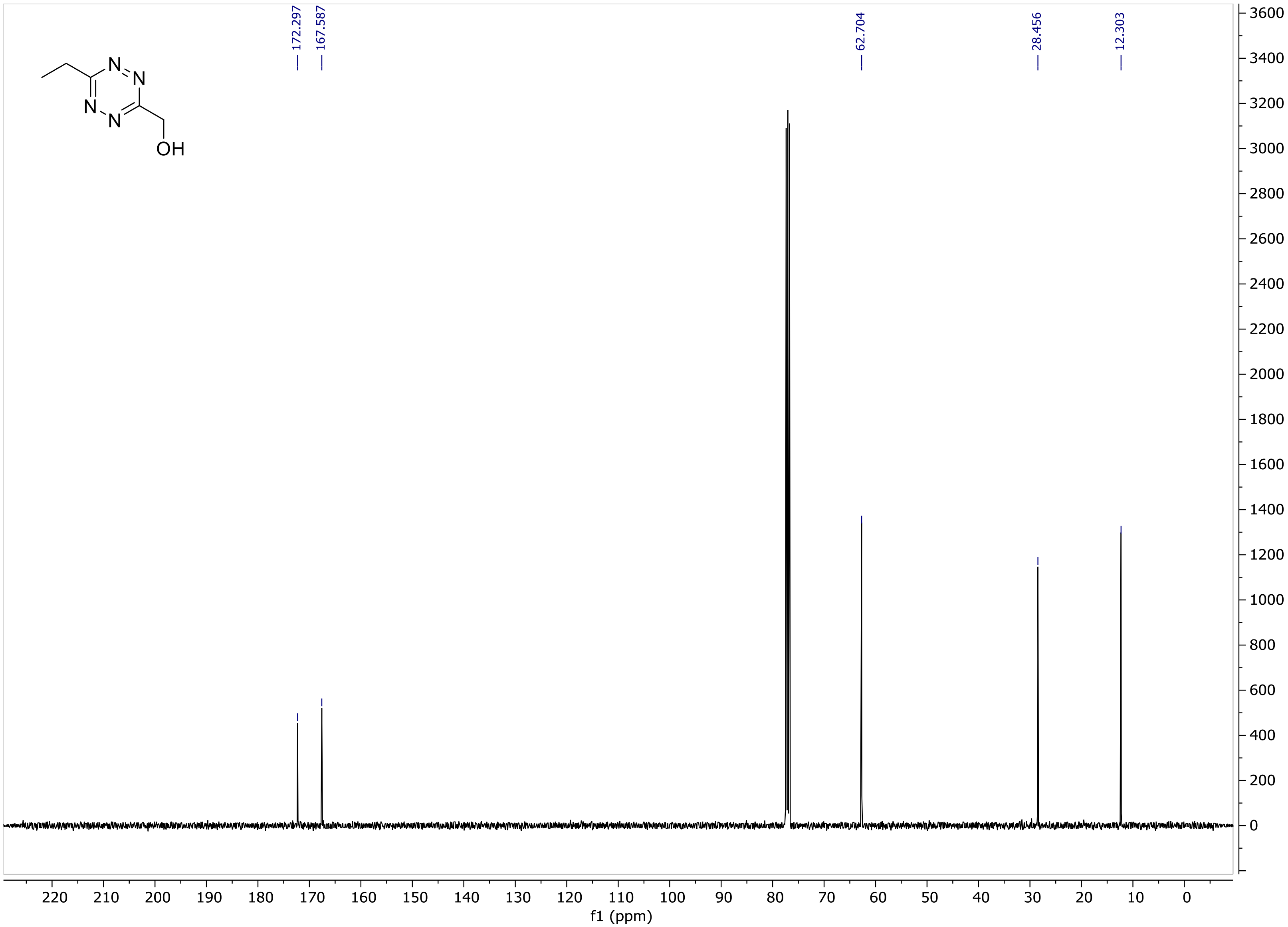

**$^{1}$H NMR compound 3a**

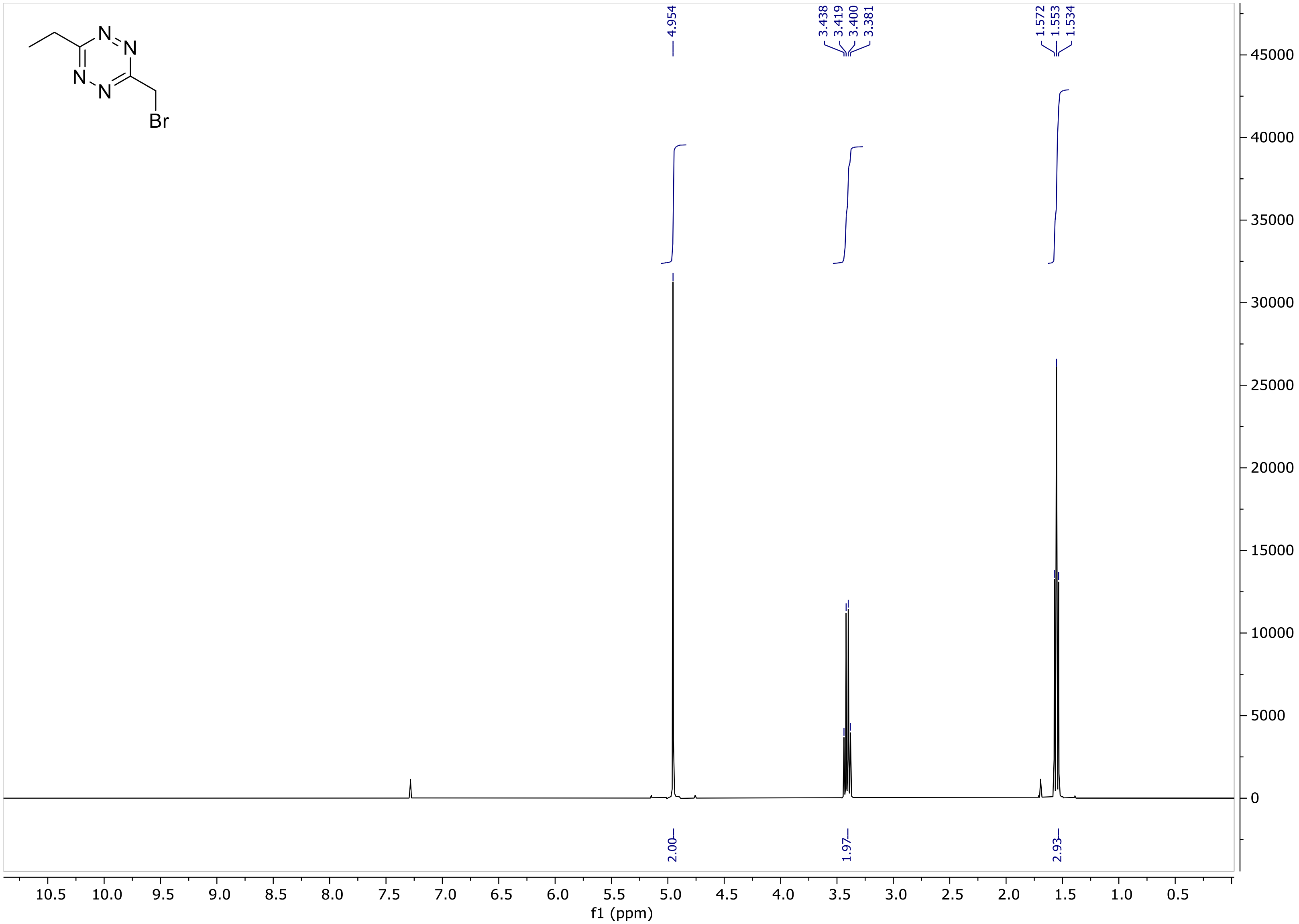

**$^{13}$C NMR compound 3a**

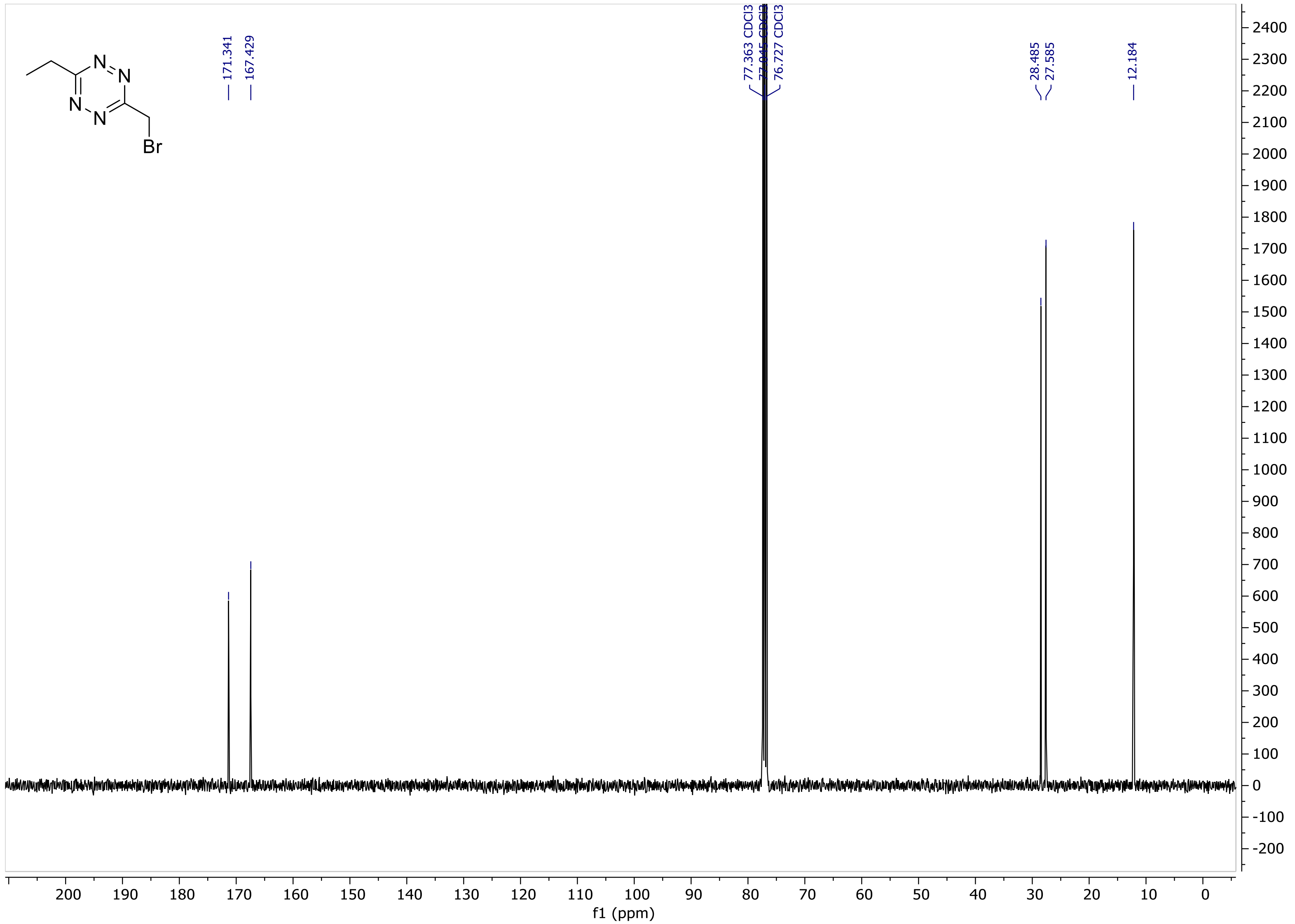

**$^{1}$H NMR compound 2b**

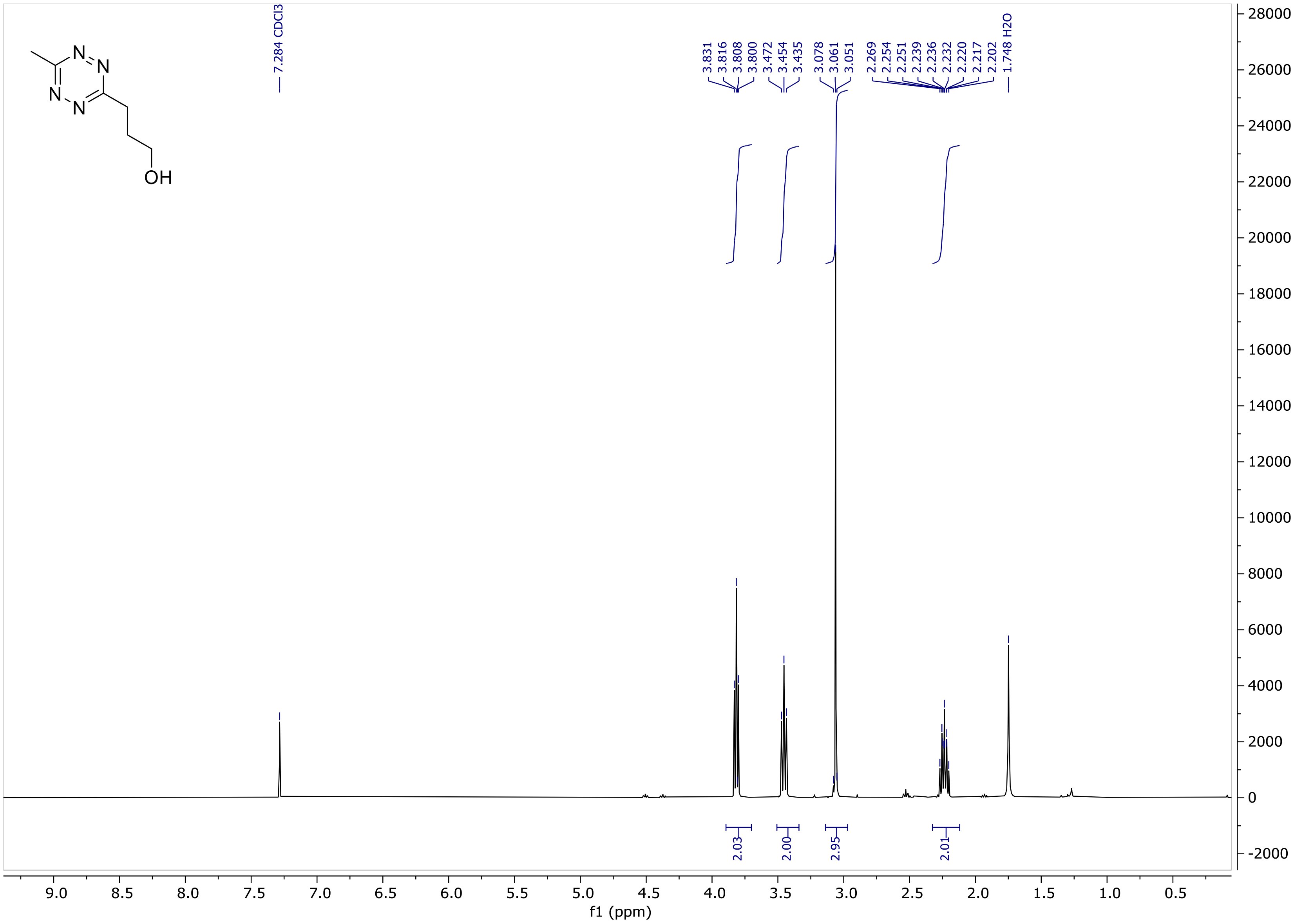

**$^{13}$C NMR compound 2b**

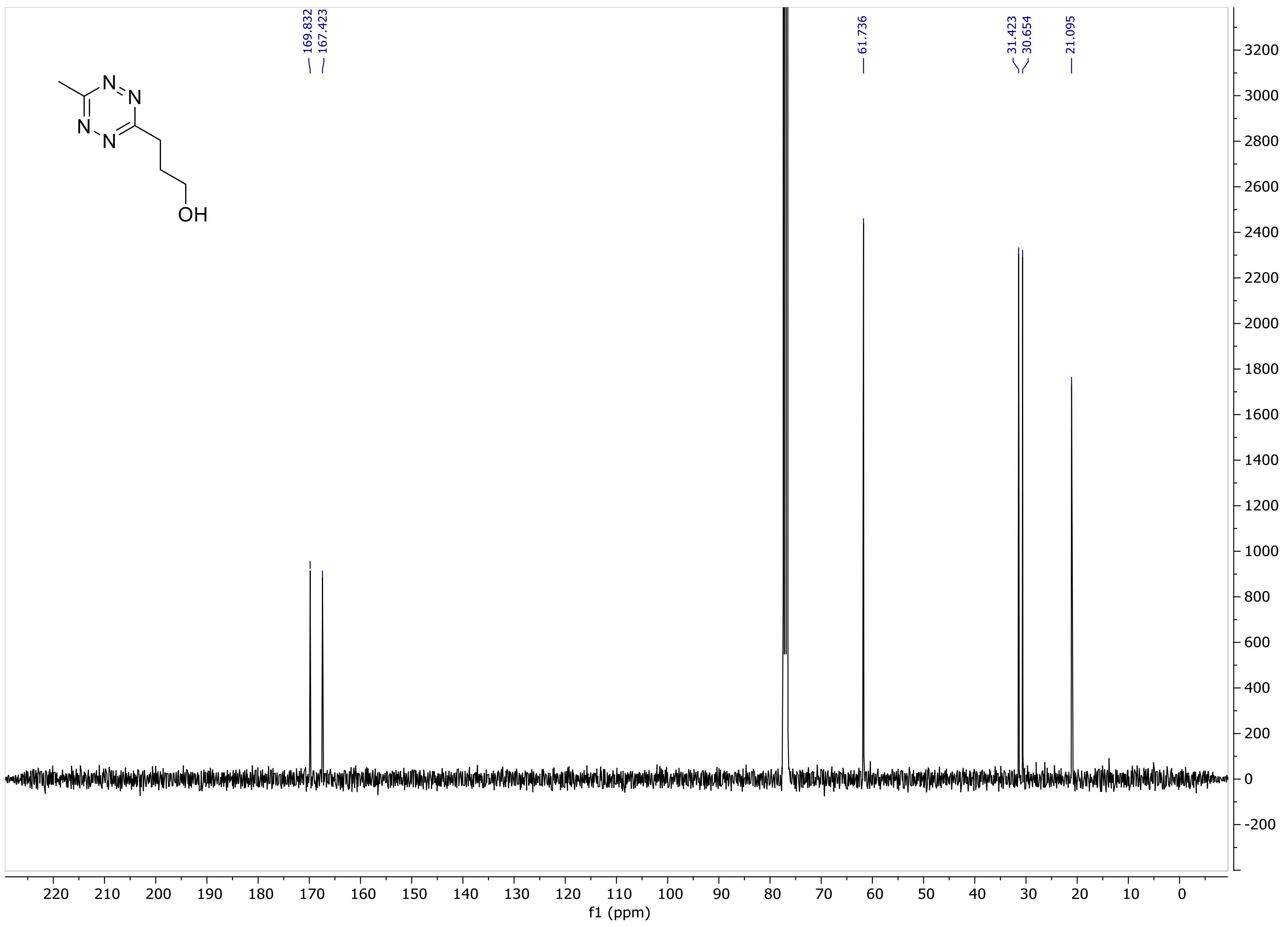

**$^{1}$H NMR compound 3b**

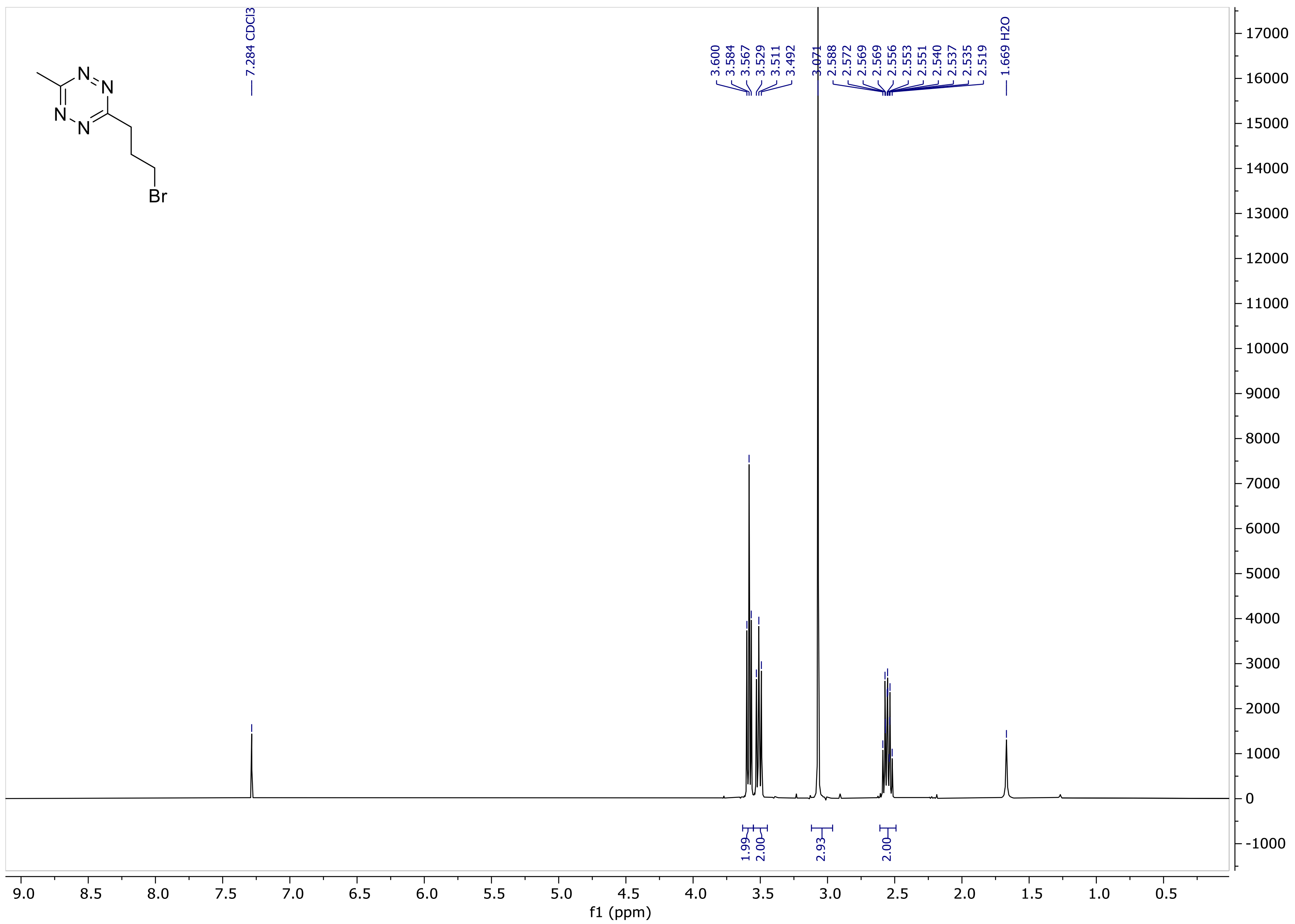

**$^{13}$C NMR compound 3b**

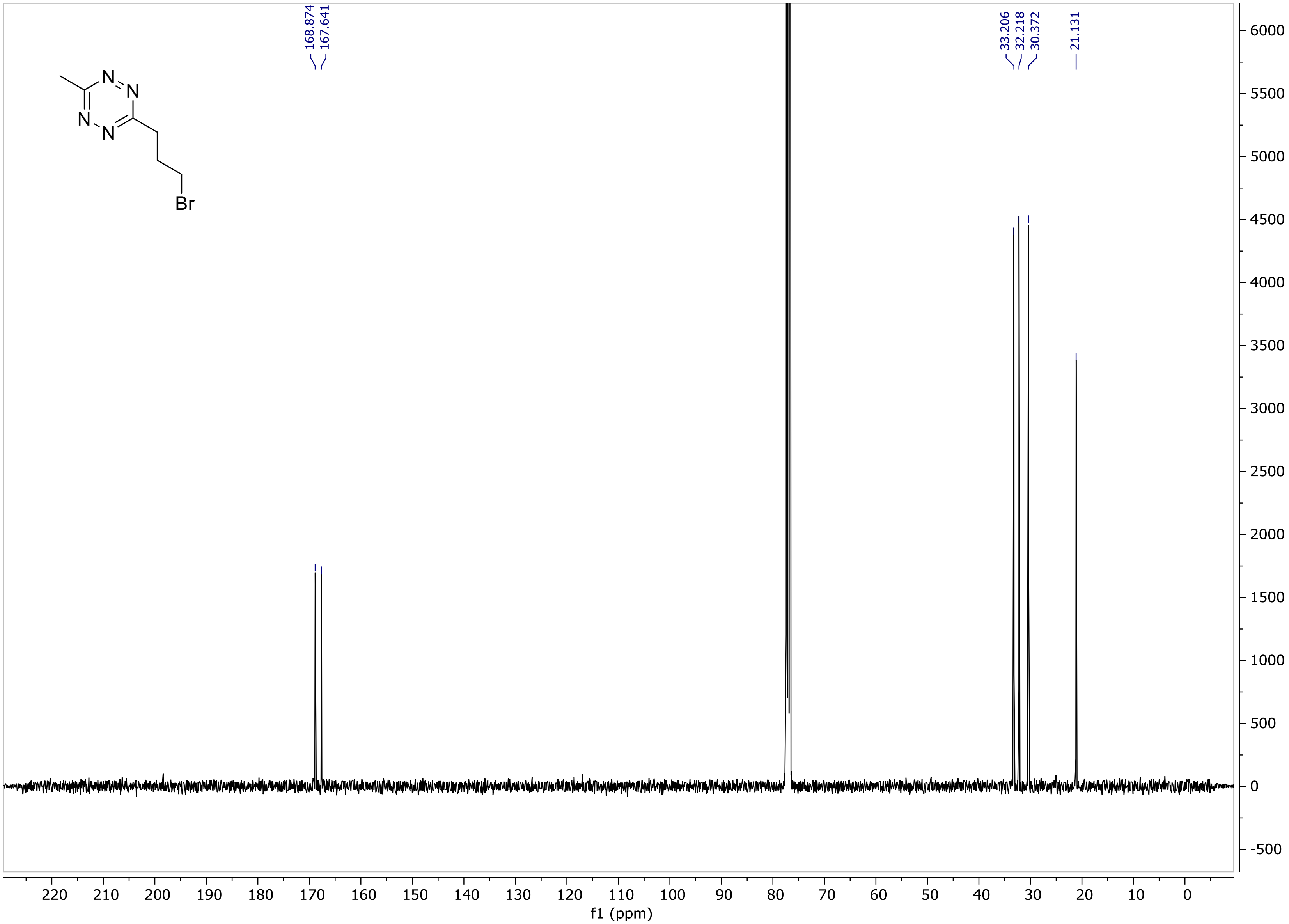

## $^{1}$H NMR compound 1c

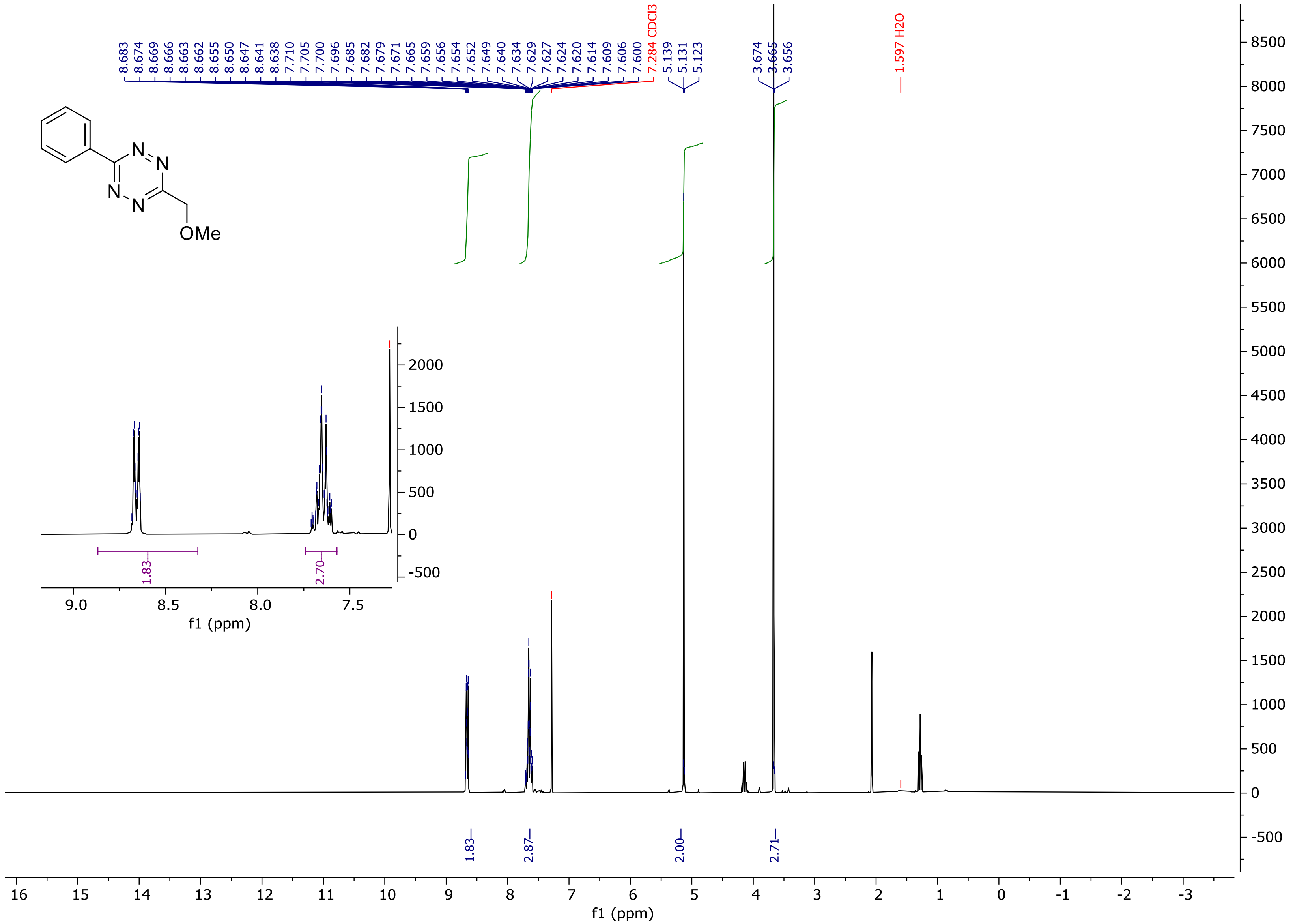

## $^{1}$H NMR compound 2c

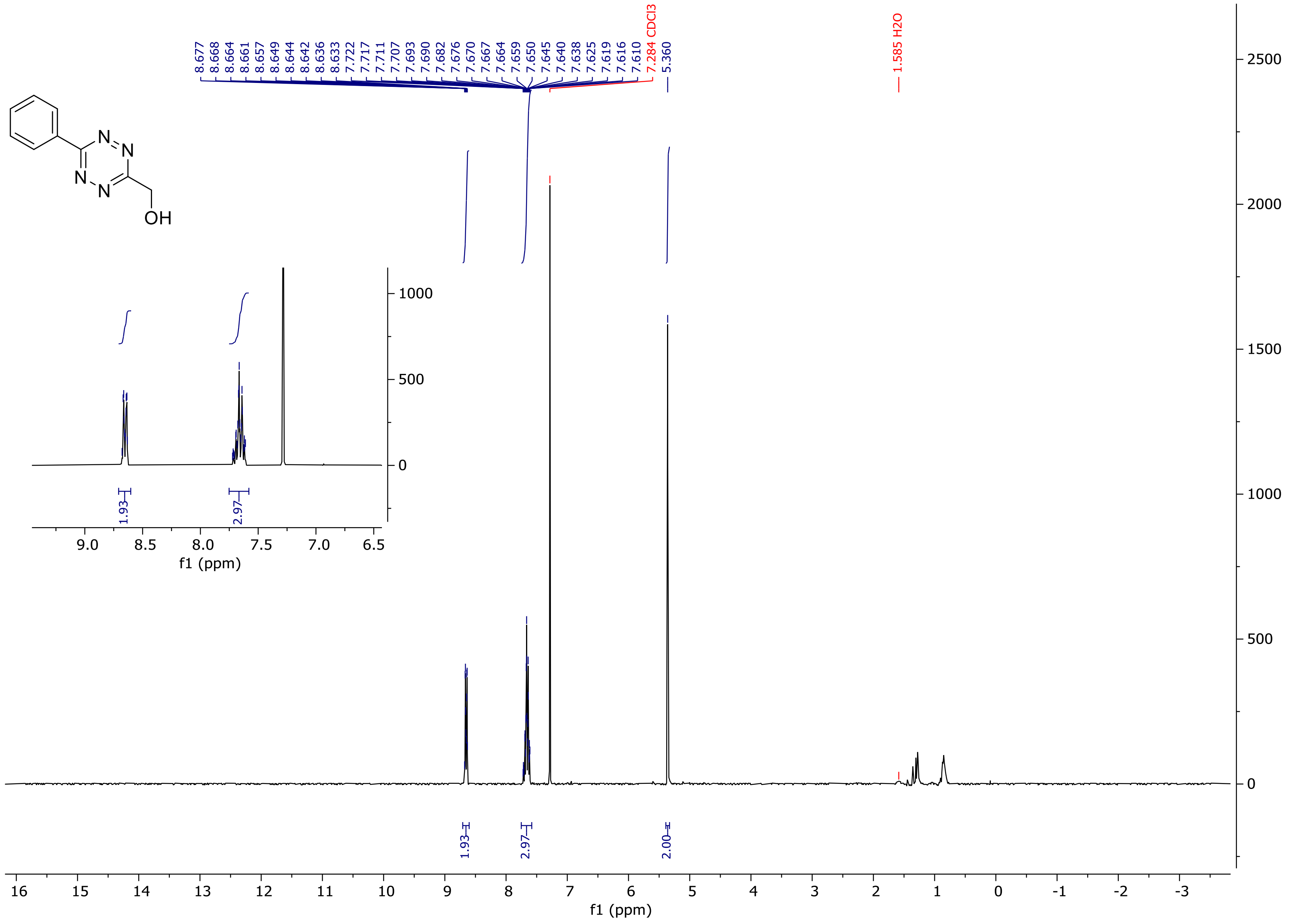

**$^{13}$C NMR compound 2c**

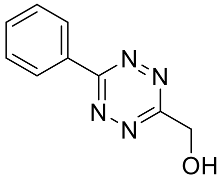

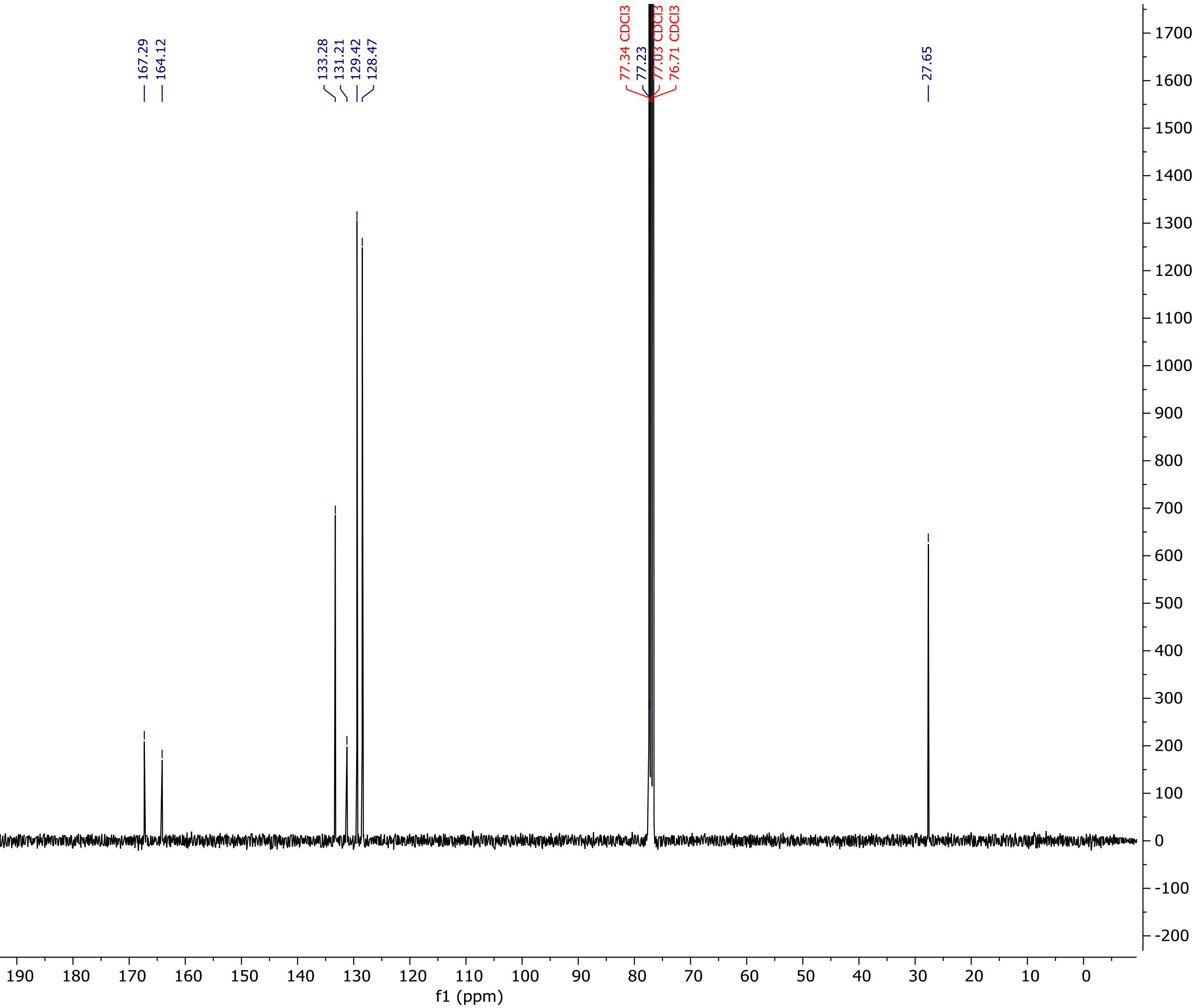

**$^{1}$H NMR compound 3c**

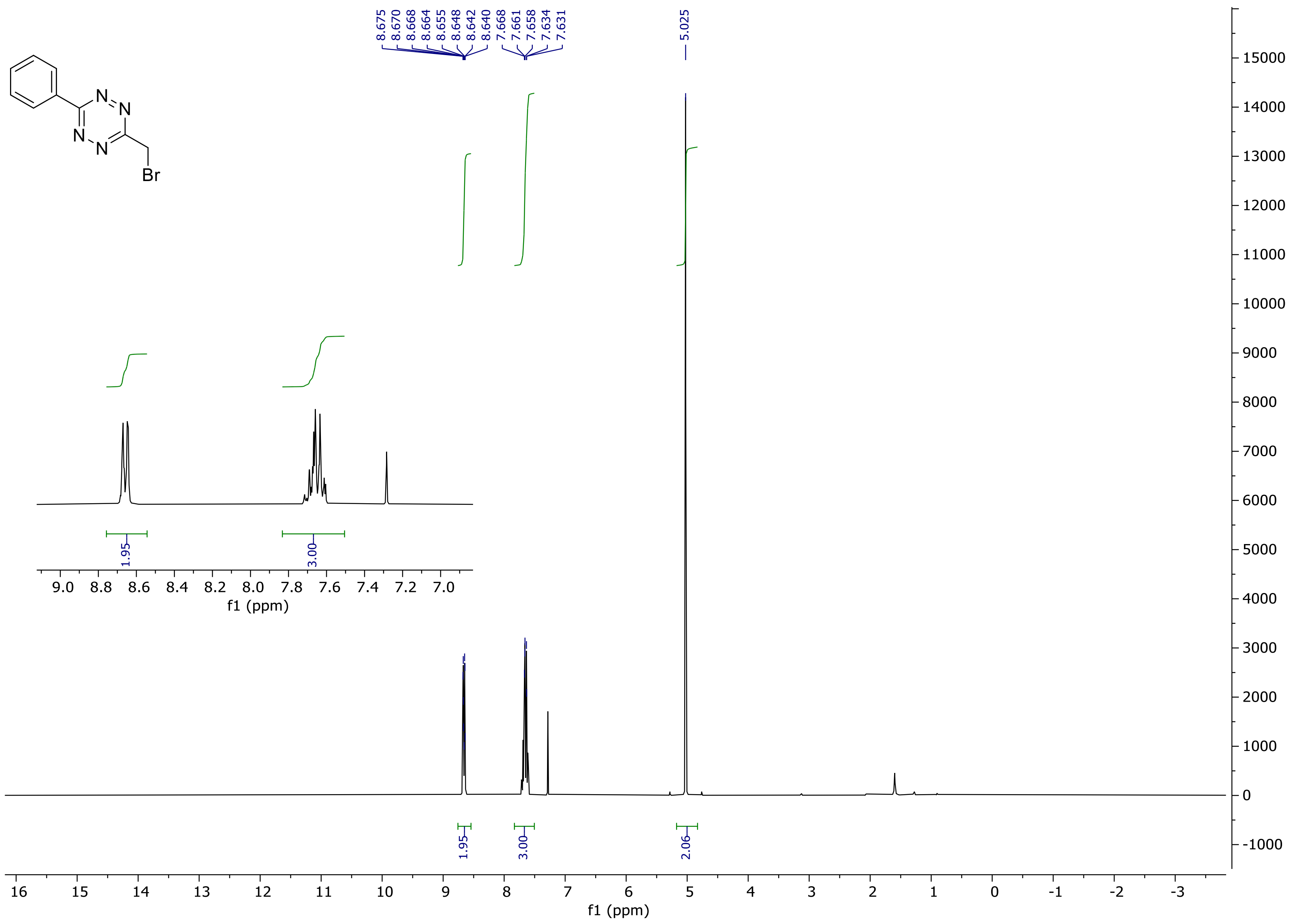

**$^{13}C$ NMR compound 3c**

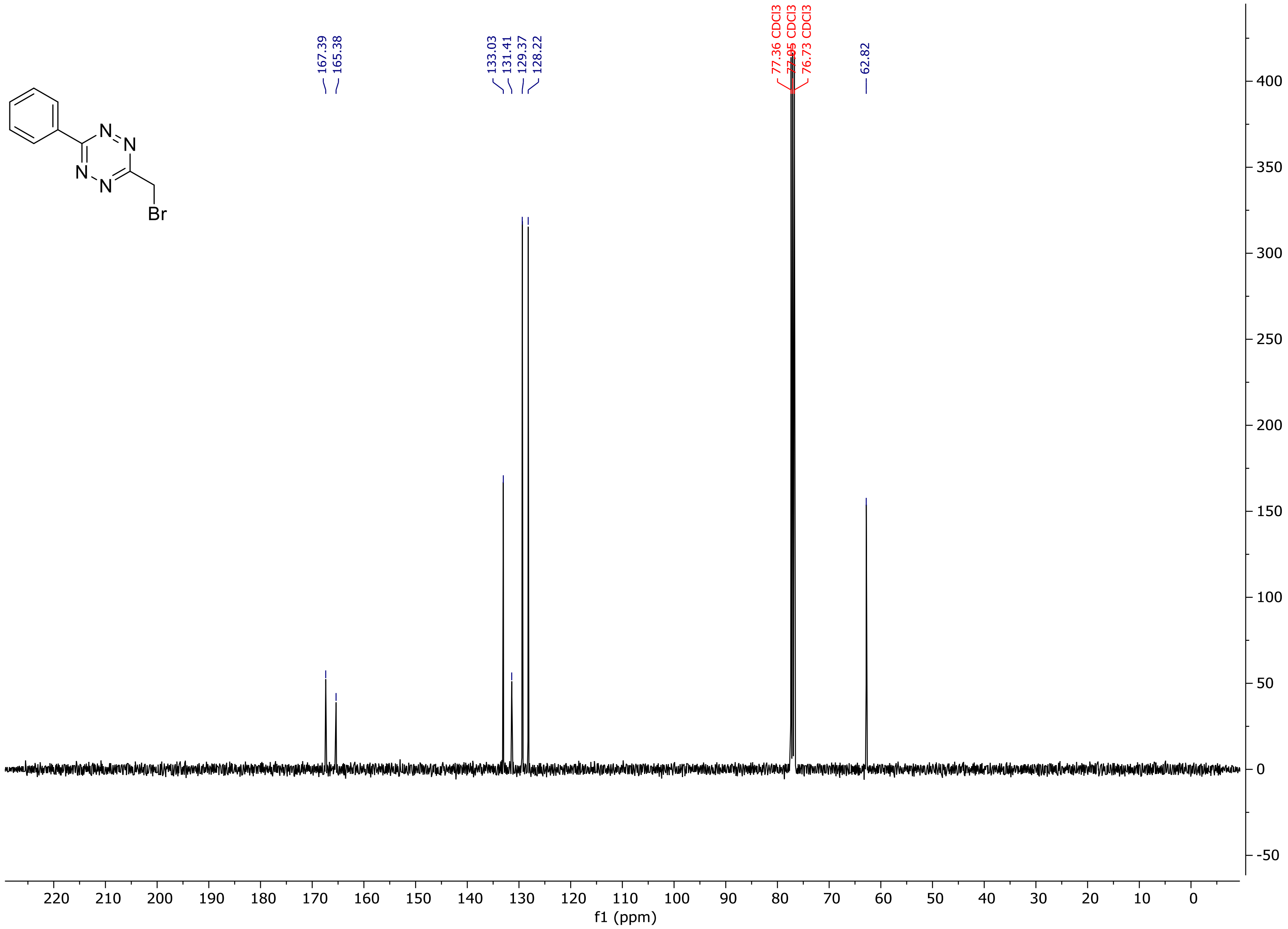

**$^{1}$H NMR compound 5a**

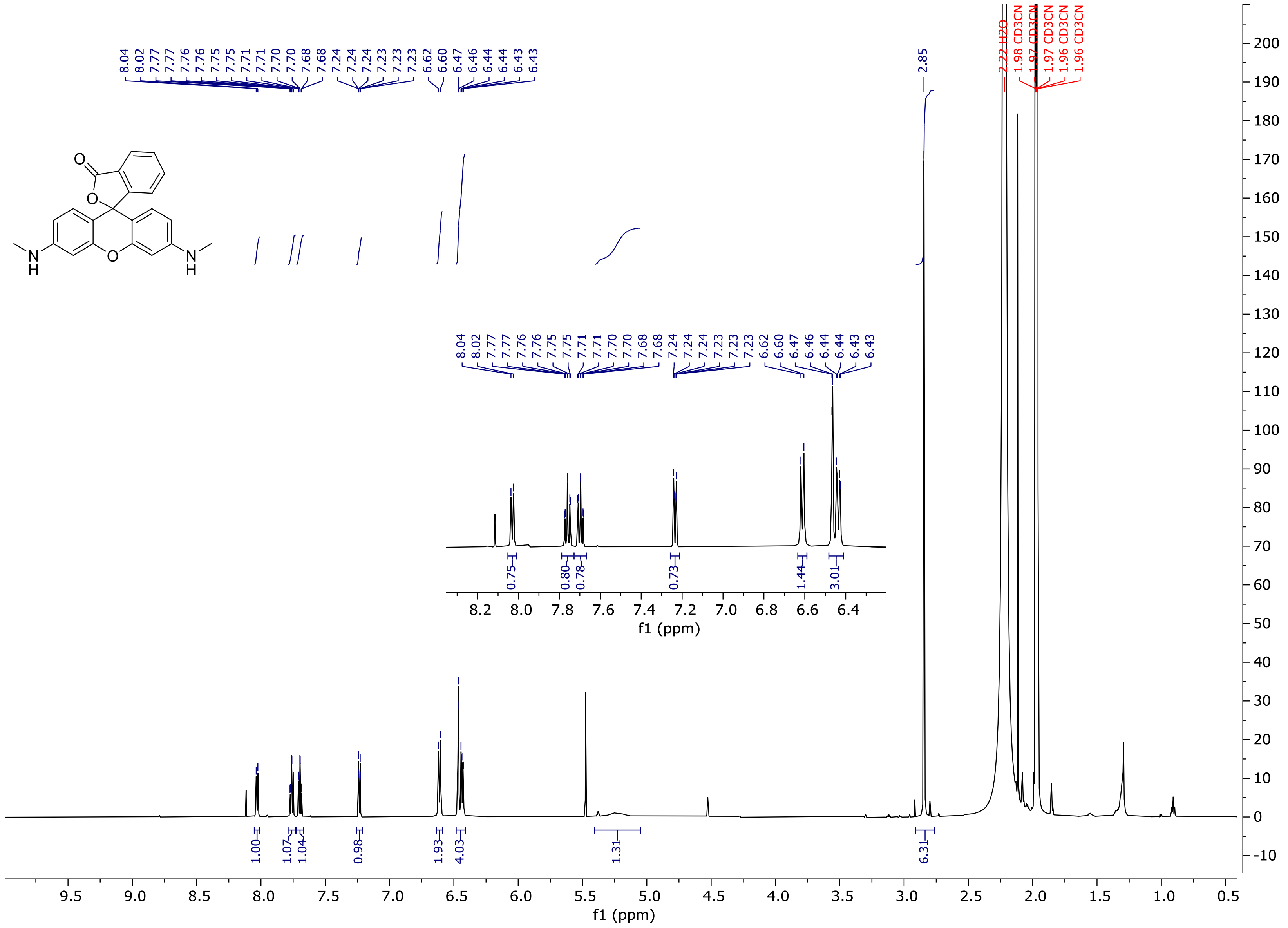

**$^{1}$H NMR compound 5b**

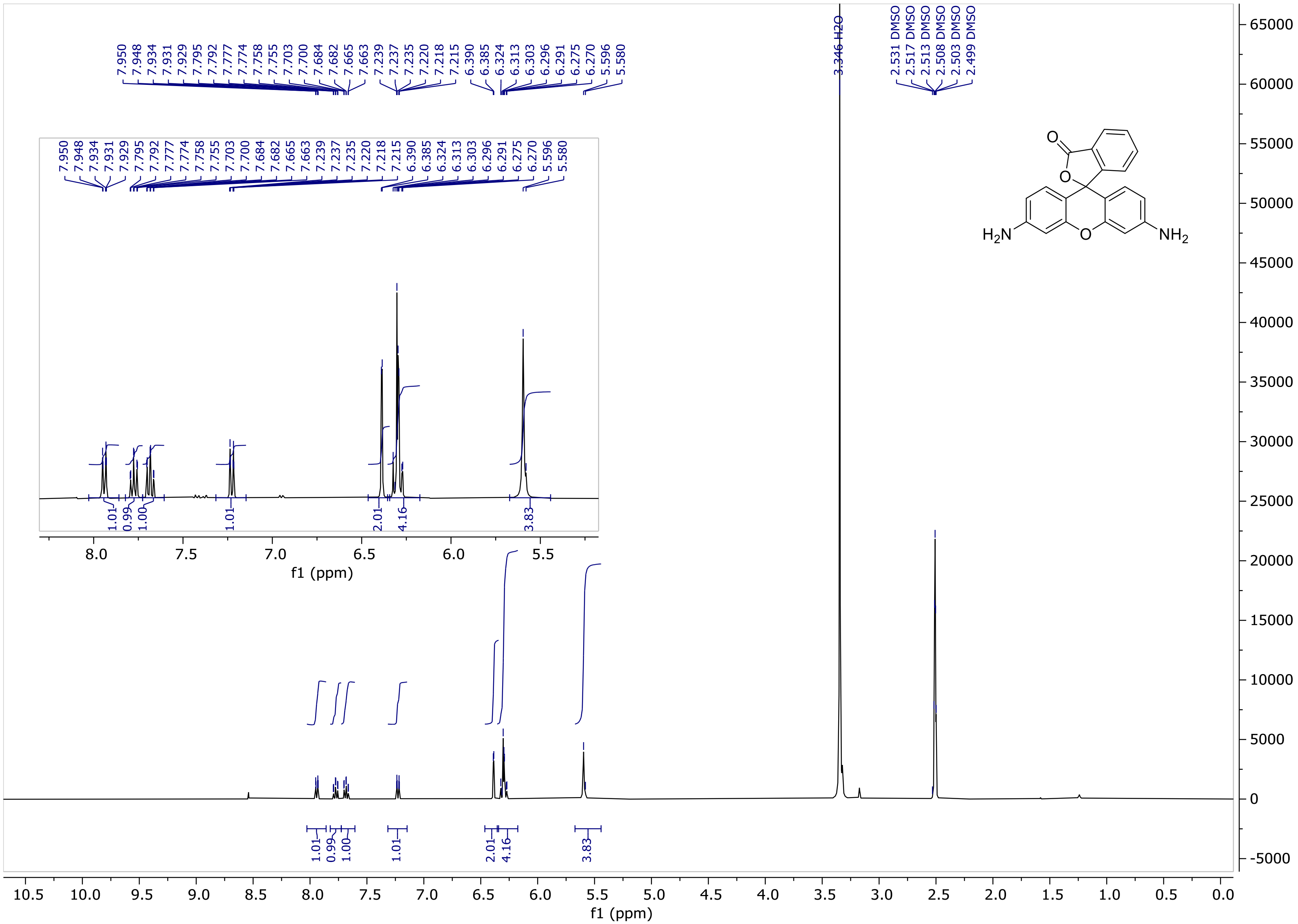

**$^{13}$C NMR compound 5b**

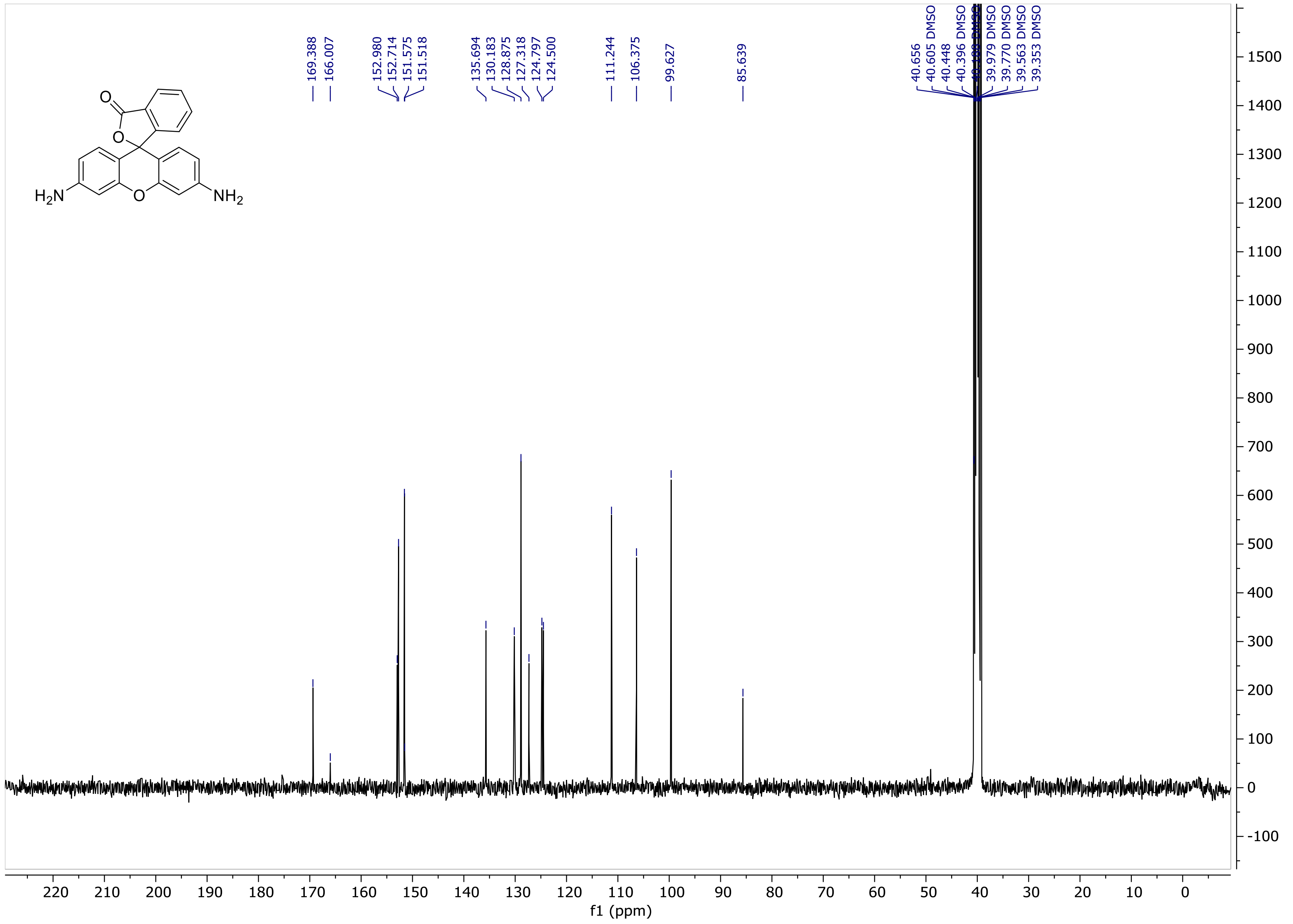

**$^{1}$H NMR compound 9**

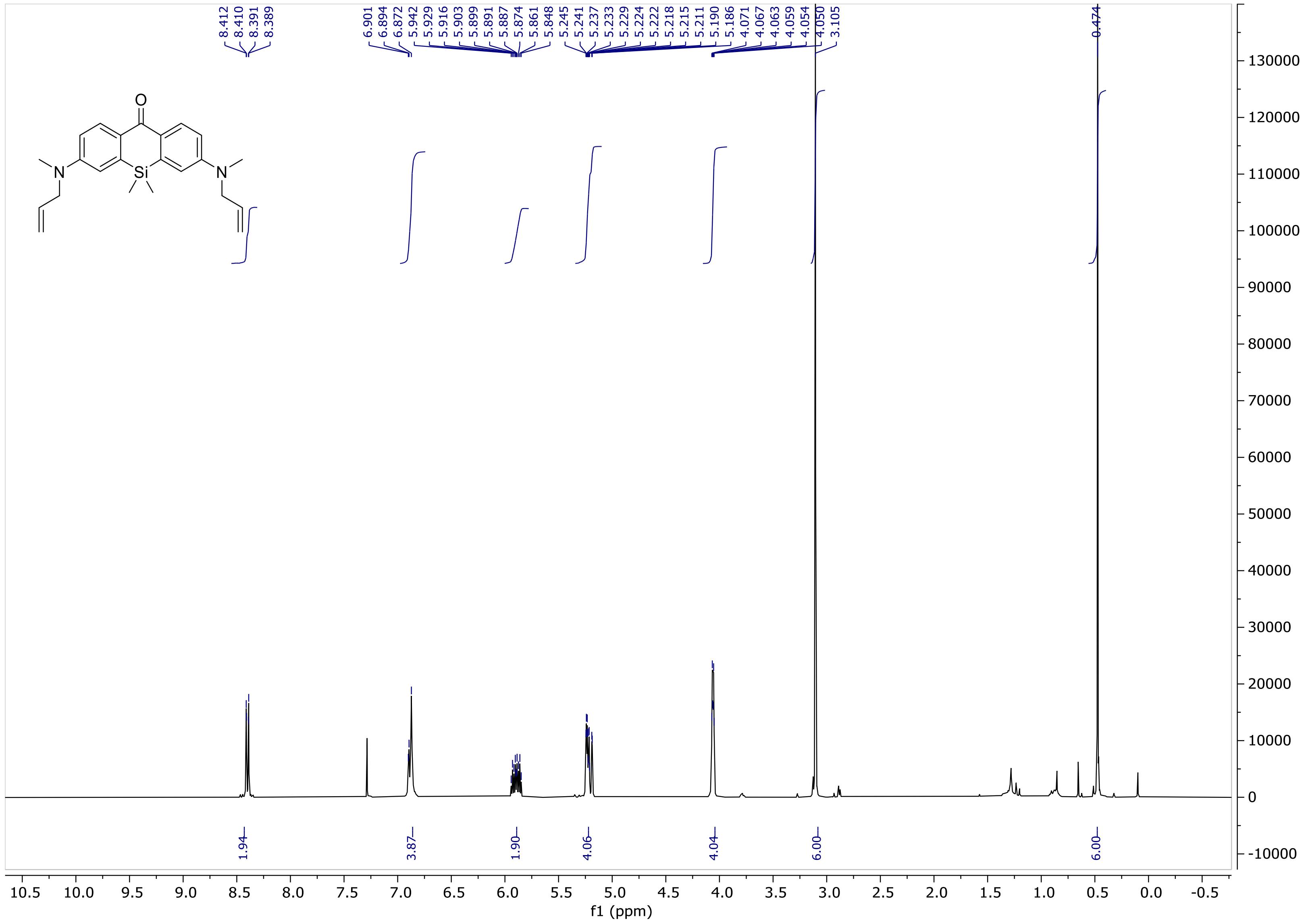

**$^{13}$C NMR compound 9**

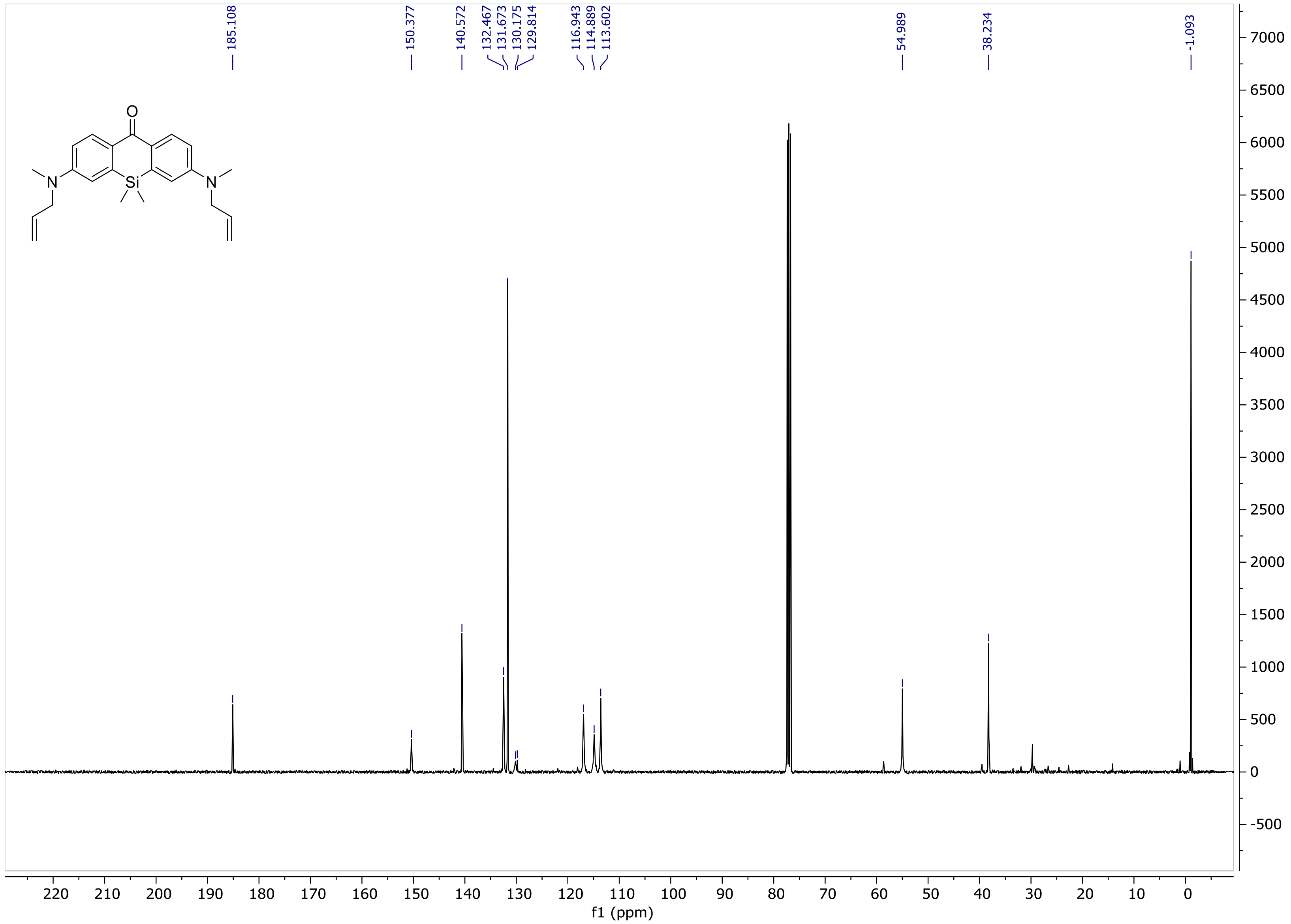

**$^{1}$H NMR compound 10**

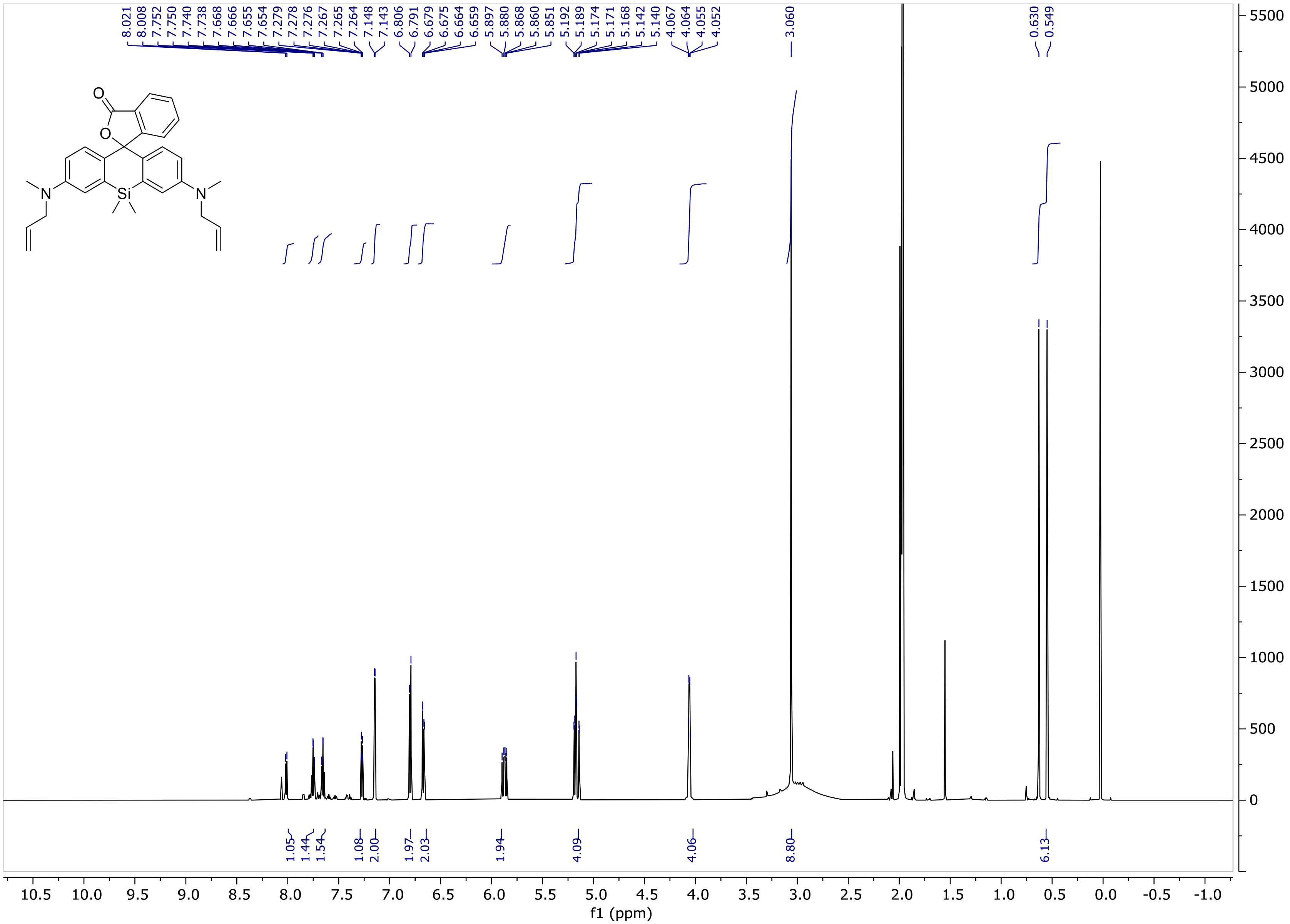

**$^{13}$C NMR compound 10**

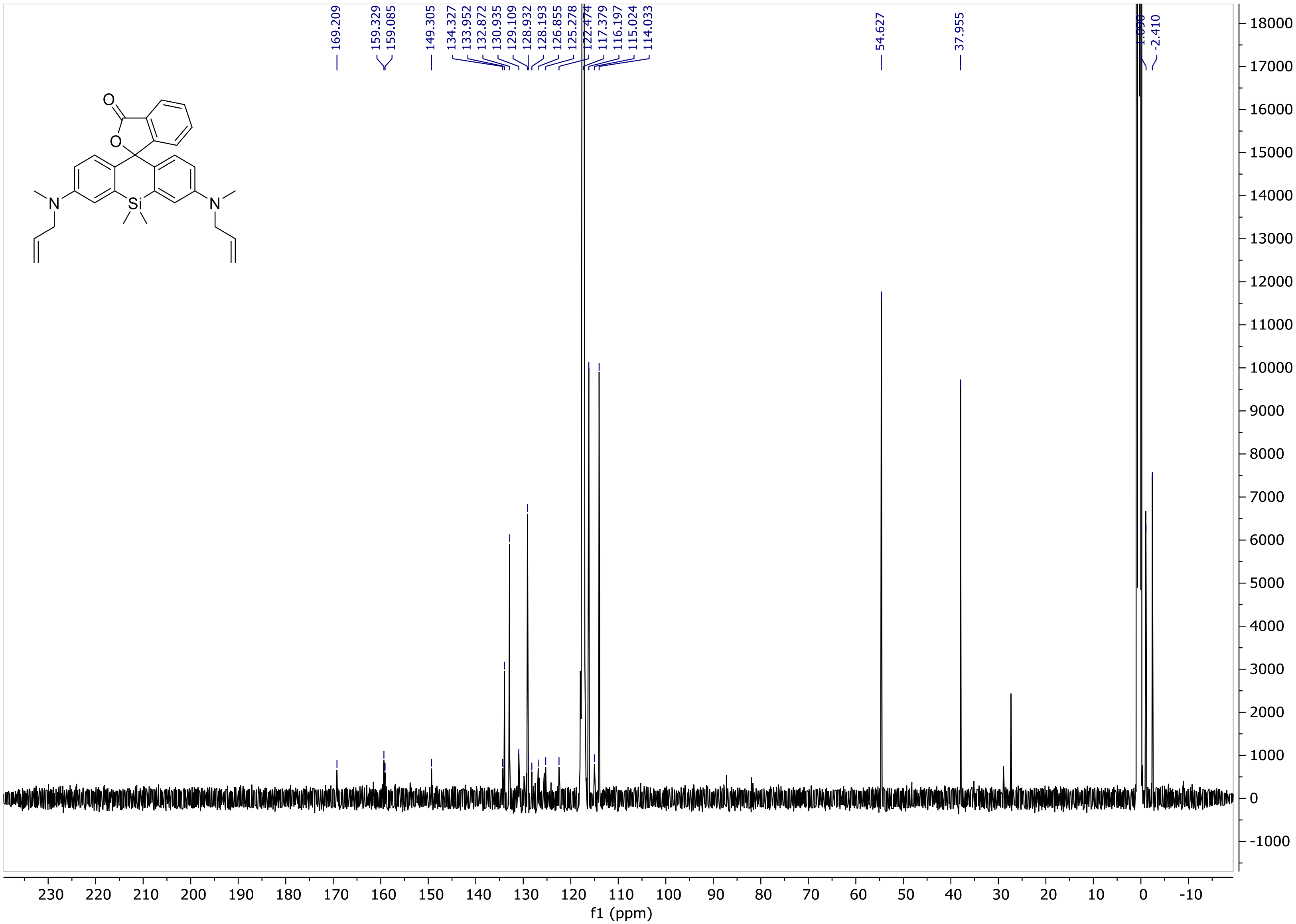

**$^{1}$H NMR compound 11**

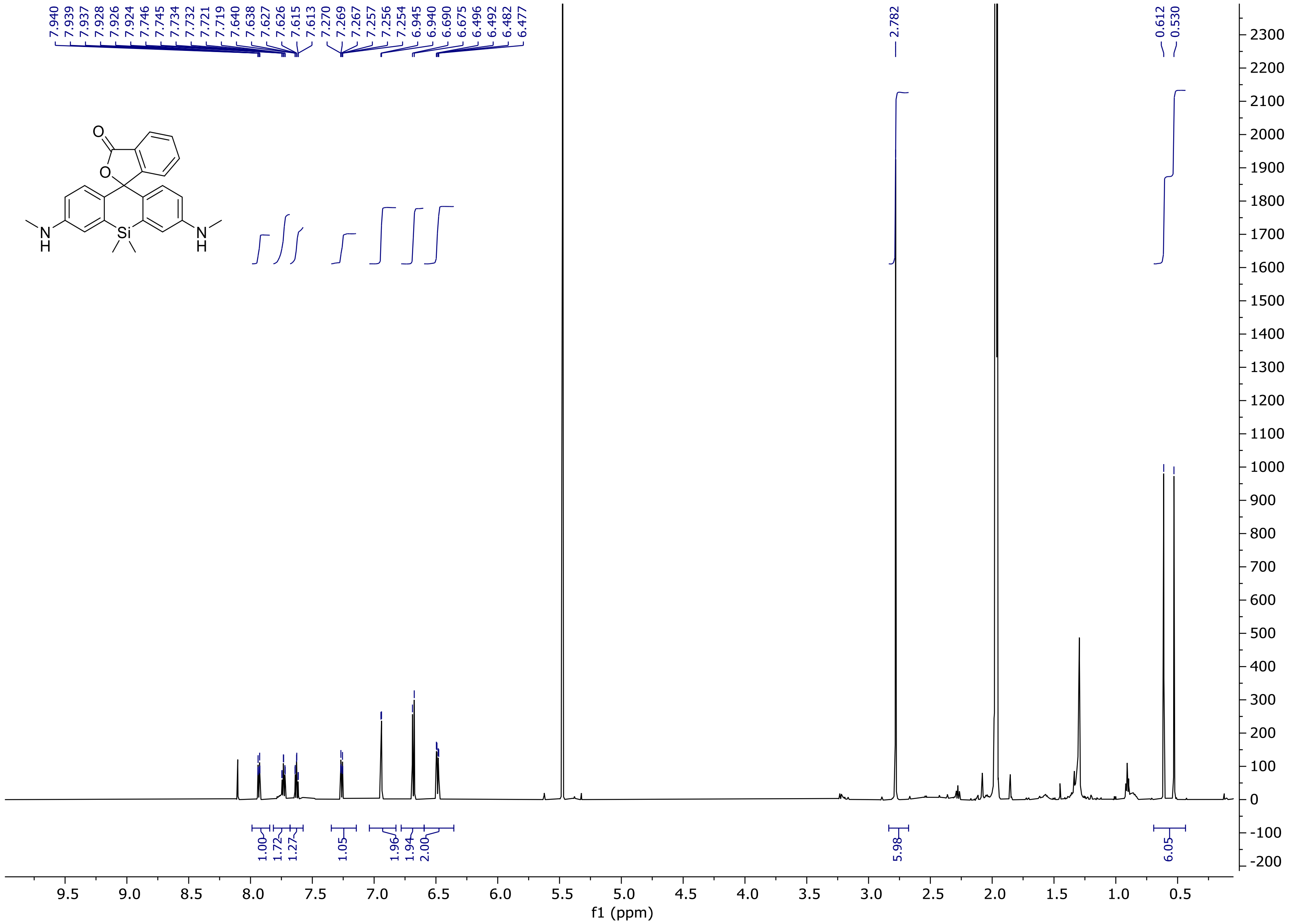

**$^{13}$C NMR compound 11**

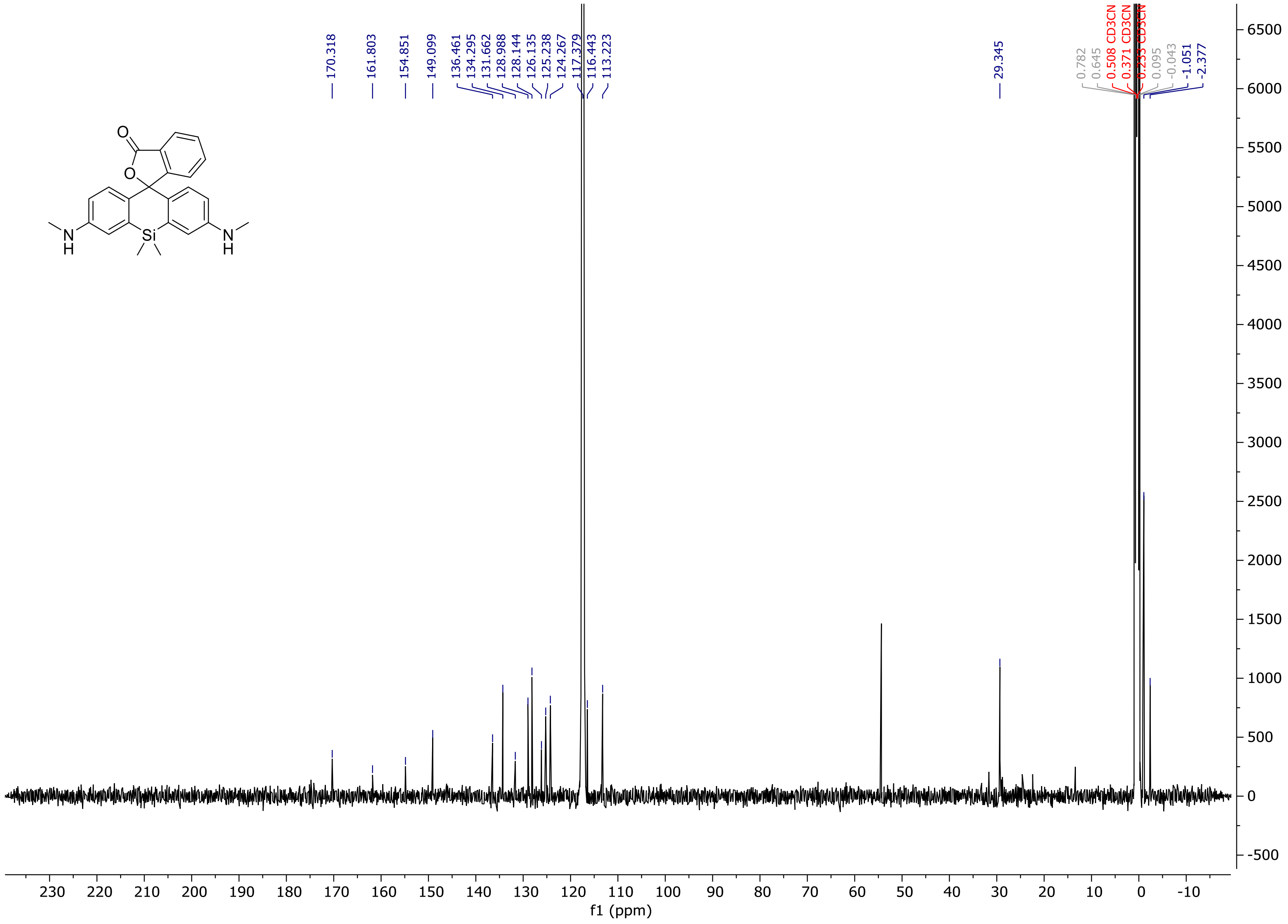

**$^{1}$H NMR compound Rh506monoCTet**

(in MeOD the open form is favorite)

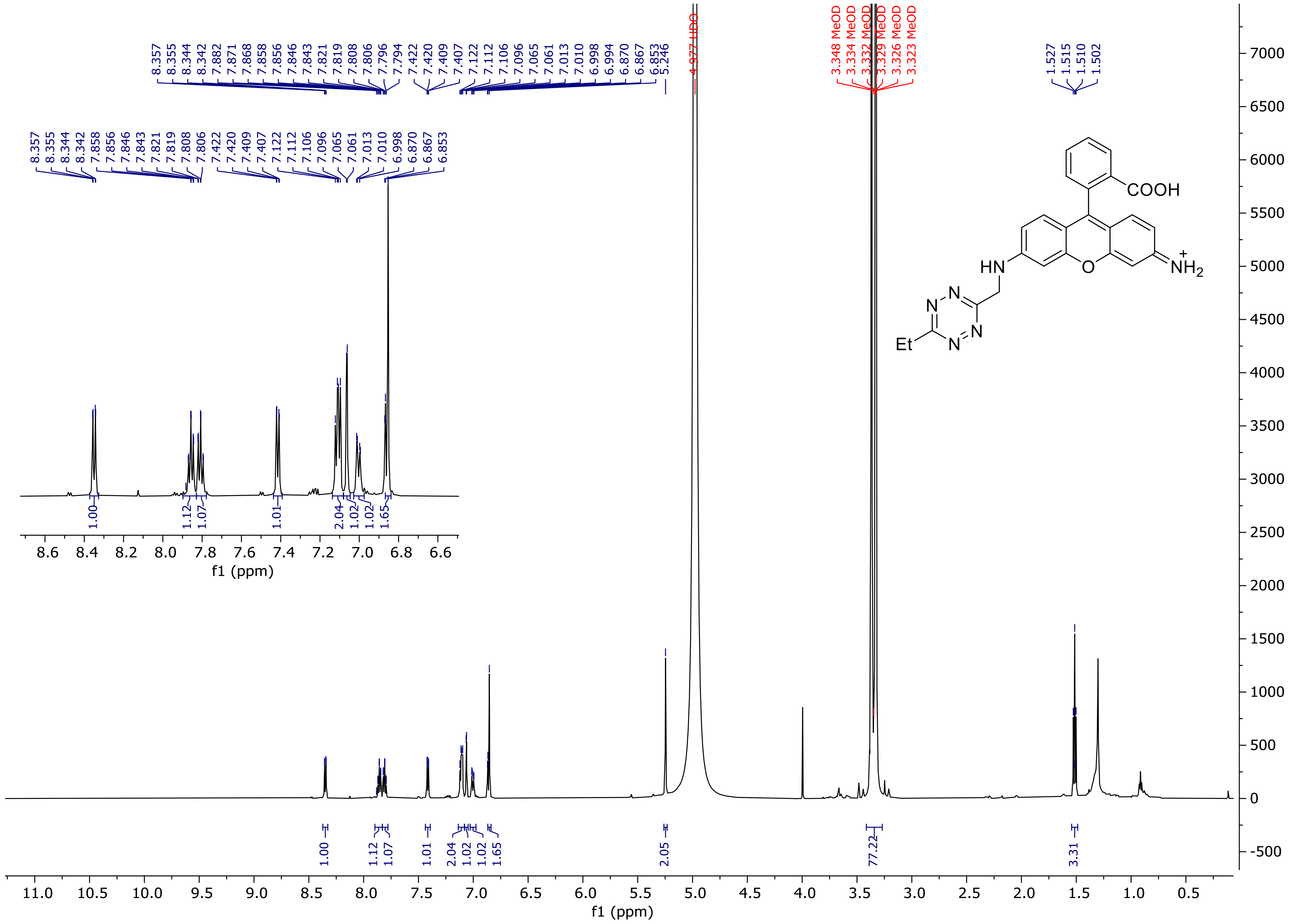

**$^{1}H$-$^{13}C$ HMBC 2D-NMR compound Rh506monoCTet**

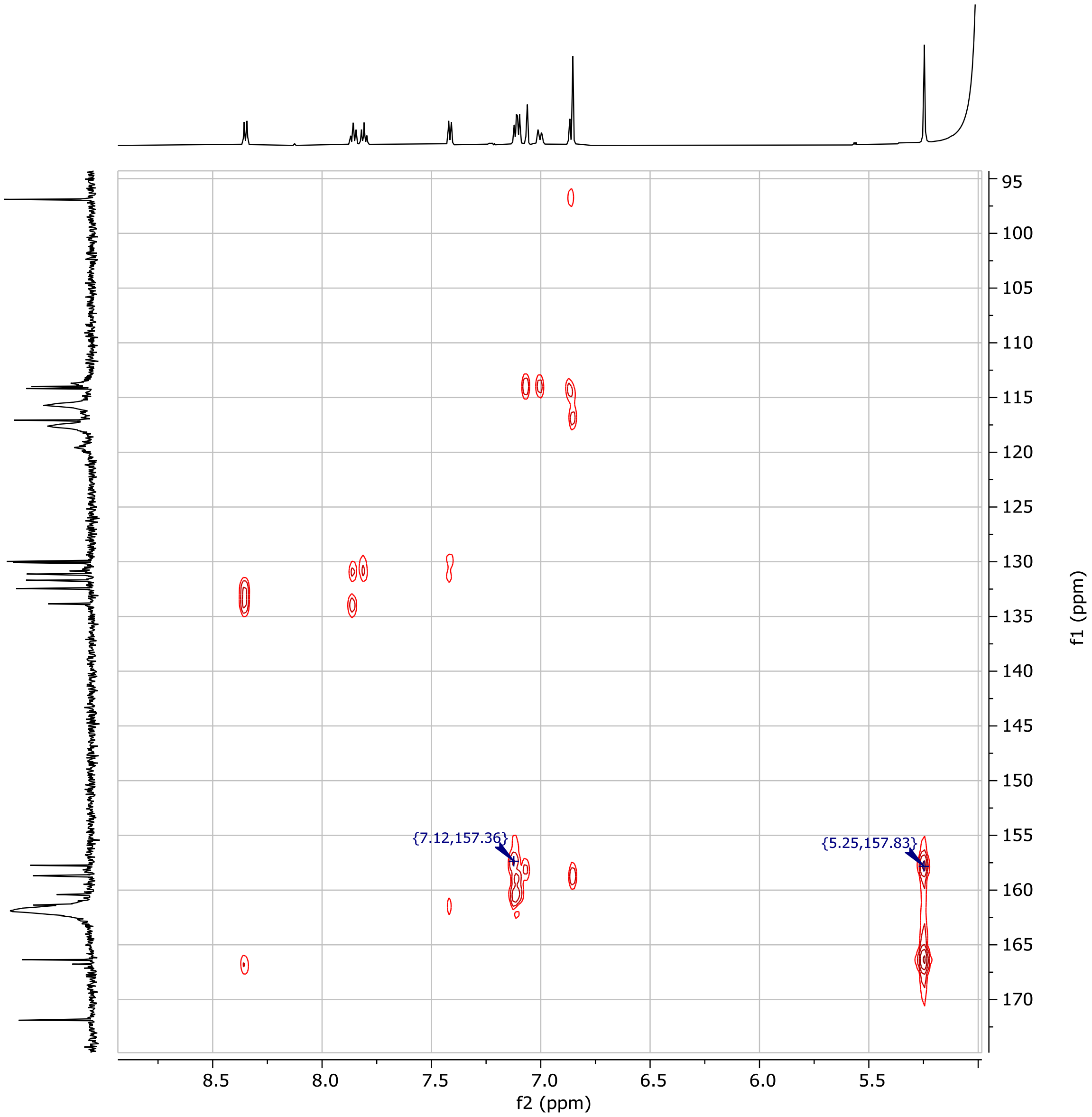


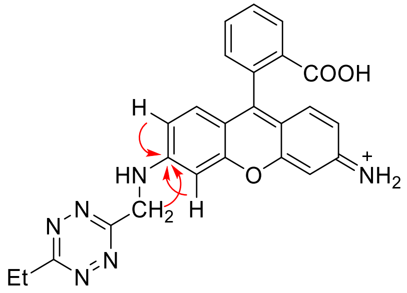

**$^{13}$C NMR compound Rh506monoCTet**

(in MeOD the open form is favorite)

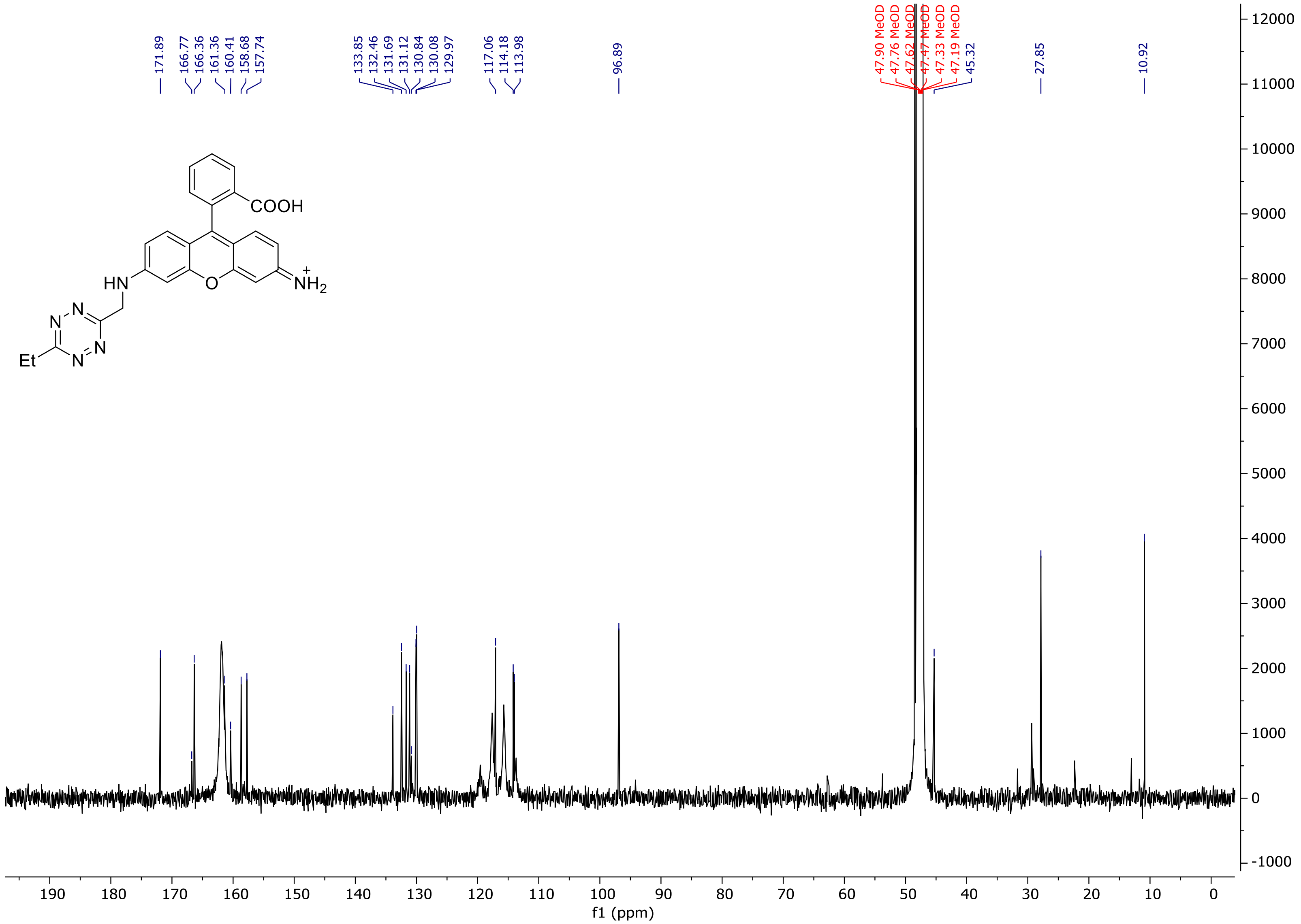

**$^{1}$H NMR compound Rh518bisCTet**

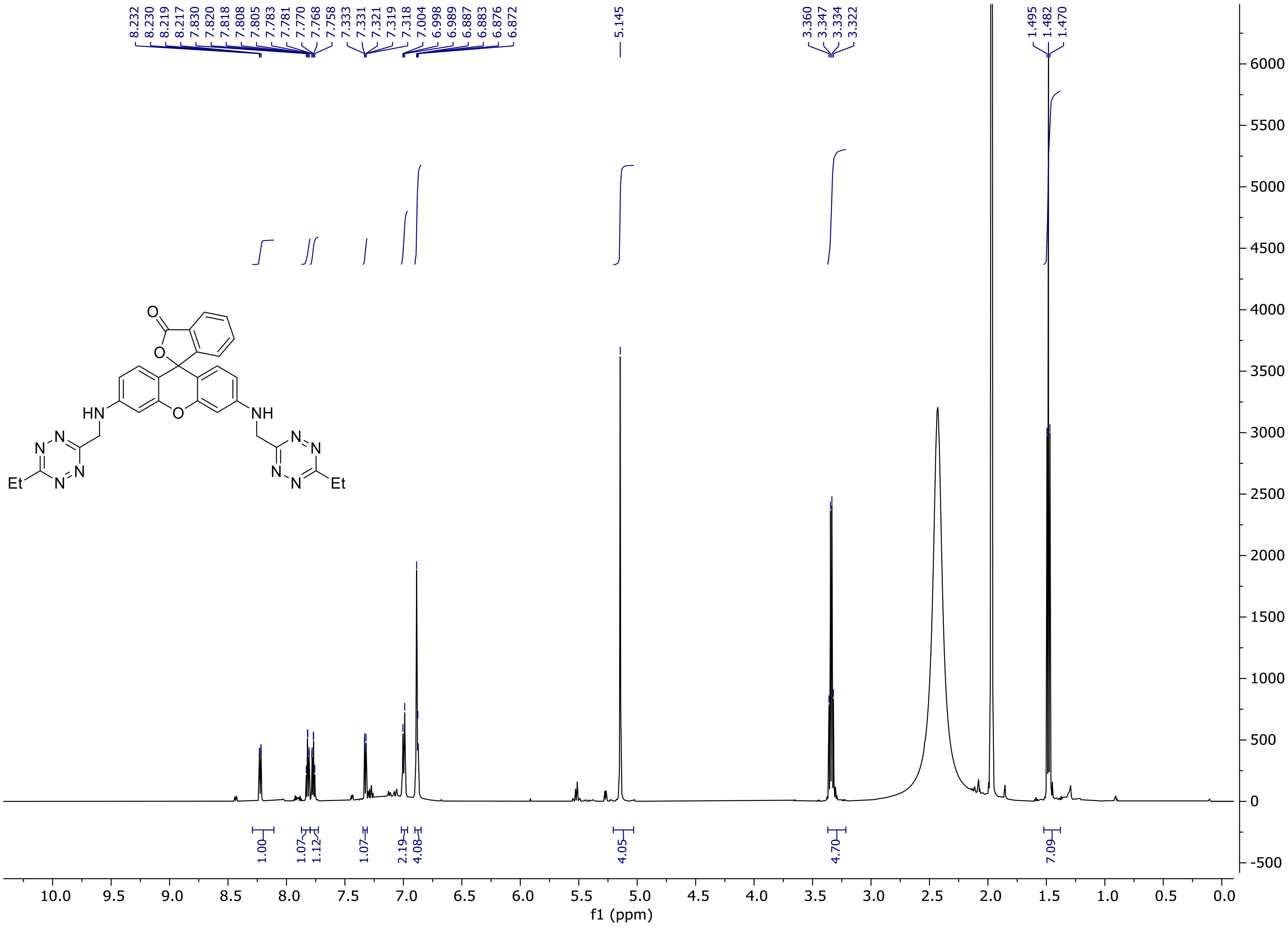

**$^{1}H$-$^{13}C$ HMBC 2D-NMR compound Rh518bisCTet**

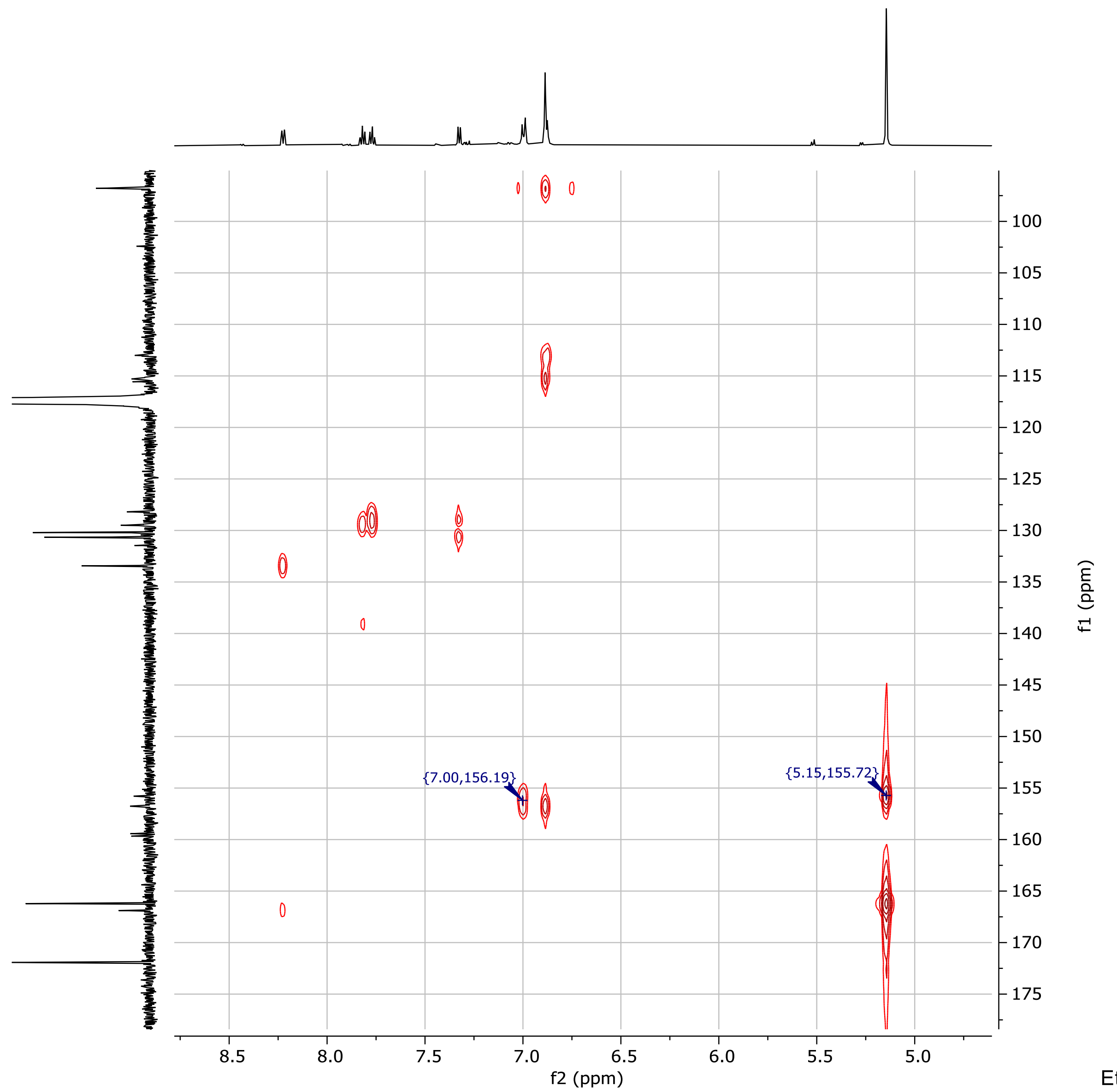


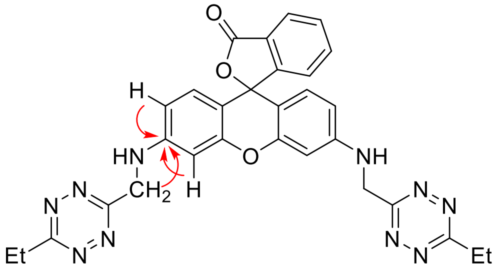

**$^{13}$C NMR compound Rh518bisCTet**

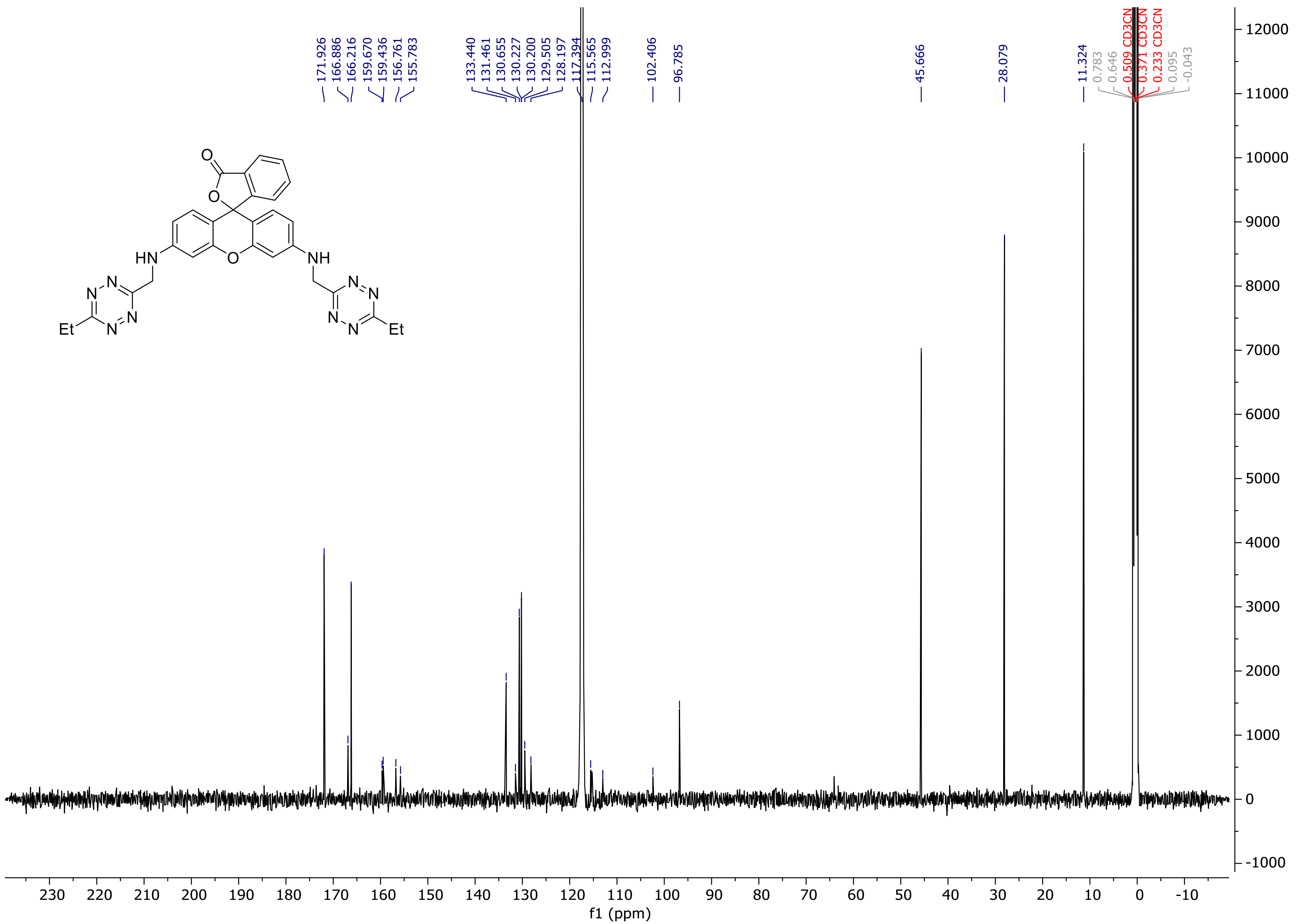

**$^{1}$H NMR compound Rh528monoCTet**

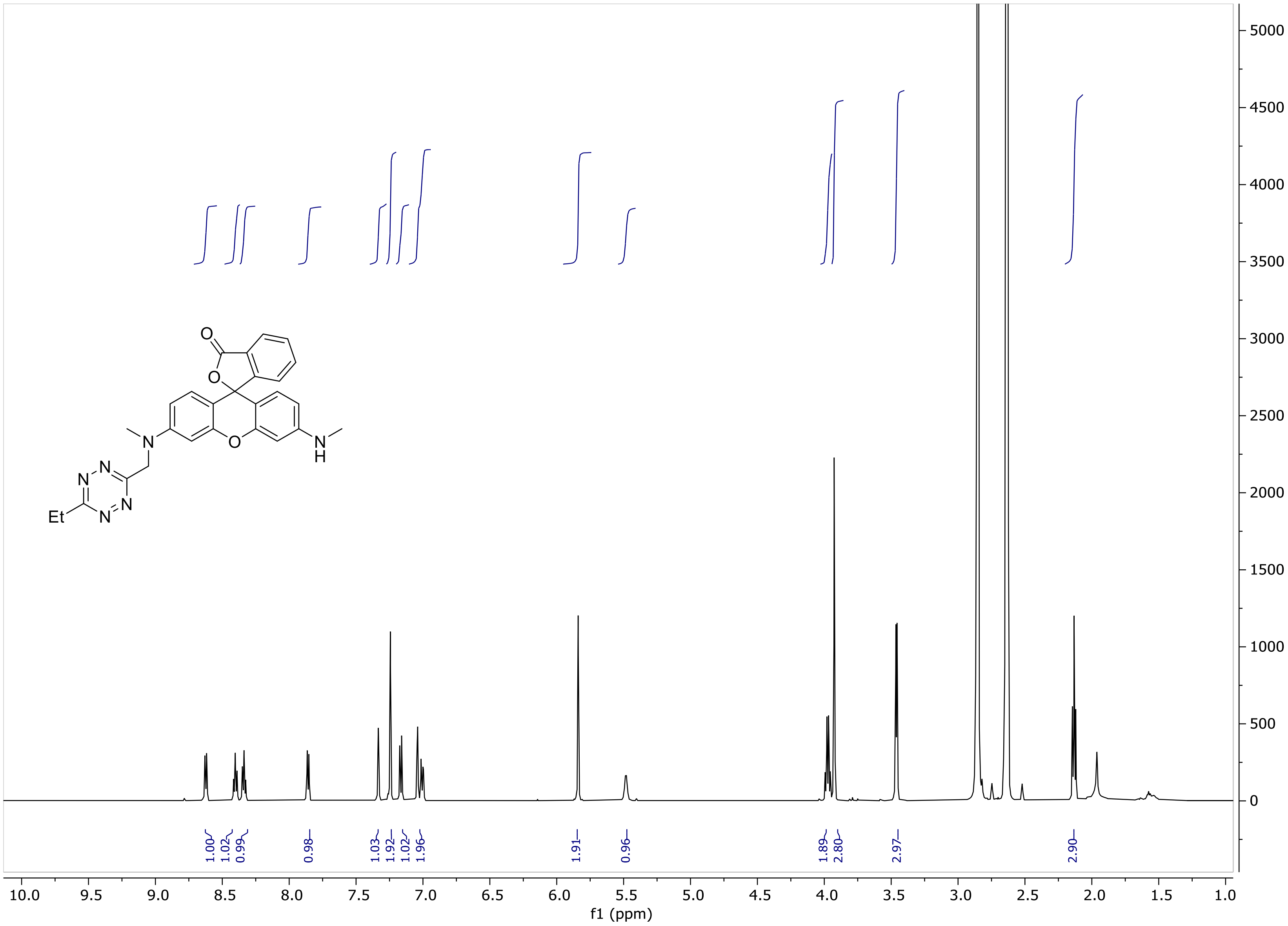

$^{1}$H-$^{13}$C HMBC 2D-NMR compound Rh528monoCTet

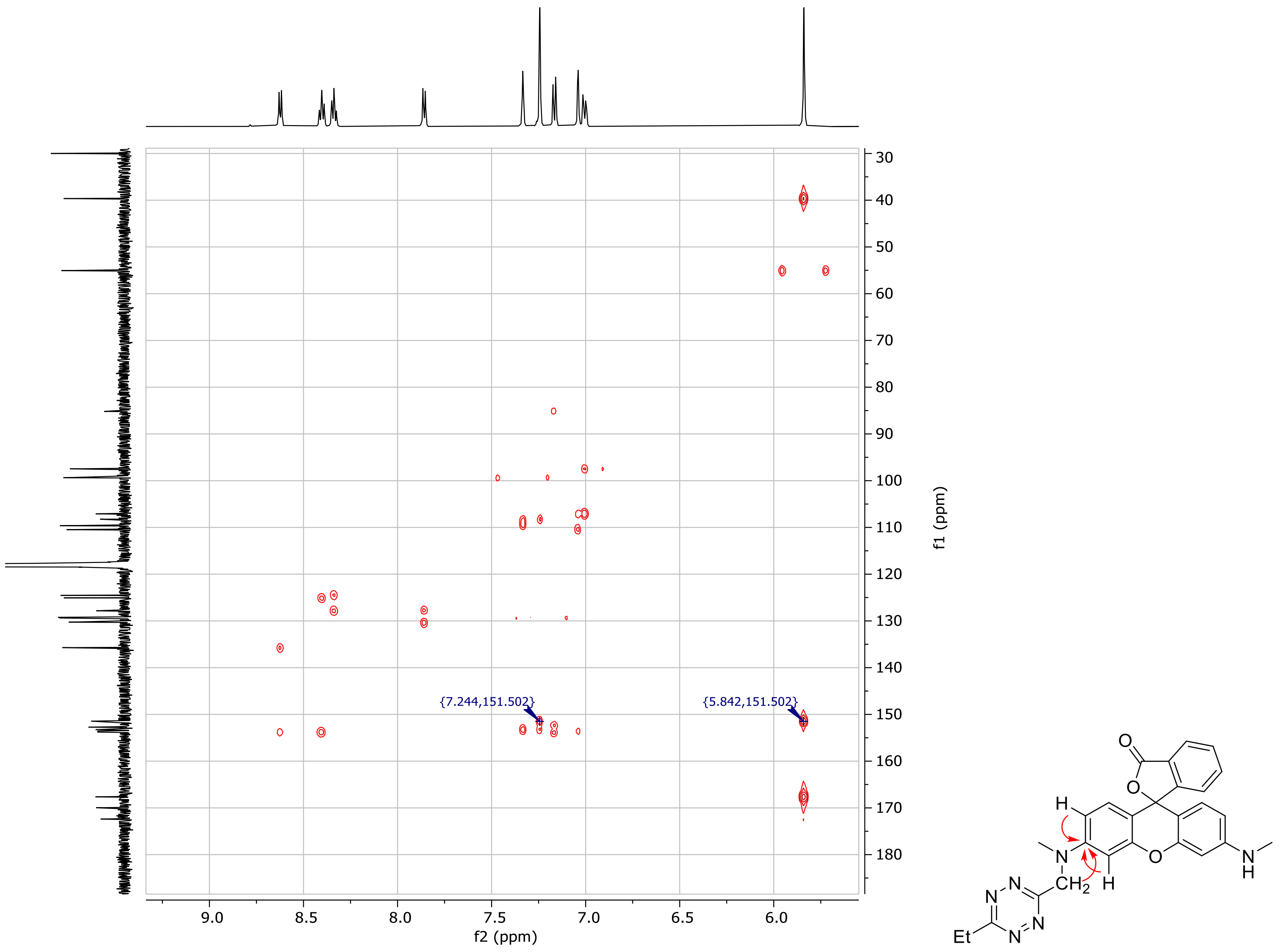

**$^{13}$C NMR compound Rh528monoCTet**

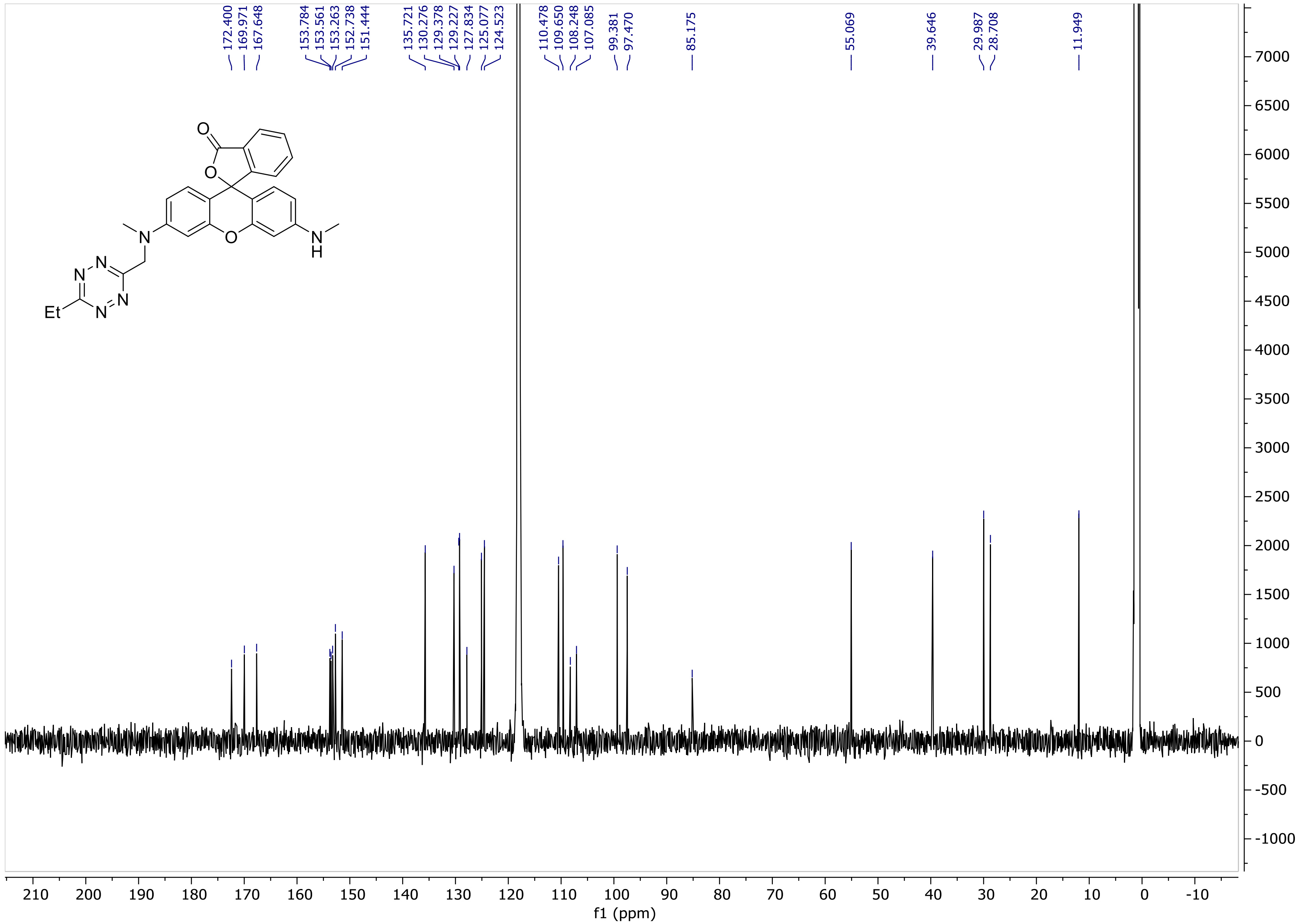

**$^{1}$H NMR compound Rh538bisCTet**

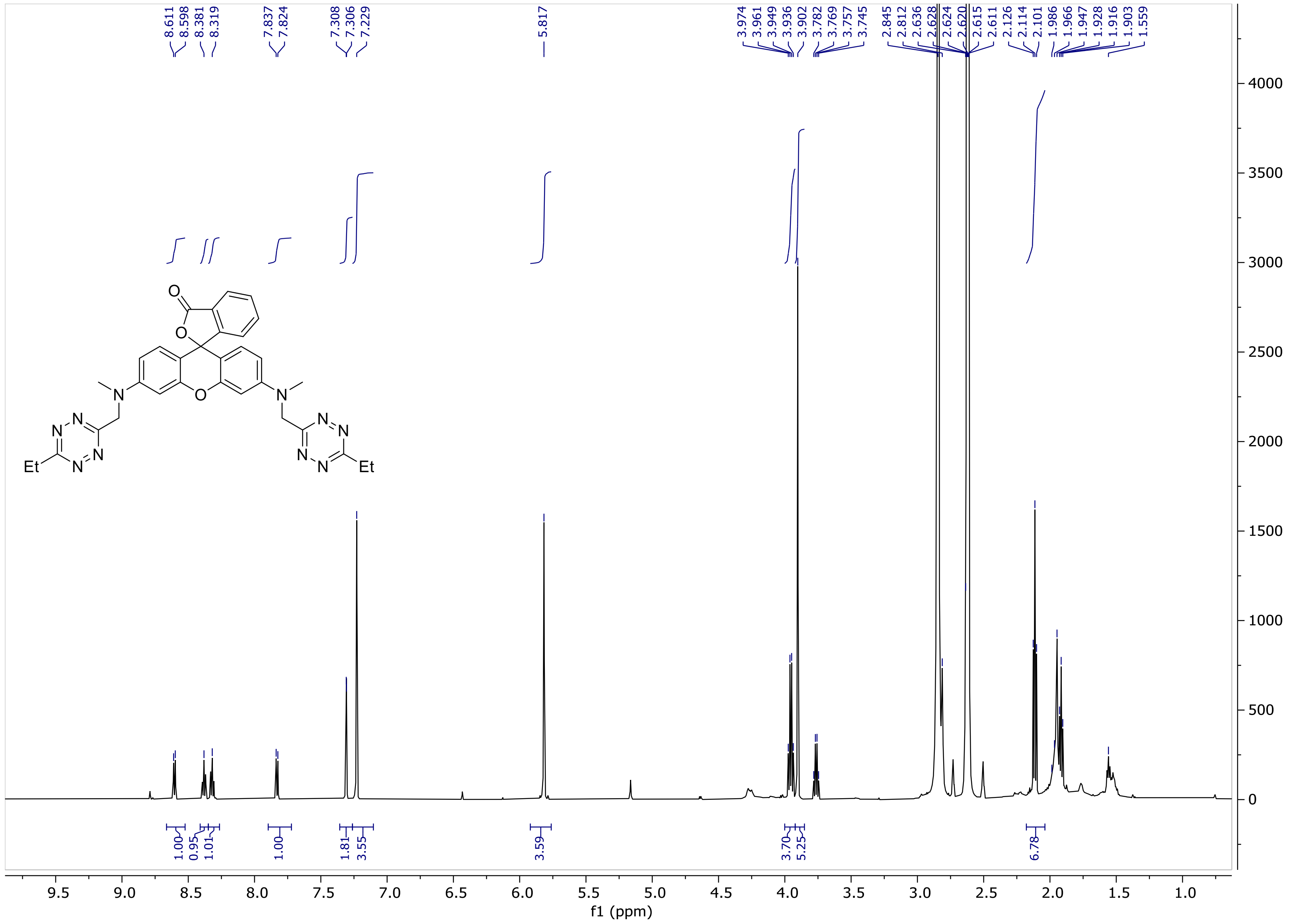

**$^{1}H$-$^{13}C$ HMBC 2D-NMR compound Rh538bisCTet**

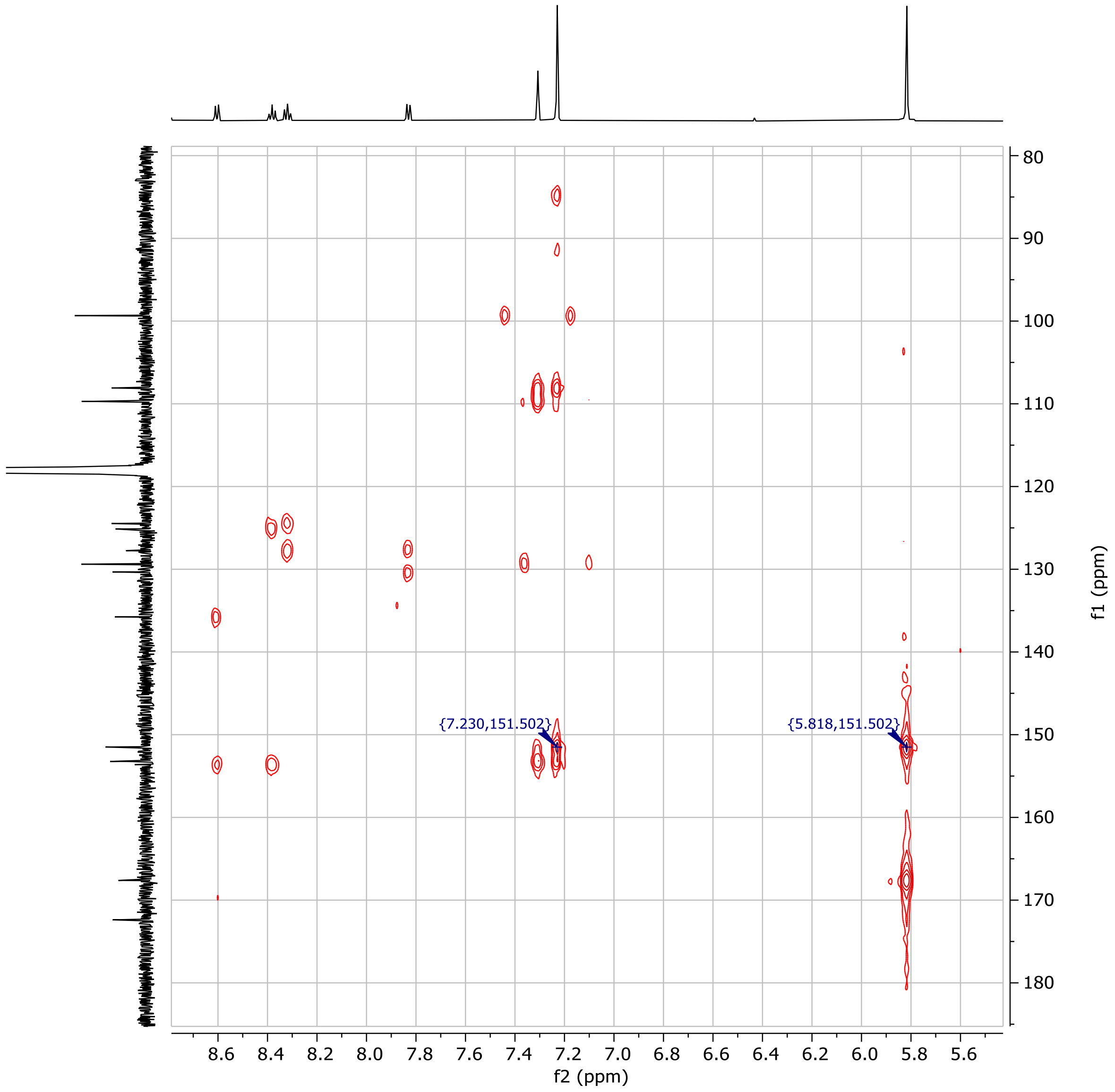


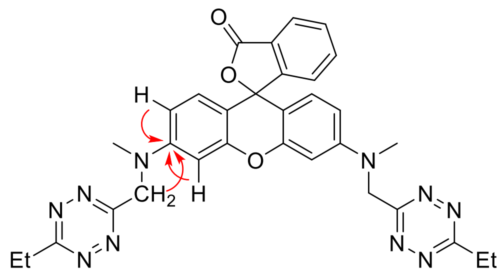

**$^{13}$C NMR compound Rh538bisCTet**

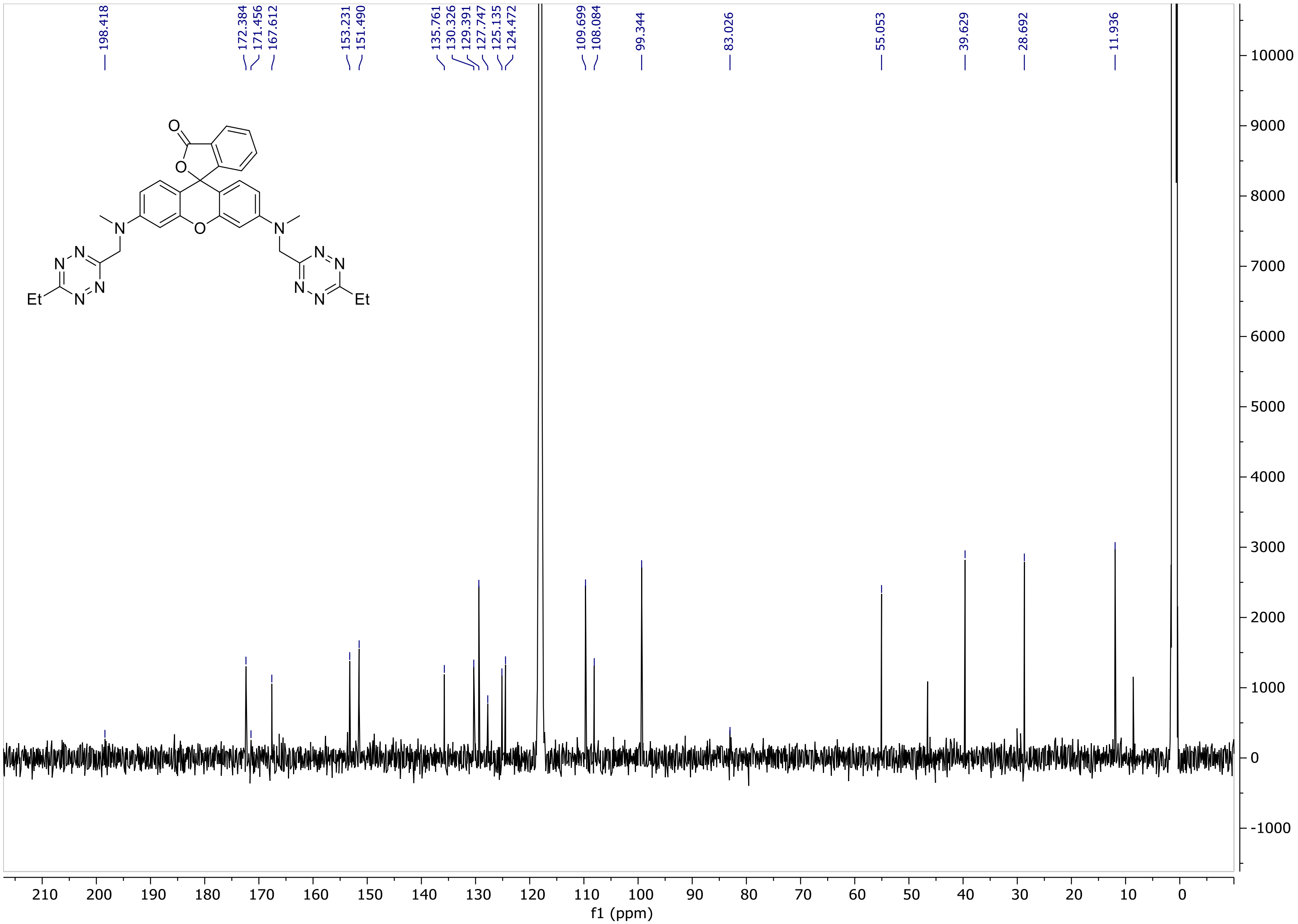

## $^{1}$H NMR compound SiRh628monoCTet

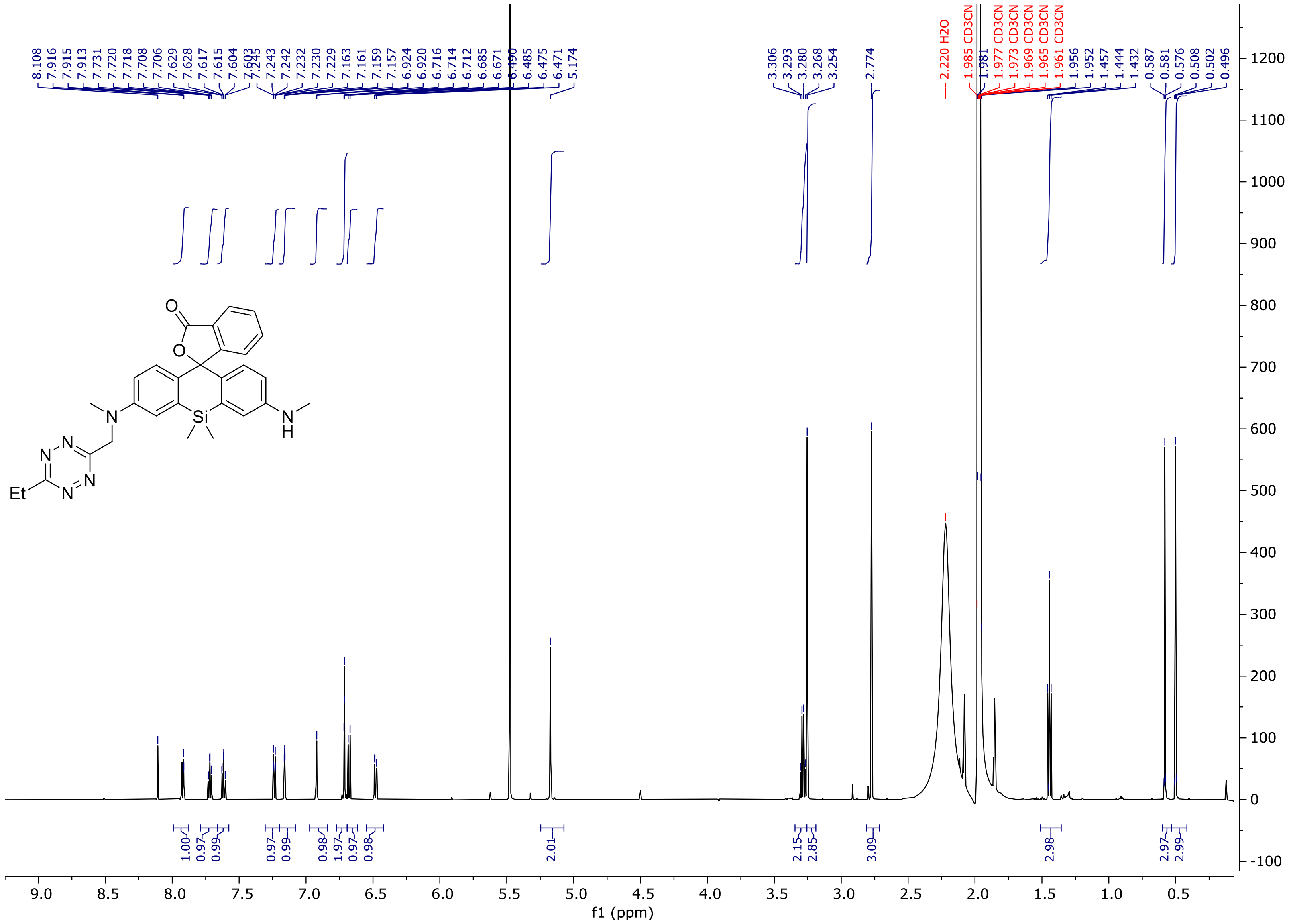

**$^{1}H$-$^{13}C$ HMBC 2D-NMR compound SiRh628monoCTet**

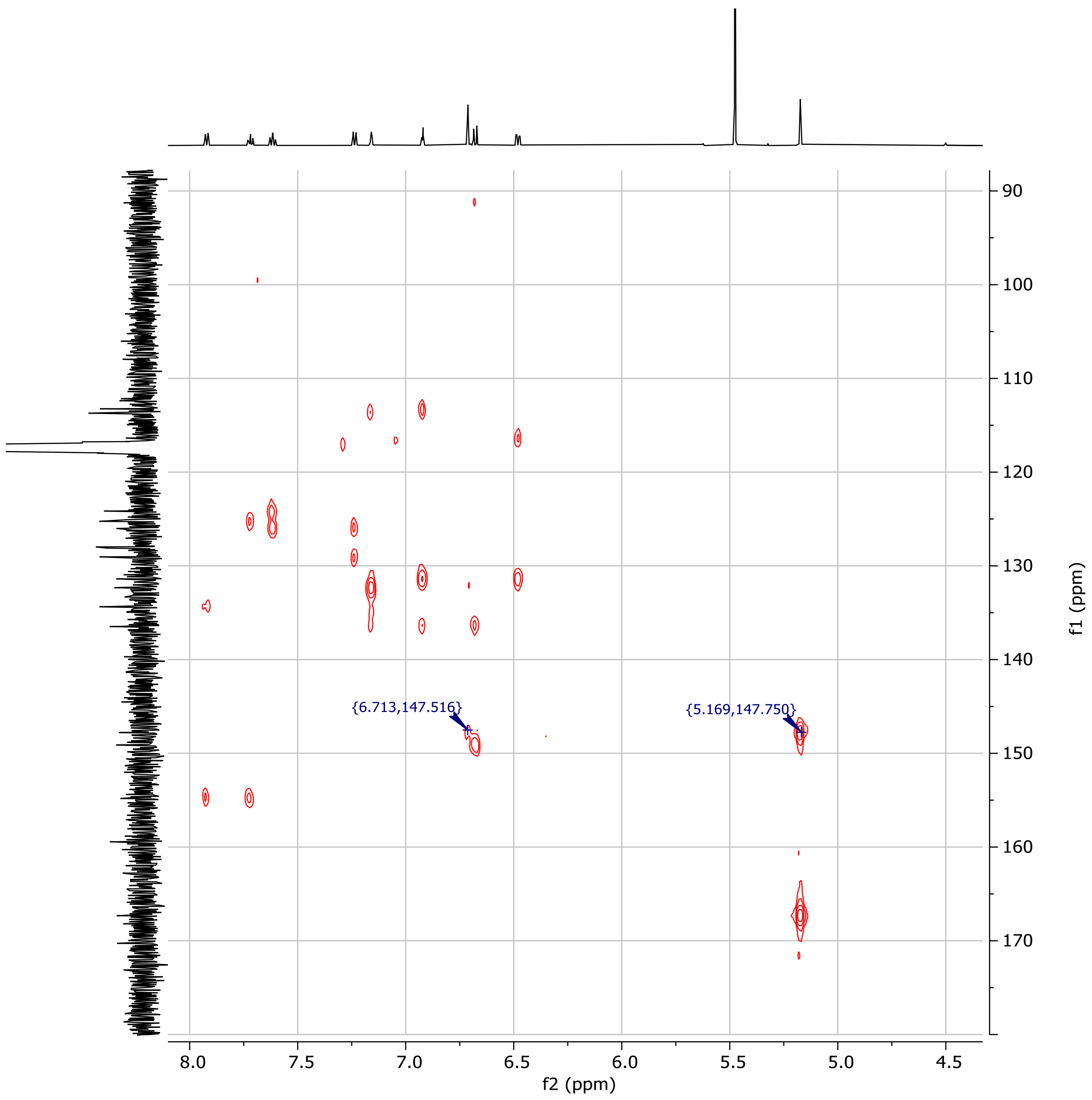


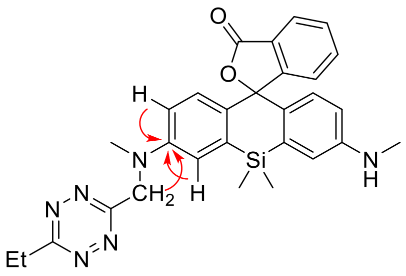

**$^{13}$C NMR compound SiRh628monoCTet**

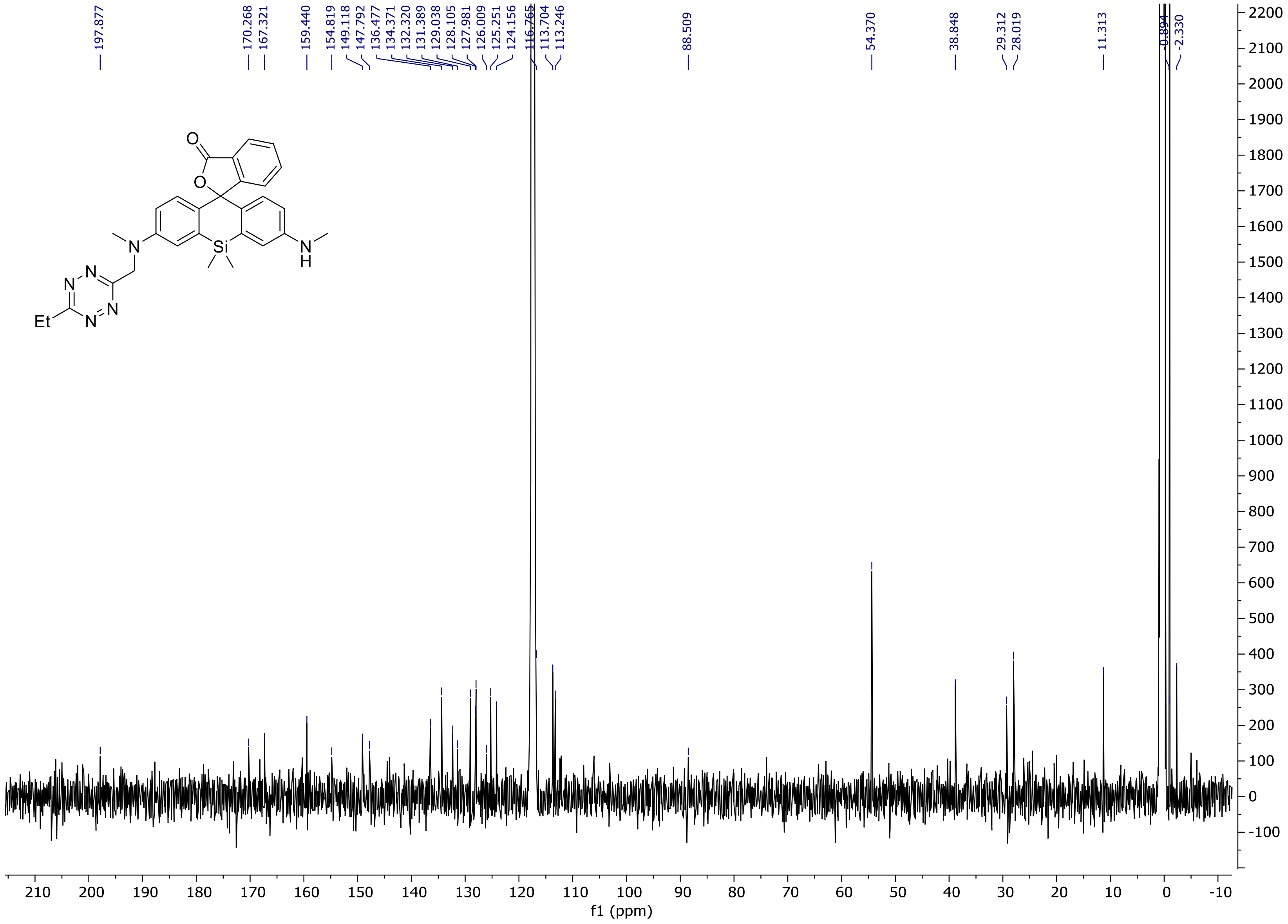

**$^1$H NMR compound SiRh640bisCTet**

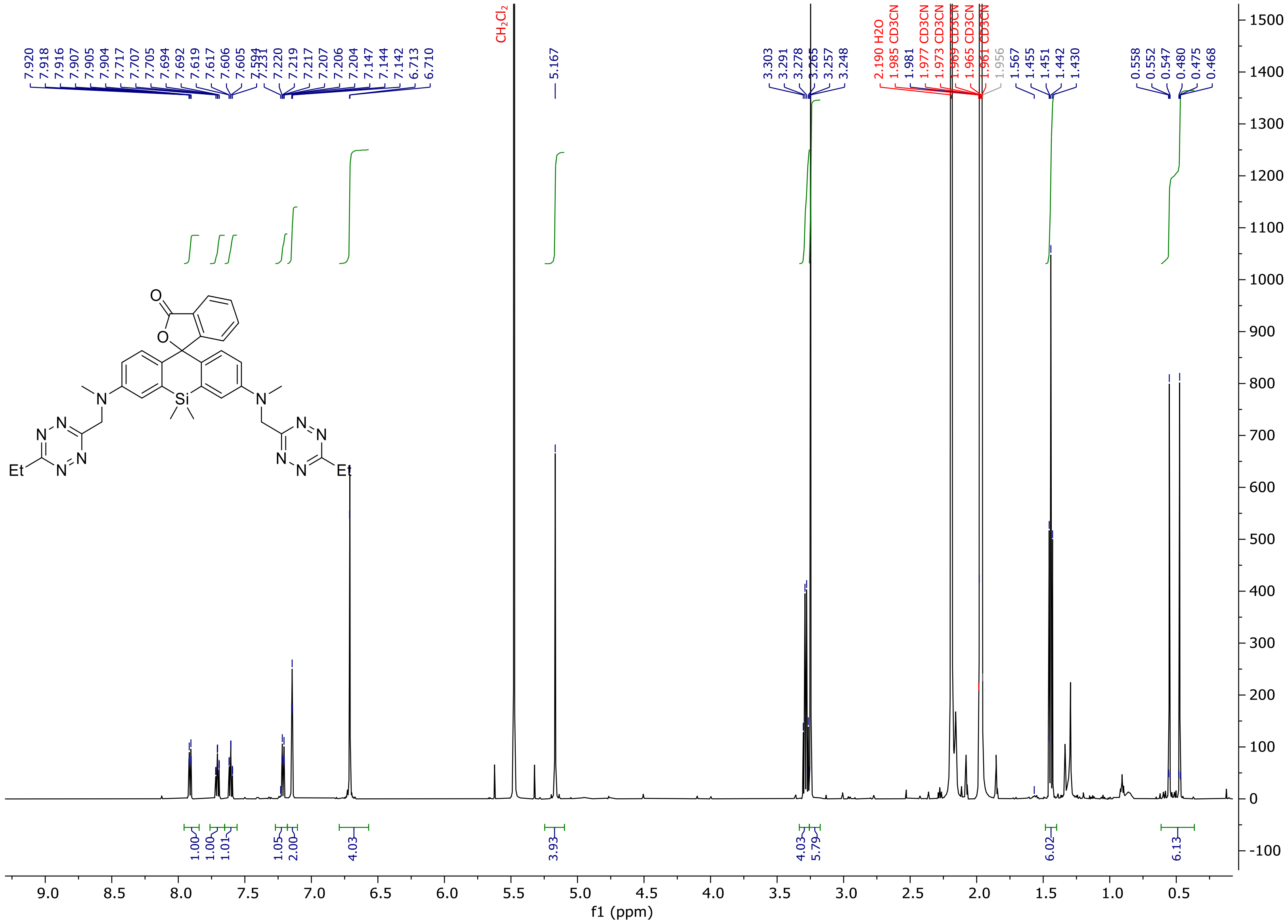

**$^{1}$H-$^{13}$C HMBC 2D-NMR compound SiRh640bisCTet**

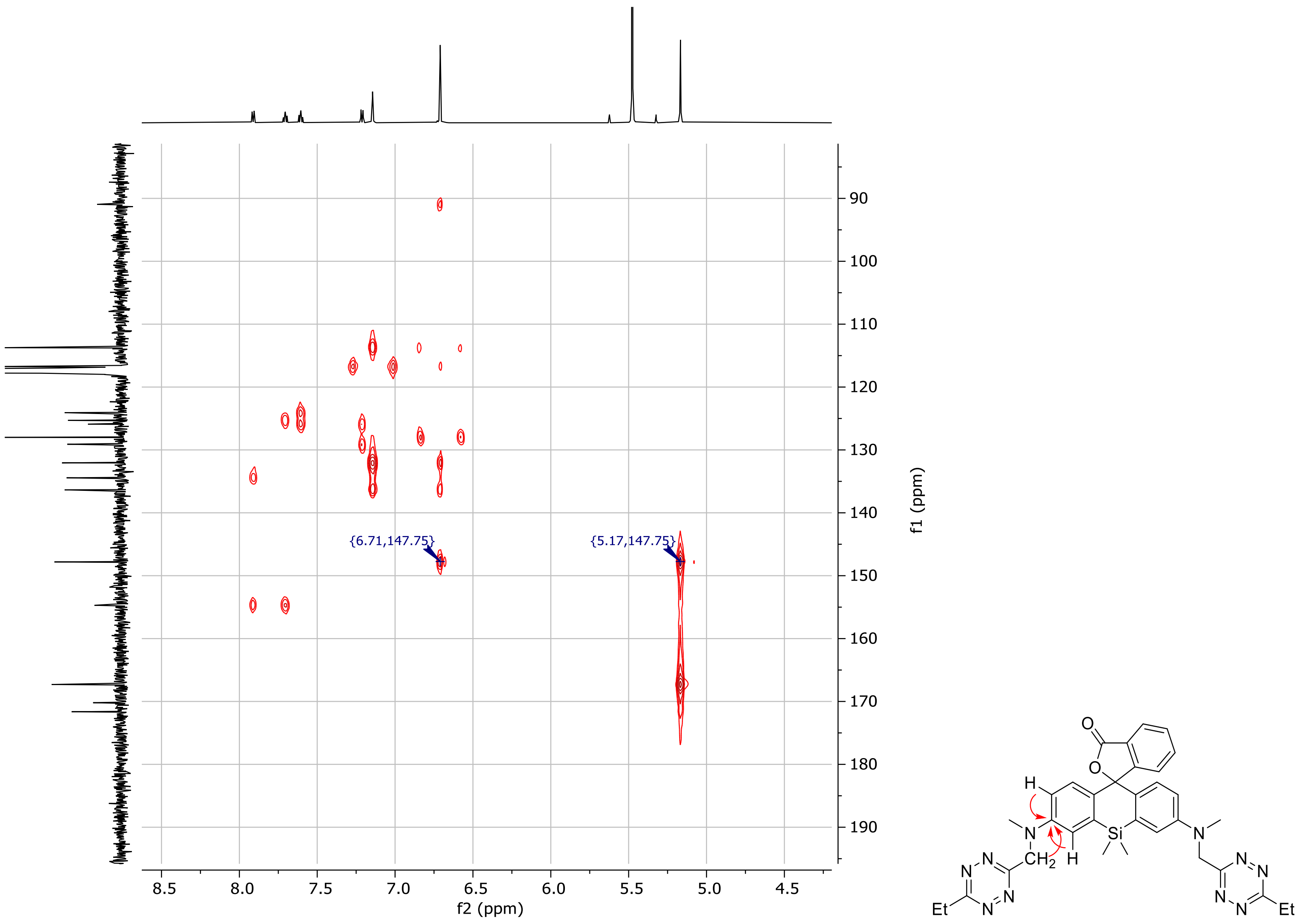

**$^{13}$C NMR compound SiRh640bisCTet**

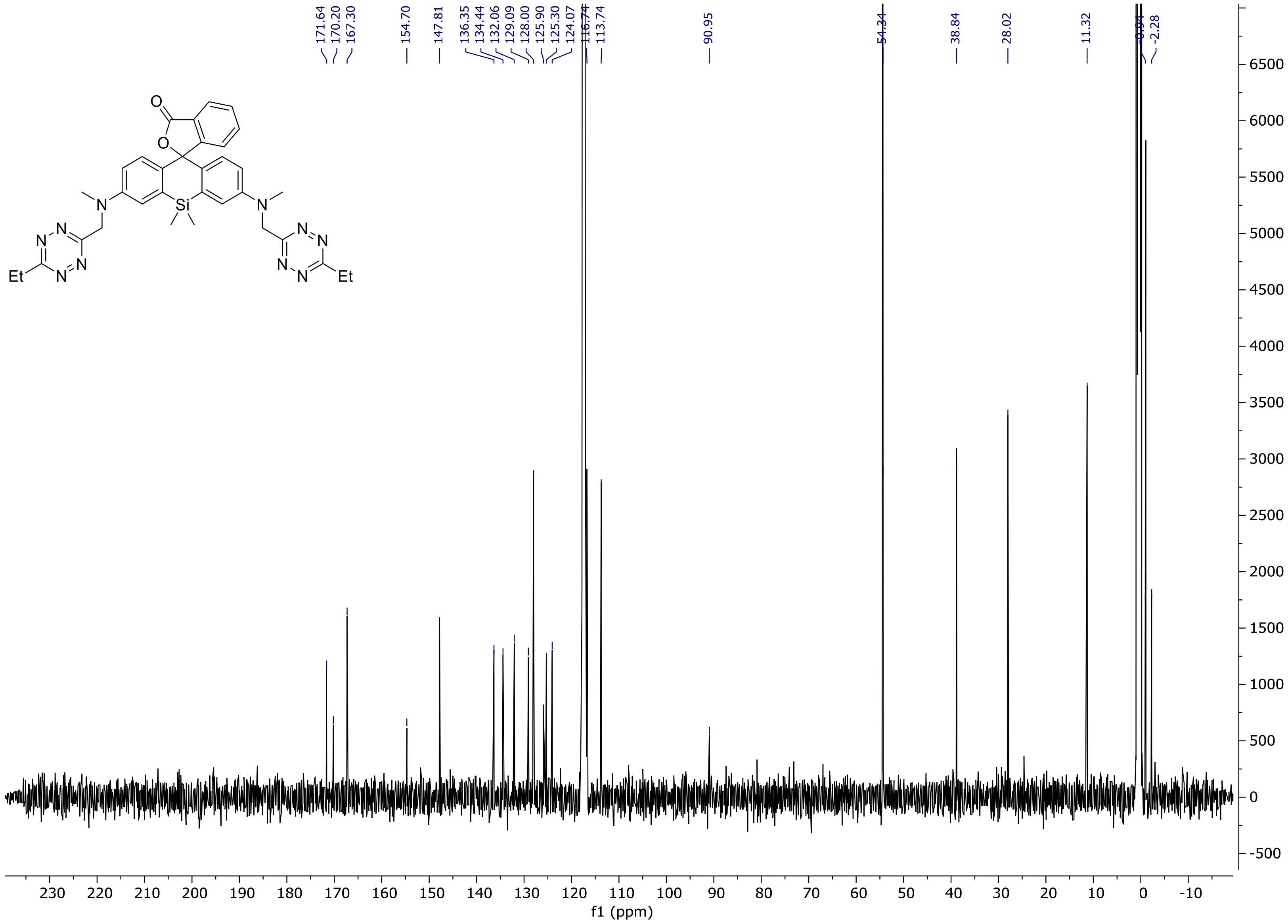

## $^{1}$H NMR compound SiRh634monoC3Tet

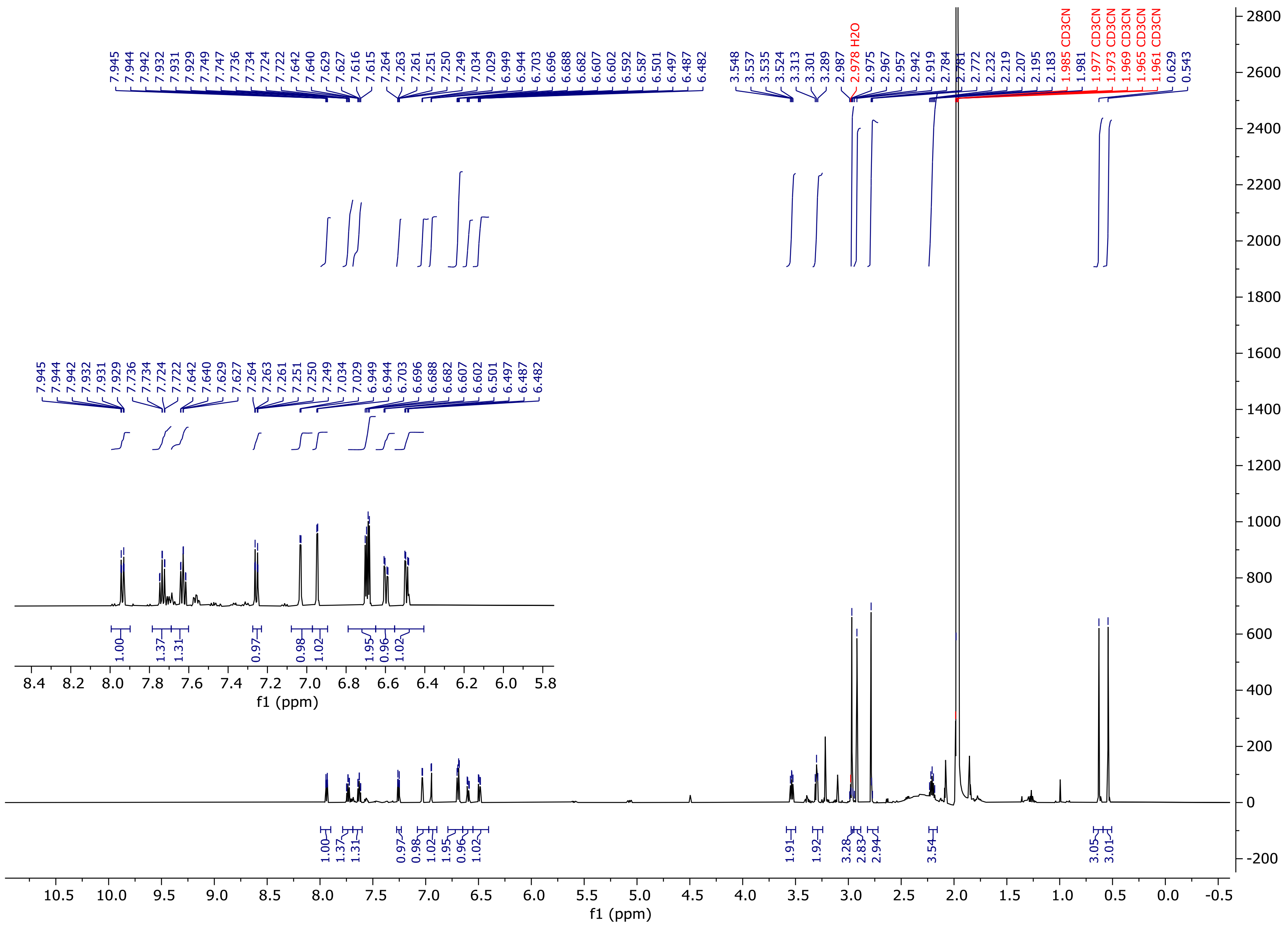

**$^{1}H$-$^{13}C$ HMBC 2D-NMR compound SiRh634monoC3Tet**

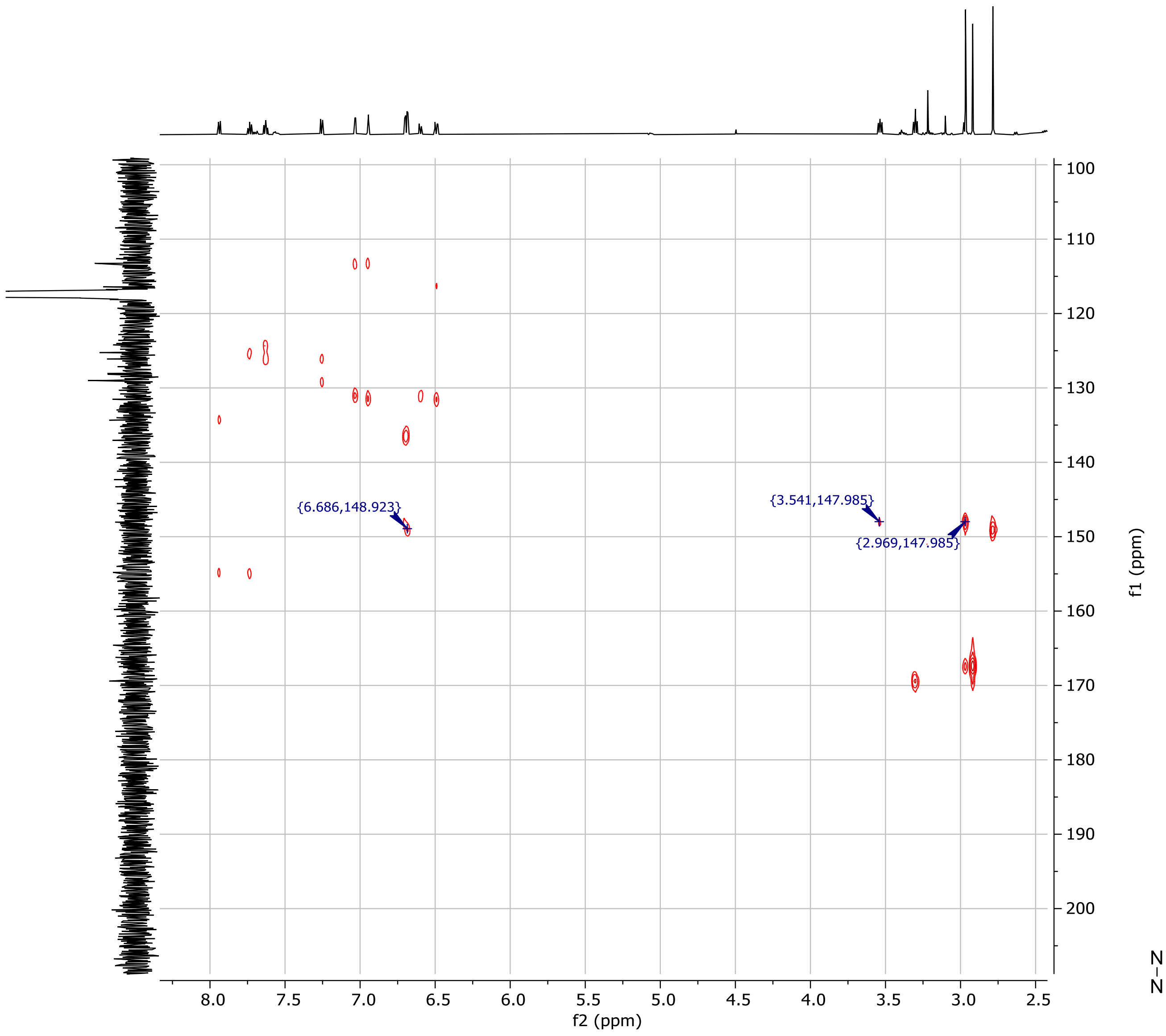


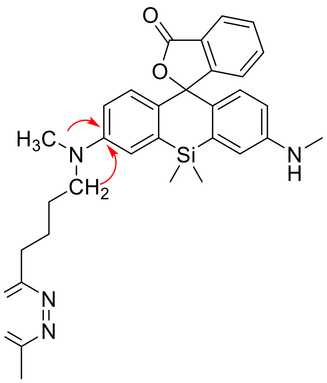

Supplement: Supplementary file 1 [file ja6c04723_si_001.pdf]
